# Genetic and pathway complexity in Alzheimer’s disease: Insights from multi-omic data about the immune response and mitochondrial function

**DOI:** 10.4103/NRR.NRR-D-25-00184

**Published:** 2025-09-29

**Authors:** Xuan Xu, Jiang Li, Fei Wang, Ke Xue, Junwen He, Xiangyu Meng, Yin Shen

**Affiliations:** 1 School of Life Sciences, Anhui Medical University, Hefei, Anhui Province, China; 2 Clinical Big Data Research Center, The Seventh Affiliated Hospital, Sun Yat-sen University, Shenzhen, Guangdong Province, China; 3 School of Basic Medical Science, Anhui Medical University, Hefei, Anhui Province, China; 4 College of Informatics, Huazhong Agricultural University, Wuhan, Hubei Province, China; 5 Health Science Center, Hubei Minzu University, Enshi, Hubei Province, China; 6 School of Biomedical Engineering, Anhui Medical University, Hefei, Anhui Province, China

**Keywords:** Alzheimer’s disease, DNA methylation, epigenetic, gene regulatory networks, machine learning, mitochondrial dysfunction, multi-omics analysis, nerve regeneration, neuroinflammation

## Abstract

Despite recent developments, the genetics and biology of Alzheimer’s disease remain insufficiently characterized. As an important first step toward developing effective treatment strategies to slow or prevent Alzheimer’s disease onset, the identification of relevant genetic markers is crucial. In the present study, we analyzed transcriptomic and multi-omic datasets across multiple cohorts (the Alzheimer’s Disease Neuroimaging Initiative, Religious Orders Study and Rush Memory and Aging Project, Mount Sinai Brain Bank, and Mayo Clinic Alzheimer’s Disease Genetics Studies) using gene set enrichment analysis, machine learning algorithms, and polygenic risk scoring to identify gene sets relevant to Alzheimer’s disease risk and pathological features. For prioritized gene sets, we performed epigenome-wide association studies to assess DNA methylation patterns, and used multi-omic mediation analysis to characterize the causal gene regulatory networks. Overall, we identified several key gene sets relevant to Alzheimer’s disease pathology—particularly, those related to immune system function and mitochondrial dysfunction. Upregulated pathways, including neutrophil degranulation and tumor necrosis factor-α signaling pathways, correlated strongly with aspects of neuroinflammation in Alzheimer’s disease. By contrast, downregulated oxidative phosphorylation pathways further suggested mitochondrial dysfunction. Gene sets that contained mitochondrially located genes (e.g., *SGK1* and *LRRK1*) were identified as significantly contributing to neurodegeneration. Moreover, genes such as *CXCL1*, *TGFB2*, and *DUSP1* were consistently implicated in all datasets, thus emphasizing their involvement in immune modulation and mitochondrial function. The multimodal investigation outlined in the current study represents useful steps toward comprehending the genetic architecture of Alzheimer’s disease, including an expanded understanding of the spatial interactions of genes associated with disease susceptibility. Mitochondrial dysfunction and immune modulation were pathological pathways that converged on Alzheimer’s disease and future treatment novel options. Using the frameworks provided in the current comprehensive study, we present opportunities to explore targeted treatment strategies that may alter immune systems and mitochondrial function to optimize treatment outcomes for individuals at increased risk of or living with Alzheimer’s disease.

## Graphical abstract

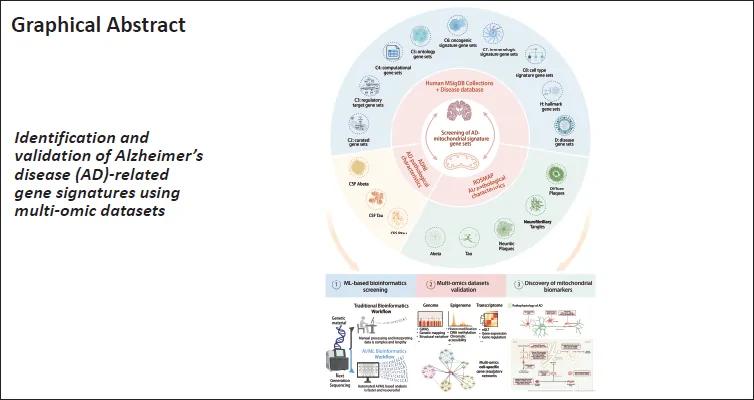

## Introduction

Alzheimer’s disease (AD) is a progressive neurodegenerative disorder that is characterized by cognitive decline, primarily in the aging population (Andrews et al., 2023). Its multi-factorial etiology arises from complex interactions among genetic, environmental, and biological factors, thereby presenting considerable challenges for neuroscience and aging research (Dunn et al., 2019). As the world continues to age, the motivation to better understand the mechanisms involved in AD has heightened because of the negative social, economic, and personal impacts of this disease (Zhang et al., 2021). Although advances have been made, important knowledge gaps remain regarding the genetics underlying AD as well as the biological pathways by which these genetics predetermine subsequent phenotypes. For example, how do AD-associated mutations in genes including *APOE*, *PSEN1*, and *PSEN2* lead to processes, such as the accumulation of amyloid-β (Aβ) and tau proteins, that are pathological features of AD (Dourlen et al., 2019; Kunkle et al., 2019)? In addition, what are the overall signaling pathways—perhaps involving the immune response, mitochondrial involvement, and other pathways—that link these genetic elements to neuronal death and cognitive impairment (Seto et al., 2021; Abubakar et al., 2022; Gureev et al., 2022)? These factors need to be determined to develop future therapies that treat, delay, or prevent AD.

Some aspects of the genetic and molecular mechanisms of AD have already been studied. For example, we know that *APOE* gene variants—specifically, those with the ε4 genotype—cause increased Aβ accumulation, and that *PSEN1* and *PSEN2* mutations alter the processing of amyloid precursor protein and lead to faster plaque formation (Andrews et al., 2023). Additionally, toxic hyperphosphorylated tau, which also accumulates in the brain in AD, is responsive to both genetic and environmental factors that contribute to a widespread increase in neurofibrillary tangles (NFTs) and environments conducive to neuronal death (Pluta and Czuczwar, 2024). Simultaneously, the advent of machine learning and artificial intelligence applications in AD research has leveraged the use of predictive models to forecast disease progression and even identify and characterize biomarkers. For example, some predictive models have highlighted the benefits of combining imaging and genomic data to forecast longitudinal cognitive decline (Zhou et al., 2021), whereas other multi-omic studies have systematically examined the molecular networks across AD progression to develop staging classifiers (Chiu et al., 2022). However, despite progress in the field, important challenges remain for developing system-level models that include genomics, transcriptomics, and epigenomics (Kavitha et al., 2022).

Given the current gaps in our understanding of how different genetic and molecular components contribute to AD pathogenesis, the goals of the present study were to systematically identify and characterize the gene sets and regulatory pathways that influence the susceptibility to and progression of AD. On the basis of our prior knowledge that implicates tau protein and genes in accumulation of tau protein, we believe that these as well as other genetic variants are likely acting upon complex biological networks—such as representative networks that are associated with the immune response, mitochondrial involvement, and neuronal signaling—to provide a basis for AD pathology.

**Figure 1:**
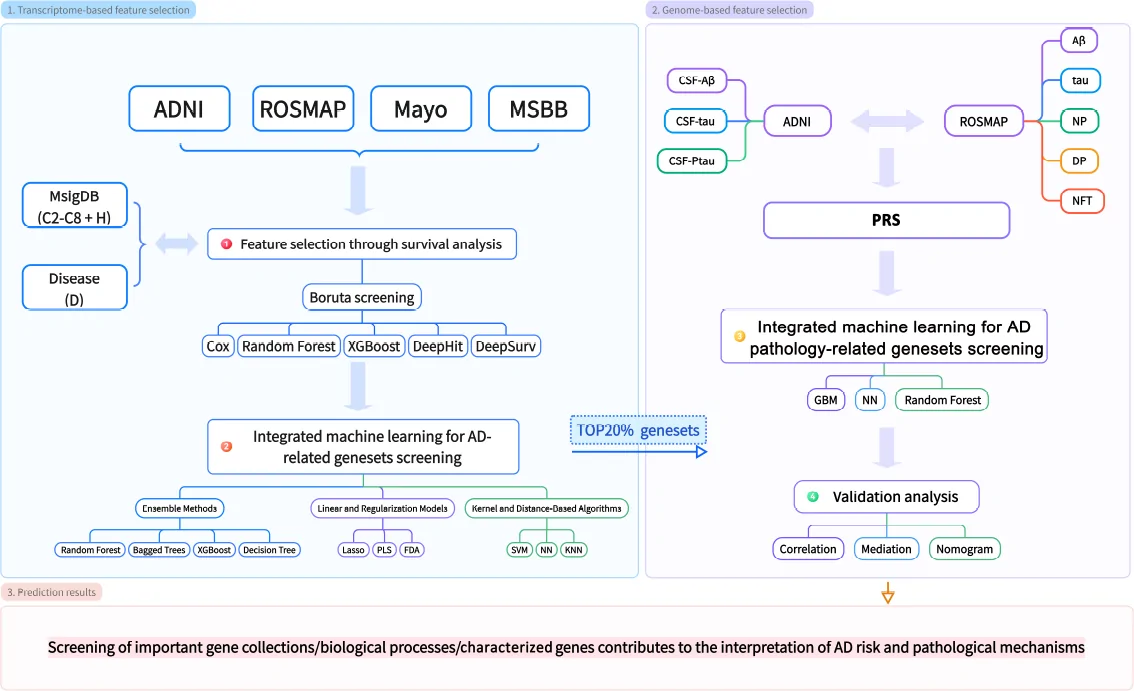
Multi-omics machine learning framework for AD pathway analysis. Our study integrates multiple data sources (MSigDB, ADNI, ROSMAP, Mayo, and MSBB cohorts) with polygenic risk scores (PRS) and CSF-tau biomarkers to investigate the genetic architecture of AD. The analytical pipeline incorporates diverse machine learning approaches, including deep learning models (DeepHit, DeepSurv), ensemble methods (Random Forest, Bagged Trees, XGBoost), and conventional algorithms (Decision Trees). This comprehensive computational framework enables systematic identification of AD-associated gene sets and biological pathways, providing insights into disease risk factors and pathological mechanisms. AD: Alzheimer’s disease.

To test this hypothesis, we used an integrative multi-omic approach to combine genomic, transcriptomic, and epigenomic data from four large AD cohorts. Our machine learning framework, which was applied across independent populations, aimed to: (1) identify functional gene sets associated with AD-related pathological elements, (2) prioritize candidate genes that can influence the progression or onset of the disease, and (3) examine the epigenetic regulatory mechanisms that modulate these gene networks. This work expands on our knowledge and understanding of the molecular basis of AD and provides actionable insights for future precision diagnostics and interventions. A more detailed description of the analysis and validation approach is shown in **Figure 1**.

## Methods

### Data sources

Transcriptomic and phenotypic data about AD were obtained from the RNA-sequencing Harmonization Study and the AD Neuroimaging Initiative (ADNI). The RNA-sequencing Harmonization Study standardized its processing pipeline across three well-characterized AD cohorts: the Religious Orders Study and Rush Memory and Aging Project (ROSMAP) (De Jager et al., 2018), the Mount Sinai Brain Bank (MSBB), and the Mayo Clinic AD Genetics Studies (Mayo). The ROSMAP project is nested within two prospective cohort studies that began in 1994 and 1997 and were designed to examine health outcomes, especially in terms of AD dementia and cognitive impairment risk factors. The MSBB cohort includes 364 brain specimens obtained from the Mount Sinai/JJ Peters VA Medical Center Brain Bank (MSBB-Mount Sinai National Institutes of Health Neurobiobank, https://dss.niagads.org/cohorts/mount-sinai-brain-bank-msbb/) (Wang et al., 2018a), and robust inclusion and exclusion criteria were used to capture the complete AD spectrum. The Mayo dataset (https://adknowledgeportal.synapse.org/Explore/Studies/DetailsPage/StudyDetails?Study=syn5550404) includes post-mortem temporal cortex and cerebellum samples from individuals diagnosed with AD, progressive supranuclear palsy, pathological aging, and non-demented controls (Allen et al., 2018). Braak staging, tau pathology, and cognitive ratings across all studies were used to ensure diagnostic consistency. These datasets came from the Accelerating Medicines Partnership (AMP)-AD Knowledge Portal (https://www.synapse.org). The ADNI is a longitudinal, multi-center study that aims to identify and validate biomarkers for AD (Saykin et al., 2015), and includes RNA profiling data from 811 subjects across cognitively normal (CN), mild cognitive impairment (MCI), and AD cohorts. Information regarding the ADNI can be found at http://www.adni-info.org/.

To evaluate the associations among selected genes that are involved in a variety of biological processes, we conducted gene set enrichment analysis (GSEA). The GSEA was performed using 34 535 gene sets presented in the Molecular Signatures Database (MSigDB, https://www.gsea-MSigDB.org/gsea/MSigDB/) (Liberzon, 2014). The MSigDB is a core bioinformatic resource that was constructed to provide gene sets annotated to different biological functions, organized by Clusters C2–C8 and H. The H category represents a specific biological state or biological process that comprises genes with coherent expression profiles; the different biological functions can include the immune response, cell cycle regulatory genes, and cellular metabolic functions. Gene–disease associations were derived from the DISEASES database (https://diseases.jensenlab.org/Search) (Grissa et al., 2022). Disease-related genes were selected at a disease knowledge maximum of five in the Knowledge channel. Genes localized to mitochondria were selected based on information from the COMPARTMENTS database (https://compartments.jensenlab.orgSearch) (Binder et al., 2014), which includes information about where genes are localized at a subcellular level. From the COMPARTMENTS database, we considered genes that had a knowledge channel score > 3 as reliable mitochondrially localized genes. Overall, we selected 1474 genes that met these criteria to be included in our study as mitochondrial localization candidates. The specific screening criteria are described in detail in our previous research (Xu et al., 2022, 2024).

### Pathological measurements of Alzheimer’s disease

In the ADNI cohort, cerebrospinal fluid (CSF) concentrations of Aβ and tau proteins were measured using electrochemiluminescence immunoassay. Of the 1550 samples with genotype data, Aβ_42_ was quantified in 1128 samples, whereas phosphorylated tau (p-tau) and total tau were measured in 1127 samples. In the ROSMAP cohort, quantitative assessments were provided for five AD pathological markers, including neuritic plaques (NPs), diffuse plaques (DPs), and NFTs across five brain regions: the middle frontal cortex, middle temporal cortex, inferior parietal cortex, internal olfactory cortex, and hippocampus. Counts were obtained via silver dip staining, and the square root of the mean across these regions was calculated to evaluate the severity of AD pathology (Bennett et al., 2012a, b). The additional quantification of Aβ and p-tau was performed in eight brain regions: the hippocampus, internal olfactory cortex, middle frontal cortex, inferior temporal lobe, angular gyrus, calcarine cortex, anterior cingulate cortex, and superior frontal cortex. Immunohistochemical staining and image analysis with stereological methods were used to measure the percentage area occupied by Aβ and the density of tau tangles. Average values across these regions were then used to estimate the overall cerebral levels of Aβ and p-tau. The pathological phenotypic characteristics of participants in the ADNI, ROSMAP, MSBB and Mayo cohorts are summarized in **Additional Table 1**.

### Feature selection through survival analysis

To identify the variables that are significantly associated with AD status (AD *vs*. CN), we used a multi-step feature selection approach. Initially, the Boruta algorithm—a feature selection method based on random forests (RFs)—was applied to each dataset to screen for differential genes. Next, a survival analysis model was constructed using five algorithms: the Cox proportional hazards model, RF, XGBoost, and neural networks (NN; DeepSurv and DeepHit). These algorithms were used to assess the effects of selected variables on AD risk, with AD diagnosis as the endpoint and age at AD onset as the survival time. Model performance was evaluated using the concordance index, Brier score, and area under the concordance/discrimination curve. The three top-performing algorithms were chosen using variable importance aggregation. The overall importance score (*IMP*_i_^all^) of variable *i* was determined by the weighted average of its importance scores from these three models, with weights proportional to the performance ranks of the models, as follows:

*IMP*_i_^all^ = (*Model1*_i_^imp^ × 0.42) + (*Model2*_i_^imp^ × 0.33) + (*Model3*_i_^imp^ × 0.25)

Where *Model1*_i_^imp^, *Model2*_i_^imp^, and *Model3*_i_^imp^ represent the importance scores of variables *i* in the three top-ranked models. This variable selection was performed using the R packages mlr3verse (Lang et al., 2024a), mlr3extralearners (Sonabend and Schratz, 2025), mlr3proba (Sonabend et al., 2021), mlr3measures (Lang et al., 2024b), and survivalmodels (Sonabend and Foucher, 2024).

### Integrated machine learning framework for Alzheimer’s disease–related gene set identification

To construct predictive models, we selected the top 20% of variables according to their aggregated importance from the survival analysis. To predict AD status across different cohorts, we implemented 10 machine learning algorithms, which we categorized into three groups: ensemble methods, linear and regularization models, and kernel and distance-based algorithms.

1. Ensemble methods: these methods aggregate the outputs from multiple models to enhance predictive performance.

• RF: constructs multiple decision trees, aggregating their outputs (using a majority vote for classification or mean prediction for regression) to improve robustness.

• Decision tree: uses data-derived decision rules for classification; although prone to overfitting, it forms the basis for many ensemble approaches.

• Bagged trees: reduces variance by averaging predictions from decision trees trained on bootstrap samples.

• XGBoost: an improved gradient boosting method with regularization, designed for large datasets to avoid overfitting.

2. Linear and regularization models: these models are commonly used to learn linear relationships and can be used for feature selection through regularization.

• Least absolute shrinkage and selection operator: a linear model with regularization, designed to model a sparse model by estimating a coefficient of 0 for the least important predictors.

• Partial least squares: relates component-identifying predictors that maximize covariance with the response and prediction, and has methods to deal with collinearity among predictors.

• Flexible discriminant analysis: a linear classification method that combines features to best separate classes.

3. Kernel and distance-based algorithms: these models are particularly well-suited to capturing linear relationships and performing feature selection through regularization.

• Support vector machine (SVM): finds an optimal hyperplane that maximizes the margin between classes.

• NN: inspired by brain architecture, these models capture complex, non-linear patterns through multi-layer architectures.

• K-nearest neighbors (KNN): classifies data based on the majority class among nearest neighbors in feature space.

We standardized the model-building process using the tidymodels framework in R. Each model was optimized through a grid search with 30 hyperparameter combinations, and was evaluated using 10-fold cross-validation to ensure robustness. Performance metrics included the area under the receiver operating characteristic curve (AUC) and accuracy. For top-performing models, feature importance was assessed using the DALEXtra (Biecek, 2018) R package, which extends the DALEX (Biecek, 2018) package to interpret various machine learning models. Feature importance scores were then aggregated across individual models and weighted by model performance rankings to identify key contributors to AD prediction (**Figure 1**).

### Pathway enrichment analysis and protein–protein interaction network analysis for Alzheimer’s disease-related genes

We performed pathway enrichment analysis and protein–protein interaction (PPI) network analysis for differentially expressed genes (DEGs) and important gene sets; this was made possible with the curated datasets and specified thresholds using the pathlinkR package in R (Blimkie et al., 2024). Next, the identified pathways were mapped against gene sets from the Reactome and Hallmark datasets with a significance threshold of *P* < 0.05 and a fold-change threshold of > 0.5 to identify pathways with strong associations with the relevant genes of interest. This provided us with biologically interesting pathways that were enriched within the relevant gene set—especially, any pathways relevant to AD. High-confidence PPI networks were then created using the STRING database (https://string-db.org) (Szklarczyk et al., 2021) with an interaction score threshold of 700, which included only the highest confidence interactions. We also mapped the DEGs onto PPI networks to flag potential regulatory interactions associated with AD. The visualization depicted the expression status of the genes by node color (upregulated genes: pink, downregulated genes: purple), emphasizing the marked differences in protein interactions that included pathways associated with AD and regulatory mechanisms. This integrative approach provided a focused view of the enriched pathways and high-confidence interactions while also highlighting the functionally relevant relationships associated with AD pathology.

### Gene set polygenic risk score calculations

Polygenic risk score (PRS) quantify the genetic susceptibility of an individual to traits or diseases by aggregating the contributions of multiple genetic variants. The calculation began by identifying trait-associated single nucleotide polymorphisms (SNPs) from genome-wide association study (GWAS) data. Genotype data were then subjected to quality control using PLINK software (v1.90b4.10) (Chang et al., 2015). The PRS was then calculated by summing the products of the effect sizes (denoted as *A*_i_) from GWAS and the corresponding number of risk alleles (denoted as *G*_ij_) for each individual *j* across all relevant SNPs *i*. This can be expressed mathematically as:


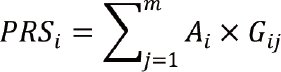


where *m* is the summative number of SNPs related to the trait. This score is then standardized, and the predictive capacity of the score is assessed using regression modelling. PRSice-2 (Euesden et al., 2015) was used to calculate the PRS score, and standardization was conducted using the methods outlined in our previous publications (Xu et al., 2022, 2024), including the descriptions of quality control and the parameters for calculating PRS scores. To score genetic risk potential, we implemented PRS models on the top 20% of gene sets across nine categories (C2–C8, H, and D) of top notable gene sets in the MSigDB based on overall significance. The PRS data were obtained from a recent AD GWAS with a sample size of 94,437 (Kunkle et al., 2019). Summary statistics for 10,538,401 SNPs were made available from stage 1, which included 21,982 AD cases and 41,944 controls. The SNPs included in the PRS models adhered to genes in gene sets from the top 20% of significance.

### Integrated machine learning for Alzheimer’s disease pathology–related gene set screening

We used the caret R package to construct gradient boosting machine, NN, and RF models to predict eight pathological phenotypes associated with AD. When training the models, we conducted grid search optimization to tune the hyperparameters to further improve predictive accuracy. We used cross-validation to evaluate the performance of each model based on root mean squared error and *R*^2^ criteria. To interpret the feature contributions of individual features, we used the DALEX package and DALEXtra extension to visualize and perform detailed feature importance analysis across complex models. We then calculated feature root mean squared error scores across the models and weighted them based on performance ranking by their inverse relationship to RMSE values, to emphasize the contributions from models with the best predictive accuracy.

### Epidemiological data description and pre-processing

For the present study, methylation profiling was performed on ADNI and ROSMAP datasets. The ADNI dataset used the Illumina EPIC array (Illumina, San Diego, CA, USA) to analyze 1720 samples from 653 subjects across AD, MCI, and CN groups, covering approximately 866,000 CpG sites. A stratified, randomized incomplete block design ensured that all samples from a given subject were placed on a single chip, with the remaining chip space allocated to age-matched subjects of the opposite sex. Technical replicability was evaluated using duplicate DNA samples across chips, resulting in 1920 processed samples (**Additional Table 1**). Subject selection was based on two experimental factors: (1) longitudinal data availability (subjects with two or more time points); and (2) diagnosis progression (subjects transitioning from CN to MCI, CN to AD, or MCI to AD). Samples that underwent technical replication but were excluded from the final analysis included 199 duplicates and one triplicate. In the ROSMAP cohort, DNA was extracted from frozen dorsolateral prefrontal cortex tissue from 761 deceased participants, and methylation profiling was performed using the Illumina Infinium HumanMethylation450 BeadChip. Quality control measures involved the exclusion of low-quality probes and samples, with adjustments for batch effects (De Jager et al., 2014).

Raw methylation data (idat files) were imported into R and preprocessed using the Bioconductor bigmelon package, which calculated detection P-values, methylation, and unmethylation intensities from which beta values were derived. Processed data underwent further analysis using the limma and ChAMP pipelines (Tian et al., 2017). Probes were filtered based on the following criteria: (1) detection *P*-values > 0.01; (2) < 3 beads in ≥ 5% of samples; (3) non-CpG probes (Chen et al., 2013); (4) probes linked to SNPs; (5) probes with multiple binding targets; and (6) probes on sex chromosomes (Zhou et al., 2017). Batch effects were adjusted using ComBat (Johnson et al., 2007). Cellular proportions were estimated using reference DNA methylation maps, and adjustments for cell-type influence on whole blood data were made using RefbaseEWAS (Houseman et al., 2012). The “estimateCellCounts” function in the minfi R package (Aryee et al., 2014) was used to quantify blood cell type proportions in each sample, accounting for inter-group variations in probe intensities.

### Epigenome-wide association analysis

The epigenome serves as a dynamic interface between genetic predisposition and environmental influences. Although DNA sequences remain stable, epigenetic modifications exhibit plasticity that may contribute to disease pathogenesis. In AD, these modifications represent potential therapeutic targets because of their reversible nature. Our investigation used pigenome-wide association analysis (EWAS) methodology to systematically profile the DNA methylation patterns associated with AD. With this in mind, we used the R package “meffil” (Min et al., 2018) to ensure quality control, and performed EWAS analysis by fitting linear regression models based on the “limma” package to assess methylation sites that are related to AD risk. In our analysis, we evaluated nominal associations between DNA methylation and AD while accounting for potential confounders that may inflate variability (age, sex, date of testing, education levels, and cellular composition) to account for methylation pattern influences. “Meffil” has four models in general: (1) model without covariates, (2) model with covariates, (3) model with an extra step using existing surrogate variables (which are derived from surrogate variable analysis), and (4) model with variables from independent surrogate variable analysis. To increase the robustness of our results, we applied all four model types in the analysis. Given the disparities in sample sizes between cohorts, we used adjusted significance thresholds for each dataset (ADNI: *P* < 1 × 10^–7^, ROSMAP: *P* < 1 × 10^–5^).

### Multi-omic integration for analyzing cell-specific regulatory networks

We used single-cell gene regulatory network prediction from multi-omics (scGRNom) (Jin et al., 2021) to integrate multi-omic data for understanding the regulatory networks involved in AD, to generate networks specific to cellular data. scGRNom allowed us to use single-cell resolution to connect transcription factors (TFs) and regulatory features (enhancers and promoters) to their respective target gene(s). scGRNom was used to integrate chromatin interaction, epigenomic, and transcriptomic data from the major primary human brain cell types (excitatory and inhibitory neurons, microglia, and oligodendrocytes). The analysis pipeline can be divided into four main steps:

1. Chromatin interaction analysis: chromatin interaction data (e.g., Hi-C) was integrated to identify the potential interactions between enhancers and promoters within topologically associating domains. We used the GenomicInteractions R package (Harmston et al., 2015) to annotate these interactions and map them to genes, referencing genomic annotations from TxDb.Hsapiens.UCSC.hg19.knownGene.

2. Identification of TF binding sites (TFBS): potential TFBS within interacting enhancers and promoters were inferred. A preliminary GRN was constructed based on TFBS mapping, with position weight matrices obtained from the JASPAR database (https://jaspar.elixir.no/) using TFBSTools (Tan and Lenhard, 2016). This reference network linked TFs to interacting enhancers and promoters.

3. Refinement of regulatory network and pathway activity: elastic net regression was used to refine the initial GRN using TF expression levels to predict target gene expression. TFs with high regression coefficients were identified as key regulators. The final GRN comprised TFs with high coefficients, target genes, and enhancers filtered for high accessibility using single-cell assay for transposase-accessible chromatin sequencing data (http://resource.psychencode.org/), thus ensuring cell-type specificity.

4. Identification of cell type-specific disease genes and regulatory elements: cell type-specific GRNs were integrated with AD-associated SNPs from GWAS to identify the disease genes and regulatory elements unique to each cell type. The GenomicRanges R package (Lawrence et al., 2013) was used to identify any overlap between disease-associated SNPs and regulatory elements, whereas motifbreakR (Coetzee et al., 2015) was used to identify SNPs that potentially disrupt TFBS on enhancers and promoters. Affected genes were classified as cell type-specific AD-related genes. Chromatin interaction and enhancer data were sourced from Nott et al. (2019), and single-cell gene expression data came from the ROSMAP project (syn18485175) (Mathys et al., 2019).

### Ethics approval and consent to participate

The data used in the current study were obtained from four publicly available cohorts: ADNI, ROSMAP, MSBB, and Mayo. All participants or their legally authorized representatives provided written informed consent at the time of enrollment. Each original study was approved by the relevant institutional review boards at the respective institutions. Our research adheres to the terms outlined in the data use agreements. Specifically, data were accessed through the AD Knowledge Portal (https://adknowledgeportal.org), which governs access to the ADNI, ROSMAP, MSBB, and Mayo datasets. Detailed information on the ethical approvals and data access procedures can be found on the websites of ADNI (https://adni.loni.usc.edu/) and ROSMAP (https://www.radc.rush.edu/). For the MSBB and Mayo databases, ethical protocols were established through the AMP-AD program under the guidance of the respective institutions.

## Results

### Screening for Alzheimer’s disease risk–related markers

**Figure 2:**
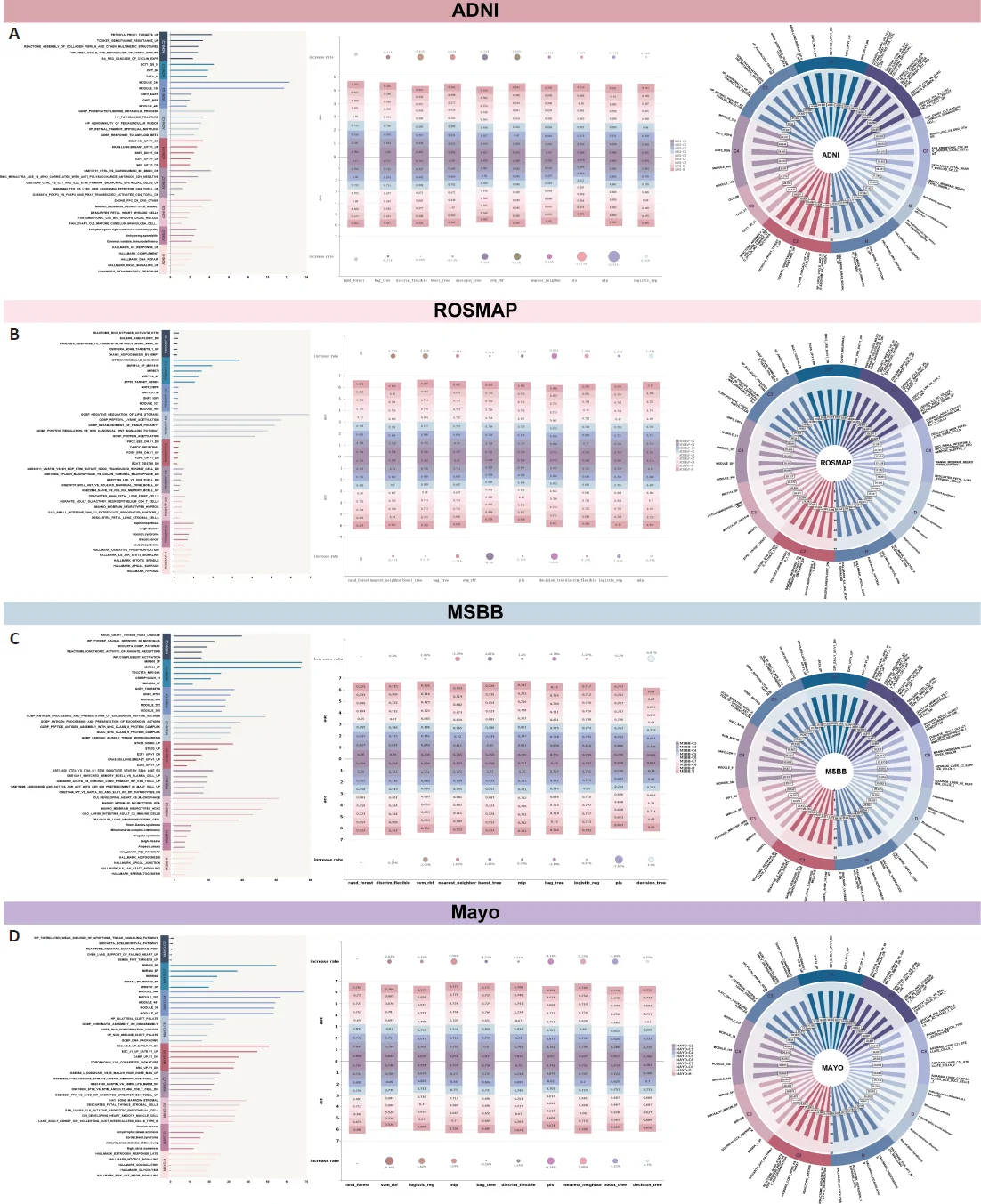
Systematic evaluation of gene set categories in the AD-related dataset. This figure summarizes the analysis of nine distinct gene set categories (C2–C8, H, and D from the MSigDB and DISEASE databases) across four major AD cohorts: AD Neuroimaging Initiative (ADNI; A), Religious Orders Study and Rush Memory and Aging Project (ROSMAP; B), Mount Sinai Brain Bank (MSBB; C), and Mayo Clinic AD Genetics Studies (Mayo; D). Through survival analysis modeling, the following number of significant AD risk-associated gene sets were identified: 68 (ADNI), 200 (ROSMAP), 484 (MSBB), and 716 (Mayo), with statistical significance. The figure presents the five highest-ranking gene sets per category. Among the ten evaluated machine learning models, the Random Forest (RF) model demonstrated the highest predictive accuracy across all the datasets. The highest area under the receiver operating characteristic curve (AUC) values observed were 0.734 (C5) in ADNI, 0.794 (C2) in ROSMAP, 0.780 (C2) in MSBB, and 0.846 (C2) in Mayo. The variable importance assessment across the integrated machine learning model identified the highest-ranked gene sets across all categories: *C7_GSE40274 FOXP3 vs. PBX1 activated CD4 T cells downregulated* (*IMP*^all^ = 29.165) in the ADNI, *D_Joubert syndrome* (*IMP*^all^ = 30.285) in ROSMAP, *C7_PBMC APSV Wetvax Age 18–40* (*IMP*^all^ = 30.590) in MSBB, and *C4_Module 229* (*IMP*^all^ = 30.868) in Mayo. A comparison across all four datasets indicated that MSBB and Mayo presented the highest intersection of important variables in the C6 category, with 13 common features. The gene sets with the highest significance in each category were *C2_Reactome Keratan Sulfate Degradation* (*IMP*^all^ = 58.014), *C3_MIR6794 5P* (*IMP*^all^ = 57.485), *C4_Module 229* (*IMP*^all^ = 30.868), *C5_GOCC DNA Packaging Complex* (*IMP*^all^ = 30.540), *C6_Cahoy Neuronal* (*IMP*^all^ = 89.410), *C7_GSE21546 WT versus SAP1A KO ELK1 KO Thymocytes Downregulated* (*IMP*^all^ = 59.149), *C8_HAY Bone Marrow Stromal* (*IMP*^all^ = 58.859), *H_Hallmark IL6 JAK STAT3 Signaling* (*IMP*^all^ = 57.960), and *D_Rheumatoid Arthritis* (*IMP*^all^ = 58.699). AD: Alzheimer’s disease.

To identify gene sets associated with AD risk across multiple datasets (C2–C8, H, and D from the MSigDB and DISEASE databases), we conducted a comprehensive screening based on survival models and machine learning approaches (**Additional Figures 1** and **2**). We identified 68, 200, 484, and 716 gene sets associated with AD risk in the ADNI (**Figure 2A**), ROSMAP (**Figure 2B**), MSBB (**Figure 2C**), and Mayo (**Figure 2D**) datasets, respectively. The top five gene sets with the highest importance scores in each category are presented in **Figure 2** (left). Among the 10 machine learning models that were evaluated for predictive performance, the RF model demonstrated the highest accuracy. The AUC values in each dataset were 0.734 (C5) in ADNI, 0.794 (C2) in ROSMAP, 0.780 (C2) in MSBB, and 0.846 (C2) in Mayo (**Figure 2**, middle; **Additional Table 2**). An analysis of variable importance from the composite machine learning model revealed the following top-ranked gene sets: for ADNI, C7_GSE40274 FOXP3 *vs* PBX1 Activated CD4 T Cell Downregulated (*IMP*^all^ = 29.165); for ROSMAP, D_Joubert Syndrome (*IMP*^all^ = 30.285); for MSBB: C7_ PBMC APSV Wetvax Age 18 to 40 (*IMP*^all^ = 30.590); and for Mayo: C4_Module 229 (*IMP*^all^ = 30.868) (**Figure 2**, right).

Cross-dataset analyses revealed that the most overlap occurred between MSBB and MAYO in the C6 categories, with 13 important overlapping variables. The variables with the highest general importance across categories included C2_Reactome Keratan Sulfate Degradation (*IMP*^all^ = 58.014), C3_MIR6794 5P (*IMP*^all^ = 57.485), C4_Module 229 (*IMP*^all^ = 30.868), C5_GOCC DNA Packaging Complex (*IMP*^all^ = 30.540), C6_Cahoy Neuronal (*IMP*^all^ = 89.410), C7_GSE21546 WT *versus* SAP1A KO ELK1 KO Thymocytes Downregulated (*IMP*^all^ = 59.149), C8_HAY Bone Marrow Stromal (*IMP*^all^ = 58.859), H_Hallmark IL6 JAK STAT3 Signaling (*IMP*^all^ = 57.960), and D_Rheumatoid Arthritis (*IMP*^all^ = 58.699) (**Additional Table 3**). These findings provide a foundational map of AD-associated gene sets that are linked to cognition, and validate the power of machine learning models for identifying predictive markers across cohorts.

**Figure 3:**
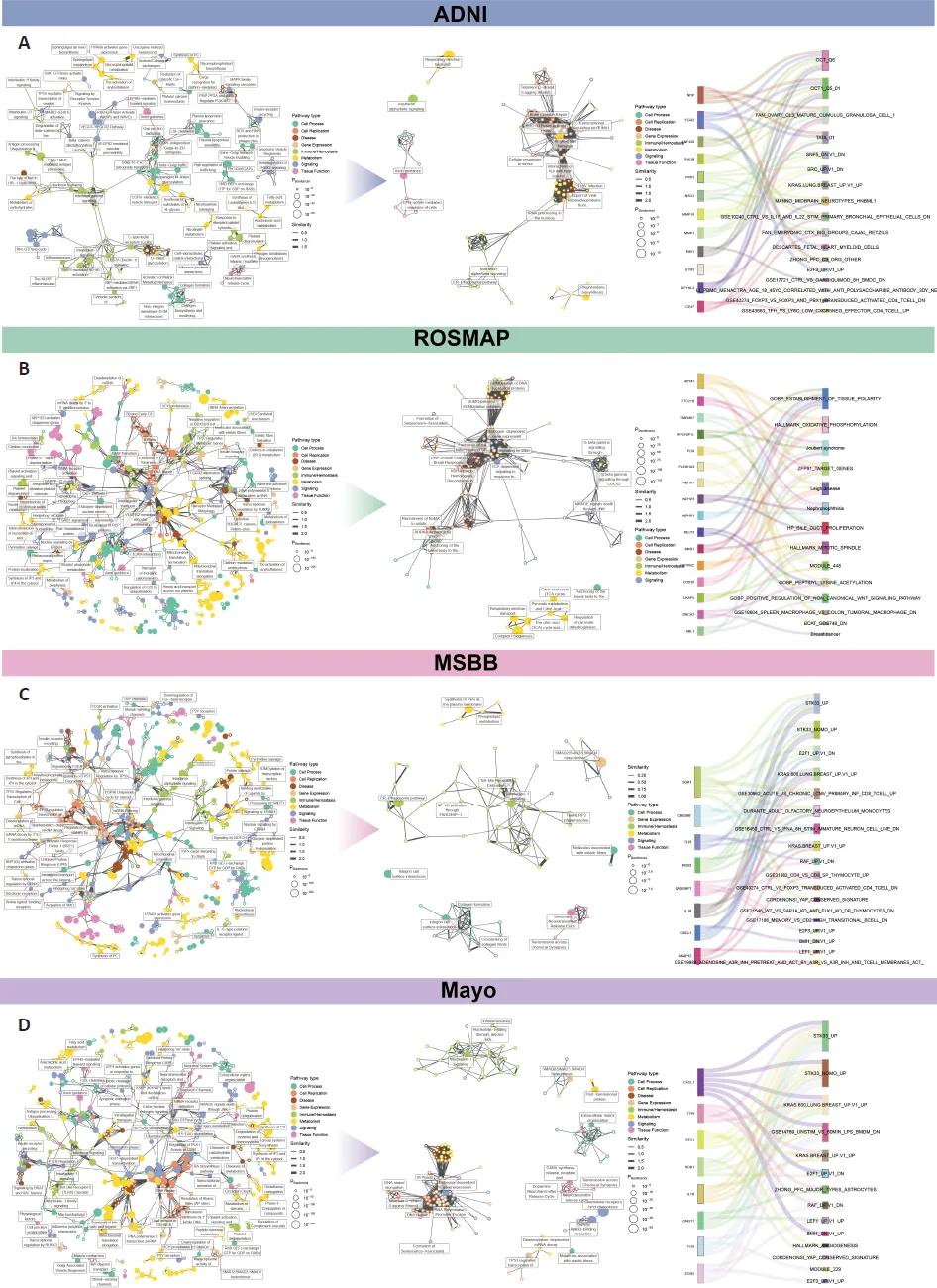
Differential gene expression networks reveal immune activation and metabolic dysregulation in AD across four cohorts. Differential expression analysis across four major AD cohorts—AD Neuroimaging Initiative (ADNI; A), Religious Orders Study and Rush Memory and Aging Project (ROSMAP; B), Mount Sinai Brain Bank (MSBB; C), and Mayo Clinic AD Genetics Studies (Mayo; D)—revealed distinct patterns of immune dysregulation and metabolic perturbation. The visualized pathways for each dataset depict shared and unique biological processes influenced by AD pathology. (A) ADNI analysis revealed that neutrophil degranulation (R-HSA-6798695) was the primary upregulated process, a feature that was consistent across all cohorts. The key hub genes identified through protein‒protein interaction (PPI) network analysis included *CDK2*, *E2F3*, *LRRK1*, *NFKB1*, and *HDAC1*, with *LRRK1* and *NFKB1* also identified as mitochondrial localization genes, indicating mitochondrial involvement in AD. (B) ROSMAP demonstrated significant downregulation of respiratory electron transport (R-HSA-611105), a pattern also observed in the MSBB and Mayo cohorts. The hub genes included *YWHAE*, *ECT2*, *HDAC5*, and *BRCA1*, with significant downregulation noted in the Complex I biogenesis pathway (R-HSA-6799198, *P* = 1.62 × 10^–133^). (C) MSBB analysis corroborated the upregulation of neutrophil degranulation. PPI analysis revealed hub genes such as *MYC*, *JUN*, *FN1*, *SQSTM1*, *LRRK2*, *BCL6*, and *NFKB1*, with LRRK2 and NFKB1 again being mitochondrially localized genes. Additionally, pathways related to interleukin-10 signaling and SMAD2/SMAD3:SMAD4 heterotrimer regulation of transcription were significantly upregulated. (D) The Mayo cohort exhibited significant upregulation of the chemokine receptor and binding pathway (R-HSA-380108, *P* = 2.25 × 10^–57^), which was the most significantly upregulated pathway. The hub genes included *JUN*, *CDKN1A*, *HSPA1A*, *VIM*, *MYC*, *BAG3*, and *NFKB1*, with *HSPA1A* and *NFKB1* also identified as mitochondrially localized genes, reinforcing the involvement of mitochondria in AD pathology in this cohort. Cross-cohort integration revealed conserved dysregulated processes, particularly immune activation and mitochondrial dysfunction. Notably, *JUN*, *MYC*, and *NFKB1* were common hub genes across multiple datasets, highlighting their central role in AD-related immune and metabolic pathways. AD: Alzheimer’s disease.

Gene expression analysis revealed immune dysfunction and metabolic dysregulation in the AD cohorts. To further characterize the functional consequences of the predicted gene sets in the respective cohorts, we conducted a series of differential expression analyses to expose patterns of dysregulation across biological processes and cell types. By combining the results of the enrichment analyses across all four datasets, we identified that neutrophil degranulation (R-HSA-6798695) was the most upregulated biological process, with the greatest frequency across all cohorts (ADNI, ROSMAP, MSBB, and Mayo). Conversely, the biological process with the most downregulated occurrences across cohorts and the greatest significance was respiratory electron transport (R-HSA-611105), which occurred with the greatest frequency in three datasets (ROSMAP, MSBB, and Mayo) (**Figure 3**, right and **Additional Table 4**). We also counted how many differentially expressed high-ranking genes were identified by searching universally, using categories from all datasets. The chemokine receptor and chemokine binding pathway in the Mayo dataset was the most significant (R-HSA-380108, *P* = 2.25 × 10^–57^). In the ROSMAP dataset, significant downregulation was detected in the complex I biogenesis pathway (R-HSA-6799198, *P* = 1.62 × 10^–133^). It is also worth noting that there were significantly upregulated processes, such as SMAD2/SMAD3: SMAD4 heterotrimer regulating transcription, interleukin-10 signaling, and molecules associated with elastic fibers in both the MSBB and Mayo datasets. Overall, the processes that exhibited significant upregulation mostly involved immune functions and signal transduction, whereas pathways and biological processes that were downregulated were focused on metabolism. Genes associated with the aforementioned processes from the occurring gene lists were *CXCL1*, *IL1B*, *SGK1*, and *TFPI*, which co-occurred in the MSBB and Mayo datasets. Notably, *EGR2* was detected in both the ADNI and Mayo datasets, and SGK1 was identified as a mitochondrially localized gene (**Figure 3**, middle; **Additional Table 4**).

Network analysis of PPI from InnateDB enabled us to detect hub genes through betweenness centrality. The identified hub genes for each dataset included the following: for ADNI, *CDK2*, *E2F3*, *LRRK1*, *NFKB1*, and *HDAC1*; for ROSMAP, *YWHAE*, *ECT2*, *HDAC5*, and *BRCA1*; for MSBB, *MYC*, *JUN*, *FN1*, *SQSTM1*, *LRRK2*, *BCL6*, and *NFKB1*; and for Mayo, *JUN*, *CDKN1A*, *HSPA1A*, *VIM*, *MYC*, *BAG3*, and *NFKB1*. In particular, *JUN*, *MYC*, and *NFKB1* were shared between the datasets. Among all hub genes, *LRRK1*, *NFKB1*, *LRRK2*, and *HSPA1A* were characterized as mitochondrially localized genes (**Figure 3**, right).

An assessment of the Hallmark gene sets covered by those identified genes revealed significant upregulation in the epithelial–mesenchymal transition, inflammatory response, and tumor necrosis factor (TNF)-α signaling via NFKB pathways, across all datasets. By contrast, oxidative phosphorylation showed significant downregulation in the same datasets. Of the screened important genes, the *KRAS* signaling up pathway (which occurred in the ADNI, MSBB, and Mayo datasets) was the most commonly identified in both the upregulated and downregulated gene sets. The TNF-α signaling via *NFKB* gene set also occurred in every dataset as a downregulated pathway. Furthermore, among all identified pathways, those related to signal transduction showed the greatest proportion of changes in both upstream and downstream processes (**Additional Figure 3** and **Additional Table 5**). Together, these results indicate consistent changes in the immune response and metabolic dysregulation across AD cohorts, thereby implicating both pathways in the development of the disease.

### Characteristic and functional analysis of key biological gene sets

**Figure 4:**
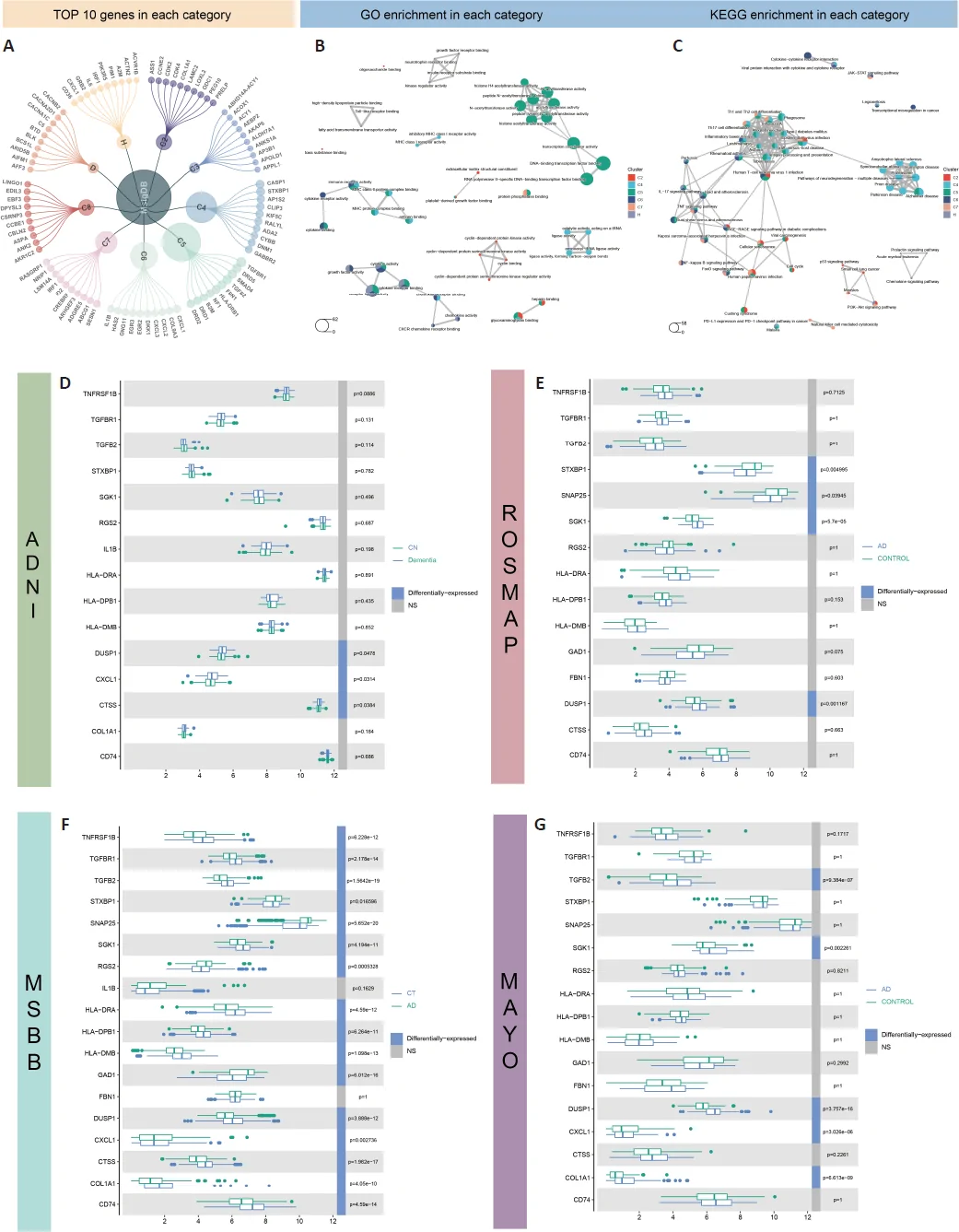
Integrative analysis of gene set enrichment and differential expression patterns across AD cohorts. A systematic evaluation of gene set significance across various Molecular Signatures Database (MSigDB) categories in AD was conducted. (A) Analysis identified the ten most prevalent genes within each cluster. Cluster C2 focused on cell cycle regulation (*CDK2*, *CDK4*); Cluster C3 emphasized lipid metabolism (*ABHD14A*, *ACY1*); Cluster C5 was associated with the immune response (*TGFBR1*, *TGFB2*); and Cluster C6 highlighted inflammation (*CXCL1*, *IL1B*). Notably, *IL1B* and *TGFB2* appeared in multiple clusters, linking the processes of growth, metabolism, immunity, and inflammation. (B) GO enrichment analysis revealed key enriched terms (*P* ≥ 0.05, *q* < 0.2): “cyclin-dependent protein serine/threonine kinase activity” in Cluster C2, “MHC protein complex binding” in Cluster C4, and “acetyltransferase activity” in Cluster C5. Cluster C6 was particularly enriched for “cytokine activity,” underscoring specialized roles in the immune response, cell cycle regulation, and epigenetic modifications. (C) Kyoto Encyclopedia of Genes and Genomes (KEGG) pathway enrichment highlighted diverse biological processes across clusters. Cluster C2 was enriched for cancer-related pathways such as “small cell lung cancer,” whereas Cluster C4 was linked to neurodegenerative disorders such as Parkinson’s disease and AD. Cluster C5 emphasized immune responses to viral infections (e.g., Epstein‒Barr virus), whereas Cluster C6 involved TNF and IL-17 signaling. Cluster H included a broader range of pathways, including the TNF signaling pathway. (D–G) Differential expression analysis of highly recurring genes (occurring ≥ 8 times across categories) across four independent datasets: AD Neuroimaging Initiative (ADNI; D), Religious Orders Study and Rush Memory and Aging Project (ROSMAP; E), Mount Sinai Brain Bank (MSBB; F), and Mayo Clinic AD Genetics Studies (Mayo; G). The MSBB dataset presented the most pronounced differential expression signals, with genes such as *TGFB2*, *DUSP1*, and *CXCL1* showing consistent and statistically significant changes across cohorts. Notably, *TGFB2*, *DUSP1*, and *CTSS* displayed particularly strong differential expression in the MSBB dataset, which aligns closely with findings from the ADNI and Mayo and reinforces their potential roles in AD-related neurodegenerative processes. AD: Alzheimer’s disease.

To further characterize the identified gene sets by their biological meaning, we considered their functional annotations using Gene Ontology (GO) and Kyoto Encyclopedia of Genes and Genomes (KEGG) enrichment analyses. We systematically analyzed the significance across several categories within MSigDB for the gene sets. We first identified and analyzed the top 10 gene frequencies in each category (**Figure 4A**). Cluster C2 described the cell cycle with *CDK2* and *CDK4*, whereas C3 emphasized lipid metabolism with *ABHD14A-ACY1*. Cluster C5 summarized immune responses with *TGFBR1* and *TGFB2*, in contrast to Cluster C6, which highlighted inflammation with *CXCL1* and *IL1B*. Cluster C8 integrated hormonal and immune pathways involving *IL6* genes. The overlap of important genes across many clusters, especially of *IL1B* and *TGFB2*, indicates their importance across all biological significance; they mapped to overall health along with growth, metabolism, immunity, and inflammation (**Figure 4B** and **C**).

We devised gene selection criteria in the functional analyses, which included the following. First, from both clusters of categories, we determined the genes with above-average importance scores. We then counted how many times each gene appeared, and only kept those that were proteins of a gene that was reflected in at least two of the categories for downstream analysis We performed a GO enrichment analysis using *P* ≥ 0.05 and *q* < 0.2. The number of enriched entries varied across the clusters (C2: 24, C4: 33, C5: 174, C6: 18, C7: 1, and H: 34) (**Figure 4B** and **Additional Table 6**). When we compared terms related to each cluster, C2 was related to processes that regulate the cell cycle, included terms such as “cyclin-dependent protein serine/threonine kinase activity” (*P* = 1.15 × 10^–4^), and was significantly related to cell cycle regulation. C4 was very enriched for immune response-related functions such as “MHC protein complex binding” (*P* = 8.26 × 10^–13^), which further indicates its relevance in antigen presentation. C5 was prominently marked by “acetyltransferase activity” (*P* = 8.37 × 10^–70^), thereby underscoring its potential involvement in epigenetic regulation. C6 was defined by “cytokine activity” (*P* = 9.92 × 10^–4^), which indicates that it participates in cytokine-mediated immune signaling. C7 and Cluster H also demonstrated relevant functional associations, with C7 linked to “protein phosphatase binding” (*P* = 1.18 × 10^–4^) and H related to various receptor activities. Comparing the clusters, C2 and C7 were only related to a handful of relevant terms, whereas C4 and C5 had a wider range of ministry functions; C4 ministered more functions related to the immune response, whereas C5 had acetyltransferase activity. C6, with its cytokine-related functions, showed intermediate breadth of associations compared with other clusters, highlighting its involvement in both immune signaling and cell communication pathways. Finally, H had a diverse collection of functions that related to receptor activities and binding functions.

KEGG pathway enrichment analysis of the various clusters displayed an abundant assortment of biological processes (**Figure 4C** and **Additional Table 6**). In C2, pathways enriched in cancer were strongly represented, such as “small cell lung cancer” (*P* = 3.91 × 10^–7^) and “viral carcinogenesis” (*P* = 0.0004), thus indicating the importance of genetic predispositions relevant to cancer development and viral infections. C4 exhibited a strong association with neurodegenerative disorders, as illustrated by the clustering of genes in pathways such as Parkinson’s disease (*P* = 1.48 × 10^–14^) and AD (*P* = 8.91 × 10^–12^), with key genes and pathways that may inform us of these conditions. C5 was prominently enriched in immune responses to viral infections, such as Epstein–Barr virus (*P* = 3.55 × 10^–14^) and hepatitis B (*P* = 0.00667), indicating the heightened activation of immune-related genes in these pathways. C6, with its emphasis on cytokine signaling and interactions between immune cells, was enriched in the TNF signaling pathway (*P* = 9.77 × 10^–14^) and interleukin (IL)-17 signaling pathway (*P* = 0.00151), suggesting a predominant role for these clusters as modulators of immune responses. Although it was less enriched, C7 was linked to immune system dynamics and the viral response, with pathways such as T-helper (Th)1 and Th2 cell differentiation (*P* = 0.0011) and hepatitis C (*P* = 0.0029), thus highlighting their importance in immune cell function and viral pathogenesis. Lastly, in cluster H, a diverse group of pathways was enriched, such as the TNF signaling pathway (*P* = 7.98 × 10^–5^) and hepatitis B (*P* = 0.00667), thereby reflecting its multifaceted role in immune signaling and disease-associated cellular processes. These functional profiles elucidate the different biological roles of the required gene clusters and extend the relationship to neurodegeneration, immune development, and metabolic regulation in AD (**Additional Figure 4**).

### Cross-dataset assessment of differential gene expression profiles

To characterize the consistency of expression and their biological relevance to the key candidate genes across cohorts, we performed a cross-dataset differential gene expression analysis focusing on genes with high recurrence in functional categories. In our analysis, we focused on the highly recurring genes (eight or more times in the categories) and explored their associated differential expression. Our cross-dataset analysis that incorporated ADNI (**Figure 4D**), ROSMAP (**Figure 4E**), MSBB (**Figure 4F**), and Mayo (**Figure 4G**) exhibited distinct expression profiles for AD-associated genes. The MSBB dataset had the greatest degree of differential gene expression, with most genes showing strong significance. By contrast, the ADNI dataset exhibited a more subtle pattern of differential expression. We identified consistent findings for three core genes—*TGFB2*, *DUSP1*, and *CXCL1*—with respect to their differential expression across datasets. Within the ADNI dataset, significant expression differences were observed for *DUSP1* (*P* = 0.0478), CXCL1 (*P* = 0.0314), and *CTSS* (*P* = 0.0384). The ROSMAP cohort further supported the role of *DUSP1* (*P* = 0.00117). The MSBB dataset was distinctly greater for differential gene expression, particularly in terms of *TGFB2* (*P* = 1.56 × 10^–9^), *DUSP1* (*P* = 3.89 × 10^–12^), and *CTSS* (*P* = 1.96 × 10^–17^), which was supportive of the findings from ADNI. The Mayo dataset had very small *P*-values for *DUSP1* (*P* = 3.76 × 10^–16^), *TGFB2* (*P* = 9.38 × 10^–7^), and *COL1A1* (*P* = 6.63 × 10^–9^), which provides strong evidence for their association with neurodegeneration. The cross-cohort expression patterns indicate the broad strength and repeatability of important AD-related genes such as *TGFB2*, *DUSP1*, and *CXCL1*, and further support their potential as reproducible molecular indicators of AD pathology.

### Assessment of Alzheimer’s disease pathology–related markers

To examine the associations between important gene sets and AD pathological mechanisms, we used PRS models with the genotype data from ADNI and ROSMAP. To capture the top 50% of prominent polygenic scores, we used three algorithmic machine learning methods and performed rigorous selection of the best gene sets, derived from three CSF biomarkers from the ADNI dataset and five pathological phenotypes from the ROSMAP dataset, which yielded 15 and five important gene sets, respectively. The subsequent gene sets were believed to play important roles in the presentation of various AD pathological phenotypes. Correlation matrices were developed to elucidate the interactions between genetic factors and phenotypic traits, as well as their connections to AD and relevant demographic markers.

**Figure 5:**
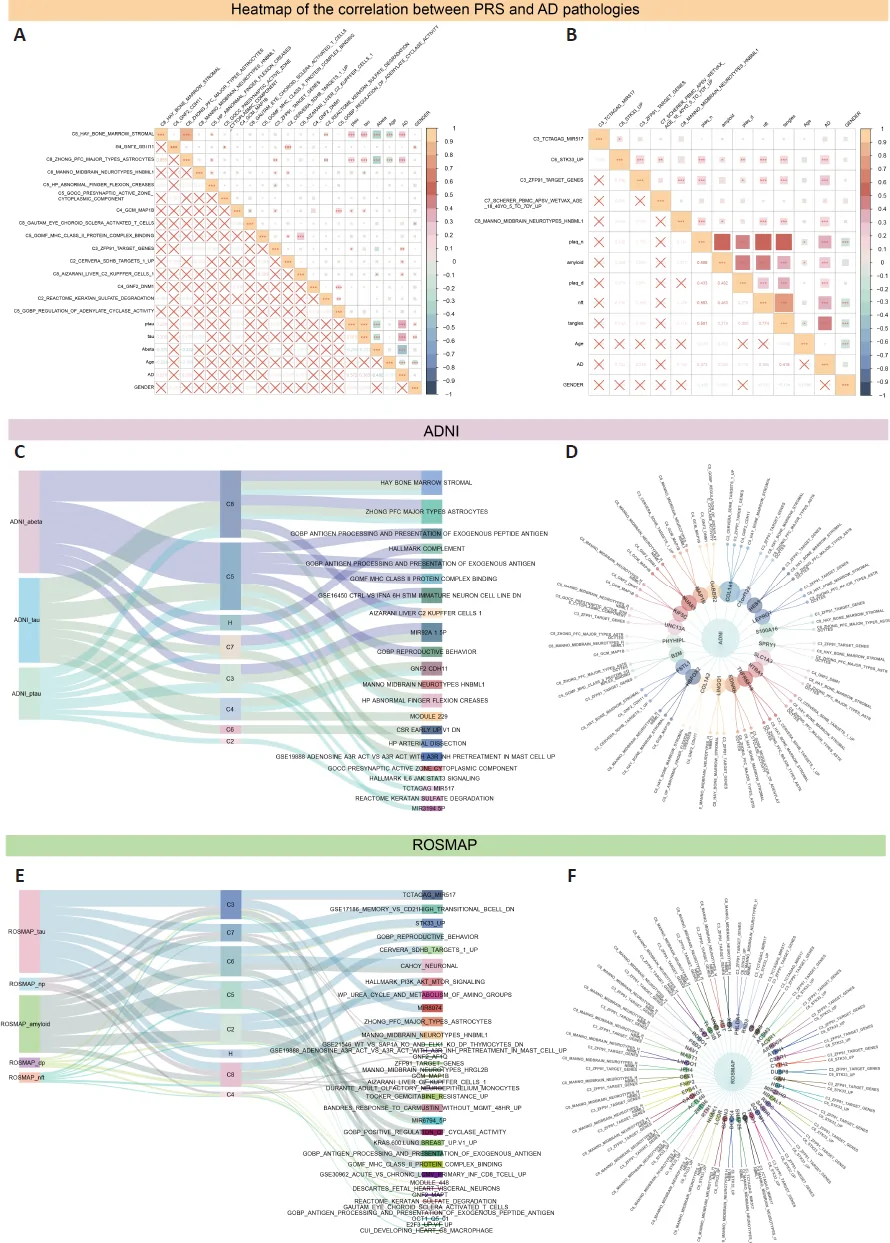
Integration of polygenic risk scores with AD pathological features across the ADNI and ROSMAP cohorts. This study investigated gene set associations with AD pathogenesis through polygenic risk score (PRS) analysis in the ADNI and ROSMAP cohorts. Machine learning algorithms were employed to identify the top 50% important polygenic scores, yielding 15 pivotal gene sets from ADNI and five from ROSMAP. (A) Heatmap of polygenic risk score (PRS) correlations with Alzheimer’s disease (AD) phenotypes in the ADNI cohort. A significant positive correlation was detected between *C8_Hay Bone Marrow Stromal* and *C8_Zhong PFC* *major type astrocytes* (*P* < 0.001, *Cor* = 0.855), with both negatively associated with CSF Aβ levels (*P* < 0.001, *Cor* = –0.375 and –0.332, respectively). Additional gene sets, including *C4_GCM Map1B*, *C3_ZFP91 Target Genes*, *C2_Cervera SDHB Targets 1 Up*, and *C8_Aizarani Liver C2 Kupffer Cells 1*, were significantly correlated with AD phenotypes. (B) Heatmap of PRS correlations with AD phenotypes in the ROSMAP cohort. *C6_STK33 Up*, *C3_ZFP91 Target Genes*, and *C8_Manno Midbrain Neurotypes HNBML1* exhibited significant associations with multiple AD pathologies, including neuritic plaque (NP), diffuse plaque (DP), neurofibrillary tangles (NFT), tau, ptau, and overall AD status. Notably, *C6_STK33* was positively correlated with *C3_ZFP91* target genes (*P* < 0.001, *Cor* = 0.166). Cross-cohort analysis identified *C3_ZFP91 target genes* as a shared AD-related gene set (*P* < 0.01, *Cor*_ADNI_ = 0.095; *P* < 0.001, *Cor*_ROS_ = 0.218). (C) Sankey diagram of the top-ranking gene sets and their associated immune-related pathways in the ADNI dataset. Gene sets prioritized from CSF Aβ, tau, and ptau phenotypes were largely enriched in immune processes, including antigen presentation, IL-6 signaling, and the complement cascade. (D) Circular plot of frequently occurring genes within AD-associated gene sets in the ADNI dataset. A total of 21 genes met the recurrence threshold (≥ 3 appearances). Notably, *COL1A1* exhibited extensive overlap across several gene sets, including *C2_Cervera SDHB Targets 1 Up*, *C3_ZFP91 Target Genes*, *C4_GNF2 CDH11*, and *C8_Hay Bone Marrow Stromal*. Other key genes included *C1orf122*, *HES1*, *LEPROT*, *S100A16*, and *B2M*, implicating them in immune and neural pathways. (E) Sankey diagram of gene set–pathway associations in the ROSMAP cohort. Enriched pathways included metabolic signaling (e.g., *PI3K-AKT-MTOR*) and immune regulation. Distinct gene sets, such as *TCTAGAG MIR517* and *C6_STK33 Up*, which are absent in *ADNI*, revealed unique ROSMAP-specific signatures. (F) Circular plot of frequently occurring genes in the ROSMAP gene set associated with AD pathology. A total of 43 genes met the inclusion threshold (≥ 2 appearances), including *PHLDA1*, *RND3*, *CS*, *FBRS*, *ACTL6B*, *CACNG7*, and *EPB41*, many of which are involved in microRNA regulation and mitochondrial function. *COL1A1* and *PHLDA1* were notable for their widespread presence and classification as mitochondrial epistatic genes. AD: Alzheimer’s disease; ADNI: AD Neuroimaging Initiative; Mayo: Mayo Clinic AD Genetics Studies; MSBB: Mount Sinai Brain Bank; ROSMAP: Religious Orders Study and Rush Memory and Aging Project.

There was a high correlation between *C8_Hay Bone Marrow Stromal* and *C8_Zhong PFC Major Types Astrocytes* in the ADNI dataset (*P* < 0.001, Pearson correlation coefficient (*Cor*) = 0.855). Both gene sets were negatively correlated with CSF Aβ levels (*P* < 0.001, *Cor* = −0.375 and −0.332). Furthermore, *C4_GCM Map1B* was significantly correlated with CSF p-tau and tau proteins (*P* < 0.05, Cor = 0.074 and 0.071, respectively). Additionally, *C3_ZFP91 Target Genes*, *C2_Cervera SDHB Targets 1 Up*, and *C8_Aizarani Liver C2 Kupffer Cells 1* were all significantly associated with AD (*P* < 0.01, *P* < 0.05, *P* < 0.05; *Cor* = 0.095, 0.075, and 0.072, respectively). There were also significant associations between *C3_ZFP91 Target Genes* and three distinct gene sets: *C8_Zhong PFC Major Types Astrocytes*, *C8_Manno Midbrain Neurotypes HNBML1*, and *C5_HP Abnormal Finger Flexion Creases* (*P* < 0.01, *P* < 0.05, *P* < 0.05; Cor = 0.093, 0.063, and −0.069, respectively) (**Figure 5A**).

In the ROSMAP dataset, *C6_STK33 Up* was significantly associated with five pathological phenotypes of brain tissue and AD status. In addition, *C3_ZFP91* Target Genes was significantly associated with NP, DP, NFT, p-tau, and AD. Similarly, *C8_Manno Midbrain Neurotypes HNBML1* was significantly associated with NP, Aβ, NFT, p-tau, and AD. Notably, *C6_STK33 Up* demonstrated a positive correlation with *C3_ZFP91 Target Genes* (*P* < 0.001, *Cor* = 0.166). Cross-dataset comparison revealed *C3_ZFP91 Target Genes* as a common significant correlate of AD in both datasets (*P*_ADNI_ < 0.01, *Cor*_ADNI_ = 0.095; *P*_ROSMAP_ < 0.001, *Cor*_ROSMAP_ = 0.218) (**Figure 5B**). These findings demonstrate strong and consistent associations between specific gene sets and AD pathologies, thereby offering mechanistic insights into disease progression.

### Comparative analysis of Alzheimer’s disease pathological phenotypes

The analysis of gene set associations across several datasets and pathological measures helps us to understand how genetic variations correlate to different aspects of AD pathology. For each pathological indicator, we performed a pairwise comparative analysis and identified our top 10 gene sets based on overall significance. For the ADNI dataset, CSF Aβ was the most important indicator of pathology, and the C5 gene set had the highest associations between the top 10 indicators. Conversely, CSF tau had the most overall importance and the most associations for phenotype indicators of the C6 gene set. Overall, we identified a consistent emphasis on immune-related pathways in the ADNI dataset, including pathways related to the complement system as well as IL-6 and Janus kinase–signal transducer and activator of transcription 3 signaling. Neuronal inflammation may thus play a role in the development of AD pathology, especially because the immune response may be influential for disease course and pathophysiology. Furthermore, gene sets such as *C8_Hay Bone Marrow Stromal* and *C5_GOBP Antigen Processin*g showed importance for antigen presentation and immune interactions (**Figure 5C**).

By contrast, the ROSMAP dataset displayed a wider spectrum of pathways (**Figure 5E**), including metabolic signaling cascades such as the PI3K–AKT–mTOR pathway—an axis that is commonly implicated in metabolic disturbances associated with neurodegenerative diseases. Additionally, enrichment analysis revealed gene sets with unique markers—specifically, *TCTAGAG MIR517* and *STK33 Up*, which were not found in the ADNI dataset—thereby presenting more layers of complexity in relation to the biological processes presented in these data. The common gene functions in both datasets, especially in terms of antigen processing and presentation, illustrate the potential role of immune mechanisms in AD pathology.

A quantitative approach to the shared gene sets revealed commonality in many genes by frequency (frequency threshold: ADNI ≥ 3, ROSMAP ≥ 2, **Additional Table 7**). This approach revealed 21 genes in the ADNI dataset and 43 genes in the ROSMAP dataset, yielding three common genes: *LINGO1*, *NUAK1*, and *UNC13A*. Notably, these genes had been previously noted as mitochondrial epistatic genes (Xu et al., 2022, 2024). In the ADNI dataset (**Figure 5D**), *COL1A1* had numerous associations with other gene sets including *C2_Cervera SDHB Targets 1 Up*, *C3_ZFP91 Target Genes*, *C4_GNF2 CDH11*, and *C8_Hay Bone Marrow Stromal*. Other genes (e.g., *C1orf122*, *HES1*, *LEPROT*, and *S100A16*) were associated with *C3-ZFP91 Target Genes*, *C8_Hay Bone Marrow Stromal*, and *C8_Zhong PFC Major Types Astrocytes*, suggesting a possible role in neural processes and, potentially, in immune-related functions. B2M was associated with *C3_ZFP91 Target Genes*, *C5_GOMF MHC Class II Protein Complex Binding*, and *C8_Zhong PFC Major Types Astrocytes*, highlighting its role in the immune response and neuronal function.

In the ROSMAP dataset (**Figure 5F**), PHLDA1 was associated with *C3_ZFP91 Target Genes*, *C6_STK33 Up*, and *C8_Manno Midbrain Neurotypes HNBML1*, indicating its potential involvement in multiple biological mechanisms. Other genes (e.g., *RND3*, *CS*, and *FBRS*) were associated with *C3_TCTAGAG MIR517* and *C6_STK33 Up*, implying possible interactions with microRNA (miRNA) regulation and various cellular responses. Many genes, including *ACTL6B*, *CACNG7*, and *EPB41*, were connected to *C3_ZFP91 Target Genes* and *C8_Manno Midbrain Neurotypes HNBML1*, thus confirming the repeat importance of these pathways. Notably, *COL1A1* and *PHLDA1*, which appeared most frequently across multiple gene sets, were classified as mitochondrial epistatic genes. In summary, the comparative analyses imply a convergence of immune, metabolic, and mitochondrial mechanisms in AD pathology, and suggest that common gene signatures across pathological phenotypes and datasets may be informative.

### Analysis of polygenic genetic risk associations between multiple diseases and Alzheimer’s disease

**Figure 6:**
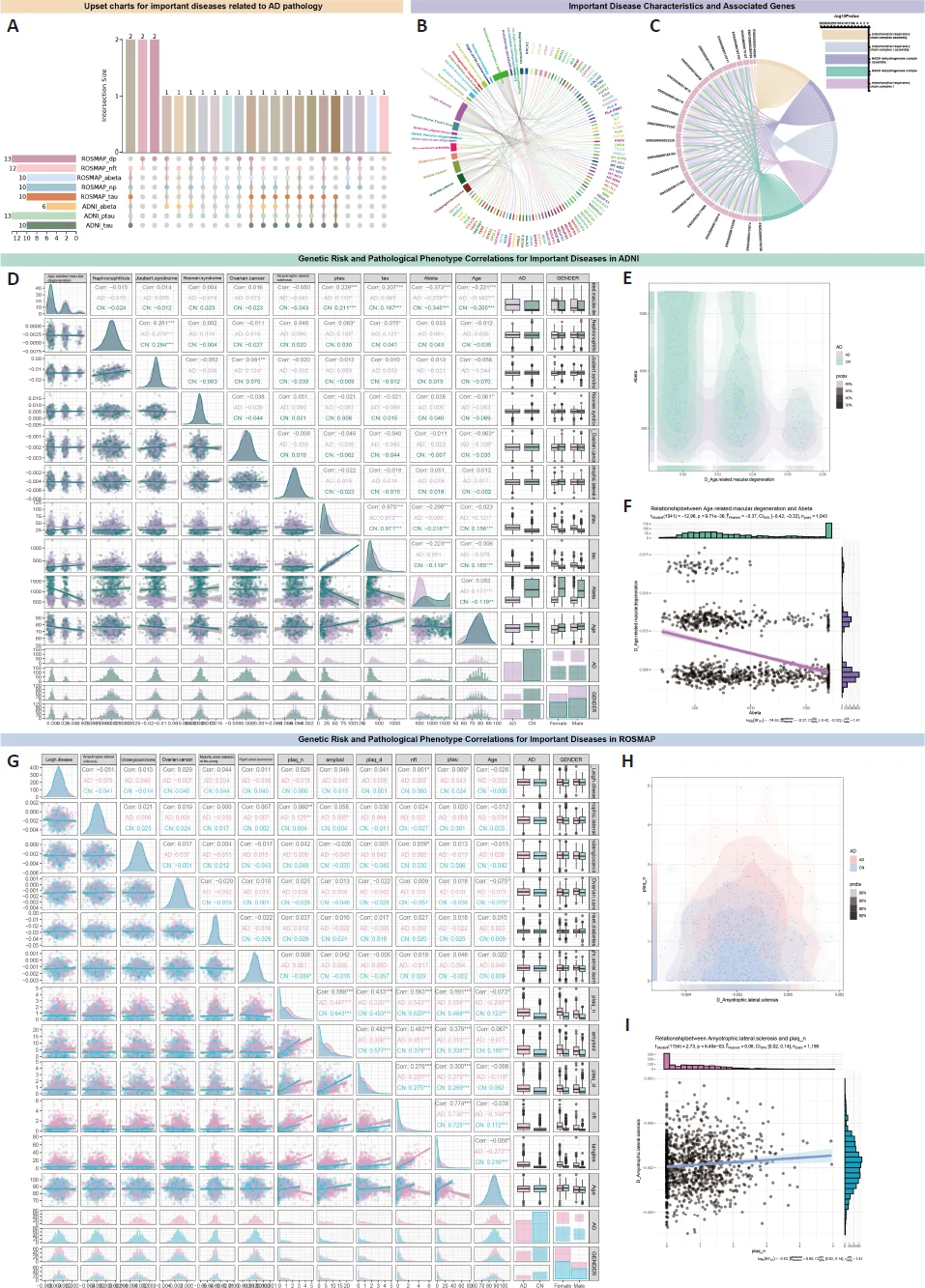
Associations between various diseases and AD pathologies across the ADNI and ROSMAP cohorts. The associations between diseases and different AD pathologies were explored. (A) A multigene risk scoring model was developed using the gene with the strongest association for each disease. Diseases that surpassed the average importance for each phenotype were identified, with ADNI_ptau and ROSMAP_DP displaying the most diverse disease characteristics (13 each). Age-related macular degeneration was the most significant and was correlated with seven other pathological phenotypes. (B) Key genes linked to these diseases, including *MT-ATP6*, *MT-ND1*, and *MT-ND2*, were analyzed, with Leigh disease showing associations with multiple mitochondrial genes. Additionally, *BRCA1*, *BRCA2*, *CTLA4*, *PTEN*, and *TP53* were highlighted as critical genes, particularly those related to breast cancer and autoimmune disorders. (C) GO analysis revealed associations with mitochondrial respiratory chain complex assembly (GO: 0033108), NADH dehydrogenase complex assembly (GO: 0010257), and immune processes such as T-cell differentiation (GO: 0042102). (D) Correlation matrix in the ADNI dataset showing associations between disease risk and AD phenotypes. AMD was negatively correlated with CSF Aβ (*P* < 0.001, *Cor* = –0.373) and positively correlated with CSF tau and ptau (*P* < 0.05). Nephronopathy was positively correlated with both CSF pathology and Joubert syndrome risk (*P* < 0.001, *Cor* = 0.281). (E) Density plot illustrating higher AMD polygenic risk scores in AD patients than in non-AD individuals in the ADNI. (F) Scatter plot showing a significant negative correlation between AMD risk and CSF Aβ levels according to the ADNI (*P* < 0.001). (G) Correlation matrix in ROSMAP indicating positive associations of ALS with NP (*P* < 0.01) and of Leigh disease with NFT and p-tau (*P* < 0.05). (H) Density plot displaying elevated Leigh disease risk in AD individuals in ROSMAP. (I) Scatter plot showing positive correlations between Leigh disease and NFT (*P* < 0.05) and between cholangiocarcinoma risk and NFT levels (*P* < 0.05). AD: Alzheimer’s disease; ADNI: AD Neuroimaging Initiative; AMD: age-related macular degeneration; Aβ: amyloid-β; CSF: cerebrospinal fluid; Mayo: Mayo Clinic AD Genetics Studies; MSBB: Mount Sinai Brain Bank; NFT: neurofibrillary tangle; p-tau: phosphorylated tau; ROSMAP: Religious Orders Study and Rush Memory and Aging Project.

To investigate the associations between various diseases and AD pathologies, we constructed PRS models, incorporating the most strongly associated gene for each disease. Through comprehensive modeling and feature importance analyses, we identified diseases that exhibited above-average associations with each AD-related phenotype. Of these, ADNI_ptau and ROSMAP_DP exhibited the highest number of disease associations (13 each) (**Figure 6A**). Age-related macular degeneration emerged as the top-ranking disease, with potentially significant associations with seven additional pathological phenotypes beyond ROSMAP_DP. Nephronophthisis was implicated in six phenotypes, excluding ROSMAP_DP and ROSMAP_NFT (**Additional Table 8**). For the diseases that were identified as important features in at least two pathological phenotypes, we analyzed the genes with the strongest associations (genes with a maximum score of five in the DISEASE database) (**Figure 6B**). Leigh disease was associated with the greatest number of genes; these were predominantly mitochondrial genes (e.g. *MT-ATP6*, *MT-ND1*, and *MT-ND2*), as well as nuclear-encoded genes involved in mitochondrial complex I assembly (e.g., *NDUFA1*, *NDUFA2*, and *NDUFA9*). Among the individual genes, *BRCA1*, *BRCA2*, *CTLA4*, *PTEN*, and *TP53* exhibited the highest disease association frequency (all linked with breast cancer), and *TP53* was specifically identified as a mitochondrially localized gene. *CTLA4* was linked to alopecia areata, breast cancer, and rheumatoid arthritis, thus indicating its involvement not only in oncological contexts but also in autoimmune disorders. GO analysis also revealed significant enrichment in mitochondrial respiratory chain complex assembly (GO: 0033108, false discovery rate [FDR] = 2.10 × 10^–24^) and reduced nicotinamide adenine dinucleotide dehydrogenase complex assembly (GO: 0010257, FDR = 3.07 × 10^–23^), alongside immune processes such as T cell differentiation (e.g. GO: 0042102, FDR = 0.0009; **Figure 6C** and **Additional Table 9**).

Further exploration of the top 50% of diseases with the highest importance in each phenotypic association in the ADNI and ROSMAP databases revealed that the risk of age-related macular degeneration in ADNI differed significantly between AD and non-AD individuals. Individuals at high risk for age-related macular degeneration showed an elevated probability of developing AD (**Figure 6D–F**). This condition was also significantly associated with all three CSF pathology markers, demonstrating the strongest negative correlation with CSF_Aβ (*P* < 0.001; *Cor*_CSF_Aβ_ = –0.373, *Cor*_CSF_tau_ = 0.207, *Cor*_CSF_ptau_ = 0.229). Nephronophthisis showed a significant positive correlation with the risk of Joubert syndrome (*P* < 0.001, *Cor* = 0.281) and was positively correlated with CSF_ptau and CSF_tau in the AD group (*P* < 0.05, *Cor* = 0.105/0.121). In the ROSMAP dataset, amyotrophic lateral sclerosis exhibited the strongest association with NP in AD patients (*P* < 0.01, *Cor* = 0.125; **Figure 6G–I**). Leigh disease exhibited significant positive correlations with NFT and p-tau levels (*P* < 0.05, *Cor* = 0.061/0.069), and cholangiocarcinoma was also positively correlated with NFT levels (*P* < 0.05, *Cor* = 0.059). A nomogram derived from the ADNI analysis indicated that conditions such as age-related macular degeneration and mitochondrial complex I deficiency significantly affect the age of onset of AD. Furthermore, the ROSMAP results underscored the substantial effects of diseases such as rheumatoid arthritis and Joubert syndrome (*P* < 0.001; **Additional Figure 5A** and **B**). Together, these results suggest potential pleiotropic effects that link systemic diseases to AD pathology (particularly, involving immune and mitochondrial-related genes).

### Epigenetic profiling reveals Alzheimer’s disease–associated enriched methylation patterns

**Figure 7:**
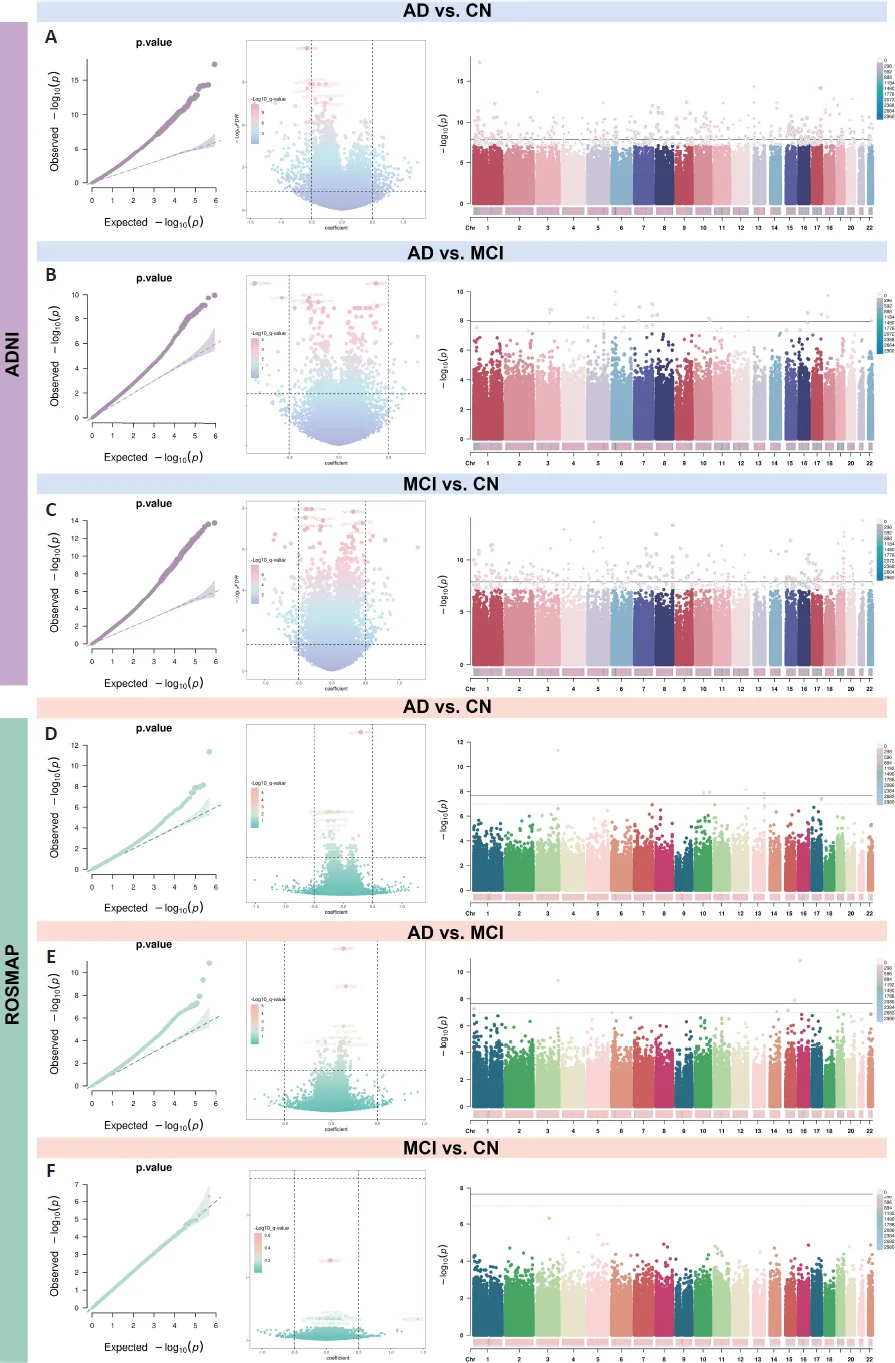
Genome-wide methylation profile analysis across AD progression stages. EWAS analysis revealed significant methylation changes across different cognitive stages in the ADNI and ROSMAP cohorts. Specifically, comparisons were made between AD patients and cognitively healthy individuals. (A) Volcano plot of EWAS results in the ADNI dataset comparing AD patients to CN individuals. Significant differentially methylated CpG sites were identified, with more widespread changes observed compared with the findings with ROSMAP. (B) EWAS volcano plot for AD patients *vs.* MCI patients in the ADNI dataset. Several CpG sites displayed significant differences in methylation, highlighting potential epigenetic shifts during disease progression. (C) Comparison of MCI *vs.* CN in the ADNI showing relatively fewer differentially methylated CpG sites, suggesting earlier-stage epigenetic alterations. (D) Volcano plot of the ROSMAP EWAS results for AD patients *vs.* CNs. Although fewer significant CpG sites were observed than in the ADNI, key epigenetic signals were still detected. (E) EWAS results for AD patients *vs.* MCI patients in ROSMAP. Limited significant CpG differences were noted, indicating subtler epigenetic changes at this stage in this cohort. (F) MCI *vs.* CN comparison in ROSMAP. The number of differentially methylated sites was similar to that in the ADNI, providing insight into transitional epigenetic regulation. Notably, the ADNI cohort demonstrated a greater number of significant associations than did the ROSMAP cohort, with the following results: AD *vs.* CN (ADNI = 144, ROSMAP = 28), AD *vs*. MCI (ADNI = 111, ROSMAP = 71), and MCI *vs*. CN (ADNI = 18, ROSMAP = 20). AD: Alzheimer’s disease; ADNI: AD Neuroimaging Initiative; CN: cognitively normal; EWAS: epigenome-wide association study; MCI: mild cognitive impairment; ROSMAP: Religious Orders Study and Rush Memory and Aging Project.

To explore the epigenetic mechanisms underlying AD, we analyzed DNA methylation patterns using EWAS across disease stages and brain regions. EWAS identified significant methylation sites in the ADNI and ROSMAP datasets when comparing AD with CN individuals (**Figure 7A** and **D**), AD with MCI (**Figure 7B** and **E**), and MCI with CN (**Figure 7C** and **F**). Notably, ADNI demonstrated more pronounced methylation differences compared with ROSMAP (AD *vs*. CN: 144 *vs*. 71; AD *vs*. MCI: 111 *vs.* 28; MCI *vs.* CN: 18 *vs.* 20) (**Additional Table 10**). We selected CpG sites that corresponded to genes that appeared at least three times and showed significant associations in one cohort (ADNI = 111, ROSMAP = 33) for further analysis (**Additional Table 11**).

**Figure 8:**
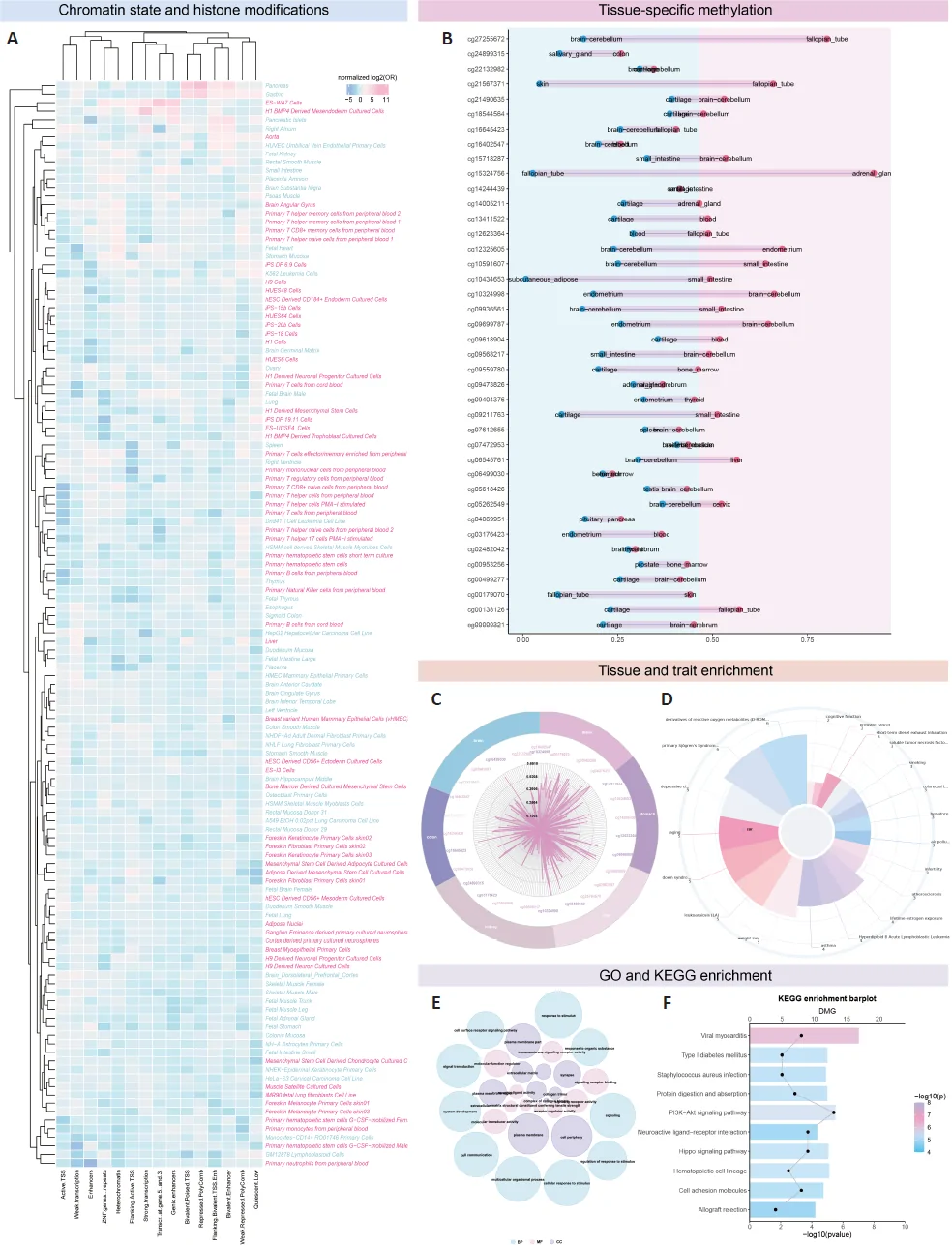
Comprehensive methylation and functional enrichment analysis in the ADNI cohort. Epigenetic analysis of CpG loci was conducted to compare methylation patterns between AD and MCI patients *versus* CN controls,, with a focus on chromatin state, tissue-specific methylation, and enrichment analyses. (A) Heatmap illustrating the chromatin state and histone modification enrichment for significant CpGs in various cell types, with a particular focus on transcriptional activity at the 5’ and 3’ ends of genes. Significant enrichment was detected for genetic enhancers, strong transcription sites, and transcription initiation sites in both ES-WA7 and H1 BMP4-derived mesendoderm cultured cells, as well as bivalent regulatory regions in pancreatic and gastric tissues. (B) Tissue-specific methylation patterns demonstrated the hypermethylation or hypomethylation status across different tissues for specific CpG sites. Notably, increased methylation was detected for CpGs such as *cg21567371 (COL7A1)*, *cg15718287 (DCLK1)*, and *cg07472953 (CSF3)* in brain-related regions. (C) The radar plot shows tissue-specific correlations, with the strongest correlations observed between cg04069951 and brain-related tissue (*Cor* = 0.614), whereas cg09211763 exhibited the strongest negative correlation with the brain (*Cor* = –0.645). (D) Trait enrichment analysis revealed significant correlations between identified CpG sites and traits such as primary Sjögren’s syndrome, reactive oxygen species metabolites (D-ROMs) in plasma, aging, depressive disorders, and cognitive function. This highlights the relevance of these methylation patterns to neurodegeneration and aging-related comorbidities. (E) Gene Ontology (GO) analysis of loci enriched in the nervous system. Several loci, including *cg02482042 (ATP2B2)*, *cg07472953 (CSF3)*, and *cg22132982 (WNT7A)*, are highly expressed in the nervous system. These loci were significantly associated with pathways such as viral myocarditis, PI3K-Akt signaling, and hematopoietic cell lineage, suggesting broad roles in immune regulation and signal transduction. (F) KEGG pathway enrichment for differentially methylated genes (DMGs). KEGG enrichment analysis revealed significant involvement of DMGs in pathways related to viral infections, immune regulation, and cellular processes, including cell adhesion and hematopoietic cell differentiation. Pathways such as the PI3K-Akt signaling pathway are particularly enriched in AD-associated genes. AD: Alzheimer’s disease; ADNI: AD Neuroimaging Initiative; CN: cognitively normal; MCI: mild cognitive impairment.

In the ADNI dataset, significant enrichment for transcriptional activity was found at the 5′ and 3′ ends of genes in the ES-WA7 cell line, with trends for genic enhancers in H1 BMP4-derived mesendoderm cells. The notable enrichment of bivalent/poised transcriptional start site (TSS) and Polycomb statuses was observed in pancreatic and gastric tissue (**Figure 8A** and **Additional Table 12**). Histone modifications (particularly, H3K27me3) were enriched in pancreatic and hematopoietic stem cells (odds ratio = 4.42 and 4.14; **Additional Figure 6A** and **Additional Table 13**). Tissue-specific methylation patterns were evident for cg21567371 (*COL7A1*), cg15718287 (*DCLK1*), and cg07472953 (*CSF3*) in the brain, with cg10324998 (TGFBR3) and cg09699787 (*PXDN*) showing high expression specificity (hypergeometric enrichment *P*-value (*P*_hyper_) = 0.663, 0.647) (**Figure 8B** and **Additional Table 14**). The strongest correlations were noted between cg04069951 and brain tissue (*Cor* = 0.614) and cg09211763 (*Cor* = −0.645) (**Figure 8C** and **Additional Table 15**). Significant associations were also identified with aging, depressive disorder, and cognitive function (*P* = 1.51 × 10^–5^, 1.58 × 10^–5^, and 0.00381, respectively). These loci demonstrated significant correlations with primary Sjögren’s syndrome and derivatives of reactive oxygen metabolites in plasma (*P* = 2.30 × 10^–6^ and 2.90 × 10^–6^ respectively) (**Figure 8D**). Key genes, including cg02482042 (*ATP2B2*), cg07472953 (*CSF3*), and cg22132982 (*WNT7A*), were highly expressed in the nervous system and were significantly associated with pathways such as viral myocarditis, phosphoinositide 3-kinase (PI3K)–protein kinase B (Akt) signaling, and hematopoietic cell lineage (FDR = 3.34 × 10^–5^, 0.000535, and 0.000591, respectively; **Figure 8E**, **8F**, **Additional Figure 6B–D**, and **Additional Table 16**).

**Figure 9:**
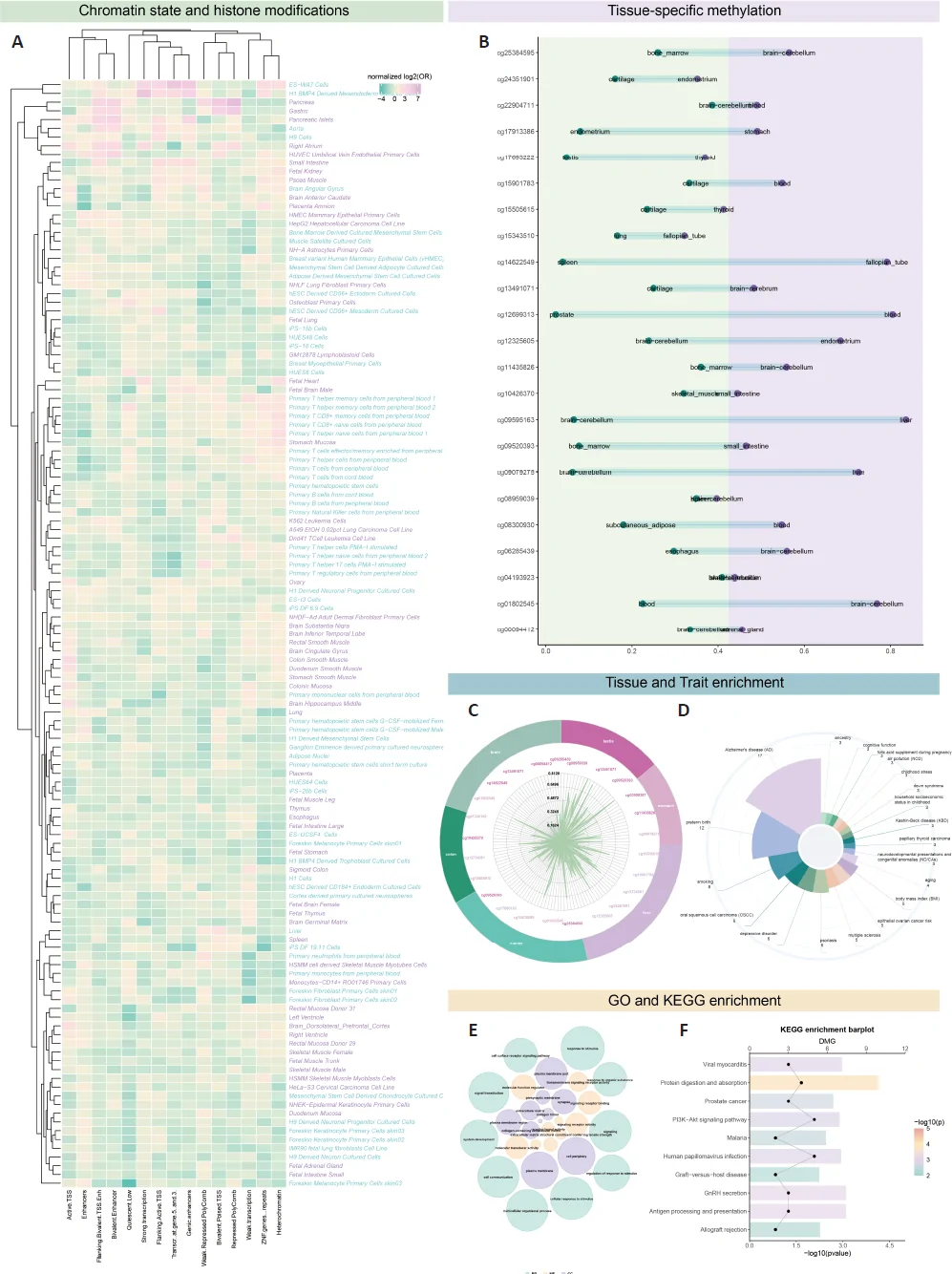
Methylation patterns and phenotypic correlations of significant CpG sites in the ROSMAP cohort. (A) Significant enrichment of methylation sites was identified within the transcription start and end states in ES-WA7 cells, which is consistent with findings from the ADNI study. Enrichment was also observed in the bivalent/poised TSS, flanking bivalent TSS/Enh, bivalent enhancer, and repressed PolyComb states in pancreatic and gastric tissues. In T-cell-related cell lines, including primary T helper memory cells from peripheral blood and primary T CD8^+^ naive cells, notable enrichment of ZNF genes and repeats, as well as heterochromatin, was detected. (B) Higher methylation levels were observed for loci such as *cg06285439* (*NRP2*), *cg12699313* (*GNS*), and *cg08959039* (*COL4A2*) in the brain-cerebellum, with *cg01802545* (*LGMN*) exhibiting the strongest expression specificity in this region (*P*_hyper_ = 0.769). Methylated sites, including cg04193923 (*CSF3*), cg01802545 (*LGMN*), and cg13491071 (*ROR2*), are predominantly expressed in the nervous system. (C) Within the brain, the CpG site cg17913386 showed the strongest correlation with tissue-specific expression (Cor = 0.268). (D) Phenotypic analysis revealed significant associations with AD (*P* = 1.66 × 10^–17^), confirming the relevance of the identified loci. Notable correlations were also found with preterm birth, smoking, psoriasis, and depressive disorders (*P* = 1.22 × 10^–12^, 1.07 × 10^–8^, 6.00 × 10^–7^, and 1.43 × 10^–6^, respectively). The loci screened for aging and cognitive function in the ADNI were similarly enriched in ROSMAP, with additional links to neurodevelopmental conditions and congenital anomalies (ND/CAs) (*P* = 1.78 × 10^–5^, 0.000359, 0.00168). (E) GO enrichment analysis of methylated sites. GO analysis revealed that the 33 differentially methylated loci were predominantly involved in signal transduction and cell communication pathways (FDR = 0.00157). Immune processes, including protein digestion and absorption pathways (FDR = 0.0260), were significantly enriched, indicating their potential role in AD pathogenesis. (F) KEGG pathway enrichment analysis of methylated sites. KEGG pathway analysis of the 33 methylated sites highlighted their involvement in key pathways, such as immune responses and signal transduction. Notably, protein digestion and absorption pathways were significantly enriched (FDR = 0.0260), emphasizing the importance of these processes in AD-related epigenetic regulation (Additional Figure 4). AD: Alzheimer’s disease; ADNI: AD Neuroimaging Initiative.

Consistent with findings from the ADNI cohort, the ROSMAP study revealed the significant enrichment of methylation sites within the transcription start and end states of ES-WA7 cells. Furthermore, significant enrichment was observed in the bivalent/poised TSS, flanking bivalent TSS/enhancer, bivalent enhancer, and repressed PolyComb states within pancreas and gastric tissue. In T-cell-related cell lines, such as primary Th memory cells from peripheral blood and primary T cluster of differentiation (CD)8^+^ naive cells, a marked enrichment of zinc finger protein genes and repeats, as well as heterochromatin, was detected (**Figure 9A** and **Additional Table 12**). Histone modifications exhibited highly significant enrichment for right atrium H3K27me3 alterations (odds ratio = 8.88) (**Additional Figure 6E**).

Elevated methylation levels were detected in in the brain-cerebellum for cg06285439 (*NRP2*), cg12699313 (*GNS*), and cg08959039 (*COL4A2*), whereas cg01802545 (*LGMN*) demonstrated the strongest expression specificity (*P*_hyper_ = 0.769) (**Figure 9B** and **Additional Table 14**). Within the brain, cg17913386 demonstrated the strongest correlation between methylation levels and tissue-specific gene expression patterns (Cor = 0.268) (**Figure 9C** and **Additional Table 15**). Methylated sites such as cg04193923 (*CSF3*), cg01802545 (*LGMN*), and cg13491071 (*ROR2*) were predominantly expressed in the nervous system (**Additional Figure 6F–H**). Phenotypically, the most significant associations were observed with AD (*P* = 1.66 × 10^–17^), thus validating the identified loci. Additional associations were observed with preterm birth, smoking, psoriasis, and depression (*P* = 1.22 × 10^–12^, 1.07 × 10^–8^, 6.00 × 10^–7^, and 1.43 × 10^–6^, respectively). Loci linked to aging and cognitive function, which were initially screened for in ADNI, were also enriched in ROSMAP and were associated with neurodevelopmental presentations and congenital anomalies (*P* = 7.78 × 10^–5^, 0.000359, and 0.00168, respectively; **Figure 9D**). GO analysis indicated that these 33 methylated sites were predominantly involved in signal transduction and cell communication pathways (FDR = 0.00157), immune processes (FDR = 0.00696), and protein digestion and absorption pathways (FDR = 0.0260; **Figure 9E**, **9F** and **Additional Table 16**). These epigenetic insights reinforce the involvement of immune, neural, and aging-related processes in AD and provide complementary layers of regulatory evidence.

### Elucidating direct causal gene regulatory relationships through multi-omic mediation analysis

To uncover the potential causal relationships between candidate gene sets and AD pathology, we performed mediation analysis and constructed cell type-specific regulatory networks. In the ADNI cohort, we identified a significant mediation effect of *C8_Hay Bone Marrow Stromal* on CSF_ptau and CSF_tau (*P* < 0.004). In the ROSMAP cohort, several gene sets exhibited causal relationships with AD pathology, including *C8_Zhong PFC Major Types Astrocytes* and DP (*P* < 0.01), *C6_STK33 Up* and p-tau (*P* < 0.001), *C6_Cahoy Neuronal* and Aβ (*P* = 0.024), *C8_Durante Adult Olfactory Neuroepithelium Immature Neurons* and NFT (*P* = 0.024), *C8_Manno Midbrain Neurotypes HNBML1* and NFT/NP (*P* = 0.002 and 0.001, respectively), and *C3-ZFP91 Target Genes* and NP (*P* < 0.001; **Additional Table 17**).

**Figure 10:**
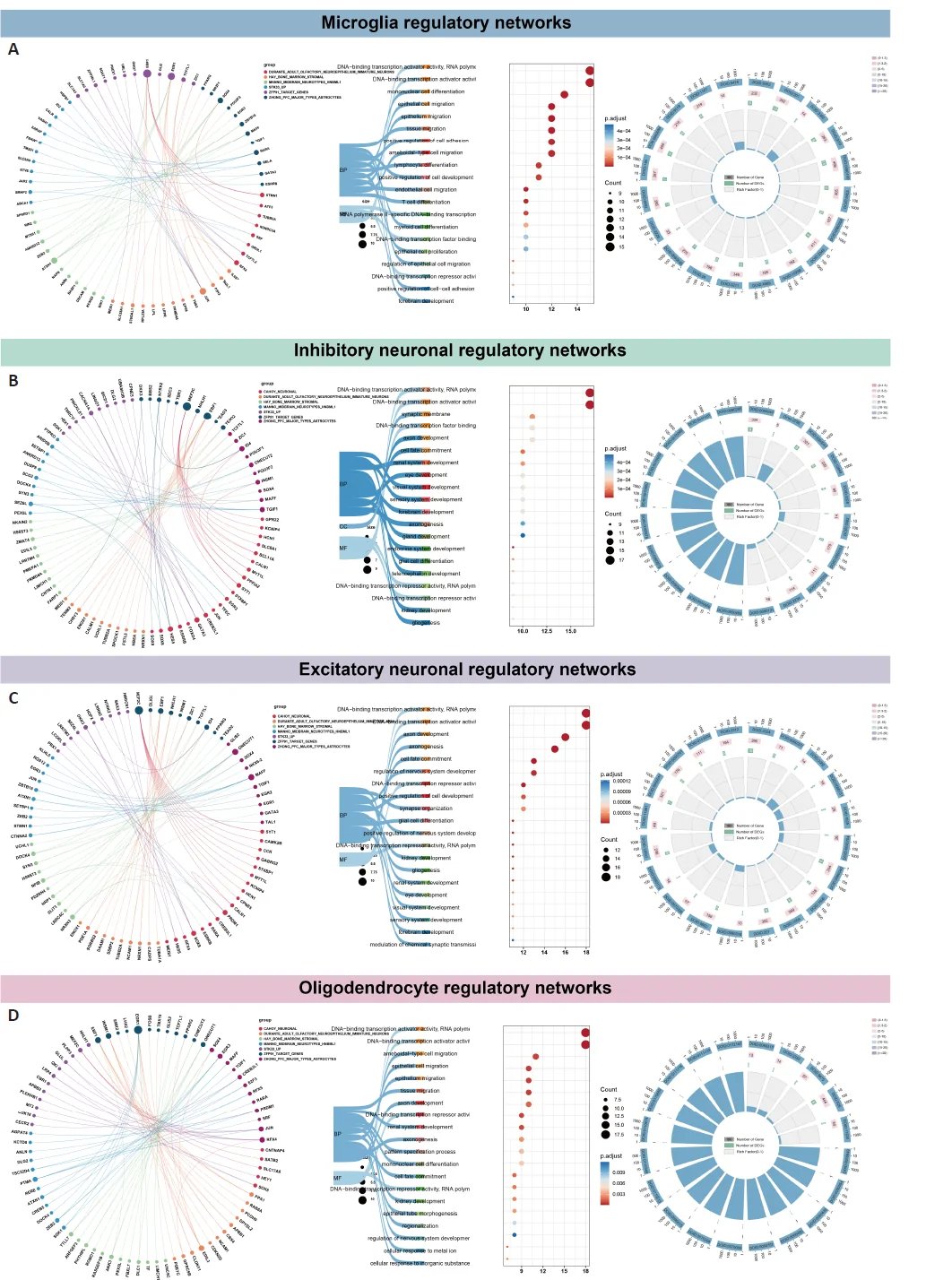
Cell type-specific multiomics regulatory network analysis in neural tissues. Multiomics causal regulatory network analysis revealed distinct regulatory patterns across four major neural cell types. Each cell-specific network (A–D) comprises three components: gene regulatory relationships, functional pathway enrichment, and disease association patterns. (A) In microglia, 64 regulatory pairs were identified, with *ATXN1* and *EBF1* emerging as the most prominent regulators. The *RPL23A-EBF1* pair presented the highest confidence score, indicating strong regulatory interactions. (B) For inhibitory neurons, *MEF2C* regulated the greatest number of genes, and the *ENOX1-TEAD3* pair showed the highest confidence in regulatory relationships. (C) In excitatory neurons, *MEF2C* was again a key regulator, with the pair of *PDE1A-ONECUT1* having the highest confidence score, underscoring its regulatory importance. (D) Oligodendrocytes were characterized by *EGR1* as the main regulator, with the *TTLL7-EGR1* pair exhibiting the highest confidence. Functional enrichment analysis revealed that each cell type regulatory network was associated with specific biological functions, such as miRNA transcription regulation in microglia and axon development in excitatory neurons. Disease enrichment analyses revealed associations with fatty liver disease, mood disorders, and developmental disorders across the different networks, highlighting the diverse implications of these regulatory interactions in neurobiology.

To further explore the regulatory relationships within these gene sets, multi-omic causal networks were constructed for four neural cell types: microglia, inhibitory neurons, excitatory neurons, and oligodendrocytes. Permutation tests identified 89,974 regulatory pairs in microglia (**Figure 10A**), 139,428 in inhibitory neurons (**Figure 10B**), 128,411 in excitatory neurons (**Figure 10C**), and 82,413 in oligodendrocytes (**Figure 10D**). The top 10% of regulatory relationships, ranked by confidence, were then selected for a detailed analysis (**Additional Table 18**). In microglia, we identified 64 regulatory pairs; *ATXN1* and *EBF1* regulated the highest number of target genes (six and nine, respectively), predominantly in the *C8_Manno Midbrain Neurotypes HNBML1* and *C8_Durante Adult Olfactory Neuroepithelium Immature Neurons* gene sets. The RPL23A–EBF1 pair exhibited the highest confidence (coefficient = 1.48) (**Figure 10A**, left). In inhibitory neurons, MEF2C regulated the most target genes (nine), followed by EBF1 with seven; the ENOX1–TEAD3 pair had the highest confidence (coefficient = 6.44) (**Figure 10B**, left). For excitatory neurons, MEF2C again regulated the most target genes (10), followed by ONECUT1 (6); the PDE1A–ONECUT1 pair had the highest confidence (coefficient = 4.16) (**Figure 10C**, left). In oligodendrocytes, EGR1 regulated 10 genes, and the TTLL7–EGR1 pair had the highest confidence (coefficient = 4.01) (**Figure 10D**, left).

Functional analysis revealed the significant enrichment of DNA-binding transcription activator activity across all cellular networks. The microglial subnetwork was predominantly associated with miRNA transcription regulation (FDR = 1.60 × 10^–6^) (**Figure 10A**, middle). The inhibitory neuron subnetwork was mainly linked to endocrine system development (FDR = 2.68 × 10^–6^) (**Figure 10B**, middle). Excitatory neurons were most significantly associated with axon development (FDR = 1.98 × 10^–8^) (**Figure 10C**, middle). By contrast, the oligodendrocyte network was most significantly enriched for epithelial cell migration (FDR = 0.000647) (**Figure 10C**, middle; **Additional Table 19**). Furthermore, disease enrichment analysis revealed that the microglial subnetwork was most significantly associated with fatty liver disease, connective tissue cancer, and liposarcoma (FDR = 0.001) (**Figure 10A**, right). The inhibitory neuron subnetwork was linked to pervasive developmental disorder (FDR = 0.0343), followed by cerebellar disease and autism spectrum disorder (FDR = 0.0493) (**Figure 10B**, right). Excitatory neuron subnetworks were enriched for mood disorder (FDR = 0.005), autosomal dominant intellectual developmental disorder, and liposarcoma (FDR = 0.0117) (**Figure 10C**, right; **Additional Table 20**). Overall, these causal inference results highlight the critical regulatory hubs and pathways involved in AD, thereby deepening our understanding of its molecular complexity at the network level.

## Discussion

AD is a multifactorial neurodegenerative disorder that is caused by complex mechanisms that are not completely understood. Traditional models—most notably, the amyloid cascade hypothesis—emphasize Aβ plaque accumulation as the primary driver of AD pathology. However, this hypothesis does not adequately explain the other clinical manifestations in patients with AD, including neuronal degradation, synaptic dysfunction, and altered metabolic activity. In the current research, we present a new paradigm that places mitochondrial dysfunction and immune activation as central contributors to the development of AD. Using machine learning methodologies and data from various AD cohorts, our results identify important genetic factors linked to mitochondrial and immune dysfunction in AD, thus further expanding our understanding of the underlying AD pathogenesis.

Using our exhaustive machine learning screening approach, we identified multiple significant gene sets related to AD pathogenesis. Of the 10 machine learning models that were evaluated, the RF model demonstrated the highest predictive accuracy, with AUC values ranging from 0.734 to 0.846. When we conducted a cross-dataset analysis, we identified the greatest cross-over between the MSBB and Mayo datasets; a select few important variables maintained a significant relevance to AD risk, including the *C6_Cahoy Neuronal* gene set, which repeatedly demonstrated the importance of neuronal function and synaptic integrity that likely contributes to AD development. Our findings support previous studies that have identified neuronal loss and synaptic dysfunction as an important contributor to cognitive decline (Calvo-Rodriguez and Bacskai, 2021). Furthermore, it is important to emphasize the relevance of the *C4_Module 229*, with ATPase activity, to mitochondrial dysfunction as a central factor in AD. Mitochondrial dysfunction results in neuronal energy deficits, oxidative stress, and synaptic degeneration, which are all features of AD pathology. These findings are therefore consistent with prior studies that highlighted mitochondrial dysfunction as a central mechanism in neurological disease (Weidling and Swerdlow, 2020).

Our cross-dataset enrichment analysis indicated that immune activation is a central dysregulated function in AD and highlighted neutrophil degranulation as the most upregulated factor. As previously noted, neuroinflammation that is mediated by activated immune cells (such as neutrophils and microglia) accelerates the progression of AD (Zenaro et al., 2015; Le Page et al., 2017; Dong et al., 2018; Aries and Hensley-McBain, 2023). Similar to this finding, gene sets related to epithelial–mesenchymal transition, TNF-α signaling, and nuclear factor (NF)-κB also point to the role of inflammation, and—more specifically—to its role in Aβ accumulation and tau hyperphosphorylation (Nafea et al., 2023). Moreover, our analysis highlighted the downregulation of pathways involved in respiratory electron transport, including complex I biogenesis, indicating that mitochondrial dysfunction is likely implicated in neuronal energy deficits and oxidative stress in AD. This finding aligns with previous studies that emphasize metabolic disturbances as key factors in AD progression (Fišar et al., 2016). Furthermore, the implications of the downregulation of these mitochondrial pathways reinforces the need for novel therapeutic approaches that target mitochondrial health (Perluigi et al., 2024).

The present study also identified several key genes, including *SGK1*, *LRRK1*, and *NFKB1* (all of which are mitochondrially localized), and highlighted the intricate mechanisms of mitochondrial failure and immune activation pathways in AD. These genes are associated with the regulation of immune activation, inflammation, cognitive stress responses, and neurodegeneration (Zenaro et al., 2015; Le Page et al., 2017; Dong et al., 2018; Aries and Hensley-McBain, 2023). Notably, SGK1 expression is increased in the aging human brain, and is activated in the development of tau pathology, which may involve the formation of serine/threonine-protein kinase Sgk1 (SGK1)–glycogen synthase kinase-3 beta (GSK-3β)–tau complexes (Elahi et al., 2021). This suggests that mitochondrial defects may lead to accelerated tau pathology by facilitating the formation of harmful protein complexes that lead to cognitive decline. Equally important is the emergence of *LRRK1*, a lesser-known homolog of the Parkinson’s disease-associated gene *LRRK2*. Although the role of *LRRK2* in Parkinson’s disease is well-established (Henderson et al., 2019; Mamais et al., 2024), *LRRK1* has remained largely unexplored in AD. Our findings, which are supported by preliminary evidence, suggest that *LRRK1* may drive neurodegeneration through similar mechanisms of mitochondrial disruption and neuroinflammation (Small and Petsko, 2020). This may open up new avenues for research linking mitochondrial defects in Parkinson’s disease and AD with shared pathogenic processes. NF-κB is a ubiquitous regulator of inflammation that was also identified in the current study. The activation of NF-κB is associated with neuroinflammation that both accelerates Aβ accumulation and tau hyperphosphorylation in AD. When persistent, NF-κB activity leads to chronic neuroinflammation that ultimately hastens AD disease progression (Novoa et al., 2022). Recent evidence suggests that mitochondrial dysfunction and immune activation pathways are correlated, indicating that co-targeting both pathways may represent an effective therapeutic approach for AD (Perluigi et al., 2024).

Further investigation into gene sets from the MSigDB cohort provided crucial insights. Gene sets from Clusters C4, C5, and C6 were heavily enriched for immune responses and inflammation. A comparative analysis across our datasets consistently flagged genes such as *TGFB2*, *DUSP1*, and *CXCL1* as critical genetic regulators. *TGFB2* and *DUSP1* modulate mitochondrial quality control and mitogen-activated protein kinase regulation, respectively, thereby underscoring their relevance in AD progression (von Bernhardi et al., 2015; Iloun et al., 2021; Kayhan et al., 2023). *CXCL1* has been associated with increased tau cleavage and is potentially a therapeutic target for tauopathies (Zhang et al., 2013, 2015). The ADNI dataset had fewer DEGs than the MSBB dataset, and the consistent appearance of *TGFB2* and *DUSP1* across the cohorts strengthens the case for their importance. This cross-validation also implicated other mitochondrial-linked genes, such as *COL1A1*, which was notably present in the Mayo dataset (Comba et al., 2022).

We also identified important relationships between particular gene sets and CSF biomarkers, such as *C8_HAY Bone Marrow Stromal* and *C8_Zhong PFC Major Types Astrocytes*, that correlated with CSF Aβ levels. This suggests that astrocytes have a critical role in modulating Aβ toxicity (Liddelow et al., 2017). In addition, our polygenic risk assessment indicated strong correlations with age-related macular degeneration, suggesting that inflammation, oxidative stress, and Aβ aggregation might be shared mechanisms that help to support mitochondrial dysfunction and immune dysregulation, and might represent a shared pathway of cognitive decline for both AD and other neurodegenerative diseases (Blasiak et al., 2022; Kaštelan et al., 2024).

Our analysis of whole-transcriptome methylation profiles revealed distinct epigenetic fingerprints in AD-related genes, particularly within crucial nervous system pathways such as PI3K–Akt signaling. These results further support the idea that epigenetic regulation is important for understanding AD progression, and that a lack of mitochondrial integrity may represent a disorder in terms of maintaining cellular functions in the brain. The multi-omic mediation analysis further illustrated the role of microglial gene expression in neuroinflammation and synaptic pruning, with miRNA transcriptional regulation as the predominant factor during the AD development process (Li et al., 2024). Although this research provides a valuable and detailed overview of AD pathology, future research is required. In the present study, we described several innovations and improvements relative to previous AD research. One major advance is our use of data from independent, multi-AD cohorts, thus allowing us to limit the biases that come from executing a single cohort study and improve the generalizability of our results. In contrast to many previous studies that explored specific datasets or populations, the current work used a cross-dataset approach. As a result, the gene sets and molecular pathways that were identified were consistent, despite studying several heterogeneous groups of patients. This aspect of our work is essential for generating knowledge to develop therapeutic strategies to ameliorate AD on a population scale. Furthermore, we applied machine learning-based advanced GSEA, which helps to elucidate complex, non-linear relationships among gene sets and AD pathogenesis that would not normally be captured using traditional statistical approaches. Machine learning-based screening can be successful for identifying new biomarkers and pathways related to complex diseases such as AD, because machine learning can represent a more dynamic and less systematic understanding of the disease (Kavitha et al., 2022; Martin et al., 2023). The inclusion of multi-omic data, particularly whole-transcriptome methylation data, offered a system-level understanding of how epigenetic regulation orchestrates the genetic risk of AD, which is a crucial aspect that has been highlighted in recent landmark studies (De Jager et al., 2018; Bai et al., 2020; Kumar et al., 2021).

Furthermore, although others have noted the importance of mitochondria (Calvo-Rodriguez and Bacskai, 2021; Cheng et al., 2022; Ashleigh et al., 2023), our study places a much stronger emphasis on mitochondrial dysfunction as a core, initiating mechanism of AD. We identified mitochondria-associated genes, such as *TGFB2*, *DUSP1*, *CXCL1*, *TGFB2*, *SGK1*, and *LRRK1*, that have been understudied in AD but are important for the mitochondrial DNA repair and quality control system. On the basis of these findings, we reinforce the idea that mitochondrial health is essential for delaying the progression of AD. Our findings are congruent with previous studies that have implicated mitochondrial dysfunction as playing a major role in neurodegenerative diseases (Wang et al., 2018b; Ashleigh et al., 2023). However, our findings have highlighted specific genes that may serve as new therapeutic targets for improving mitochondrial function when treating AD. Additionally, our studies confirm the role of immune dysregulation—especially neutrophil activation and epithelial–mesenchymal transition—in mediating neuroinflammation, which is key to the development of AD. Although the involvement of immune activation and inflammation in AD has been described previously (Heppner et al., 2015, 2015; Novoa et al., 2022), our findings offer important evidence that chronic neuroinflammation induced by immune cells, such as neutrophils, can promote disease progression (Aries and Hensley-McBain, 2023). Together, this strengthens the idea that targeting the immune system, along with mitochondrial health, may be highly therapeutically beneficial. Furthermore, our results provide a novel understanding of the polygenic risk of AD by delineating uniquely shared genetic pathways with other age-related diseases, such as age-related macular degeneration. Both AD and age-related macular degeneration have similar pathological pathways and common etiological mechanisms, including oxidative stress and Aβ aggregation, which are common risk factors that can be researched holistically. There may therefore be opportunities to develop therapeutic strategies for both AD and other neurodegenerative diseases by targeting mitochondrial dysfunction and immune dysregulation (Atamna and Frey, 2007; Beamer and Shepherd, 2012; Banoth and Cassel, 2018; Weidling and Swerdlow, 2020; Blasiak et al., 2022). Overall, the multi-layered approach used in the present study offers new knowledge about AD pathogenesis, and highlights new avenues for therapeutic targets in the future.

## Limitations

The current study has some limitations. Although the strength of our work lies in the integration of four independent AD cohorts, this is also a source of unavoidable complexity. Each cohort has its own unique history, with differences in recruitment strategies, sample collection, and demographic makeup. Although we worked to account for these differences, they introduce a layer of heterogeneity that might still influence our findings and limit their direct applicability to all populations. A clear path forward for the field is the adoption of more harmonized, multi-cohort designs that prioritize sample diversity and standardized protocols from the outset. Furthermore, we must approach the outputs of our machine learning models with caution; these algorithms are exceptionally skilled at identifying complex patterns within high-dimensional data, but this power comes with the risk of overfitting. The vast and interconnected nature of gene regulatory networks means that some of the relationships that we identified may be statistically sound but not biologically causal. Additionally, a critical limitation of our cross-sectional design is that the immune and mitochondrial pathway alterations we observed were primarily detected in patients who already had established AD. This temporal constraint means we cannot definitively distinguish whether these changes represent causative factors in AD development or consequential effects that occur as a result of the disease process. Future longitudinal studies tracking individuals from preclinical stages through disease progression will be essential to establish the causal directionality of these relationships. We therefore view our findings not as definitive conclusions, but as high-priority, data-driven hypotheses that require rigorous experimental validation. Lastly, the biological scope of our investigation was intentionally focused on the transcriptome and epigenome. Although powerful, this lens does not capture the full story. Crucial events in AD pathology, such as post-translational modifications of proteins (e.g., tau) or the intricate dance of PPI, were outside the scope of this analysis. Our model of AD is therefore a detailed but incomplete snapshot. The next frontier will be to expand this integrative framework, weaving in layers of proteomics and metabolomics. Only by embracing this multi-modal complexity can we hope to develop effective biomarkers and therapies that target the dynamic, interconnected processes at the heart of AD.

## Conclusions

Our study used computational methods to identify gene sets related to AD pathogenesis in four independent datasets, with important findings of consistent relationships with neuronal function and mitochondrial function. Our results point to the importance of neuronal health in slowing AD-related decline. Specific gene sets, such as *C6_Cahoy Neuronal*, underscore the importance of synaptic integrity. Moreover, mitochondrial dysfunction, linked to ATPase activity and oxidative stress, emerged as a core pathogenic mechanism. Immune activation was also identified as a critical contributor to AD, particularly through neutrophil degranulation mechanisms. This was combined with evidence of inhibited mitochondrial functioning. The cross-dataset analyses included critical regulatory genes, such as *TGFB2*, *DUSP1*, and *CXCL1*, that regulate mitochondrial quality, the immune response, and tau pathology. Collectively, our integration of genetic, epigenetic, and transcriptomic data reveals new dimensions of AD mechanisms and suggests that mitochondrial and immune pathways may be novel therapeutic targets for future interventions.

## Additional files:

***Additional Table 1:*** *Basic characteristics of samples from the ADNI, ROSMAP, MSBB and Mayo cohorts.*

Additional Table 1 Basic characteristics of samples from the ADNI, ROSMAP, MSBB and Mayo cohorts.

***Additional Table 2:*** *Model performance of ten machine learning models for predicting AD risk.*

Additional Table 2 Model performance of ten machine learning models for predicting AD risk.

***Additional Table 3:*** *Modeled importance of each class of important gene collections in the MSigDB and Disease databases.*

Additional Table 3 Modeled importance of each class of important gene collections in the MsigDB and Disease databases

***Additional Table 4:*** *Biological function enrichment results for differential genes and important characterization genes based on Reactome.*

Additional Table 4 Biological function enrichment results for differential genes and important characterization genes based on Reactome

***Additional Table 5:*** *PathwayIenrichment results for differential genes and important characterization genes based on Reactome.*

Additional Table 5 PathwayIenrichment results for differential genes and important characterization genes based on Reactome

***Additional Table 6:*** *Comparison of biological functions between each category.*

Additional Table 6 Comparison of biological functions between each category

***Additional Table 7:*** *Summary of genes in the set of significant features associated with AD pathology indicators.*

Additional Table 7 Summary of genes in the set of significant features associated with AD pathology indicators

***Additional Table 8:*** *Summary of a collection of disease genes significantly associated with AD pathology.*

Additional Table 8 Summary of a collection of disease genes significantly associated with AD pathology

***Additional Table 9:*** *Biological functions of important disease signature genes.*

Additional Table 9 Biological functions of important disease signature genes

***Additional Table 10:*** *Summary of significant CpGs for epigenome-wide association analysis based on ADNI and ROSMAP cohorts.*

Additional Table 10 Summary of significant CpGs for epigenome-wide association analysis based on ADNI and ROSMAP cohorts.

***Additional Table 11:*** *Epigenome-wide association analysis results of important characterization genes corresponding to CpGs.*

Additional Table 11 Epigenome-wide association analysis results of important characterization genes corresponding to CpGs

***Additional Table 12:*** *Enrichment results of chromatin state for important CpGs loci.*

Additional Table 12 Enrichment results of chromatin state for important CpGs loci

***Additional Table 13:*** *Enrichment results of histone modifications at important CpGs sites.*

Additional Table 13 Enrichment results of histone modifications at important CpGs sites

***Additional Table 14:*** *Tissue-specific annotation of important CpGs loci.*

Additional Table 14 Tissue-specific annotation of important CpGs loci

***Additional Table 15:*** *Relationship between methylation levels of important CpGs loci and nearby gene expression.*

Additional Table 15 Relationship between methylation levels of important CpGs loci and nearby gene expression

***Additional Table 16:*** *Enrichment results of important CpGs loci for traits and biological functions.*

Additional Table 16 Enrichment results of important CpGs loci for traits and biological functions

***Additional Table 17:*** *Results of mediation analysis of AD pathologic features and disease gene sets.*

Additional Table 17 Results of mediation analysis of AD pathologic features and disease gene sets

***Additional Table 18:*** *Important regulatory relationships in four cell-specific gene regulatory networks.*

Additional Table 18 Important regulatory relationships in four cell-specific gene regulatory networks.

***Additional Table 19:*** *Biological function enrichment of important nodes of four cell-specific gene regulatory networks.*

Additional Table 19 Biological function enrichment of important nodes of four cell-specific gene regulatory networks

***Additional Table 20:*** *Disease enrichment of important nodes of four cell-specific gene regulatory networks.*

Additional Table 20 Disease enrichment of important nodes of four cell-specific gene regulatory networks

***Additional Figure 1:*** *Performance evaluation of survival models for AD risk assessment across ADNI and ROSMAP cohorts.*

Additional Figure 1 Performance evaluation of survival models for AD risk assessment across ADNI and ROSMAP cohorts.
Model performance for predicting AD risk based on important variables identified by the Boruta algorithm was presented. Data were obtained from two independent cohorts: ADNI (left panel) and ROSMAP (right panel). Survival analysis was performed using five distinct algorithms: Cox proportional hazards model, Random Forest, XGBoost, DeepSurv, and DeepHit. Each row (C2 to C8, and H) represents a unique combination of selected variables analyzed, with corresponding sub-panels displaying predicted survival curves and performance metrics, including the concordance index (C-index), Brier score, and area under the concordance/discrimination curve (C/D AUC). The accompanying bar charts highlight model performance rankings based on these metrics, revealing cohort-specific variations in predictive efficacy and the differential impact of selected variables on AD risk assessment.

***Additional Figure 2:*** *Survival model evaluation for AD risk prediction across MSBB and Mayo cohorts.*

Additional Figure 2 Survival model evaluation for AD risk prediction across MSBB and Mayo cohorts.
Building on the findings from Figure S2, data were sourced from two independent cohorts—MSBB (left panel) and Mayo (right panel). Our study utilized five distinct algorithms: Cox proportional hazards model, Random Forest, XGBoost, DeepSurv, and DeepHit, to conduct survival analyses. Each row (C2 to C8, H, and D) represented a different set of selected variables. The sub-panels illustrated the predicted survival curves alongside key performance metrics, including the concordance index (C-index), Brier score, and area under the concordance/discrimination curve (C/D AUC). Bar charts highlighted the top three models exhibiting the highest performance for each variable combination, emphasizing the variation in model efficacy between the MSBB and Mayo cohorts. This extended analysis aimed to further elucidate the influence of key variables on AD risk prediction, reinforcing the insights gained from the previous evaluation in Additional Figure 2.

***Additional Figure 3:*** *Pathway convergence analysis across AD cohorts reveals shared and distinct molecular signature.*

Additional Figure 3 Pathway convergence analysis across AD cohorts reveals shared and distinct molecular signature.
Comprehensive analysis of pathway enrichment across ADNI, ROSMAP, MSBB, and Mayo datasets revealed distinct patterns of intersection within nine MsigDB and Disease categories (C2-C8, H, D). The highest pathway overlap was observed between MSBB and Mayo datasets within category C6 (13 intersections). The variables with the highest enrichment scores for each category were as follows: *C2_Reactome Keratan Sulfate Degradation* (*IMP^all^* = 58.014), *C3_MIR6794 5P* (*IMP^all^* = 57.485), *C4_Module 229* (*IMP^all^* = 30.868), *C5_GOCC DNA Packaging Complex* (*IMP^all^* = 30.540), *C6_Cahoy* *Neuronal* (*IMP^all^* = 89.410), *C7_GSE21546 WT versus SAP1A KO ELK1 KO Thymocytes Downregulated* (*IMP^all^* = 59.149), *C8_HAY Bone Marrow* *Stromal* (*IMP^all^* = 58.859), *H_Hallmark IL6 JAK STAT3 Signaling* (*IMP^all^* = 57.960), and *D_Rheumatoid Arthritis* (*IMP^all^* = 58.699). These results underscored both distinct and overlapping pathways enriched across different AD datasets, reflecting the heterogeneity of disease mechanisms. Notably, the MSBB and Mayo cohorts exhibited the most significant intersections in several categories, suggesting shared biological processes. The findings highlight the involvement of various biological functions, including inflammatory signaling (e.g., HALLMARK_IL6_JAK_STAT3_SIGNALING) and processes related to cellular differentiation and immune responses.

***Additional Figure 4:*** *Cross-cohort analysis of hallmark gene sets reveals conserved pathway alterations in AD.*

Additional Figure 4 Cross-cohort analysis of hallmark gene sets reveals conserved pathway alterations in AD.
Hallmark gene set analysis based on differentially expressed genes across four AD datasets: ADNI (A), ROSMAP (B), MSBB (C), and Mayo (D). The network graphs illustrated the significantly upregulated and downregulated hallmark pathways. Gene sets EPITHELIAL MESENCHYMAL TRANSITION, INFLAMMATORY RESPONSE, and TNFA SIGNALING VIA NFKB were significantly upregulated across all four datasets. OXIDATIVE PHOSPHORYLATION was significantly downregulated, indicating shared mitochondrial dysfunction in AD pathology. Among the important genes analyzed, KRAS SIGNALING UP (co-occurring in ADNI, MSBB, and Mayo), was the most frequently noted in both up- and down-regulation analyses. Additionally, the TNFA SIGNALING VIA NFKB pathway was co-downregulated in ADNI, MSBB, and Mayo. Overall, gene sets related to signal transduction accounted for the largest proportion of up- and down-regulated changes, highlighting the role of signaling pathways in AD mechanisms across different cohorts.

***Additional Figure 5:*** *Nomogram-based prediction of AD onset incorporating comorbidity profiles in ADNI and ROSMAP cohorts.*

Additional Figure 5 Nomogram-based prediction of AD onset incorporating comorbidity profiles in ADNI and ROSMAP cohorts.
(A) The nomogram for the ADNI cohort illustrated that comorbid conditions, particularly age-related macular degeneration and mitochondrial complex I deficiency, significantly influenced the predicted age of onset for AD. Other diseases, such as amyotrophic lateral sclerosis, ovarian cancer, and Noonan syndrome, also contributed to the prediction model, albeit to varying extents. Gender and age were shown to be moderate predictors, with males exhibiting slightly higher predictive points for AD onset. The cumulative effect of these conditions was reflected in the total points and the corresponding odds ratio at the bottom, suggesting how various factors collectively impacted the risk for developing AD. (B) The ROSMAP nomogram further supported the predictive power of comorbid conditions in determining AD onset, highlighting the significant influence of rheumatoid arthritis and Joubert syndrome (*P* < 0.001). Other conditions, such as Ehlers-Danlos syndrome and Brugada syndrome, also played notable roles in the prediction. Gender differences were emphasized, with the male population showing slightly higher risk odds in certain conditions. As with ADNI, the total points scale provided an overall risk estimate, confirming the multifactorial nature of AD development in the ROSMAP cohort. Both nomograms demonstrated the importance of considering a wide range of genetic and environmental comorbidities to accurately predict AD onset across different populations.

***Additional Figure 6:*** *Tissue-specific methylation patterns and genomic distributions in ADNI and ROSMAP cohorts.*

Additional Figure 6 Tissue-specific methylation patterns and genomic distributions in ADNI and ROSMAP cohorts.
(A) Histone modification enrichment analysis indicated that the key loci were predominantly associated with H3K27me3 modifications across various tissues and cell lines, with particularly strong enrichment observed in pancreatic tissue and primary hematopoietic stem cells. (B)f specific sites in neural tissues, notably cg02482042 (*ATP2B2*), cg07472953 (*CSF3*), and cg22132982 (*WNT7A*), which were predominantly found in the nervous system. These sites were significantly associated with pathways like Viral Myocarditis, indicating potential roles in neurological and immune regulation. (C) A bubble plot displaying the *P*-values for correlations, highlighting brain-specific loci. The three most significant loci in the brain were cg09211763, cg04069951, and cg13411522, with larger bubbles indicating greater significance. (D) Genomic position analysis showed that the majority of these methylation loci were located in intergenic regions, suggesting regulatory roles in gene expression rather than direct coding effects. (E) Similar to ADNI, histone modifications result for the ROSMAP dataset showed significant enrichment for H3K27me3 alterations specifically in the Right Atrium, with an odds ratio of 8.88. (F) Neural tissue-specific methylation patterns emerged for sites including cg04193923 (*CSF3*), cg01802545 (*LGMN*), and cg13491071 (*ROR2*), were predominantly expressed in the nervous system, aligning with findings from the ADNI cohort. (G) A bubble plot of *P*-values demonstrating significant associations in the brain, with cg15901783 being the most strongly associated locus. Other significant associations were concentrated in the kidney and liver. (H) Genomic position analysis in the ROSMAP cohort also revealed that most of the identified loci were located in intergenic regions, further supporting their involvement in gene regulation.

## Data Availability

*The datasets analyzed in this study are publicly available from recognized repositories: the Alzheimer’s Disease Neuroimaging Initiative (ADNI, https://adni.loni.usc.edu/), the Religious Orders Study and Memory and Aging Project (ROSMAP, https://www.radc.rush.edu/), the Mount Sinai Brain Bank (MSBB, https://www.synapse.org/#!Synapse:syn3159438), and the Mayo Clinic Alzheimer’s Disease Genetics Study (MAYO, https://www.synapse.org/#!Synapse:syn5550404). Data generated or analyzed during this study, including gene enrichment results and selected genetic markers, are available from the corresponding author upon reasonable request.*
