# Supplementary material for: Genetic and pathway complexity in Alzheimer’s disease: Insights from multi-omic data about the immune response and mitochondrial function
Source: Neural Regen Res. 2025 Sep 29;21(9):4406–23. doi: 10.4103/NRR.NRR-D-25-00184 (PMC13557549; doi:10.4103/NRR.NRR-D-25-00184)
Supplement: Supplementary file 1 [file NRR-21-4406_Suppl1.pdf]

Additional Table 1 Basic characteristics of samples from the ADNI, ROSMAP, MSBB and Mayo cohorts.

ADNI RNA-seq	CN	MCI	Dementia	*P*.overall
	n=246	n=383	n=114	
**RID**	2737 (1880)	2938 (1470)	2104 (1813)	<0.001
**COLPROT**				0.002
ADNI2	171 (69.5%)	213 (55.6%)	65 (57.0%)	
ADNIGO	75 (30.5%)	170 (44.4%)	49 (43.0%)	
**Age**	74.3 (5.75)	72.0 (7.42)	74.6 (7.58)	<0.001
**Education**	16.4 (2.68)	16.0 (2.79)	16.0 (2.94)	0.129
**Ethnicity**				0.707
Unknown	1 (0.41%)	1 (0.26%)	1 (0.88%)	
Not Hisp/Latino	241 (98.0%)	372 (97.1%)	110 (96.5%)	
Hisp/Latino	4 (1.63%)	10 (2.61%)	3 (2.63%)	
**PTGENDER**				0.006
Female	129 (52.4%)	167 (43.6%)	40 (35.1%)	
Male	117 (47.6%)	216 (56.4%)	74 (64.9%)	
**Race**				0.256
Am Indian/Alaskan	1 (0.41%)	1 (0.26%)	0 (0.00%)	
Asian	2 (0.81%)	5 (1.31%)	4 (3.51%)	
Hawaiian/Other PI	0 (0.00%)	2 (0.52%)	0 (0.00%)	
Black	16 (6.50%)	11 (2.87%)	3 (2.63%)	
White	226 (91.9%)	357 (93.2%)	106 (93.0%)	
More than one	1 (0.41%)	5 (1.31%)	1 (0.88%)	
Unknown	0 (0.00%)	2 (0.52%)	0 (0.00%)	
**Years.bl**	1.96 (2.33)	0.98 (1.88)	2.79 (2.24)	<0.001
**DXCHANGE**				.
Conversion: MCI to Dementia	0 (0.00%)	0 (0.00%)	17 (14.9%)	
Conversion: NL to MCI	0 (0.00%)	10 (2.61%)	0 (0.00%)	
Reversion: Dementia to MCI	0 (0.00%)	1 (0.26%)	0 (0.00%)	
Reversion: MCI to NL	2 (0.81%)	0 (0.00%)	0 (0.00%)	
Reversion: MCI to NL	0 (0.00%)	0 (0.00%)	61 (53.5%)	
Stable: Dementia to Dementia	0 (0.00%)	0 (0.00%)	36 (31.6%)	
Stable: MCI	0 (0.00%)	213 (55.6%)	0 (0.00%)	
Stable: MCI to MCI	0 (0.00%)	159 (41.5%)	0 (0.00%)	
Stable: NL	171 (69.5%)	0 (0.00%)	0 (0.00%)	
Stable: NL to NL	73 (29.7%)	0 (0.00%)	0 (0.00%)	
**Age**	76.2 (6.50)	73.0 (7.96)	77.4 (7.71)	<0.001
**DX**				<0.001
AD	0 (0.00%)	0 (0.00%)	114 (100%)	
nonAD	246 (100%)	383 (100%)	0 (0.00%)	

ROSMAP RNA-seq		AD	Control	*P*.overall
		n=152	n=86	
**msex**				0.028
	0	106 (69.7%)	47 (54.7%)	
	1	46 (30.3%)	39 (45.3%)	
**cogdx**				<0.001
	1	0 (0.00%)	86 (100%)	
	4	152 (100%)	0 (0.00%)	
**Apoe_genotype**				<0.001
	0	89 (58.6%)	77 (89.5%)	
	1	61 (40.1%)	8 (9.30%)	
	2	2 (1.32%)	1 (1.16%)	
**Age_death**		88.2 (3.14)	83.4 (5.85)	<0.001
**Educ**		16.2 (3.39)	16.4 (3.60)	0.817

MSBB RNA-seq		AD	CT	Other	*P*.overall
		n=589	n=273	n=385	
**Sex**					<0.001
	Female	367 (62.3%)	149 (54.6%)	279 (72.5%)	
	Male	222 (37.7%)	124 (45.4%)	106 (27.5%)	
**Apoe4_allele**					<0.001
	0	335 (56.9%)	215 (78.8%)	264 (68.6%)	
	1	197 (33.4%)	58 (21.2%)	121 (31.4%)	
	2	57 (9.68%)	0 (0.00%)	0 (0.00%)	
**Age_death**		83.5 (7.14)	79.8 (9.03)	85.5 (5.77)	<0.001

Mayo RNA-seq		AD	CONTROL	PATH_AGE	PSP	*P*.overall
		n=159	n=143	n=58	n=163	
**Sex**						0.003
	Female	96 (60.4%)	70 (49.0%)	33 (56.9%)	66 (40.5%)	
	Male	63 (39.6%)	73 (51.0%)	25 (43.1%)	97 (59.5%)	
**APOE4**						.
	0	76 (47.8%)	124 (86.7%)	39 (67.2%)	140 (85.9%)	
	1	71 (44.7%)	19 (13.3%)	19 (32.8%)	21 (12.9%)	
	2	12 (7.55%)	0 (0.00%)	0 (0.00%)	2 (1.23%)	
**Ageat death (yr)**		82.5 (7.64)	82.5 (8.40)	85.0 (4.25)	74.0 (6.43)	<0.001
**Tissue**						0.993
CBE		79 (49.7%)	72 (50.3%)	28 (48.3%)	82 (50.3%)	
TCX		80 (50.3%)	71 (49.7%)	30 (51.7%)	81 (49.7%)	

ROSMAP genome cohorts		Control	MCI	AD	Other	*P*.overall
		n=376	n=288	n=471	n=21	
**Amyloid**		2.83 (3.49)	3.78 (4.00)	5.49 (4.32)	3.78 (5.55)	<0.001
**plaq_n**		0.48 (0.60)	0.71 (0.70)	1.21 (0.92)	0.54 (0.64)	<0.001
**plaq_d**		0.58 (0.74)	0.71 (0.84)	0.92 (0.79)	0.51 (0.70)	<0.001
**nft**		0.31 (0.40)	0.52 (0.55)	1.00 (0.96)	0.35 (0.30)	<0.001
**tangles**		3.18 (3.77)	4.96 (5.27)	10.9 (10.3)	2.86 (2.83)	<0.001
**AD**		0.00 (0.00)	0.00 (0.00)	1.00 (0.00)	0.00 (0.00)	0
**AGE_AD**		86.1 (6.68)	88.0 (6.39)	86.8 (6.57)	84.2 (6.16)	0.001
**GENDER**						0.227
	0	241 (64.1%)	178 (61.8%)	322 (68.4%)	12 (57.1%)	
	1	135 (35.9%)	110 (38.2%)	149 (31.6%)	9 (42.9%)	
**APOE**		-0.03 (0.63)	0.09 (0.58)	0.25 (0.69)	0.05 (0.67)	<0.001
**cts_mmse30_lv**		28.0 (1.73)	25.6 (3.22)	12.6 (8.56)	13.8 (7.67)	<0.001
**APOE_**						.
	22	3 (0.80%)	1 (0.35%)	3 (0.64%)	0 (0.00%)	
	23	67 (17.8%)	31 (10.8%)	43 (9.13%)	4 (19.0%)	
	24	4 (1.06%)	5 (1.74%)	10 (2.12%)	1 (4.76%)	
	33	246 (65.4%)	193 (67.0%)	260 (55.2%)	11 (52.4%)	
	34	52 (13.8%)	56 (19.4%)	141 (29.9%)	5 (23.8%)	
	44	4 (1.06%)	2 (0.69%)	14 (2.97%)	0 (0.00%)	

ADNI genome cohorts		AD	Non AD	*P*.overall
		n=864	n=555	
**APOE**				.
	22	2 (0.23%)	0 (0.00%)	
	23	80 (9.26%)	16 (2.88%)	
	24	13 (1.50%)	13 (2.34%)	
	33	473 (54.7%)	172 (31.0%)	
	34	246 (28.5%)	262 (47.2%)	
	44	50 (5.79%)	92 (16.6%)	
**Gender**				0.632
	Female	492 (56.9%)	324 (58.4%)	
	Male	372 (43.1%)	231 (41.6%)	
**AGE**		77.5 (7.33)	74.8 (8.11)	<0.001
**Tau_Abeta_age**		54.6 (36.4)	50.4 (39.5)	0.046
**Education**		16.1 (3.15)	14.9 (4.51)	<0.001
**Abeta**		844 (621)	458 (403)	<0.001
**tau**		195 (154)	251 (209)	<0.001
**ptau**		16.2 (17.9)	22.2 (24.4)	<0.001

Table notes：AD: Alzheimer's disease; ADNI: AD Neuroimaging Initiative; Mayo: Mayo Clinic AD Genetics Studies; MSBB: Mount Sinai Brain Bank; ROSMAP: Religious Orders Study and Rush Memory and Aging Project.
