# Supplementary material for: Genetic and pathway complexity in Alzheimer’s disease: Insights from multi-omic data about the immune response and mitochondrial function
Source: Neural Regen Res. 2025 Sep 29;21(9):4406–23. doi: 10.4103/NRR.NRR-D-25-00184 (PMC13557549; doi:10.4103/NRR.NRR-D-25-00184)
Supplement: Supplementary file 2 [file NRR-21-4406_Suppl10.pdf]

Additional Table 10 Summary of significant CpGs for epigenome-wide association analysis based on ADNI and ROSMAP cohorts.

Additional Table 10 Summary of significant CpGs for epigenome-wide association analysis based on ADNI and ROSMAP cohorts.

ADNI

AD vs CN	chromosom	position	X	p.value.non	p.value.all	p.value.isva	p.value.sva	coefficient.non	coefficient.al	coefficient.isv	coefficient.sv	p.value	fdr	p.holm	t.statistic	coefficient	coefficient.ci.hig	coefficient.ci.lo	coefficient.s	n
1	chr2	[illegible]	[illegible]	1.10E-11	5.87E-13	[illegible]	[illegible]	-0.029175987	-0.0313072	-0.01800254	-0.03026296	5.87E-13	2.48E-07	4.96E-07	-7.3037141	-0.0313072	-0.022895293	-0.039719149	[illegible]	[illegible]
2	chr7	[illegible]	[illegible]	3.06E-11	3.50E-13	[illegible]	[illegible]	0.052475209	[illegible]	[illegible]	[illegible]	3.50E-13	2.48E-07	2.96E-07	[illegible]	[illegible]	0.073079484	0.042367182	[illegible]	[illegible]
3	chr6	[illegible]	[illegible]	9.88E-12	2.65E-12	2.04E-08	[illegible]	-0.042557845	-0.0444179	-0.03969808	-0.03886756	2.65E-12	7.49E-07	2.25E-06	-7.086674	-0.0444179	-0.03211775	-0.056718046	[illegible]	[illegible]
4	chr1	[illegible]	[illegible]	5.06E-07	5.02E-12	5.36E-06	[illegible]	-0.046715266	-0.0601451	-0.04682984	-0.05855925	5.02E-12	1.06E-06	4.24E-06	-6.9934176	-0.0601451	-0.043267702	-0.077022507	[illegible]	[illegible]
5	chr17	[illegible]	[illegible]	6.19E-10	2.97E-11	8.10E-06	[illegible]	-0.028057216	-0.0298239	-0.02169341	-0.02747897	2.97E-11	5.03E-06	2.52E-05	-6.7267537	-0.0298239	-0.021123234	-0.038524634	[illegible]	[illegible]
6	chr1	[illegible]	[illegible]	8.93E-10	5.65E-11	[illegible]	[illegible]	0.051155219	[illegible]	[illegible]	[illegible]	5.65E-11	6.90E-06	4.78E-05	[illegible]	[illegible]	0.07296508	0.039628877	[illegible]	[illegible]
7	chr10	1440572	[illegible]	2.63E-11	6.84E-11	3.31E-05	[illegible]	-0.036197926	-0.0361565	-0.02763031	-0.03284314	6.84E-11	6.90E-06	5.79E-05	-6.5986839	-0.0361565	-0.025403656	-0.04690938	[illegible]	[illegible]
8	chr10	[illegible]	[illegible]	2.75E-11	8.08E-11	5.60E-06	[illegible]	-0.040669485	-0.0404958	-0.03241493	-0.0368794	8.08E-11	6.90E-06	6.84E-05	-6.5727813	-0.0404958	-0.028405001	-0.052586646	[illegible]	[illegible]
9	chr11	[illegible]	[illegible]	6.48E-10	8.15E-11	[illegible]	[illegible]	-0.013234171	-0.0140638	-0.01019100	-0.01154666	8.15E-11	6.90E-06	6.90E-05	-6.5714236	-0.0140639	-0.009863974	-0.018263811	[illegible]	[illegible]
10	chr12	[illegible]	[illegible]	2.25E-05	7.34E-11	2.20E-05	[illegible]	-0.018037758	-0.0250170	-0.01965099	-0.02401766	7.34E-11	6.90E-06	6.21E-05	-6.5878431	-0.025017	-0.017564789	-0.03246929	[illegible]	[illegible]
11	chr13	[illegible]	[illegible]	2.17E-08	1.01E-10	[illegible]	[illegible]	-0.038830472	-0.0448153	-0.01406621	-0.04080121	1.01E-10	7.76E-06	8.53E-05	-6.5382971	-0.0448154	-0.03136429	-0.058266447	[illegible]	[illegible]
12	chr16	[illegible]	[illegible]	1.91E-10	1.34E-10	1.01E-08	[illegible]	-0.036852796	-0.0372400	-0.03962496	-0.04039767	1.34E-10	9.46E-06	[illegible]	-6.4936068	-0.03724	-0.025985735	-0.048494364	[illegible]	[illegible]
13	chr16	[illegible]	[illegible]	3.32E-09	1.61E-10	6.67E-08	[illegible]	-0.030231928	-0.0334818	-0.03378013	-0.03434845	1.61E-10	1.05E-05	[illegible]	-6.4645789	-0.0334818	-0.023317848	-0.043645801	[illegible]	[illegible]
14	chr1	[illegible]	[illegible]	4.85E-11	2.24E-10	[illegible]	[illegible]	0.039869925	[illegible]	[illegible]	[illegible]	2.24E-10	1.35E-05	[illegible]	[illegible]	[illegible]	0.0514428	0.027334612	[illegible]	[illegible]
15	chr10	[illegible]	[illegible]	1.44E-08	2.59E-10	1.98E-05	[illegible]	-0.027930142	-0.0316328	-0.02367167	-0.0318667	2.59E-10	1.37E-05	[illegible]	-6.3894857	-0.0316328	-0.021917288	-0.041348365	[illegible]	[illegible]
16	chr16	1309550	[illegible]	9.94E-09	2.48E-10	[illegible]	[illegible]	-0.026323335	-0.0292019	-0.0159749	-0.02851799	2.48E-10	1.37E-05	[illegible]	-6.3961193	-0.0292019	-0.020242283	-0.03816151	[illegible]	[illegible]
17	chr3	[illegible]	[illegible]	5.74E-10	2.95E-10	[illegible]	[illegible]	-0.036133684	-0.0369232	-0.02368765	-0.03610142	2.95E-10	1.38E-05	[illegible]	-6.3688938	-0.0369232	-0.025546154	-0.048300291	[illegible]	[illegible]
18	chr8	[illegible]	[illegible]	4.20E-08	2.86E-10	[illegible]	[illegible]	-0.027566647	-0.0319258	-0.01096138	-0.02669888	2.86E-10	1.38E-05	[illegible]	-6.3738303	-0.0319259	-0.022096236	-0.041755483	0.0050089	[illegible]
19	chr15	[illegible]	[illegible]	6.55E-08	3.23E-10	0.0006524	[illegible]	0.050719143	0.0601610	0.04106464	0.05790138	3.23E-10	1.44E-05	[illegible]	-6.354097	-0.060161	-0.041580592	-0.078741501	[illegible]	[illegible]
20	chr6	[illegible]	[illegible]	3.20E-09	3.66E-10	3.72E-07	[illegible]	0.042601797	[illegible]	[illegible]	[illegible]	3.66E-10	1.47E-05	[illegible]	6.3340662	[illegible]	0.06015787	0.03169869	[illegible]	[illegible]

ROSMAP

AD vs CN	chromosom	position	X	p.value.non	p.value.all	p.value.isva	p.value.sva	coefficient.non	coefficient.al	coefficient.isv	coefficient.sv	p.value	fdr	p.holm	t.statistic	coefficient	coefficient.ci.hig	coefficient.ci.lo	coefficient.s	n
1	chr3	[illegible]	[illegible]	1.60E-16	9.42E-13	4.80E-12	8.80E-14	0.030052175	[illegible]	[illegible]	[illegible]	#####	4.45E-07	4.45E-07	[illegible]	0.0291312	0.036777909	0.021484498	[illegible]	[illegible]
2	chr17	[illegible]	[illegible]	2.50E-11	4.65E-09	3.99E-08	3.40E-08	-0.027268491	-0.0277778	-0.02584997	-0.02304457	#####	[illegible]	[illegible]	-6.0585939	-0.0277778	-0.018750774	-0.036804902	[illegible]	[illegible]
3	chr10	[illegible]	[illegible]	5.11E-19	9.53E-09	1.36E-08	2.36E-08	-0.012480759	-0.0083593	-0.00897023	-0.0083175	#####	[illegible]	[illegible]	-5.9263046	-0.0083593	-0.005582122	-0.01113652	[illegible]	[illegible]
4	chr7	[illegible]	[illegible]	5.87E-12	2.19E-08	1.20E-07	7.69E-07	-0.026733655	-0.0251471	-0.02144526	-0.01855586	#####	[illegible]	0.0103625	-5.7699042	-0.0251471	-0.016566094	-0.033728168	[illegible]	[illegible]
5	chr10	[illegible]	[illegible]	8.59E-07	4.36E-08	2.66E-06	2.91E-06	0.018348644	[illegible]	[illegible]	0.01864637	#####	[illegible]	[illegible]	[illegible]	[illegible]	0.031201344	0.015050346	[illegible]	[illegible]
6	chr10	[illegible]	[illegible]	2.21E-12	6.39E-08	1.15E-08	1.15E-07	-0.029270134	-0.0259127	-0.02525516	-0.02063029	#####	[illegible]	[illegible]	-5.564438	-0.0259127	-0.016743945	-0.035081515	[illegible]	[illegible]
7	chr11	7037872	[illegible]	3.80E-08	6.04E-08	1.16E-07	8.77E-07	-0.024219114	-0.0263912	-0.02266247	-0.01795902	#####	[illegible]	[illegible]	-5.5753123	-0.0263912	-0.017071343	-0.035711098	[illegible]	[illegible]
8	chr17	[illegible]	[illegible]	2.31E-09	7.82E-08	1.95E-07	9.79E-07	-0.017663285	-0.0180373	-0.01632748	-0.01402844	#####	[illegible]	[illegible]	-5.5249312	-0.0180374	-0.011609511	-0.024465226	[illegible]	[illegible]
9	chr4	[illegible]	[illegible]	6.13E-12	1.13E-07	6.86E-06	2.67E-06	-0.023813004	-0.0210080	-0.01665577	-0.0158783	#####	[illegible]	[illegible]	-5.4525083	-0.021008	-0.01342211	-0.028593987	[illegible]	[illegible]
10	chr5	[illegible]	[illegible]	1.20E-08	1.05E-07	1.63E-06	2.59E-05	-0.022997202	-0.0219653	-0.01815711	-0.01410994	#####	[illegible]	[illegible]	-5.4661795	-0.0219653	-0.014053565	-0.029877117	[illegible]	[illegible]
11	chr3	1.5E+08	[illegible]	1.77E-09	1.60E-07	1.85E-06	6.31E-06	-0.023980117	-0.0243014	-0.01651181	-0.01389916	#####	[illegible]	[illegible]	-5.3835121	-0.0243014	-0.015413797	-0.03318906	[illegible]	[illegible]
12	chr8	[illegible]	[illegible]	1.55E-16	1.43E-07	3.20E-07	4.13E-07	-0.046931906	-0.0310629	-0.03230399	-0.02988762	#####	[illegible]	[illegible]	-5.4055374	-0.031063	-0.019748757	-0.042377157	[illegible]	[illegible]
13	chr6	[illegible]	[illegible]	1.28E-09	1.72E-07	7.60E-06	5.66E-06	-0.029079588	-0.0282057	-0.02335401	-0.01953018	#####	[illegible]	[illegible]	-5.3693017	-0.0282058	-0.017862921	-0.038548613	[illegible]	[illegible]
14	chr13	[illegible]	[illegible]	3.13E-14	2.21E-07	1.31E-08	3.21E-08	-0.036442973	-0.0276467	-0.02521984	-0.02172235	#####	[illegible]	[illegible]	-5.3190005	-0.0276467	-0.017412999	-0.037880437	[illegible]	[illegible]
15	chr3	1.7E+08	[illegible]	2.19E-05	2.08E-07	1.63E-06	2.14E-09	0.009172039	[illegible]	[illegible]	[illegible]	#####	[illegible]	[illegible]	[illegible]	[illegible]	0.017025167	0.007840853	[illegible]	[illegible]
16	chr8	[illegible]	[illegible]	7.17E-09	2.03E-07	1.01E-05	1.15E-05	-0.007828791	-0.0075067	-0.00662559	-0.00645521	#####	[illegible]	0.0959178	-5.3360454	-0.0075067	-0.00473692	-0.010276564	0.0014068	[illegible]
17	chr13	[illegible]	[illegible]	1.32E-11	3.39E-07	1.90E-07	1.05E-07	-0.046393091	-0.0400600	-0.03441364	-0.03125059	#####	[illegible]	[illegible]	-5.2324278	-0.04006	-0.024986056	-0.055134025	[illegible]	[illegible]
18	chr11	[illegible]	[illegible]	1.41E-09	3.84E-07	9.99E-06	1.91E-05	-0.016358415	-0.0151563	-0.01357701	-0.01281773	#####	[illegible]	[illegible]	-5.206595	-0.0151564	-0.009424956	-0.020887764	[illegible]	[illegible]
19	chr8	[illegible]	[illegible]	1.84E-17	4.00E-07	1.65E-06	9.60E-07	-0.032608743	-0.0201178	-0.02019198	-0.0188758	#####	[illegible]	[illegible]	5.1983603	-0.0201179	-0.012498209	-0.027737526	[illegible]	[illegible]
20	chr14	[illegible]	[illegible]	1.76E-11	4.65E-07	4.60E-07	7.94E-07	-0.026386286	-0.0207823	-0.01946064	-0.01579278	#####	[illegible]	[illegible]	-5.1673177	-0.0207823	-0.012863709	-0.028700917	[illegible]	[illegible]

AD: Alzheimer's disease; MCI: Mild Cognitive Impairment; CN: Cognitively Normal
