# Supplementary material for: Genetic and pathway complexity in Alzheimer’s disease: Insights from multi-omic data about the immune response and mitochondrial function
Source: Neural Regen Res. 2025 Sep 29;21(9):4406–23. doi: 10.4103/NRR.NRR-D-25-00184 (PMC13557549; doi:10.4103/NRR.NRR-D-25-00184)
Supplement: Supplementary file 3 [file NRR-21-4406_Suppl11.pdf]

Additional Table 11 Epigenome-wide association analysis results of important characterization genes corresponding to CpGs

	Gene	gene_cou	variables	adni_adco	adni_adco_f	adni_admci	adni_admci_f	adni_mcico	adni_mcico_f	rosh_adco	rosh_adco_f	rosh_admci	rosh_admci_f	adni_adco_c	adni_admci_c	adni_mcico_c	rosh_adco_c	rosh_admci_c	rosh_mcico_c	fdr_significant	fdr_count
1	HLA-DRA	10	SCHERER_PBMC_APSV_WETVAX_AGE_18_40YO_5_TO_7DY_UP/GOMF_MHC_CLASS_II_PROTEIN_COMPLEX_BINDING/GOBP_ANTIGEN_PROCESSING_AND_PRESENTATION_OF_EXOGENOUS_ANTIGEN/GOBP_ANTIGEN_PROCESSING_AND_PRESENTATION_OF_EXOGENOUS_PEPTIDE_ANTIGEN/GOBP_PEPTIDE_ANTIGEN_ASSEMBLY_[illegible]	[illegible]	[illegible]	[illegible]	[illegible]	[illegible]	[illegible]	NA	NA	[illegible]	[illegible]	[illegible]	[illegible]	[illegible]	NA	[illegible]	NA	adni_adco_fdr, rosh_admci_fdr	2
2	COL1A1	9	HAY_BONE_MARROW_STROMAL/CSR_EARLY_UP.V1_DN/HP_ARTERIAL_DISSECTION/HP_LACK_OF_SKIN_ELASTICITY/HP_STRIAE_DISTENSAE/GNF2_CDH11/MIR92A_1_UP/CERVERA_SDHB_TARGETS_1_UP/REACTOME_ASSEMBLY_OF_COLLAGEN_FIBRILS_AND_OTHER_MULTIMERIC_STRUCTURES	NA	NA	[illegible]	[illegible]	[illegible]	[illegible]	NA	NA	NA	NA	NA	[illegible]	[illegible]	NA	NA	NA	adni_admci_fdr	1
3	GAD1	8	E2F3_UP.V1_UP/BCAT.100_UP.V1_UP/GOBP_EXPLORATION_BEHAVIOR/GOBP_LOCOMOTORY_EXPLORATION_BEHAVIOR/GOCC_PRESYNAPTIC_ACTIVE_ZONE/GNF2_AF1Q/GNF2_RTN1/MODULE_274	[illegible]	[illegible]	NA	NA	NA	NA	NA	NA	NA	NA	[illegible]	NA	NA	NA	NA	NA	adni_adco_fdr	1
4	SIK1	8	[illegible]	NA	NA	[illegible]	[illegible]	[illegible]	[illegible]	NA	NA	NA	NA	NA	[illegible]	[illegible]	NA	NA	NA	adni_admci_fdr	1
