# Supplementary material for: Genetic and pathway complexity in Alzheimer’s disease: Insights from multi-omic data about the immune response and mitochondrial function
Source: Neural Regen Res. 2025 Sep 29;21(9):4406–23. doi: 10.4103/NRR.NRR-D-25-00184 (PMC13557549; doi:10.4103/NRR.NRR-D-25-00184)
Supplement: Supplementary file 4 [file NRR-21-4406_Suppl12.pdf]

**Additional Table 12 Enrichment results of chromatin state for important CpGs loci**

| ADNI | | | | | | | | | | | | | | | |
|---|---|---|---|---|---|---|---|---|---|---|---|---|---|---|---|
| cell | Active TSS | Flanking Active TSS | Transcr. at gene 5' and 3' | Strong transcription | Weak transcription | Genic enhancers | Enhancers | ZNF genes & repeats | Heterochromatin | Bivalent/Poised TSS | Flanking Bivalent TSS/Enh | Bivalent Enhancer | Repressed PolyComb | Weak Repressed PolyComb | Quiescent/Low |
| ES-I3 Cells | [illegible] | [illegible] | [illegible] | [illegible] | [illegible] | [illegible] | [illegible] | [illegible] | [illegible] | [illegible] | [illegible] | [illegible] | [illegible] | [illegible] | [illegible] |
| ES-WA7 Cells | [illegible] | [illegible] | [illegible] | [illegible] | [illegible] | [illegible] | [illegible] | [illegible] | [illegible] | [illegible] | [illegible] | [illegible] | [illegible] | [illegible] | [illegible] |
| H1 Cells | [illegible] | [illegible] | [illegible] | [illegible] | [illegible] | [illegible] | [illegible] | [illegible] | [illegible] | [illegible] | [illegible] | [illegible] | [illegible] | [illegible] | [illegible] |
| H1 BMP4 Derived Mesendoderm Cultured Cells | [illegible] | [illegible] | [illegible] | [illegible] | [illegible] | [illegible] | [illegible] | [illegible] | [illegible] | [illegible] | [illegible] | [illegible] | [illegible] | [illegible] | [illegible] |
| H1 BMP4 Derived Trophoblast Cultured Cells | [illegible] | [illegible] | [illegible] | [illegible] | [illegible] | [illegible] | [illegible] | [illegible] | [illegible] | [illegible] | [illegible] | [illegible] | [illegible] | [illegible] | [illegible] |
| H1 Derived Mesenchymal Stem Cells | [illegible] | [illegible] | [illegible] | [illegible] | [illegible] | [illegible] | [illegible] | [illegible] | [illegible] | [illegible] | [illegible] | [illegible] | [illegible] | [illegible] | [illegible] |
| H1 Derived Neuronal Progenitor Cultured Cells | [illegible] | [illegible] | [illegible] | [illegible] | [illegible] | [illegible] | [illegible] | [illegible] | [illegible] | [illegible] | [illegible] | [illegible] | [illegible] | [illegible] | [illegible] |
| H9 Cells | [illegible] | [illegible] | [illegible] | [illegible] | [illegible] | [illegible] | [illegible] | [illegible] | [illegible] | [illegible] | [illegible] | [illegible] | [illegible] | [illegible] | [illegible] |
| H9 Derived Neuronal Progenitor Cultured Cells | [illegible] | [illegible] | [illegible] | [illegible] | [illegible] | [illegible] | [illegible] | [illegible] | [illegible] | [illegible] | [illegible] | [illegible] | [illegible] | [illegible] | [illegible] |
| H9 Derived Neuron Cultured Cells | [illegible] | [illegible] | [illegible] | [illegible] | [illegible] | [illegible] | [illegible] | [illegible] | [illegible] | [illegible] | [illegible] | [illegible] | [illegible] | [illegible] | [illegible] |
| hESC Derived CD184+ Endoderm Cultured Cells | [illegible] | [illegible] | [illegible] | [illegible] | [illegible] | [illegible] | [illegible] | [illegible] | [illegible] | [illegible] | [illegible] | [illegible] | [illegible] | [illegible] | [illegible] |
| hESC Derived CD56+ Ectoderm Cultured Cells | [illegible] | [illegible] | [illegible] | [illegible] | [illegible] | [illegible] | [illegible] | [illegible] | [illegible] | [illegible] | [illegible] | [illegible] | [illegible] | [illegible] | [illegible] |
| hESC Derived CD56+ Mesoderm Cultured Cells | [illegible] | [illegible] | [illegible] | [illegible] | [illegible] | [illegible] | [illegible] | [illegible] | [illegible] | [illegible] | [illegible] | [illegible] | [illegible] | [illegible] | [illegible] |
| HUES48 Cells | [illegible] | [illegible] | [illegible] | [illegible] | [illegible] | [illegible] | [illegible] | [illegible] | [illegible] | [illegible] | [illegible] | [illegible] | [illegible] | [illegible] | [illegible] |
| HUES6 Cells | [illegible] | [illegible] | [illegible] | [illegible] | [illegible] | [illegible] | [illegible] | [illegible] | [illegible] | [illegible] | [illegible] | [illegible] | [illegible] | [illegible] | [illegible] |
| HUES64 Cells | [illegible] | [illegible] | [illegible] | [illegible] | [illegible] | [illegible] | [illegible] | [illegible] | [illegible] | [illegible] | [illegible] | [illegible] | [illegible] | [illegible] | [illegible] |
| IMR90 fetal lung fibroblasts Cell Line | [illegible] | [illegible] | [illegible] | [illegible] | [illegible] | [illegible] | [illegible] | [illegible] | [illegible] | [illegible] | [illegible] | [illegible] | [illegible] | [illegible] | [illegible] |
| iPS-15b Cells | [illegible] | [illegible] | [illegible] | [illegible] | [illegible] | [illegible] | [illegible] | [illegible] | [illegible] | [illegible] | [illegible] | [illegible] | [illegible] | [illegible] | [illegible] |
| iPS-18 Cells | [illegible] | [illegible] | [illegible] | [illegible] | [illegible] | [illegible] | [illegible] | [illegible] | [illegible] | [illegible] | [illegible] | [illegible] | [illegible] | [illegible] | [illegible] |
| iPS-20b Cells | [illegible] | [illegible] | [illegible] | [illegible] | [illegible] | [illegible] | [illegible] | [illegible] | [illegible] | [illegible] | [illegible] | [illegible] | [illegible] | [illegible] | [illegible] |
| iPS DF 6.9 Cells | [illegible] | [illegible] | [illegible] | [illegible] | [illegible] | [illegible] | [illegible] | [illegible] | [illegible] | [illegible] | [illegible] | [illegible] | [illegible] | [illegible] | [illegible] |
| iPS DF 19.11 Cells | [illegible] | [illegible] | [illegible] | [illegible] | [illegible] | [illegible] | [illegible] | [illegible] | [illegible] | [illegible] | [illegible] | [illegible] | [illegible] | [illegible] | [illegible] |
| Mesenchymal Stem Cell Derived Adipocyte Cultured Cells | [illegible] | [illegible] | [illegible] | [illegible] | [illegible] | [illegible] | [illegible] | [illegible] | [illegible] | [illegible] | [illegible] | [illegible] | [illegible] | [illegible] | [illegible] |
| ES-UCSF4 Cells | [illegible] | [illegible] | [illegible] | [illegible] | [illegible] | [illegible] | [illegible] | [illegible] | [illegible] | [illegible] | [illegible] | [illegible] | [illegible] | [illegible] | [illegible] |
| Adipose Derived Mesenchymal Stem Cell Cultured Cells | [illegible] | [illegible] | [illegible] | [illegible] | [illegible] | [illegible] | [illegible] | [illegible] | [illegible] | [illegible] | [illegible] | [illegible] | [illegible] | [illegible] | [illegible] |
| Bone Marrow Derived Cultured Mesenchymal Stem Cells | [illegible] | [illegible] | [illegible] | [illegible] | [illegible] | [illegible] | [illegible] | [illegible] | [illegible] | [illegible] | [illegible] | [illegible] | [illegible] | [illegible] | [illegible] |
| Breast Myoepithelial Primary Cells | [illegible] | [illegible] | [illegible] | [illegible] | [illegible] | [illegible] | [illegible] | [illegible] | [illegible] | [illegible] | [illegible] | [illegible] | [illegible] | [illegible] | [illegible] |
| Breast variant Human Mammary Epithelial Cells (vHMEC) | [illegible] | [illegible] | [illegible] | [illegible] | [illegible] | [illegible] | [illegible] | [illegible] | [illegible] | [illegible] | [illegible] | [illegible] | [illegible] | [illegible] | [illegible] |
| Primary monocytes from peripheral blood | [illegible] | [illegible] | [illegible] | [illegible] | [illegible] | [illegible] | [illegible] | [illegible] | [illegible] | [illegible] | [illegible] | [illegible] | [illegible] | [illegible] | [illegible] |
| Primary neutrophils from peripheral blood | [illegible] | [illegible] | [illegible] | [illegible] | [illegible] | [illegible] | [illegible] | [illegible] | [illegible] | [illegible] | [illegible] | [illegible] | [illegible] | [illegible] | [illegible] |
| Primary B cells from cord blood | [illegible] | [illegible] | [illegible] | [illegible] | [illegible] | [illegible] | [illegible] | [illegible] | [illegible] | [illegible] | [illegible] | [illegible] | [illegible] | [illegible] | [illegible] |
| Primary B cells from peripheral blood | [illegible] | [illegible] | [illegible] | [illegible] | [illegible] | [illegible] | [illegible] | [illegible] | [illegible] | [illegible] | [illegible] | [illegible] | [illegible] | [illegible] | [illegible] |
| Primary T cells from cord blood | [illegible] | [illegible] | [illegible] | [illegible] | [illegible] | [illegible] | [illegible] | [illegible] | [illegible] | [illegible] | [illegible] | [illegible] | [illegible] | [illegible] | [illegible] |
| Primary T cells from peripheral blood | [illegible] | [illegible] | [illegible] | [illegible] | [illegible] | [illegible] | [illegible] | [illegible] | [illegible] | [illegible] | [illegible] | [illegible] | [illegible] | [illegible] | [illegible] |
| Primary hematopoietic stem cells | [illegible] | [illegible] | [illegible] | [illegible] | [illegible] | [illegible] | [illegible] | [illegible] | [illegible] | [illegible] | [illegible] | [illegible] | [illegible] | [illegible] | [illegible] |
| Primary hematopoietic stem cells short term culture | [illegible] | [illegible] | [illegible] | [illegible] | [illegible] | [illegible] | [illegible] | [illegible] | [illegible] | [illegible] | [illegible] | [illegible] | [illegible] | [illegible] | [illegible] |
| Primary T helper memory cells from peripheral blood 2 | [illegible] | [illegible] | [illegible] | [illegible] | [illegible] | [illegible] | [illegible] | [illegible] | [illegible] | [illegible] | [illegible] | [illegible] | [illegible] | [illegible] | [illegible] |
| Primary T helper naive cells from peripheral blood 1 | [illegible] | [illegible] | [illegible] | [illegible] | [illegible] | [illegible] | [illegible] | [illegible] | [illegible] | [illegible] | [illegible] | [illegible] | [illegible] | [illegible] | [illegible] |
| Primary T helper naive cells from peripheral blood 2 | [illegible] | [illegible] | [illegible] | [illegible] | [illegible] | [illegible] | [illegible] | [illegible] | [illegible] | [illegible] | [illegible] | [illegible] | [illegible] | [illegible] | [illegible] |
| Primary T helper memory cells from peripheral blood 1 | [illegible] | [illegible] | [illegible] | [illegible] | [illegible] | [illegible] | [illegible] | [illegible] | [illegible] | [illegible] | [illegible] | [illegible] | [illegible] | [illegible] | [illegible] |
| Primary T helper cells PMA-I stimulated | [illegible] | [illegible] | [illegible] | [illegible] | [illegible] | [illegible] | [illegible] | [illegible] | [illegible] | [illegible] | [illegible] | [illegible] | [illegible] | [illegible] | [illegible] |
| Primary T helper 17 cells PMA-I stimulated | [illegible] | [illegible] | [illegible] | [illegible] | [illegible] | [illegible] | [illegible] | [illegible] | [illegible] | [illegible] | [illegible] | [illegible] | [illegible] | [illegible] | [illegible] |
| Primary T helper cells from peripheral blood | [illegible] | [illegible] | [illegible] | [illegible] | [illegible] | [illegible] | [illegible] | [illegible] | [illegible] | [illegible] | [illegible] | [illegible] | [illegible] | [illegible] | [illegible] |
| Primary T regulatory cells from peripheral blood | [illegible] | [illegible] | [illegible] | [illegible] | [illegible] | [illegible] | [illegible] | [illegible] | [illegible] | [illegible] | [illegible] | [illegible] | [illegible] | [illegible] | [illegible] |
| Primary T cells effector/memory enriched from peripheral blood | [illegible] | [illegible] | [illegible] | [illegible] | [illegible] | [illegible] | [illegible] | [illegible] | [illegible] | [illegible] | [illegible] | [illegible] | [illegible] | [illegible] | [illegible] |
| Primary Natural Killer cells from peripheral blood | [illegible] | [illegible] | [illegible] | [illegible] | [illegible] | [illegible] | [illegible] | [illegible] | [illegible] | [illegible] | [illegible] | [illegible] | [illegible] | [illegible] | [illegible] |
| Primary T CD8+ naive cells from peripheral blood | [illegible] | [illegible] | [illegible] | [illegible] | [illegible] | [illegible] | [illegible] | [illegible] | [illegible] | [illegible] | [illegible] | [illegible] | [illegible] | [illegible] | [illegible] |
| Primary T CD8+ memory cells from peripheral blood | [illegible] | [illegible] | [illegible] | [illegible] | [illegible] | [illegible] | [illegible] | [illegible] | [illegible] | [illegible] | [illegible] | [illegible] | [illegible] | [illegible] | [illegible] |
| Mesenchymal Stem Cell Derived Chondrocyte Cultured Cells | [illegible] | [illegible] | [illegible] | [illegible] | [illegible] | [illegible] | [illegible] | [illegible] | [illegible] | [illegible] | [illegible] | [illegible] | [illegible] | [illegible] | [illegible] |
| Primary hematopoietic stem cells G-CSF-mobilized Female | [illegible] | [illegible] | [illegible] | [illegible] | [illegible] | [illegible] | [illegible] | [illegible] | [illegible] | [illegible] | [illegible] | [illegible] | [illegible] | [illegible] | [illegible] |
| Primary hematopoietic stem cells G-CSF-mobilized Male | [illegible] | [illegible] | [illegible] | [illegible] | [illegible] | [illegible] | [illegible] | [illegible] | [illegible] | [illegible] | [illegible] | [illegible] | [illegible] | [illegible] | [illegible] |
| Muscle Satellite Cultured Cells | [illegible] | [illegible] | [illegible] | [illegible] | [illegible] | [illegible] | [illegible] | [illegible] | [illegible] | [illegible] | [illegible] | [illegible] | [illegible] | [illegible] | [illegible] |
| Cortex derived primary cultured neurospheres | [illegible] | [illegible] | [illegible] | [illegible] | [illegible] | [illegible] | [illegible] | [illegible] | [illegible] | [illegible] | [illegible] | [illegible] | [illegible] | [illegible] | [illegible] |
| Ganglion Eminence derived primary cultured neurospheres | [illegible] | [illegible] | [illegible] | [illegible] | [illegible] | [illegible] | [illegible] | [illegible] | [illegible] | [illegible] | [illegible] | [illegible] | [illegible] | [illegible] | [illegible] |
| Foreskin Fibroblast Primary Cells skin01 | [illegible] | [illegible] | [illegible] | [illegible] | [illegible] | [illegible] | [illegible] | [illegible] | [illegible] | [illegible] | [illegible] | [illegible] | [illegible] | [illegible] | [illegible] |
| Foreskin Fibroblast Primary Cells skin02 | [illegible] | [illegible] | [illegible] | [illegible] | [illegible] | [illegible] | [illegible] | [illegible] | [illegible] | [illegible] | [illegible] | [illegible] | [illegible] | [illegible] | [illegible] |
| Foreskin Keratinocyte Primary Cells skin02 | [illegible] | [illegible] | [illegible] | [illegible] | [illegible] | [illegible] | [illegible] | [illegible] | [illegible] | [illegible] | [illegible] | [illegible] | [illegible] | [illegible] | [illegible] |
| Foreskin Keratinocyte Primary Cells skin03 | [illegible] | [illegible] | [illegible] | [illegible] | [illegible] | [illegible] | [illegible] | [illegible] | [illegible] | [illegible] | [illegible] | [illegible] | [illegible] | [illegible] | [illegible] |
| Foreskin Melanocyte Primary Cells skin01 | [illegible] | [illegible] | [illegible] | [illegible] | [illegible] | [illegible] | [illegible] | [illegible] | [illegible] | [illegible] | [illegible] | [illegible] | [illegible] | [illegible] | [illegible] |
| Foreskin Melanocyte Primary Cells skin03 | [illegible] | [illegible] | [illegible] | [illegible] | [illegible] | [illegible] | [illegible] | [illegible] | [illegible] | [illegible] | [illegible] | [illegible] | [illegible] | [illegible] | [illegible] |
| Primary mononuclear cells from peripheral blood | [illegible] | [illegible] | [illegible] | [illegible] | [illegible] | [illegible] | [illegible] | [illegible] | [illegible] | [illegible] | [illegible] | [illegible] | [illegible] | [illegible] | [illegible] |
| Adipose Nuclei | [illegible] | [illegible] | [illegible] | [illegible] | [illegible] | [illegible] | [illegible] | [illegible] | [illegible] | [illegible] | [illegible] | [illegible] | [illegible] | [illegible] | [illegible] |
| Aorta | [illegible] | [illegible] | [illegible] | [illegible] | [illegible] | [illegible] | [illegible] | [illegible] | [illegible] | [illegible] | [illegible] | [illegible] | [illegible] | [illegible] | [illegible] |
| Liver | [illegible] | [illegible] | [illegible] | [illegible] | [illegible] | [illegible] | [illegible] | [illegible] | [illegible] | [illegible] | [illegible] | [illegible] | [illegible] | [illegible] | [illegible] |
| Brain Angular Gyrus | [illegible] | [illegible] | [illegible] | [illegible] | [illegible] | [illegible] | [illegible] | [illegible] | [illegible] | [illegible] | [illegible] | [illegible] | [illegible] | [illegible] | [illegible] |
| Brain Anterior Caudate | [illegible] | [illegible] | [illegible] | [illegible] | [illegible] | [illegible] | [illegible] | [illegible] | [illegible] | [illegible] | [illegible] | [illegible] | [illegible] | [illegible] | [illegible] |
| Brain Cingulate Gyrus | [illegible] | [illegible] | [illegible] | [illegible] | [illegible] | [illegible] | [illegible] | [illegible] | [illegible] | [illegible] | [illegible] | [illegible] | [illegible] | [illegible] | [illegible] |
| Brain Germinal Matrix | [illegible] | [illegible] | [illegible] | [illegible] | [illegible] | [illegible] | [illegible] | [illegible] | [illegible] | [illegible] | [illegible] | [illegible] | [illegible] | [illegible] | [illegible] |
| Brain Hippocampus Middle | [illegible] | [illegible] | [illegible] | [illegible] | [illegible] | [illegible] | [illegible] | [illegible] | [illegible] | [illegible] | [illegible] | [illegible] | [illegible] | [illegible] | [illegible] |
| Brain Inferior Temporal Lobe | [illegible] | [illegible] | [illegible] | [illegible] | [illegible] | [illegible] | [illegible] | [illegible] | [illegible] | [illegible] | [illegible] | [illegible] | [illegible] | [illegible] | [illegible] |
| Brain_Dorsolateral_Prefrontal_Cortex | [illegible] | [illegible] | [illegible] | [illegible] | [illegible] | [illegible] | [illegible] | [illegible] | [illegible] | [illegible] | [illegible] | [illegible] | [illegible] | [illegible] | [illegible] |
| Brain Substantia Nigra | [illegible] | [illegible] | [illegible] | [illegible] | [illegible] | [illegible] | [illegible] | [illegible] | [illegible] | [illegible] | [illegible] | [illegible] | [illegible] | [illegible] | [illegible] |
| Colonic Mucosa | [illegible] | [illegible] | [illegible] | [illegible] | [illegible] | [illegible] | [illegible] | [illegible] | [illegible] | [illegible] | [illegible] | [illegible] | [illegible] | [illegible] | [illegible] |
| Colon Smooth Muscle | [illegible] | [illegible] | [illegible] | [illegible] | [illegible] | [illegible] | [illegible] | [illegible] | [illegible] | [illegible] | [illegible] | [illegible] | [illegible] | [illegible] | [illegible] |
| Duodenum Mucosa | [illegible] | [illegible] | [illegible] | [illegible] | [illegible] | [illegible] | [illegible] | [illegible] | [illegible] | [illegible] | [illegible] | [illegible] | [illegible] | [illegible] | [illegible] |
| Duodenum Smooth Muscle | [illegible] | [illegible] | [illegible] | [illegible] | [illegible] | [illegible] | [illegible] | [illegible] | [illegible] | [illegible] | [illegible] | [illegible] | [illegible] | [illegible] | [illegible] |
| Esophagus | [illegible] | [illegible] | [illegible] | [illegible] | [illegible] | [illegible] | [illegible] | [illegible] | [illegible] | [illegible] | [illegible] | [illegible] | [illegible] | [illegible] | [illegible] |
| Fetal Adrenal Gland | [illegible] | [illegible] | [illegible] | [illegible] | [illegible] | [illegible] | [illegible] | [illegible] | [illegible] | [illegible] | [illegible] | [illegible] | [illegible] | [illegible] | [illegible] |
| Fetal Brain Male | [illegible] | [illegible] | [illegible] | [illegible] | [illegible] | [illegible] | [illegible] | [illegible] | [illegible] | [illegible] | [illegible] | [illegible] | [illegible] | [illegible] | [illegible] |
| Fetal Brain Female | [illegible] | [illegible] | [illegible] | [illegible] | [illegible] | [illegible] | [illegible] | [illegible] | [illegible] | [illegible] | [illegible] | [illegible] | [illegible] | [illegible] | [illegible] |
| Fetal Heart | [illegible] | [illegible] | [illegible] | [illegible] | [illegible] | [illegible] | [illegible] | [illegible] | [illegible] | [illegible] | [illegible] | [illegible] | [illegible] | [illegible] | [illegible] |
| Fetal Intestine Large | [illegible] | [illegible] | [illegible] | [illegible] | [illegible] | [illegible] | [illegible] | [illegible] | [illegible] | [illegible] | [illegible] | [illegible] | [illegible] | [illegible] | [illegible] |
| Fetal Intestine Small | [illegible] | [illegible] | [illegible] | [illegible] | [illegible] | [illegible] | [illegible] | [illegible] | [illegible] | [illegible] | [illegible] | [illegible] | [illegible] | [illegible] | [illegible] |
| Fetal Kidney | [illegible] | 3.382718749 | 9.174251852 | 0.335492421 | 0.627831827 | 4.234439981 | [illegible] | 4.666902506 | 3.779932809 | 1.704967692 | 3.939190372 | 8.249394057 | 1.741134744 | 1.718587171 | 0.70670835 |
| Pancreatic Islets | 0.4414795 | 2.556371419 | 13.881853 | 0.697654526 | 0.845089355 | 41.29771115 | 0.7026312 | 3.23857438 | 1.065931477 | 3.794870898 | 65.17630055 | 61.50387821 | 4.216769517 | 1.67236225 | 0.908060397 |
| Fetal Lung | 0.6665734 | 1.989263343 | 3.072348658 | 0.81460143 | 0.620510086 | 1.237735718 | 1.5591256 | 2.741398259 | 1.512018611 | 1.559475553 | 1.00422866 | 2.462677574 | 2.619403316 | 1.33718062 | 0.539900239 |
| Fetal Muscle Trunk | 0.7433663 | 1.012282718 | 3.517080981 | 0.566142937 | 0.968541445 | 0.299950934 | 1.3108801 | 3.513941746 | 1.71929925 | 0.595360235 | 1.349020984 | 2.416842741 | 1.333519811 | 1.40895697 | 0.609582551 |
| Fetal Muscle Leg | 0.6822478 | 1.187344709 | 2.123009981 | 0.453409887 | 0.903949934 | 0.267238993 | 1.7363016 | 2.087295056 | 1.185499752 | 0.516366376 | 1.333260999 | 3.446577018 | 1.865086147 | 0.93231391 | 0.467925988 |
| Placenta | 0.6295406 | 0.58824439 | 5.450933715 | 0.657840865 | 0.926029457 | 0.446439889 | 1.4581732 | 2.250616895 | 0.275903589 | 1.853688779 | 1.042651619 | 3.047953797 | 2.196920459 | 1.9837161 | 0.451541758 |
| Fetal Stomach | 0.604326 | 0.606316804 | 1.766437869 | 0.570013715 | 0.870385791 | 0.351214422 | 2.8475793 | 0.945147797 | 0.953817029 | 1.090544282 | 2.260699919 | 3.159002317 | 1.325927024 | 0.804639145 | 0.52108327 |
| Fetal Thymus | 0.2461291 | 0.485244397 | 2.58928208 | 0.314152151 | 0.711677581 | 3.020955376 | 1.4684254 | 2.297502835 | 0.393978698 | 3.544736303 | 0.356999565 | 2.377809142 | 3.834539027 | 1.418527871 | 0.662366825 |
| Gastric | 0.3147782 | 1.451073611 | 6.906120772 | 0.662439801 | 1.267128887 | 0.605580433 | 1.6360061 | 2.671359419 | 0.721140744 | 167.829235 | 26.83143037 | 23.75077042 | 71.71478283 | 3.003081768 | 1.071047289 |
| Left Ventricle | 0.6893372 | 2.004963266 | 7.391363243 | 0.655133375 | 0.903826639 | 1.293515326 | 2.0949735 | 4.158194733 | 0.925366748 | 1.025907316 | 2.842703584 | 2.201131389 | 2.99915963 | 1.324621956 | 0.452899607 |
| Lung | 0.2809888 | 1.45234461 | 6.301711007 | 0.926507556 | 0.775837695 | 2.240910048 | 2.3700831 | 0.775697436 | 1.08898657 | 5.593522227 | 3.745691518 | 0.717977529 | 3.246914444 | 1.711044866 | 0.840485972 |
| Ovary | 0.5207383 | 1.138540737 | 20.22119467 | 1.033499026 | 0.756324669 | 1.86163212 | 2.8957893 | 3.229240202 | 2.656700258 | 3.111363884 | 3.844690863 | 2.233278464 | 3.097517335 | 0.816609573 | 0.817057221 |
| Pancreas | 0.434293 | 1.04088003 | 6.930354864 | 0.605773292 | 1.061615378 | 0.616648135 | 2.4416514 | 1.696932397 | 2.397435003 | 600.7410747 | 53.67003818 | 88.49253413 | 213.2370418 | 2.00950722 | 0.739730019 |
| Placenta Amnion | 0.4188508 | 1.548326345 | 15.8918951 | 0.801876614 | 0.720226891 | 2.081737442 | 2.0834386 | 16.10969912 | 3.966155384 | 2.907162786 | 1.589403161 | 1.320713659 | 2.992582409 | 1.780612237 | 0.70044054 |
| Psoas Muscle | 0.5405851 | 1.056051465 | 18.00577851 | 0.609921337 | 0.635543672 | 6.409567735 | 1.8917305 | 9.271429558 | 1.661875336 | 0.972388036 | 1.143045699 | 2.479316402 | 2.683908171 | 2.056798504 | 0.910779176 |
| Rectal Mucosa Donor 29 | 0.591835 | 2.201074004 | 2.638100873 | 0.507194811 | 0.940713985 | 1.222936668 | 1.5195012 | 4.480532044 | 1.427792735 | 1.546916788 | 0.898281043 | 5.695933672 | 2.51317939 | 1.512308612 | 0.613369232 |
| Rectal Mucosa Donor 31 | 0.4563565 | 1.271957162 | 4.452365637 | 0.483155333 | 0.894197023 | 1.23204695 | 1.8589939 | 5.523631941 | 1.586492646 | 0.611186813 | 3.059274014 | 6.528111473 | 4.304713177 | 0.979560487 | 0.721995688 |
| Rectal Smooth Muscle | 0.514427 | 2.464999564 | 4.207208686 | 0.697034167 | 0.817695457 | 4.151917632 | 2.4434931 | 5.050267127 | 1.726970718 | 0.388513982 | 2.175661975 | 21.15363581 | 2.498474618 | 1.772524542 | 0.523421771 |
| Right Atrium | 1.1254313 | 1.249337947 | 0.224392321 | 3.935242254 | 1.256425535 | 3.057968214 | 2.2245385 | 4.191487246 | 0.679877869 | 10.75456034 | 33.11193754 | 27.87012498 | 4.437171144 | 1.225673899 | 0.630917799 |
| Right Ventricle | 0.8176648 | 0.585480474 | 4.373802495 | 0.458216567 | 0.838675458 | 1.662437144 | 2.7440637 | 9.293291124 | 1.657638174 | 1.485786456 | 1.227959801 | 2.013637972 | 3.36151554 | 1.448052667 | 0.691849327 |
| Sigmoid Colon | 0.2915861 | 0.822347986 | 10.11917452 | 0.415000017 | 0.956552628 | 1.065491706 | 2.4251627 | 3.778114876 | 0.80855962 | 1.255524638 | 3.15899657 | 1.571368174 | 3.477156219 | 1.834438741 | 0.545956779 |
| Skeletal Muscle Male | 0.3303645 | 1.159063738 | 0.90607054 | 0.402657021 | 1.105095765 | 0.501305682 | 2.302387 | 3.260112855 | 1.128098245 | 1.639634259 | 1.723702797 | 2.277031305 | 1.729408525 | 1.101440053 | 0.569304191 |
| Skeletal Muscle Female | 0.7441103 | 0.965510362 | 2.670449592 | 0.402204005 | 0.945054525 | 0.561354629 | 1.7415597 | 5.420891171 | 1.562330914 | 1.80866728 | 2.418240605 | 2.143873087 | 1.794716525 | 1.065803642 | 0.643719204 |
| Small Intestine | 0.3608144 | 2.16239408 | 57.99589573 | 0.458453971 | 0.682832971 | 10.26422337 | 2.1094524 | 7.920610811 | 2.401910379 | 2.151511234 | 12.16433504 | 2.360466742 | 2.913821011 | 1.49591227 | 0.840768214 |
| Stomach Mucosa | 0.2682231 | 0.70912057 | 16.56841029 | 0.933517703 | 0.440954751 | 2.712010921 | 1.6001163 | 11.2279838 | 4.863921547 | 2.501231633 | 1.404366753 | 1.990766725 | 3.475800588 | 1.568863127 | 0.705707867 |
| Stomach Smooth Muscle | 0.9079214 | 1.478188205 | 1.499797061 | 1.0363486 | 0.623279221 | 0.6232185 | 2.157467 | 4.751356917 | 1.534043388 | 0.495946832 | 2.517587196 | 2.226784474 | 1.18974942 | 1.123475947 | 0.503387227 |
| Thymus | 0.1192945 | 1.204299898 | 2.603048338 | 0.609143078 | 0.5172389 | 2.115994054 | 1.7300548 | 1.691415458 | 1.089120001 | 2.616625647 | 1.511609925 | 1.593951777 | 3.280464945 | 1.874898527 | 0.658079376 |
| Spleen | 0.3751403 | 0.365855936 | 5.176607363 | 1.20485754 | 1.032280284 | 0.311478931 | 2.3856702 | 2.757765015 | 1.14429535 | 7.222064715 | 3.092953259 | 1.558212877 | 4.311461561 | 2.276781441 | 0.585542853 |
| A549 EtOH 0.02pct Lung Carcinoma Cell Line | 0.4086419 | 2.03402163 | 4.58586716 | 0.310098696 | 0.604238781 | 2.52395651 | 1.5869568 | 2.39168896 | 1.184174116 | 0.557829053 | 2.362578569 | 8.720673545 | 3.296093104 | 1.34711753 | 0.780344033 |
| Dnd41 TCell Leukemia Cell Line | 0.0931168 | 0.594501495 | 0.583820908 | 0.453434376 | 0.730561762 | 0.684115815 | 2.1632654 | 2.553826647 | 0.957391603 | 6.681503416 | 0.986453138 | 5.065985325 | 2.538211712 | 2.155765623 | 0.813552841 |
| GM12878 Lymphoblastoid Cells | 0.4215797 | 1.378731164 | 2.739483199 | 0.521348686 | 0.419183496 | 0.601725348 | 1.2488395 | 7.433203175 | 0.470885225 | 0.196757327 | 0.321749822 | 2.777237214 | 2.342955805 | 2.162740033 | 0.811847729 |
| HeLa-S3 Cervical Carcinoma Cell Line | 0.3864213 | 1.904762412 | 6.790960772 | 0.960185846 | 0.894464197 | 0.892267843 | 1.7409167 | 3.990939487 | 0.811815907 | 2.768554922 | 0.745519051 | 3.871003687 | 1.950476749 | 1.781587148 | 0.436552404 |
| HepG2 Hepatocellular Carcinoma Cell Line | 0.6948672 | 1.673668884 | 2.314470375 | 0.155010824 | 1.374120178 | 1.89183737 | 1.9464377 | 3.010990392 | 0.655220031 | 0.561230905 | 3.825961644 | 4.93057243 | 2.653125955 | 1.516911089 | 0.424415648 |
| HMEC Mammary Epithelial Primary Cells | 0.7142719 | 2.567159343 | 6.182798344 | 0.73172136 | 1.258762145 | 1.350556586 | 1.2938233 | 4.537653759 | 1.142373028 | 1.549838295 | 6.790514274 | 3.297113534 | 1.576789804 | 1.018233473 | 0.552282349 |
| HSMM Skeletal Muscle Myoblasts Cells | 0.4284141 | 1.243828669 | 4.264349463 | 1.083275835 | 0.557399202 | 1.131386286 | 1.4853713 | 9.152522554 | 0.643031828 | 1.653422426 | 0.742284701 | 6.00755363 | 3.03251766 | 1.128385827 | 0.604672455 |
| HSMM cell derived Skeletal Muscle Myotubes Cells | 0.2779857 | 1.828502269 | 1.095457554 | 0.316297767 | 0.591189179 | 0.806615048 | 2.1899764 | 1.155977452 | 1.002850565 | 2.907899684 | 1.285194902 | 1.455807271 | 3.41852045 | 2.267196659 | 0.541516557 |
| HUVEC Umbilical Vein Endothelial Primary Cells | 0.3311792 | 1.912032465 | 2.100191798 | 0.648072706 | 0.782360707 | 1.470367359 | 2.2327362 | 9.045767413 | 1.591349406 | 11.24401146 | 18.4567353 | 21.08646544 | 1.388254669 | 1.580025601 | 0.767118682 |
| K562 Leukemia Cells | 0.1664351 | 1.140113867 | 1.185981796 | 0.849909942 | 0.642660167 | 0.701410086 | 0.8750195 | 4.234439981 | 1.665778351 | 5.122654269 | 5.966990717 | 2.900709097 | 1.581352993 | 2.302308629 | 0.946460527 |
| Monocytes-CD14+ RO01746 Primary Cells | 0.2587456 | 1.0333146 | 5.698601391 | 0.695730371 | 0.430226612 | 2.069797659 | 1.0571768 | 1.787830892 | 0.502132586 | 0.970992476 | 0.937419165 | 2.318743867 | 4.640323895 | 0.85392168 | 0.594245863 |
| NH-A Astrocytes Primary Cells | 0.6600455 | 1.349580409 | 3.905881878 | 0.626889135 | 1.00276081 | 1.297629705 | 1.5666965 | 5.122654269 | 0.61329111 | 0.813504741 | 3.763916831 | 3.159299256 | 2.075180544 | 1.526528921 | 0.400367954 |
| NHDF-Ad Adult Dermal Fibroblast Primary Cells | 0.642729 | 2.710953553 | 3.931302043 | 0.905487453 | 0.722814926 | 0.929033899 | 1.1892849 | 6.631098914 | 1.536462698 | 2.039609067 | 3.59660864 | 2.264942375 | 2.383848148 | 1.508332431 | 0.543180232 |
| NHEK-Epidermal Keratinocyte Primary Cells | 0.4184949 | 1.988208318 | 2.606453796 | 0.681064384 | 0.605688003 | 0.614866388 | 2.0869938 | 3.692886093 | 0.76583156 | 0.509083683 | 2.027150057 | 1.26718668 | 2.730211293 | 1.293929326 | 0.443368367 |
| NHLF Lung Fibroblast Primary Cells | 0.7208614 | 2.092956018 | 1.450927381 | 1.055081069 | 0.532129022 | 1.291357115 | 1.6226546 | 5.060018254 | 1.231227053 | 0.731631396 | 2.828495501 | 7.772955691 | 2.011748652 | 1.509651781 | 0.5325316 |
| Osteoblast Primary Cells | 0.491932 | 1.645420853 | 9.070526413 | 0.910649338 | 0.699381527 | 1.557913156 | 1.3255687 | 7.315886765 | 1.340553952 | 0.600403949 | 1.23204695 | 2.24803146 | 2.523333517 | 0.953020899 | 0.651867224 |

| ROSMAP | | | | | | | | | | | | | | | |
|---|---|---|---|---|---|---|---|---|---|---|---|---|---|---|---|
| cell | Active TSS | Flanking Active TSS | Transcr. at gene 5' and 3' | Strong transcription | Weak transcription | Genic enhancers | Enhancers | ZNF genes & repeats | Heterochromatin | Bivalent/Poised TSS | Flanking Bivalent TSS/Enh | Bivalent Enhancer | Repressed PolyComb | Weak Repressed PolyComb | Quiescent/Low |
| ES-I3 Cells | 0.2981817 | 2.224581312 | 16.42280969 | 2.706180498 | 1.06936177 | 13.07743515 | 3.1266794 | 16.00996188 | 4.287335119 | 1.708894607 | 4.739425514 | 2.449351791 | 0.563154684 | 0.796584603 | 0.98795412 |
| ES-WA7 Cells | 0.3071079 | 16.17224449 | 16382.88639 | 22.05402158 | 1.230285415 | 875.7274043 | 2.9106479 | 81.05724249 | 20.81065326 | 1.997193045 | 16.00996188 | 2.756428682 | 1.006771572 | 1.212610472 | 1.043204896 |
| H1 Cells | 0.1052408 | 2.303808265 | 11.8022701 | 1.95991914 | 1.80960529 | 5.946188364 | 1.4210325 | 5.332228293 | 1.263551011 | 1.71903839 | 2.180454397 | 1.079281524 | 1.069682479 | 1.350618135 | 0.778201084 |
| H1 BMP4 Derived Mesendoderm Cultured Cells | 0.2073462 | 6.463119105 | 347.7455372 | 122.6818424 | 0.69117156 | 259.0558737 | 1.4142052 | 47.58572948 | 3.099339278 | 1.518215404 | 20.21716861 | 5.876364443 | 1.810010877 | 1.76449574 | 1.492286555 |
| H1 BMP4 Derived Trophoblast Cultured Cells | 0.1284606 | 2.063642273 | 6.907677862 | 1.646108316 | 1.696854862 | 1.453616965 | 1.3942099 | 1.966011811 | 0.731503754 | 2.624735782 | 1.060631438 | 2.05373684 | 0.757847293 | 0.754740221 | 1.124954192 |
| H1 Derived Mesenchymal Stem Cells | 0.404436 | 0.930818621 | 4.813961027 | 0.832264857 | 0.783366635 | 2.977158024 | 3.0394885 | 4.803176001 | 1.134689151 | 12.48570471 | 1.78239393 | 0.76786449 | 0.753926632 | 0.625207862 | 1.062676571 |
| H1 Derived Neuronal Progenitor Cultured Cells | 0.4079409 | 1.808727141 | 36.52910781 | 0.875631121 | 1.369722456 | 12.34274366 | 3.1939823 | 5.526634979 | 1.724873783 | 1.899930198 | 7.235295663 | 3.199357071 | 1.080421855 | 1.221945603 | 1.068620075 |
| H9 Cells | 0.3143368 | 4.60395778 | 17.50743276 | 1.670452253 | 2.106749325 | 22.24848817 | 3.1105925 | 4.487072914 | 1.230282116 | 1.599956895 | 11.66542887 | 9.04333887 | 1.306596885 | 1.012523333 | 0.557226864 |
| H9 Derived Neuronal Progenitor Cultured Cells | 0.2181314 | 0.677185049 | 4.214798541 | 2.036295857 | 1.444237322 | 3.822271885 | 3.5203455 | 3.47894931 | 2.560301711 | 1.161829174 | 1.994276015 | 1.623893688 | 1.05652795 | 1.455249985 | 0.426357264 |
| H9 Derived Neuron Cultured Cells | 0.1197856 | 0.381179973 | 6.52487402 | 2.633468959 | 1.83400308 | 3.467618893 | 3.955665 | 2.83226355 | 2.037624345 | 2.069149811 | 2.738650159 | 1.431993176 | 1.111047037 | 0.489117742 | 0.474590329 |
| hESC Derived CD184+ Endoderm Cultured Cells | 0.195616 | 1.594098115 | 5.587581633 | 1.494747245 | 2.978833038 | 10.45352908 | 0.5019583 | 4.477632069 | 1.426090186 | 1.679538612 | 2.882861507 | 4.605752228 | 0.989254543 | 0.461963087 | 0.85872257 |
| hESC Derived CD56+ Ectoderm Cultured Cells | 0.1103545 | 2.006792516 | 24.39736115 | 3.967016374 | 0.92521021 | 14.05768784 | 1.6755345 | 19.19432305 | 3.68553923 | 0.693268447 | 6.795205783 | 1.230177225 | 0.548834958 | 0.574379193 | 1.418796834 |
| hESC Derived CD56+ Mesoderm Cultured Cells | 0.2666806 | 7.434725567 | 4.817050921 | 2.597246009 | 0.59618626 | 6.929318096 | 1.8941492 | 5.504343086 | 1.013170221 | 0.73470011 | 5.855734492 | 7.506891674 | 0.738079388 | 0.429699637 | 0.875085002 |
| HUES48 Cells | 0.2005584 | 2.35577104 | 8.872283556 | 1.858718912 | 1.185125935 | 7.772814507 | 1.2952528 | 6.696393833 | 1.304711924 | 2.064338325 | 1.413049868 | 2.223571235 | 0.804536724 | 0.686536896 | 1.037250316 |
| HUES6 Cells | 0.2110513 | 0.844787052 | 4.976240702 | 1.685858095 | 0.881805199 | 8.886873994 | 0.7001901 | 4.578422624 | 1.098905517 | 1.541236424 | 1.779313686 | 2.273341781 | 1.614797383 | 0.568181039 | 1.737119531 |
| HUES64 Cells | 0.1006014 | 1.531791574 | 7.5393268 | 1.901815952 | 1.190427569 | 8.467911872 | 1.8648005 | 6.866845639 | 2.66728767 | 2.344942578 | 1.926331912 | 2.765157585 | 0.712325029 | 0.681813557 | 0.883064542 |
| IMR90 fetal lung fibroblasts Cell Line | 0.2376154 | 1.448807298 | 3.367283131 | 2.226233426 | 0.703095842 | 3.34600173 | 2.4777121 | 5.401332733 | 1.647507711 | 1.375423508 | 1.390982403 | 2.862381202 | 0.235506428 | 0.959348964 | 0.332900486 |
| iPS-15b Cells | 0.1852339 | 1.542168614 | 8.872283556 | 1.86630685 | 0.98543288 | 13.06618446 | 1.3454711 | 16.06331947 | 2.343654978 | 2.076416395 | 1.671073554 | 3.068628376 | 0.928892934 | 0.52642203 | 1.282889635 |
| iPS-18 Cells | 0.2221149 | 1.079932855 | 10.40425292 | 2.312436211 | 0.830956087 | 12.05683326 | 0.844024 | 11.19797062 | 1.748776593 | 1.52494775 | 1.064667637 | 2.060745057 | 0.707653098 | 1.286116243 | 1.138165893 |
| iPS-20b Cells | 0.1028223 | 1.252811263 | 6.188807234 | 1.511153564 | 1.979852631 | 8.388683248 | 2.3485195 | 8.092953624 | 1.414563787 | 2.037721207 | 1.607266174 | 2.86506054 | 0.597993018 | 0.501022785 | 0.876529421 |
| iPS DF 6.9 Cells | 0.2662545 | 2.385206722 | 20.83931794 | 1.466609936 | 1.624165502 | 10.88458403 | 2.0998957 | 21.92624761 | 1.820607361 | 3.039834896 | 3.062937034 | 2.446530789 | 1.054530891 | 0.995497352 | 0.710910403 |
| iPS DF 19.11 Cells | 0.1150675 | 1.162322372 | 0.74640029 | 2.802375624 | 1.252709945 | 0.550793585 | 1.6475255 | 0.973799304 | 1.419957 | 10.54496683 | 0.635259833 | 1.59062228 | 2.763613229 | 1.653677528 | 1.215357076 |
| Mesenchymal Stem Cell Derived Adipocyte Cultured Cells | 0.3712128 | 0.959019583 | 8.946259953 | 3.423463424 | 2.087027126 | 3.865802926 | 2.6355996 | 11.27326725 | 4.739425514 | 1.84597767 | 5.360807777 | 5.374248982 | 0.387636642 | 0.565287931 | 1.226086046 |
| ES-UCSF4 Cells | 0.4515401 | 0.73987821 | 16.8355971 | 1.195956871 | 1.662422247 | 2.684614188 | 1.6754013 | 2.268103573 | 1.625701695 | 1.427631596 | 1.038894652 | 0.98640623 | 2.069149811 | 0.561783701 | 1.303470622 |
| Adipose Derived Mesenchymal Stem Cell Cultured Cells | 0.4588979 | 0.603018004 | 6.507915674 | 2.358596065 | 1.600054036 | 2.547474583 | 4.030312 | 12.83432624 | 5.625286778 | 0.924056233 | 3.477329709 | 3.595983147 | 0.272332629 | 0.515851385 | 0.868415821 |
| Bone Marrow Derived Cultured Mesenchymal Stem Cells | 0.2936865 | 2.147219272 | 5.737112507 | 4.186607766 | 0.81270348 | 4.023033326 | 1.63421 | 16.46873404 | 3.263208997 | 1.427070706 | 4.999297402 | 7.870336003 | 0.29834457 | 1.09794084 | 0.979827937 |
| Breast Myoepithelial Primary Cells | 0.2765538 | 1.074903703 | 6.229888952 | 2.554393751 | 0.906605145 | 4.789043053 | 1.0483167 | 5.986493474 | 2.017443394 | 1.86448622 | 1.855068709 | 1.099832344 | 2.577395366 | 0.433668954 | 1.189149681 |
| Breast variant Human Mammary Epithelial Cells (vHMEC) | 0.234683 | 1.18040973 | 30.9288683 | 1.012523333 | 2.91712486 | 4.536448389 | 2.4515612 | 29.25756149 | 2.11947758 | 2.701727532 | 11.18137408 | 1.637367056 | 0.571216224 | 0.551853292 | 0.66369286 |
| Primary monocytes from peripheral blood | 0.1766748 | 0.675090808 | 40.85842861 | 0.758043026 | 0.432687963 | 6.717770828 | 2.2498315 | 5.320880834 | 0.650505842 | 5.044384895 | 1.025174998 | 1.880728315 | 1.405572864 | 0.943277095 | 1.34988268 |
| Primary neutrophils from peripheral blood | 0.224796 | 1.206048217 | 11.32402866 | 1.327961049 | 0.457703032 | 3.949633234 | 2.2620078 | 10.19265139 | 1.490859393 | 3.536524465 | 0.932307868 | 1.098064264 | 1.215648019 | 2.930400766 | 0.691854329 |
| Primary B cells from cord blood | 0.238528 | 0.715684795 | 15.95672103 | 1.148543182 | 2.181470054 | 4.273849789 | 2.5493931 | 12.99908646 | 5.156191195 | 4.678753952 | 1.869706644 | 1.517270176 | 0.547176047 | 0.729439752 | 0.917490901 |
| Primary B cells from peripheral blood | 0.1698027 | 0.296358847 | 22.38978573 | 0.8615125 | 2.049003809 | 1.921043662 | 3.3743169 | 14.13633672 | 3.975917518 | 8.872283556 | 1.237668089 | 1.598351017 | 0.753845366 | 1.174854481 | 0.889061277 |
| Primary T cells from cord blood | 0.1218077 | 0.394250947 | 12.46507981 | 1.502289767 | 2.08951753 | 3.65221324 | 0.7698478 | 14.62770417 | 11.18137408 | 0.896199346 | 1.664602936 | 8.356429565 | 0.969265677 | 1.470164258 | 1.098248617 |
| Primary T cells from peripheral blood | 0.2045846 | 0.541721507 | 8.840972911 | 1.967037416 | 1.927587059 | 1.498224955 | 0.5599809 | 24.20226036 | 7.02637641 | 4.13510252 | 0.612000738 | 1.667466476 | 1.365508695 | 1.182052436 | 1.407456609 |
| Primary hematopoietic stem cells | 0.2198588 | 0.848751902 | 47.58572948 | 2.125489263 | 1.796802713 | 7.73686679 | 2.0876643 | 7.813152167 | 2.396395912 | 4.995992488 | 1.440366759 | 2.610048854 | 0.576831107 | 0.705114664 | 1.016959412 |
| Primary hematopoietic stem cells short term culture | 0.1835822 | 0.415686513 | 4.053244413 | 1.385704047 | 1.589227907 | 1.878312341 | 3.1106013 | 4.129490068 | 1.653303298 | 7.776829437 | 0.947917361 | 1.904286694 | 2.029074452 | 0.47746842 | 1.001038117 |
| Primary T helper memory cells from peripheral blood 2 | 0.1243731 | 0.961736091 | 27.26259554 | 1.473349065 | 1.989779296 | 5.787997308 | 0.7725324 | 41.19627764 | 12.85605221 | 13.32997925 | 5.598004757 | 1.664413368 | 1.828742899 | 1.314901995 | 1.874922306 |
| Primary T helper naive cells from peripheral blood 1 | 0.119484 | 0.458113095 | 14.5994956 | 1.085829424 | 3.155177427 | 5.424763605 | 0.4818117 | 26.18375694 | 19.95280626 | 7.569637664 | 4.554529996 | 1.78846941 | 1.768201401 | 1.386258912 | 1.319263683 |
| Primary T helper naive cells from peripheral blood 2 | 0.191075 | 0.305619126 | 0.443801197 | 1.98787748 | 2.98599762 | 3.01938619 | 2.3431661 | 12.78033121 | 4.868579272 | 3.608133459 | 1.507532861 | 1.49468674 | 0.863120843 | 1.335841946 | 1.01099566 |
| Primary T helper memory cells from peripheral blood 1 | 0.1543195 | 0.411537726 | 28.38242916 | 2.346629969 | 3.938132846 | 8.061221146 | 1.5130873 | 35.00434182 | 11.58483375 | 13.97992382 | 4.07900393 | 0.710253423 | 0.791393789 | 1.922638022 | 0.763669271 |
| Primary T helper cells PMA-I stimulated | 0.1401492 | 0.613571869 | 4.103676255 | 1.621296125 | 3.069858241 | 2.520925154 | 1.7255814 | 17.30683365 | 9.065224804 | 4.188812834 | 0.997795568 | 1.486243591 | 0.950791053 | 1.139114974 | 1.106915462 |
| Primary T helper 17 cells PMA-I stimulated | 0.2964013 | 0.331568069 | 0.552627336 | 1.539054792 | 1.501005155 | 3.218738785 | 1.1367263 | 18.70605006 | 5.012553772 | 5.794700755 | 2.341428513 | 1.185755504 | 0.598071329 | 3.186304457 | 1.098037509 |
| Primary T helper cells from peripheral blood | 0.1827066 | 0.401864839 | 14.17599784 | 1.356441534 | 3.888385537 | 2.407293286 | 0.5052965 | 17.54812649 | 5.520537628 | 6.118209373 | 1.036257363 | 1.684968762 | 1.185830377 | 1.192459692 | 1.213824919 |
| Primary T regulatory cells from peripheral blood | 0.184554 | 0.279241553 | 1.37646758 | 1.783185057 | 1.686205895 | 3.349860347 | 0.9540861 | 13.19102295 | 3.189068746 | 2.10885623 | 0.704963747 | 2.833407518 | 1.219055382 | 1.496412218 | 1.174478551 |
| Primary T cells effector/memory enriched from peripheral blood | 0.2437834 | 0.447005941 | 14.47391455 | 1.074281801 | 2.036248593 | 7.263175731 | 0.7582169 | 25.02481863 | 11.41828551 | 2.900882775 | 1.808059316 | 1.562001519 | 0.949563235 | 2.028712008 | 1.414647556 |
| Primary Natural Killer cells from peripheral blood | 0.3997288 | 0.253415323 | 13.29496416 | 1.349354609 | 2.016524189 | 3.665872371 | 0.7539582 | 13.36518098 | 1.870420781 | 12.0376014 | 1.485078492 | 1.69407336 | 0.577538794 | 0.952436036 | 1.247936445 |
| Primary T CD8+ naive cells from peripheral blood | 0.13103 | 0.39793431 | 33.88034474 | 1.470816717 | 3.314707967 | 6.319014677 | 0.8050488 | 23.16486482 | 13.03254875 | 5.776859131 | 3.66925022 | 1.091705729 | 0.696662442 | 1.505032164 | 1.274494676 |
| Primary T CD8+ memory cells from peripheral blood | 0.123119 | 0.561830071 | 19.61808402 | 1.573463613 | 2.4810362 | 6.5533341 | 1.018442 | 32.87578578 | 18.52029252 | 6.663771899 | 5.508382878 | 1.9253224 | 1.237986564 | 1.614062066 | 1.250943372 |
| Mesenchymal Stem Cell Derived Chondrocyte Cultured Cells | 0.1114722 | 1.793508511 | 6.753259425 | 3.198300461 | 1.971618953 | 2.882301914 | 2.5798497 | 12.65257563 | 1.717383974 | 1.695685847 | 1.605674759 | 3.9093145 | 0.319643648 | 0.861251856 | 0.356484637 |
| Primary hematopoietic stem cells G-CSF-mobilized Female | 0.2262868 | 1.083537855 | 16.06331947 | 0.748370307 | 2.041134554 | 3.184289908 | 2.4046372 | 6.660818238 | 2.220211376 | 7.924191605 | 0.478827467 | 3.333208131 | 0.457545498 | 0.401916859 | 1.023263109 |
| Primary hematopoietic stem cells G-CSF-mobilized Male | 0.2160924 | 0.999431296 | 7.13940364 | 1.014252132 | 1.698866011 | 2.878390771 | 4.7766045 | 4.638184924 | 1.868755308 | 5.300200904 | 0.369331861 | 3.493978532 | 1.126197935 | 0.419322767 | 0.855417433 |
| Muscle Satellite Cultured Cells | 0.229697 | 1.447120938 | 4.166626061 | 2.289036677 | 0.831599455 | 2.82525524 | 2.5608547 | 18.56631698 | 2.012652852 | 1.781529379 | 1.827150729 | 9.137510542 | 0.334216311 | 1.528965895 | 0.861508274 |
| Cortex derived primary cultured neurospheres | 0.1360792 | 0.571590646 | 16.71005374 | 0.695881651 | 2.009905359 | 2.67782852 | 1.5141056 | 6.407978647 | 2.206182964 | 3.575420075 | 2.248170139 | 1.808495719 | 1.08651164 | 1.619160811 | 1.080226248 |
| Ganglion Eminence derived primary cultured neurospheres | 0.1983737 | 1.078038096 | 10.71457965 | 3.161350742 | 1.464975698 | 4.248340404 | 1.5147439 | 6.0443299 | 2.114001897 | 4.616263035 | 1.092121884 | 2.337726813 | 0.852441226 | 0.328923724 | 1.17681205 |
| Foreskin Fibroblast Primary Cells skin01 | 0.3900705 | 0.644174695 | 1.065460583 | 1.34598766 | 0.901262094 | 1.106789612 | 2.5175914 | 4.826343194 | 0.923908074 | 0.989350002 | 2.114986637 | 1.146180955 | 0.559475578 | 2.13897632 | 1.385842725 |
| Foreskin Fibroblast Primary Cells skin02 | 0.5314108 | 0.343302212 | 1.260238184 | 2.889308677 | 0.341580959 | 1.244604363 | 2.3165537 | 7.501781596 | 1.164681556 | 1.164179386 | 1.599837837 | 1.153814547 | 1.174790533 | 1.792203854 | 1.042431665 |
| Foreskin Keratinocyte Primary Cells skin02 | 0.1378547 | 1.90299586 | 2.764127825 | 2.263523278 | 1.439206877 | 1.76394266 | 3.0055914 | 11.01966895 | 1.083528888 | 0.805097074 | 1.422912308 | 1.710157614 | 0.380128741 | 1.201695119 | 0.393704462 |
| Foreskin Keratinocyte Primary Cells skin03 | 0.2588519 | 1.593829058 | 2.77143543 | 2.689103169 | 1.121290165 | 1.590102185 | 3.5267185 | 9.274016913 | 0.721549422 | 0.81934455 | 1.181750826 | 1.337496404 | 0.463894729 | 1.24092038 | 0.399983537 |
| Foreskin Melanocyte Primary Cells skin01 | 0.2060709 | 1.870371297 | 3.701974864 | 1.775734196 | 1.107392382 | 3.04360995 | 2.0972274 | 3.07306947 | 0.921117742 | 1.585780996 | 0.774956358 | 3.235423697 | 0.354443076 | 1.223926707 | 0.730350337 |
| Foreskin Melanocyte Primary Cells skin03 | 0.2749052 | 1.240618785 | 5.337920017 | 1.935734882 | 0.635570919 | 5.34868875 | 3.8214269 | 6.305749199 | 0.811079818 | 1.206103212 | 0.561575123 | 0.961306052 | 2.146521221 | 0.963619669 | 0.160654919 |
| Primary mononuclear cells from peripheral blood | 0.373597 | 0.156517456 | 11.3070576 | 1.347875371 | 3.076993037 | 3.460370957 | 1.8562839 | 13.39970331 | 2.893534803 | 1.156877162 | 1.114784477 | 2.155773878 | 1.419219944 | 1.065933633 | 0.703666622 |
| Adipose Nuclei | 0.3318943 | 0.53305185 | 10.3827682 | 1.602516961 | 1.723326602 | 2.236779623 | 1.7170835 | 6.410713435 | 3.12015347 | 3.531514096 | 1.548455714 | 3.635062779 | 0.498439288 | 0.515797621 | 0.977036366 |
| Aorta | 0.4546986 | 5.946188364 | 44.2016725 | 3.42247806 | 1.228984615 | 18.09579743 | 1.2037865 | 6.696393833 | 0.651610595 | 5.528670401 | 40.51759878 | 33.06724341 | 3.500931074 | 1.363398246 | 0.936633307 |
| Liver | 0.1438443 | 0.500880527 | 20.98382661 | 2.701653123 | 0.870594352 | 2.566985685 | 1.3226945 | 5.179387856 | 2.114608914 | 21.95805267 | 4.736430459 | 1.103554901 | 1.158087997 | 2.41155451 | 0.949566927 |
| Brain Angular Gyrus | 0.5475772 | 3.111538323 | 35.91953913 | 0.655474496 | 1.591234834 | 5.300200904 | 0.5225335 | 23.41579085 | 10.52034479 | 1.699429285 | 6.762327441 | 5.883273286 | 0.790013267 | 2.991254113 | 0.623962878 |
| Brain Anterior Caudate | 0.4203631 | 2.85188523 | 16.27946652 | 0.476013414 | 2.793093863 | 3.595983147 | 0.50333 | 13.73933643 | 6.276759276 | 1.568724022 | 3.761629152 | 5.054592874 | 1.032139388 | 2.274875299 | 0.58354602 |
| Brain Cingulate Gyrus | 0.4884952 | 2.258586428 | 17.77549947 | 0.507373092 | 2.217743595 | 3.520707368 | 1.0142633 | 17.83858661 | 7.833479035 | 0.97781014 | 2.245675903 | 2.959844134 | 1.325867194 | 0.97573414 | 0.787668337 |
| Brain Germinal Matrix | 0.13471 | 0.521001316 | 9.576386406 | 0.380441313 | 2.865649365 | 4.832556897 | 1.353052 | 5.292720339 | 1.951656036 | 0.734534912 | 3.355535586 | 1.679520895 | 0.571515553 | 0.716673656 | 1.345400215 |
| Brain Hippocampus Middle | 0.772674 | 0.435787164 | 14.33693311 | 0.473767617 | 2.85784385 | 3.038521569 | 1.450489 | 14.18926857 | 6.135698574 | 0.949182927 | 1.083446901 | 6.702141807 | 0.754862404 | 0.617991375 | 0.390679822 |
| Brain Inferior Temporal Lobe | 0.4736413 | 1.549694177 | 16.63823529 | 0.991564046 | 2.179850484 | 3.046617786 | 1.6083927 | 14.62770417 | 6.54192049 | 1.151966089 | 2.504883511 | 3.607262828 | 0.750568161 | 2.441974141 | 0.603514768 |
| Brain_Dorsolateral_Prefrontal_Cortex | 0.6734865 | 1.314481128 | 15.8212015 | 1.117237642 | 2.049233354 | 3.124743513 | 2.4049072 | 14.90131827 | 6.438189551 | 1.110472254 | 3.346096378 | 3.877847943 | 1.584033404 | 1.022965934 | 0.456309493 |
| Brain Substantia Nigra | 0.2649548 | 2.075525211 | 26.41089204 | 2.018328569 | 2.403377037 | 4.929193439 | 1.3850417 | 16.29736399 | 7.953497492 | 2.284949753 | 4.751442488 | 3.541549225 | 0.836370272 | 1.733267507 | 0.770246881 |
| Colonic Mucosa | 0.6138845 | 0.398189429 | 15.3648099 | 1.649144882 | 3.363700997 | 4.261664225 | 1.7334802 | 10.57936037 | 3.012042901 | 1.095462262 | 0.864546694 | 2.162739339 | 0.717943317 | 0.654772381 | 0.760665521 |
| Colon Smooth Muscle | 0.8796877 | 0.713310361 | 13.72690654 | 2.052228142 | 1.949399479 | 4.472256485 | 0.9796988 | 14.41880353 | 10.33284639 | 1.177032259 | 3.426940188 | 3.106463081 | 1.207461395 | 0.379149454 | 0.841264404 |
| Duodenum Mucosa | 0.2110538 | 1.08296482 | 7.615568436 | 2.098317689 | 1.538783495 | 3.042875765 | 2.6203968 | 8.133193128 | 2.308517199 | 3.337947744 | 1.243532312 | 2.561191334 | 0.554524499 | 2.261555038 | 0.420527198 |
| Duodenum Smooth Muscle | 0.639221 | 1.695389768 | 6.383469595 | 2.972722046 | 1.380335054 | 4.997644484 | 1.3818408 | 8.164538363 | 5.867177741 | 1.301550013 | 1.098064264 | 1.955030477 | 1.014374766 | 0.318003845 | 0.803988447 |
| Esophagus | 0.303843 | 1.525847344 | 11.45295002 | 1.520998086 | 2.370754865 | 2.174557933 | 1.9483575 | 4.772614337 | 1.074017668 | 2.756940641 | 2.753360861 | 1.546423741 | 2.196312306 | 0.986531703 | 0.54969394 |
| Fetal Adrenal Gland | 0.1415608 | 0.428623762 | 8.554504917 | 1.962818463 | 0.945747638 | 2.433101965 | 2.3994902 | 4.465556449 | 1.873521671 | 1.374120594 | 1.329600982 | 2.004124701 | 0.611670137 | 1.753808821 | 0.634040305 |
| Fetal Brain Male | 0.2037044 | 0.65859724 | 74.35029975 | 3.49357271 | 1.854151158 | 26.31956348 | 1.9759786 | 1.936485072 | 3.046617786 | 4.977881576 | 2.820423402 | 1.733361879 | 0.818658899 | 2.233678288 | 0.312199986 |
| Fetal Brain Female | 0.1987538 | 0.630203222 | 9.576386406 | 0.732196929 | 2.691007812 | 5.915729554 | 0.8630681 | 5.069981821 | 2.078203506 | 1.519321976 | 1.171762684 | 3.251089732 | 1.944208537 | 1.255819397 | 0.96125171 |
| Fetal Heart | 0.177318 | 0.855747866 | 73.26187664 | 8.652837419 | 0.994677084 | 12.93267637 | 2.8811888 | 14.26942698 | 25.3131197 | 13.78928522 | 8.743322819 | 3.151430063 | 0.613340008 | 1.246937298 | 0.544268332 |
| Fetal Intestine Large | 0.2221516 | 0.758669031 | 9.974055041 | 2.376881482 | 2.219086962 | 2.414310492 | 1.5123843 | 2.322667706 | 0.861489342 | 1.357287444 | 2.478223168 | 2.426357889 | 1.004868178 | 0.660232806 | 0.743586447 |
| Fetal Intestine Small | 0.1107867 | 0.701089937 | 2.479700339 | 2.160695554 | 1.626309967 | 1.098148333 | 1.7649255 | 3.465199576 | 1.265099945 | 0.705428498 | 2.100035049 | 3.612564438 | 0.834969313 | 1.578557043 | 0.568659889 |
| Fetal Kidney | 0.1759032 | 3.512979678 | 24.94281809 | 1.584919519 | 2.210838381 | 17.20827988 | 2.5958624 | 18.85066617 | 14.2292329 | 1.204691989 | 10.05983136 | 7.615568436 | 1.318738705 | 1.364442398 | 0.655492155 |
| Pancreatic Islets | 0.474651 | 5.025868461 | 31.05568318 | 2.477209452 | 0.945408178 | 178.1765856 | 3.9072559 | 10.84547761 | 5.486236514 | 1.983249053 | 148.7087498 | 162.2609769 | 2.485291454 | 0.413977208 | 1.56756039 |
| Fetal Lung | 0.1133922 | 1.340965853 | 7.554451505 | 3.702023527 | 1.103595651 | 4.254386315 | 1.0438772 | 10.26224615 | 5.953261719 | 1.668212798 | 2.246701428 | 2.80008591 | 1.051587129 | 0.68931611 | 0.91140586 |
| Fetal Muscle Trunk | 0.3053169 | 0.45103486 | 9.268240237 | 1.122428719 | 1.294251024 | 3.034704796 | 1.6730389 | 12.27250522 | 3.204871089 | 1.297573984 | 1.210474431 | 1.63256971 | 0.44138887 | 1.179918322 | 0.890103963 |
| Fetal Muscle Leg | 0.1487898 | 1.247689715 | 5.222683919 | 0.684519946 | 1.193022994 | 3.939320108 | 1.3937193 | 7.31959537 | 2.151364945 | 1.114611363 | 1.951414957 | 1.42929288 | 0.902395101 | 0.614733997 | 1.011879549 |
| Placenta | 0.1567688 | 0.326798656 | 12.81267359 | 1.791933302 | 1.42788552 | 2.99772593 | 1.8299742 | 5.920395288 | 0.852801689 | 3.876841323 | 2.236392862 | 1.498106916 | 1.01881672 | 0.894172872 | 0.967486367 |
| Fetal Stomach | 0.2713924 | 0.778657781 | 4.272628082 | 2.497751703 | 1.245187784 | 1.142264748 | 1.7624928 | 2.611784926 | 1.505817932 | 1.151982503 | 0.554343599 | 2.28342013 | 0.275122065 | 1.066398038 | 1.108995327 |
| Fetal Thymus | 0.170955 | 0.388111373 | 7.398640496 | 0.848180991 | 3.215495341 | 3.542570875 | 1.3477987 | 6.313701829 | 1.087065766 | 3.071932438 | 1.582212965 | 1.976122062 | 1.00363737 | 1.180067102 | 0.671726187 |
| Gastric | 0.100754 | 1.062453491 | 15.93900151 | 1.822139984 | 1.245381894 | 1.901405416 | 3.4600467 | 7.180182383 | 2.041271778 | 323.7549003 | 59.92937411 | 47.14243936 | 154.9205885 | 1.953471609 | 0.921952098 |
| Left Ventricle | 0.2811905 | 2.952710445 | 17.83858661 | 0.968335337 | 1.968893809 | 2.372974375 | 2.5428692 | 13.12263334 | 3.539035042 | 2.186834593 | 2.898052264 | 1.501624737 | 1.313025529 | 1.21876704 | 0.253687426 |
| Lung | 0.3547552 | 0.788371599 | 14.01869619 | 2.110038198 | 1.637622368 | 1.774627511 | 4.0141823 | 2.05585478 | 0.46686979 | 11.78383976 | 7.862115546 | 1.47245432 | 1.743362701 | 0.513016133 | 0.61374862 |
| Ovary | 0.3578747 | 1.777903643 | 46.99656969 | 1.844496885 | 1.036161694 | 6.049199873 | 1.3020221 | 10.173393 | 3.968522865 | 6.488241411 | 7.907542205 | 4.64537868 | 2.26717143 | 1.418170606 | 0.835070871 |
| Pancreas | 0.2154875 | 1.615093655 | 16.51253516 | 1.541160578 | 0.978022082 | 1.971548322 | 1.6332011 | 4.496556932 | 1.140180707 | 1239.092919 | 120.5186518 | 191.6417271 | 451.3624387 | 7.14955417 | 1.365189051 |
| Placenta Amnion | 0.2386095 | 1.763600702 | 34.37951563 | 1.666200776 | 2.0652684 | 6.04676391 | 0.3369482 | 45.32559225 | 5.484231963 | 6.258448804 | 3.309647826 | 0.891461104 | 0.619087696 | 1.401622697 | 1.009788492 |
| Psoas Muscle | 0.3632013 | 4.039134271 | 45.86230516 | 2.339206133 | 0.997735951 | 23.74650519 | 1.1954878 | 41.08414988 | 9.349912187 | 1.01793893 | 2.351479901 | 5.089406006 | 1.126326834 | 2.220400283 | 0.899706948 |
| Rectal Mucosa Donor 29 | 0.4154852 | 0.835848539 | 7.431478328 | 2.209190475 | 2.19166692 | 4.329565927 | 3.6838774 | 15.185527 | 4.700774458 | 1.102452507 | 1.969699399 | 2.425360298 | 0.606833013 | 1.130258449 | 0.474624105 |
| Rectal Mucosa Donor 31 | 0.1099867 | 0.698457148 | 11.2564475 | 1.473147253 | 2.879692051 | 9.542702591 | 2.4240436 | 19.69441849 | 5.62107234 | 2.703977265 | 2.136695736 | 2.863955202 | 1.185930212 | 1.245849809 | 0.307863861 |
| Rectal Smooth Muscle | 0.55626 | 1.327783178 | 10.24824774 | 0.623381415 | 3.085475922 | 7.4278151 | 1.4804303 | 15.52863292 | 5.292720339 | 0.813801136 | 4.75445594 | 11.63843977 | 0.74636043 | 0.932930546 | 0.778788669 |
| Right Atrium | 0.7369802 | 0.979221506 | 0.479411217 | 11.91407198 | 1.429943006 | 8.27220135 | 2.9491501 | 10.62405527 | 2.667233339 | 22.02193925 | 65.9685981 | 56.58153953 | 11.47908653 | 0.623409665 | 0.621878149 |
| Right Ventricle | 0.475733 | 0.879431034 | 10.22732499 | 0.997508349 | 1.663307647 | 1.684163938 | 2.7668841 | 31.31248572 | 6.637282369 | 3.128030307 | 2.549235846 | 4.28364881 | 1.562671227 | 0.937367431 | 0.663226917 |
| Sigmoid Colon | 0.1153815 | 1.968908052 | 22.98889701 | 1.610561601 | 3.603783737 | 3.75406666 | 1.3838549 | 11.90467446 | 2.626596229 | 2.655293164 | 6.690464121 | 1.091123629 | 0.526873428 | 1.187729005 | 0.550402153 |
| Skeletal Muscle Male | 0.2772683 | 0.580976933 | 2.310321972 | 0.95026472 | 1.043176517 | 1.723451879 | 2.7946216 | 9.619093416 | 3.535688406 | 1.166538876 | 1.861625981 | 0.895179916 | 2.005023181 | 1.249261083 | 0.580239819 |
| Skeletal Muscle Female | 0.3009625 | 0.975383962 | 14.75986694 | 1.030218519 | 1.264484447 | 2.009855177 | 1.3014555 | 18.27182431 | 5.502325393 | 1.272676367 | 0.810238831 | 1.232067935 | 1.67357201 | 1.79796261 | 0.481611305 |
| Small Intestine | 0.1175079 | 5.317108969 | 122.6818424 | 1.576695781 | 1.957996041 | 35.5809766 | 1.2230963 | 27.91319332 | 8.106358113 | 2.273691838 | 25.02481863 | 2.509555194 | 0.723852519 | 1.4190718 | 1.097028271 |
| Stomach Mucosa | 0.1371209 | 0.916536461 | 37.07039697 | 3.487058121 | 2.13645721 | 8.702869324 | 1.3105651 | 39.04180641 | 16.96047913 | 2.675895961 | 3.018772895 | 3.950256776 | 1.446895863 | 0.503186289 | 0.785698401 |
| Stomach Smooth Muscle | 0.3621873 | 0.756649587 | 4.20767971 | 2.530970748 | 0.951273663 | 4.387669074 | 0.8252512 | 16.81767868 | 7.849817087 | 1.085829424 | 2.272177247 | 1.738807354 | 1.594501174 | 0.701584207 | 0.642142255 |
| Thymus | 0.3977791 | 0.606484299 | 7.039484849 | 1.540103721 | 1.860179495 | 3.761629152 | 1.0425808 | 4.727467273 | 1.487811325 | 1.181106511 | 1.067288733 | 3.701057894 | 1.395156947 | 1.992239279 | 0.805260754 |
| Spleen | 0.1487578 | 0.816350629 | 12.43426957 | 1.360691895 | 2.479879907 | 1.947905264 | 1.1569713 | 2.141355464 | 0.77031335 | 15.23140934 | 6.499469335 | 1.047115843 | 3.0150983 | 1.199478368 | 0.765622378 |
| A549 EtOH 0.02pct Lung Carcinoma Cell Line | 0.3819233 | 0.655070614 | 3.331123864 | 1.208495345 | 1.520761253 | 2.455826763 | 1.9577372 | 8.52061926 | 4.569957375 | 1.201578564 | 5.202751327 | 10.97161287 | 3.15629739 | 1.990368692 | 0.654071503 |
| Dnd41 TCell Leukemia Cell Line | 0.1965224 | 0.302653469 | 1.513170631 | 1.112802919 | 1.729343665 | 2.436902857 | 1.9838264 | 9.54007033 | 1.3524773 | 14.2426053 | 2.216862315 | 6.561920335 | 6.876668822 | 1.476652521 | 0.995254278 |
| GM12878 Lymphoblastoid Cells | 0.2252983 | 1.666200776 | 7.833479035 | 2.209863093 | 0.840263324 | 2.409640493 | 1.6212351 | 25.27110521 | 2.101933961 | 0.66429415 | 0.786912937 | 1.83353611 | 1.783017635 | 0.634440458 | 1.16471384 |
| HeLa-S3 Cervical Carcinoma Cell Line | 0.266357 | 0.553894565 | 9.098274314 | 2.393583948 | 1.637583318 | 3.381044004 | 3.4416497 | 12.07612656 | 0.606082409 | 5.996056218 | 1.533726832 | 4.075741798 | 0.701833094 | 1.093734383 | 0.475943465 |
| HepG2 Hepatocellular Carcinoma Cell Line | 0.182043 | 1.275197509 | 6.533386196 | 3.161003742 | 0.646444055 | 2.579053037 | 4.1456409 | 9.182094526 | 2.027809286 | 1.179636073 | 8.231802815 | 12.8778519 | 1.254547538 | 1.096618587 | 0.92490216 |
| HMEC Mammary Epithelial Primary Cells | 0.1895575 | 1.57755024 | 17.1496913 | 1.471490397 | 1.000904752 | 5.502325393 | 4.3099266 | 13.68975337 | 4.187639249 | 1.63352131 | 14.712999 | 8.919689981 | 0.729912886 | 1.699520218 | 0.787936045 |
| HSMM Skeletal Muscle Myoblasts Cells | 0.1405958 | 1.16463916 | 11.34105063 | 2.739728947 | 1.885172746 | 4.535064147 | 2.060012 | 31.05568318 | 1.668415028 | 3.565190245 | 1.571259603 | 4.427272283 | 0.70699202 | 1.595504 | 0.583974951 |
| HSMM cell derived Skeletal Muscle Myotubes Cells | 0.1142355 | 1.217766447 | 3.830113365 | 0.656314847 | 0.648369614 | 7.275030002 | 3.8156159 | 4.525400697 | 1.014827065 | 3.156581803 | 2.807618088 | 3.445964728 | 0.720728502 | 2.121019309 | 1.165308567 |
| HUVEC Umbilical Vein Endothelial Primary Cells | 0.2232393 | 0.808563954 | 6.029766721 | 0.912468439 | 1.460674779 | 5.927407643 | 4.9645575 | 31.11948493 | 5.027536867 | 26.73563868 | 41.99711708 | 85.1983714 | 1.182833793 | 1.44311422 | 0.819307792 |
| K562 Leukemia Cells | 0.2346715 | 0.925564101 | 3.533182624 | 0.887215647 | 1.270216716 | 2.806025508 | 2.3079567 | 15.12478404 | 5.761337163 | 10.798917 | 13.20249104 | 8.672779653 | 0.816966696 | 2.418291906 | 0.849732953 |
| Monocytes-CD14+ RO01746 Primary Cells | 0.1798046 | 1.279574075 | 8.240778435 | 0.686189851 | 0.44559294 | 7.956228353 | 2.6377042 | 5.691423883 | 1.662520011 | 2.137315855 | 0.485747986 | 2.683580928 | 1.325383186 | 0.817688617 | 1.170663557 |
| NH-A Astrocytes Primary Cells | 0.2309376 | 0.668563388 | 10.53503732 | 1.82211877 | 0.495642689 | 4.909904195 | 4.0805224 | 16.06331947 | 1.902143879 | 1.719199938 | 1.926584454 | 4.833919655 | 0.434954759 | 1.153330966 | 1.113035333 |
| NHDF-Ad Adult Dermal Fibroblast Primary Cells | 0.3983474 | 1.562281198 | 10.31155889 | 0.951214652 | 1.863202012 | 3.693738599 | 2.6169661 | 26.82991026 | 5.383890799 | 2.174879188 | 8.047841705 | 5.708743435 | 0.411563573 | 1.034780377 | 1.036290398 |
| NHEK-Epidermal Keratinocyte Primary Cells | 0.1162253 | 1.558766119 | 3.595118432 | 2.172311243 | 1.883488575 | 5.145868092 | 2.3180143 | 12.51677 | 0.767569949 | 1.075955417 | 1.400916564 | 9.929648091 | 0.322276535 | 0.822128803 | 0.505739207 |
| NHLF Lung Fibroblast Primary Cells | 0.4131214 | 1.35961455 | 3.565190245 | 1.841046306 | 1.9774746 | 2.19106741 | 4.1228192 | 15.49679605 | 1.905597599 | 1.557495661 | 6.027346316 | 6.201587247 | 0.377292794 | 0.236235425 | 0.844566627 |
| Osteoblast Primary Cells | 0.2128499 | 1.549997694 | 5.988881311 | 3.370406914 | 1.178752253 | 3.139256783 | 1.6245372 | 26.64203417 | 5.046083386 | 1.280795596 | 2.718559083 | 12.33454229 | 0.313159486 | 0.226781808 | 1.896775536 |

Table notes: AD: Alzheimer's disease; ADNI: AD Neuroimaging Initiative; ROSMAP: Religious Orders Study and Rush Memory and Aging Project.
