# Supplementary material for: Genetic and pathway complexity in Alzheimer’s disease: Insights from multi-omic data about the immune response and mitochondrial function
Source: Neural Regen Res. 2025 Sep 29;21(9):4406–23. doi: 10.4103/NRR.NRR-D-25-00184 (PMC13557549; doi:10.4103/NRR.NRR-D-25-00184)
Supplement: Supplementary file 5 [file NRR-21-4406_Suppl13.pdf]

**Additional Table 13 Enrichment results of histone modifications at important CpGs sites**

**ADNI**

| cell | D0se | H2A | H3K27ac | H3K27me3 | H3K4me1 | H3K4me2 | H3K4me3 | H3K79me2 | H3K9ac | H3K9me3 | H4K20me1 |
|---|---|---|---|---|---|---|---|---|---|---|---|
| ES-I3 Cells | 0 | 0 | 0 | 2.512651 | 2.4986419 | 0 | 1.4251259 | 0 | 1.5178143 | 0.4183516 | 0 |
| ES-WA7 Cells | 0 | 0 | 0 | 2.7185914 | 1.2671203 | 0 | 0.9601891 | 0 | 0.664834 | 0 | 0 |
| H1 Cells | 1.3542485 | 0.8498465 | 0.8075114 | 2.6464258 | 2.430338 | 1.8370212 | 1.1891053 | 1.1226241 | 0.7907676 | 0.9993892 | 1.4461468 |
| H1 BMP4 Derived Mesendoderm Cultured Cells | 1.1672805 | 0 | 0.9909667 | 2.5950298 | 3.0519329 | 1.2150026 | 0.9523054 | 1.2434043 | 1.1078988 | 2.1839924 | 0 |
| H1 BMP4 Derived Trophoblast Cultured Cells | 0.6771678 | 1.1057521 | 1.187831 | 1.4375677 | 1.6600136 | 1.2929677 | 0.9028219 | 0.6929397 | 0.793809 | 1.0317115 | 0 |
| H1 Derived Mesenchymal Stem Cells | 1.3917494 | 0.4658524 | 1.2837453 | 2.3074106 | 1.3808817 | 1.5335096 | 1.0860734 | 0 | 0.7880561 | 0.7978871 | 0 |
| H1 Derived Neuro0l Progenitor Cultured Cells | 1.304012 | 0 | 0.7194421 | 1.8446053 | 1.5830034 | 1.1984449 | 0.8568244 | 0 | 0.9851478 | 1.1265203 | 0 |
| H9 Cells | 1.6487696 | 1.033647 | 0.6365456 | 3.062353 | 1.2891943 | 1.2152047 | 1.2615897 | 1.2179215 | 1.1755865 | 1.433603 | 1.386254 |
| H9 Derived Neuro0l Progenitor Cultured Cells | 0 | 1.1344129 | 0 | 2.3554533 | 2.1163746 | 0 | 0.8047153 | 0 | 0 | 1.1262096 | 0 |
| H9 Derived Neuron Cultured Cells | 0 | 0.8793595 | 0 | 2.4750378 | 1.9059732 | 0 | 0.8886036 | 0 | 0 | 0.9224113 | 0 |
| hESC Derived CD184+ Endoderm Cultured Cells | 0 | 0 | 1.1109788 | 2.4306817 | 1.9218288 | 0 | 1.4618178 | 0 | 0.9655237 | 1.1451008 | 0 |
| hESC Derived CD56+ Ectoderm Cultured Cells | 0 | 0 | 0.7852323 | 2.7001761 | 1.9714942 | 0 | 1.4153418 | 0 | 0 | 0.7465496 | 0 |
| hESC Derived CD56+ Mesoderm Cultured Cells | 0 | 0 | 1.3063312 | 1.7528045 | 1.8633807 | 0 | 1.4592137 | 0 | 0 | 0.9094278 | 0 |
| HUES48 Cells | 0 | 0 | 0.8762044 | 2.9605546 | 1.9988193 | 0 | 1.5712255 | 0 | 1.2627077 | 0.7056325 | 0 |
| HUES6 Cells | 0 | 0 | 0.6466997 | 3.0072191 | 2.6378291 | 0 | 1.4719489 | 0 | 1.2750103 | 0.5452545 | 0 |
| HUES64 Cells | 0 | 0 | 0.5151075 | 2.8284845 | 2.1318453 | 0 | 1.4703587 | 0 | 1.1822676 | 1.2707878 | 0 |
| IMR90 fetal lung fibroblasts Cell Line | 0.9277544 | 0.8812318 | 0.9800167 | 1.4126295 | 1.9455981 | 1.3226613 | 0.9627388 | 1.9618415 | 1.145827 | 0.7846891 | 1.7066416 |
| iPS-15b Cells | 0 | 0 | 0 | 2.9645279 | 2.6268611 | 0 | 1.482951 | 0 | 0.9273267 | 0.7125267 | 0 |
| iPS-18 Cells | 0 | 0 | 0.4647353 | 3.370313 | 1.7964617 | 0 | 1.6110794 | 0 | 1.2099813 | 0.5397722 | 0 |
| iPS-20b Cells | 0 | 0 | 0.7099622 | 2.6559963 | 1.9496859 | 0 | 1.4268418 | 0 | 1.3771384 | 1.4162432 | 0 |
| iPS DF 6.9 Cells | 1.4551728 | 0 | 0.4554984 | 2.9768562 | 2.2933833 | 0 | 1.0974807 | 0 | 0 | 2.2077678 | 0 |
| iPS DF 19.11 Cells | 1.411625 | 0 | 0.8934536 | 2.0428748 | 1.6384571 | 0 | 1.0211818 | 0 | 0 | 0.978462 | 0 |
| Mesenchymal Stem Cell Derived Adipocyte Cultured Cells | 0 | 0 | 0 | 1.709428 | 1.4926942 | 0 | 1.2833398 | 0 | 1.1774251 | 1.4448574 | 0 |
| ES-UCSF4 Cells | 0 | 0 | 0 | 1.7663298 | 1.9846094 | 0 | 1.2619696 | 0 | 0 | 0.9319678 | 0 |
| Adipose Derived Mesenchymal Stem Cell Cultured Cells | 0 | 0 | 0 | 1.8469859 | 1.8737296 | 0 | 1.4257108 | 0 | 1.5315417 | 0.5164014 | 0 |
| Bone Marrow Derived Cultured Mesenchymal Stem Cells | 0 | 0 | 1.1847217 | 2.0831777 | 1.8422186 | 0 | 1.3654473 | 0 | 1.1739644 | 0.4159106 | 0 |
| Breast Myoepithelial Primary Cells | 0 | 0 | 0 | 1.4053399 | 2.3482668 | 0 | 1.4394152 | 0 | 1.0763611 | 0.7721666 | 0 |
| Breast variant Human Mammary Epithelial Cells (vHMEC) | 1.0809864 | 0 | 0 | 1.3117725 | 1.5644357 | 0 | 0.9755877 | 0 | 0 | 0.3101945 | 0 |
| Primary monocytes from peripheral blood | 0.8144852 | 0 | 0.6557201 | 2.5004577 | 1.5234095 | 0 | 0.6105486 | 0 | 0 | 0.8446634 | 0 |
| Primary neutrophils from peripheral blood | 0 | 0 | 0 | 3.128417 | 0.9830847 | 0 | 0.7396953 | 0 | 0 | 0.8899358 | 0 |
| Primary B cells from cord blood | 0 | 0 | 0 | 3.098611 | 1.4708567 | 0 | 0.7371739 | 0 | 0 | 0 | 0 |
| Primary B cells from peripheral blood | 1.153576 | 0 | 0.7865873 | 2.6201507 | 1.6415211 | 0 | 0.7124475 | 0 | 0 | 0.4249602 | 0 |
| Primary T cells from cord blood | 0.9133939 | 0 | 0 | 3.9994434 | 1.8630223 | 0 | 0.8626225 | 0 | 0 | 0 | 0 |
| Primary T cells from peripheral blood | 0.9739176 | 0 | 0.9919711 | 1.9548387 | 1.580449 | 0 | 0.6990232 | 0 | 0 | 3.1661719 | 0 |
| Primary hematopoietic stem cells | 0 | 0 | 0 | 4.1421329 | 1.4285033 | 0 | 1.0318542 | 0 | 0 | 1.6219428 | 0 |
| Primary hematopoietic stem cells short term culture | 0 | 0 | 0 | 3.1039677 | 1.3514489 | 0 | 0.8219435 | 0 | 0 | 1.1610446 | 0 |
| Primary T helper memory cells from peripheral blood 2 | 0 | 0 | 0.5111553 | 2.9891517 | 1.5004066 | 0 | 0.6079218 | 0 | 0 | 0 | 0 |
| Primary T helper 0ive cells from peripheral blood 1 | 0 | 0 | 0.5838434 | 2.4291597 | 1.3529144 | 0 | 0.7944569 | 0 | 0.5352701 | 0 | 0 |
| Primary T helper 0ive cells from peripheral blood 2 | 0 | 0 | 0.7545075 | 2.9008483 | 1.629799 | 0 | 0.9186774 | 0 | 0 | 1.4940204 | 0 |
| Primary T helper memory cells from peripheral blood 1 | 0 | 0 | 0.7633825 | 3.3378412 | 1.5923388 | 0 | 0.8940696 | 0 | 0 | 0 | 0 |
| Primary T helper cells PMA-I stimulated | 0 | 0 | 0.8423844 | 2.8337126 | 1.5073532 | 0 | 0.9590421 | 0 | 0 | 0 | 0 |
| Primary T helper 17 cells PMA-I stimulated | 0 | 0 | 0.6159945 | 2.9731486 | 1.2989228 | 0 | 0.8512042 | 0 | 0 | 0.5850266 | 0 |
| Primary T helper cells from peripheral blood | 0 | 0 | 0.6522584 | 3.4692965 | 1.7382292 | 0 | 0.9372325 | 0 | 0 | 0.9492091 | 0 |
| Primary T regulatory cells from peripheral blood | 0 | 0 | 0.6280389 | 2.6281541 | 1.6122938 | 0 | 1.0358836 | 0 | 0 | 0.7937976 | 0 |
| Primary T cells effector/memory enriched from peripheral blood | 0 | 0 | 0.6372768 | 2.7084479 | 1.1756831 | 0 | 0.8550212 | 0 | 0 | 0 | 0 |
| Primary 0tural Killer cells from peripheral blood | 1.1048657 | 0 | 0.9506564 | 2.8076384 | 1.5150731 | 0 | 0.6368974 | 0 | 0 | 1.2392902 | 0 |
| Primary T CD8+ 0ive cells from peripheral blood | 0 | 0 | 0.6122984 | 2.9504128 | 1.736768 | 0 | 0.6489147 | 0 | 0.4300117 | 1.7404144 | 0 |
| Primary T CD8+ memory cells from peripheral blood | 0 | 0 | 0.5126977 | 2.8103391 | 1.4596943 | 0 | 0.7433581 | 0 | 0 | 2.5879812 | 0 |
| Mesenchymal Stem Cell Derived Chondrocyte Cultured Cells | 0 | 0 | 1.317683 | 1.7954274 | 2.1554021 | 0 | 1.5239297 | 0 | 1.3048518 | 0.283384 | 0 |
| Primary hematopoietic stem cells G-CSF-mobilized Female | 0.9921507 | 0 | 0.6970623 | 3.0164882 | 1.8353696 | 0 | 1.2020048 | 0 | 0 | 0.7007557 | 0 |
| Primary hematopoietic stem cells G-CSF-mobilized Male | 0.8940185 | 0 | 0 | 3.0094302 | 1.8055768 | 0 | 1.2243355 | 0 | 0 | 1.112946 | 0 |
| Muscle Satellite Cultured Cells | 0 | 0 | 0 | 2.0646337 | 1.6391089 | 1.475331 | 1.0395261 | 0 | 1.0025258 | 0.3920113 | 0 |
| Cortex derived primary cultured neurospheres | 0 | 0 | 0 | 2.2568166 | 2.0028689 | 0 | 0.9994723 | 0 | 0 | 0.2344626 | 0 |
| Ganglion Eminence derived primary cultured neurospheres | 0 | 0 | 0 | 2.4431598 | 1.825484 | 0 | 1.2209899 | 0 | 0 | 0 | 0 |
| Foreskin Fibroblast Primary Cells skin01 | 1.2128432 | 0 | 1.2139637 | 1.419367 | 1.4857694 | 0 | 1.2478336 | 0 | 0 | 0.7228851 | 0 |
| Foreskin Fibroblast Primary Cells skin02 | 1.302658 | 0 | 1.1182009 | 1.5275889 | 1.7680815 | 0 | 1.3842956 | 0 | 0 | 0.8858466 | 0 |
| Foreskin Keratinocyte Primary Cells skin02 | 1.0163847 | 0 | 0 | 1.4796804 | 2.3314468 | 0 | 1.4010123 | 0 | 0 | 0.4638968 | 0 |
| Foreskin Keratinocyte Primary Cells skin03 | 0 | 0 | 1.067868 | 1.7075595 | 2.2895621 | 0 | 1.6503459 | 0 | 0 | 0.9030256 | 0 |
| Foreskin Melanocyte Primary Cells skin01 | 1.1523886 | 0 | 0.6722652 | 2.29006 | 1.1329299 | 0 | 0.928654 | 0 | 0 | 0.9335387 | 0 |
| Foreskin Melanocyte Primary Cells skin03 | 0 | 0 | 0.772309 | 2.1334301 | 1.692993 | 0 | 0.9066575 | 0 | 0 | 0.9565976 | 0 |
| Primary mononuclear cells from peripheral blood | 0 | 0 | 0.5292519 | 3.030559 | 0.7549872 | 0 | 0.8095373 | 0 | 0.6825336 | 0 | 0 |
| Adipose Nuclei | 0 | 0 | 1.1148867 | 1.6604146 | 1.3206866 | 0 | 1.5633055 | 0 | 1.1328544 | 0.9139816 | 0 |
| Aorta | 0 | 0 | 0.9190621 | 1.1058404 | 1.5982376 | 0 | 1.0128858 | 0 | 0 | 1.3513439 | 0 |
| Liver | 0 | 0 | 0.8453792 | 2.5583018 | 1.1461005 | 0 | 0.7830821 | 0 | 0.9429522 | 0.9320589 | 0 |
| Brain Angular Gyrus | 0 | 0 | 1.1238871 | 2.4476831 | 1.8827406 | 0 | 0.9781102 | 0 | 1.0856369 | 1.6110831 | 0 |
| Brain Anterior Caudate | 0 | 0 | 1.363018 | 2.3526868 | 2.4390048 | 0 | 1.4023973 | 0 | 0.7988216 | 1.0142174 | 0 |
| Brain Cingulate Gyrus | 0 | 0 | 1.1258131 | 2.2086952 | 1.9595391 | 0 | 1.7851088 | 0 | 1.421025 | 0.6656176 | 0 |
| Brain Germi0l Matrix | 0 | 0 | 0 | 1.5820519 | 2.818833 | 0 | 1.3949899 | 0 | 0 | 0.916803 | 0 |
| Brain Hippocampus Middle | 0 | 0 | 1.2052971 | 2.330652 | 2.1306031 | 0 | 1.3029774 | 0 | 0 | 0.5205647 | 0 |
| Brain Inferior Temporal Lobe | 0 | 0 | 1.1492326 | 2.4549734 | 1.7888685 | 0 | 1.7547513 | 0 | 0.9955947 | 1.4138405 | 0 |
| Brain_Dorsolateral_Prefrontal_Cortex | 0 | 0 | 1.3148829 | 2.2482732 | 1.8529055 | 0 | 1.6542108 | 0 | 1.1880644 | 0.4326652 | 0 |
| Brain Substantia Nigra | 0 | 0 | 1.0502779 | 2.8236226 | 1.973289 | 0 | 1.3698384 | 0 | 0.9400452 | 0.5890357 | 0 |
| Colonic Mucosa | 0 | 0 | 1.2646259 | 2.540261 | 1.5547894 | 0 | 1.6242966 | 0 | 1.3827633 | 0.6960544 | 0 |
| Colon Smooth Muscle | 0 | 0 | 1.4093513 | 2.1128863 | 1.8675952 | 0 | 1.529695 | 0 | 1.1985328 | 0 | 0 |
| Duodenum Mucosa | 0 | 0 | 0 | 3.0960654 | 1.6582067 | 0 | 1.4828027 | 0 | 0 | 0.6765154 | 0 |
| Duodenum Smooth Muscle | 0 | 0 | 1.4472837 | 2.3827493 | 1.5144565 | 0 | 1.5909624 | 0 | 1.0038476 | 0.5051988 | 0 |
| Esophagus | 0 | 0 | 1.6219053 | 2.5715514 | 2.3494972 | 0 | 1.8868351 | 0 | 0 | 0.7830978 | 0 |
| Fetal Adre0l Gland | 0.9644473 | 0 | 1.2269514 | 2.1882891 | 1.62377 | 0 | 1.3460051 | 0 | 0 | 1.1466363 | 0 |
| Fetal Brain Male | 1.2641404 | 0 | 0 | 2.8707673 | 2.3372476 | 0 | 0.7923622 | 0 | 0 | 0.8483771 | 0 |
| Fetal Brain Female | 1.4021187 | 0 | 0 | 1.9997093 | 1.6314232 | 0 | 1.3445993 | 0 | 0 | 0.8381863 | 0 |
| Fetal Heart | 1.1843373 | 0 | 0 | 3.0005344 | 1.7049514 | 0 | 0.931655 | 0 | 1.3878006 | 1.104899 | 0 |
| Fetal Intestine Large | 0.8288382 | 0 | 1.0314746 | 3.3494908 | 1.6179178 | 0 | 1.0667908 | 0 | 0 | 1.3455628 | 0 |
| Fetal Intestine Small | 0.7002483 | 0 | 1.0183974 | 3.443786 | 1.5370289 | 0 | 1.029489 | 0 | 0 | 1.0809477 | 0 |
| Fetal Kidney | 1.2964613 | 0 | 0 | 1.7850836 | 2.6719445 | 0 | 1.1066057 | 0 | 1.0774825 | 1.8528104 | 0 |
| Pancreatic Islets | 0 | 0 | 0.5007359 | 2.5832463 | 2.061279 | 0 | 0.8267269 | 0 | 0.5698461 | 0 | 0 |
| Fetal Lung | 1.1614284 | 0 | 1.1864592 | 1.1908671 | 1.5515915 | 0 | 0.9856027 | 0 | 1.0171674 | 1.0943859 | 0 |
| Fetal Muscle Trunk | 1.2516374 | 0 | 1.06687 | 1.685143 | 1.4849274 | 0 | 0.9166783 | 0 | 0 | 1.2502429 | 0 |
| Fetal Muscle Leg | 0.9974275 | 0 | 0.868856 | 2.2566408 | 1.8952416 | 0 | 1.1551401 | 0 | 0 | 0.387714 | 0 |
| Placenta | 1.0478898 | 0 | 0.848245 | 1.8135909 | 1.7178812 | 0 | 0.7725057 | 0 | 0 | 0.6771515 | 0 |
| Fetal Stomach | 1.4533146 | 0 | 1.3391377 | 1.814982 | 2.0398137 | 0 | 1.2423341 | 0 | 0 | 1.4413718 | 0 |
| Fetal Thymus | 1.0166545 | 0 | 1.0043621 | 3.2840211 | 1.564038 | 0 | 0.867468 | 0 | 0 | 1.193818 | 0 |
| Gastric | 0.5900466 | 0 | 0.7481539 | 1.9063981 | 2.027597 | 0 | 0.5737013 | 0 | 0 | 1.6245357 | 0 |
| Left Ventricle | 0 | 0 | 1.0130548 | 2.4976381 | 1.7980813 | 0 | 0.9319659 | 0 | 0 | 1.6624447 | 0 |
| Lung | 0 | 0 | 1.2839436 | 2.3043088 | 1.8705989 | 0 | 0.9176273 | 0 | 0 | 0.9966353 | 0 |
| Ovary | 1.3387713 | 0 | 1.0687876 | 1.2505872 | 1.7095569 | 0 | 1.0291944 | 0 | 0 | 1.2979983 | 0 |
| Pancreas | 0.9103394 | 0 | 0.7808557 | 4.4146144 | 2.1839001 | 0 | 0.8343733 | 0 | 0 | 1.6354227 | 0 |
| Placenta Amnion | 0 | 0 | 0.850985 | 2.1858565 | 1.4112415 | 0 | 0.8777799 | 0 | 0 | 0 | 0 |
| Psoas Muscle | 1.0261395 | 0 | 0.710296 | 2.2090037 | 1.6343294 | 0 | 1.0532868 | 0 | 0 | 0.845496 | 0 |
| Rectal Mucosa Donor 29 | 0 | 0 | 1.1680501 | 3.5160128 | 1.8616199 | 0 | 1.4304194 | 0 | 1.0673701 | 0.5457474 | 0 |
| Rectal Mucosa Donor 31 | 0 | 0 | 0.9492892 | 3.0372338 | 1.5886324 | 0 | 1.24786 | 0 | 1.2635864 | 0.6369936 | 0 |
| Rectal Smooth Muscle | 0 | 0 | 1.2565921 | 1.887765 | 2.2972829 | 0 | 1.4725996 | 0 | 1.0805012 | 1.7718594 | 0 |
| Right Atrium | 0 | 0 | 1.0310784 | 1.4662173 | 2.3876574 | 0 | 1.1859672 | 0 | 0 | 0 | 0 |
| Right Ventricle | 0 | 0 | 0.912783 | 1.5924096 | 1.5419723 | 0 | 1.1874103 | 0 | 0 | 0 | 0 |
| Sigmoid Colon | 0 | 0 | 1.2750727 | 2.3505433 | 1.6892603 | 0 | 0.9178531 | 0 | 0 | 0.3774483 | 0 |
| Skeletal Muscle Male | 0 | 0 | 0 | 2.8603008 | 1.6587765 | 0 | 1.0413775 | 0 | 1.0888807 | 0.3404854 | 0 |
| Skeletal Muscle Female | 0 | 0 | 1.0126697 | 2.7914907 | 1.6383707 | 0 | 1.2349503 | 0 | 1.1566141 | 0.4858829 | 0 |
| Small Intestine | 0.7775506 | 0 | 1.0759491 | 3.2315667 | 1.669314 | 0 | 0.6879066 | 0 | 0 | 1.4805932 | 0 |
| Stomach Mucosa | 0 | 0 | 0 | 3.4938869 | 1.5712851 | 0 | 1.1035843 | 0 | 1.1234649 | 2.6504364 | 0 |
| Stomach Smooth Muscle | 0 | 0 | 1.1354317 | 1.4762616 | 1.8282868 | 0 | 1.6593001 | 0 | 0.9807216 | 1.0663716 | 0 |
| Thymus | 0 | 0 | 0.8690209 | 3.2691605 | 1.30249 | 0 | 0.8904146 | 0 | 0 | 0.9882343 | 0 |
| Spleen | 0 | 0 | 1.3177626 | 2.5717717 | 1.4141282 | 0 | 0.6509269 | 0 | 0 | 1.2123924 | 0 |
| A549 EtOH 0.02pct Lung Carcinoma Cell Line | 0.9557326 | 0.7413183 | 1.0767977 | 2.2296596 | 1.6764489 | 1.3272279 | 1.2266055 | 1.0236083 | 1.2203547 | 0.439983 | 0.7981301 |
| Dnd41 TCell Leukemia Cell Line | 0 | 0.5414732 | 0.4903764 | 2.8297177 | 0.8532469 | 0.6317664 | 0.5089202 | 0.5981837 | 0.5790032 | 1.3070223 | 1.0836895 |
| GM12878 Lymphoblastoid Cells | 0.8505 | 0.6540969 | 0.6338033 | 2.593158 | 1.603522 | 1.0686868 | 0.8751144 | 0.5240126 | 0.6448604 | 1.2811705 | 1.5184977 |
| HeLa-S3 Cervical Carcinoma Cell Line | 0.8164451 | 0.8007631 | 0.9102296 | 1.1026981 | 1.7844022 | 1.0388217 | 0.8541841 | 0.6661546 | 0.6757766 | 2.3551924 | 0.675101 |
| HepG2 Hepatocellular Carcinoma Cell Line | 0.7986043 | 0.1543839 | 0.3759887 | 1.5020323 | 1.2470816 | 0.6996088 | 0.7326269 | 0.6508947 | 0.4116586 | 0 | 2.2693654 |
| HMEC Mammary Epithelial Primary Cells | 1.0487069 | 1.2518443 | 1.0283285 | 1.995295 | 1.7085845 | 1.4282088 | 1.1604004 | 0.8932921 | 1.0716697 | 0.830171 | 2.1615729 |
| HSMM Skeletal Muscle Myoblasts Cells | 1.0099021 | 1.0206527 | 1.1032606 | 2.0248873 | 1.6332239 | 1.1512003 | 1.0621881 | 1.8649085 | 1.2414719 | 1.1164167 | 2.1922986 |
| HSMM cell derived Skeletal Muscle Myotubes Cells | 1.0218618 | 1.0813613 | 1.1017594 | 1.4678032 | 1.5290291 | 1.4614599 | 1.2247618 | 1.3864929 | 1.2142906 | 0.2932243 | 0.695756 |
| HUVEC Umbilical Vein Endothelial Primary Cells | 0.9092071 | 0.8862085 | 0.7890824 | 2.7953555 | 1.4617031 | 1.116416 | 1.0071645 | 0.8844239 | 0.7685006 | 0.3347115 | 0.5778216 |
| K562 Leukemia Cells | 0.4439715 | 0.4599568 | 0.4182746 | 2.166053 | 0.9015903 | 0.5173403 | 0.4277951 | 0.4387334 | 0.4100482 | 1.5146637 | 1.8471258 |
| Monocytes-CD14+ RO01746 Primary Cells | 0.9337747 | 0.6661087 | 0.7506647 | 2.6773674 | 1.3572546 | 1.2344901 | 1.1924949 | 0.7401421 | 0.5869318 | 2.3307053 | 1.0341348 |
| NH-A Astrocytes Primary Cells | 0.9776255 | 0.9580379 | 0.9015963 | 2.707666 | 1.7323858 | 1.8367994 | 1.1676104 | 1.2348842 | 1.0046016 | 1.4395115 | 1.6950467 |
| NHDF-Ad Adult Dermal Fibroblast Primary Cells | 1.1764362 | 0.8526432 | 0.9850432 | 1.7560271 | 1.5096884 | 1.3053484 | 1.0259835 | 1.729478 | 1.1054163 | 0 | 2.2466203 |
| NHEK-Epidermal Keratinocyte Primary Cells | 1.0504773 | 1.3116883 | 1.141802 | 1.7819841 | 2.1061063 | 1.9381548 | 1.5754288 | 0.9371852 | 1.2349886 | 1.1883587 | 1.62986 |
| NHLF Lung Fibroblast Primary Cells | 1.1800212 | 0.7972784 | 0.8939701 | 1.7919833 | 1.2968834 | 1.2593945 | 0.8482213 | 1.4527953 | 0.7689051 | 3.9801638 | 2.9091871 |
| Osteoblast Primary Cells | 0 | 0.8433758 | 1.1107205 | 1.9534169 | 1.6011138 | 1.4791605 | 1.2160707 | 1.1028582 | 0 | 0.9360438 | 1.9540235 |

**ROSMAP**

| cell | D0se | H2A | H3K27ac | H3K27me3 | H3K4me1 | H3K4me2 | H3K4me3 | H3K79me2 | H3K9ac | H3K9me3 | H4K20me1 |
|---|---|---|---|---|---|---|---|---|---|---|---|
| ES-I3 Cells | 0 | 0 | 0 | 1.1750061 | 1.910649 | 0 | 0.6325151 | 0 | 0.5350633 | 1.8385846 | 0 |
| ES-WA7 Cells | 0 | 0 | 0 | 1.2938378 | 2.1150569 | 0 | 0.5900269 | 0 | 0 | 1.9524337 | 0 |
| H1 Cells | 0.6113074 | 0.341994 | 0.4722422 | 1.3827912 | 1.1941536 | 0.6973351 | 0.5271435 | 0.4644448 | 0.2666759 | 0.5971589 | 1.1778545 |
| H1 BMP4 Derived Mesendoderm Cultured Cells | 0.6426192 | 0 | 0.5535856 | 1.6811877 | 1.4484121 | 0.6293491 | 0.459908 | 0.4725706 | 0.6323469 | 0 | 0 |
| H1 BMP4 Derived Trophoblast Cultured Cells | 0.6990565 | 0.6299215 | 0.4927916 | 1.0628544 | 1.0084037 | 0.4301522 | 0.7339314 | 0.4899044 | 0.1332649 | 0.7693487 | 0 |
| H1 Derived Mesenchymal Stem Cells | 1.213 | 0.6149879 | 1.3444817 | 0.5471557 | 1.8079137 | 0.9634282 | 0.3183763 | 0 | 0.5792615 | 0.6388516 | 0 |
| H1 Derived Neuro0l Progenitor Cultured Cells | 0.8706532 | 0 | 0.258585 | 1.5234095 | 1.5696759 | 0.6469307 | 0.7309172 | 0 | 0.4847089 | 2.4243376 | 0 |
| H9 Cells | 0.7706311 | 0.4719738 | 0.578736 | 0.9703974 | 1.5925311 | 0.5425709 | 0.5968606 | 0 | 0.2760359 | 0.6259684 | 1.4386018 |
| H9 Derived Neuro0l Progenitor Cultured Cells | 0 | 0.4710258 | 0 | 1.0909602 | 1.5496605 | 0 | 0.4000169 | 0 | 0 | 0 | 0 |
| H9 Derived Neuron Cultured Cells | 0 | 0.4239392 | 0 | 1.3865577 | 1.5472899 | 0 | 0.3212927 | 0 | 0 | 0.4623482 | 0 |
| hESC Derived CD184+ Endoderm Cultured Cells | 0 | 0 | 0.6455495 | 1.2101137 | 1.4641485 | 0 | 0.7373125 | 0 | 0.3152245 | 0 | 0 |
| hESC Derived CD56+ Ectoderm Cultured Cells | 0 | 0 | 0.484994 | 1.081345 | 1.3056253 | 0 | 0.6273954 | 0 | 0 | 1.3716517 | 0 |
| hESC Derived CD56+ Mesoderm Cultured Cells | 0 | 0 | 0.8979512 | 0.9134489 | 2.9813477 | 0 | 0.5880343 | 0 | 0 | 1.2492361 | 0 |
| HUES48 Cells | 0 | 0 | 0.4377583 | 1.7130187 | 1.5493777 | 0 | 0.6370295 | 0 | 0.625969 | 0.7851079 | 0 |
| HUES6 Cells | 0 | 0 | 0.6829975 | 1.5059284 | 1.4461533 | 0 | 0.5113993 | 0 | 0.8025132 | 0.6249388 | 0 |
| HUES64 Cells | 0 | 0 | 0.8401198 | 1.1422309 | 0.8892638 | 0 | 0.5548782 | 0 | 0.6599783 | 1.7116588 | 0 |
| IMR90 fetal lung fibroblasts Cell Line | 1.3300014 | 0.5594829 | 1.2828325 | 0.9012094 | 2.5243391 | 1.1633535 | 0.4436086 | 0.7886046 | 1.0294197 | 0.8776916 | 0.6462821 |
| iPS-15b Cells | 0 | 0 | 0 | 1.7775717 | 1.6472269 | 0 | 0.5137118 | 0 | 0.6135902 | 1.2945547 | 0 |
| iPS-18 Cells | 0 | 0 | 0.3037688 | 1.6883953 | 0.8075 | 0 | 0.5563837 | 0 | 0.4821826 | 0.903629 | 0 |
| iPS-20b Cells | 0 | 0 | 0.2909911 | 1.0120756 | 2.0477072 | 0 | 0.6070267 | 0 | 0.4213361 | 0.6796416 | 0 |
| iPS DF 6.9 Cells | 0.611491 | 0 | 0.6255119 | 2.1270204 | 1.3457356 | 0 | 0.5187381 | 0 | 0 | 2.5121106 | 0 |
| iPS DF 19.11 Cells | 1.1546724 | 0 | 0.4676063 | 1.3515016 | 0.9430157 | 0 | 0.5630461 | 0 | 0 | 0.6066324 | 0 |
| Mesenchymal Stem Cell Derived Adipocyte Cultured Cells | 0 | 0 | 0 | 0.6683838 | 1.1832001 | 0 | 0.3687751 | 0 | 0.485547 | 0 | 0 |
| ES-UCSF4 Cells | 0 | 0 | 0 | 1.6984595 | 1.4658993 | 0 | 0.4837591 | 0 | 0 | 1.1607386 | 0 |
| Adipose Derived Mesenchymal Stem Cell Cultured Cells | 0 | 0 | 0 | 0.531023 | 2.0963843 | 0 | 0.4820083 | 0 | 0.8423453 | 0 | 0 |
| Bone Marrow Derived Cultured Mesenchymal Stem Cells | 0 | 0 | 0.8432378 | 0.6697551 | 1.5679902 | 0 | 0.560102 | 0 | 0.7457064 | 0 | 0 |
| Breast Myoepithelial Primary Cells | 0 | 0 | 0 | 0.8679997 | 1.3438214 | 0 | 0.5265315 | 0 | 0.780666 | 0.4401406 | 0 |
| Breast variant Human Mammary Epithelial Cells (vHMEC) | 1.1734942 | 0 | 0 | 0.5395148 | 2.2231403 | 0 | 0.2936825 | 0 | 0 | 1.109957 | 0 |
| Primary monocytes from peripheral blood | 0.9393311 | 0 | 1.3035126 | 1.3288483 | 1.7561954 | 0 | 0.4849564 | 0 | 0 | 0 | 0 |
| Primary neutrophils from peripheral blood | 0 | 0 | 0 | 1.2915475 | 1.0052506 | 0 | 0.7084978 | 0 | 0 | 0 | 0 |
| Primary B cells from cord blood | 0 | 0 | 0 | 1.8199307 | 1.0017986 | 0 | 0.6279402 | 0 | 0 | 0 | 0 |
| Primary B cells from peripheral blood | 0.9038775 | 0 | 0.5139857 | 0.9966157 | 0.8435068 | 0 | 0.458982 | 0 | 0 | 0 | 0 |
| Primary T cells from cord blood | 0.9623316 | 0 | 0 | 1.570324 | 1.2307487 | 0 | 0.4378916 | 0 | 0 | 0 | 0 |
| Primary T cells from peripheral blood | 0.6379143 | 0 | 0.353143 | 1.2351065 | 0.6064868 | 0 | 0.2641075 | 0 | 0 | 2.4008961 | 0 |
| Primary hematopoietic stem cells | 0 | 0 | 0 | 0.4140591 | 1.2369774 | 0 | 0.3791015 | 0 | 0 | 0 | 0 |
| Primary hematopoietic stem cells short term culture | 0 | 0 | 0 | 1.8751754 | 1.2877255 | 0 | 0.3033453 | 0 | 0 | 0.3782964 | 0 |
| Primary T helper memory cells from peripheral blood 2 | 0 | 0 | 0.5613764 | 0.4433999 | 0.6985496 | 0 | 0.287268 | 0 | 0 | 0 | 0 |
| Primary T helper 0ive cells from peripheral blood 1 | 0 | 0 | 0.1712022 | 0.3412606 | 0.429449 | 0 | 0.2752592 | 0 | 0.3600438 | 0 | 0 |
| Primary T helper 0ive cells from peripheral blood 2 | 0 | 0 | 0.1425871 | 1.2987927 | 0.7526962 | 0 | 0.3596838 | 0 | 0 | 0 | 0 |
| Primary T helper memory cells from peripheral blood 1 | 0 | 0 | 0.2494531 | 0.7086068 | 0.8363558 | 0 | 0.2973813 | 0 | 0 | 0 | 0 |
| Primary T helper cells PMA-I stimulated | 0 | 0 | 0.4494523 | 0.881555 | 0.4061432 | 0 | 0.370828 | 0 | 0 | 0 | 0 |
| Primary T helper 17 cells PMA-I stimulated | 0 | 0 | 0.2312384 | 1.0159057 | 0.8016217 | 0 | 0.2473095 | 0 | 0 | 0 | 0 |
| Primary T helper cells from peripheral blood | 0 | 0 | 0.3971857 | 1.4908513 | 0.4880039 | 0 | 0.2940039 | 0 | 0 | 0 | 0 |
| Primary T regulatory cells from peripheral blood | 0 | 0 | 0.1581618 | 0.9787324 | 0.7119688 | 0 | 0.2858297 | 0 | 0 | 0 | 0 |
| Primary T cells effector/memory enriched from peripheral blood | 0 | 0 | 0.2391231 | 0.5794306 | 0.6093999 | 0 | 0.2439932 | 0 | 0 | 0 | 0 |
| Primary 0tural Killer cells from peripheral blood | 1.0073263 | 0 | 0.4584718 | 1.2045337 | 1.0191875 | 0 | 0.4696501 | 0 | 0 | 0.5780768 | 0 |
| Primary T CD8+ 0ive cells from peripheral blood | 0 | 0 | 0.0857486 | 0.8363017 | 0.6056649 | 0 | 0.2015468 | 0 | 0.1837472 | 0 | 0 |
| Primary T CD8+ memory cells from peripheral blood | 0 | 0 | 0.5334554 | 0.4170328 | 0.6228233 | 0 | 0.2929603 | 0 | 0 | 0 | 0 |
| Mesenchymal Stem Cell Derived Chondrocyte Cultured Cells | 0 | 0 | 1.0150839 | 0.2885652 | 1.5105278 | 0 | 0.672979 | 0 | 0.7475677 | 2.0030249 | 0 |
| Primary hematopoietic stem cells G-CSF-mobilized Female | 1.2148133 | 0 | 0.4042335 | 0.9964037 | 1.4022132 | 0 | 0.6150913 | 0 | 0 | 0 | 0 |
| Primary hematopoietic stem cells G-CSF-mobilized Male | 0.8062475 | 0 | 0 | 0.9987243 | 1.5801451 | 0 | 0.5497455 | 0 | 0 | 0 | 0 |
| Muscle Satellite Cultured Cells | 0 | 0 | 0 | 1.6836435 | 1.5104508 | 0.8746166 | 0.5619934 | 0 | 0.8184167 | 0 | 0 |
| Cortex derived primary cultured neurospheres | 0 | 0 | 0 | 2.2166347 | 1.148658 | 0 | 0.4862661 | 0 | 0 | 0.7436199 | 0 |
| Ganglion Eminence derived primary cultured neurospheres | 0 | 0 | 0 | 1.312856 | 0.9649611 | 0 | 0.4979509 | 0 | 0 | 0 | 0 |
| Foreskin Fibroblast Primary Cells skin01 | 1.4027565 | 0 | 0.6747455 | 0.7509293 | 1.691744 | 0 | 0.6609863 | 0 | 0 | 1.1339216 | 0 |
| Foreskin Fibroblast Primary Cells skin02 | 1.5355409 | 0 | 0.6785806 | 0.7521939 | 1.2816028 | 0 | 0.5761733 | 0 | 0 | 1.6082816 | 0 |
| Foreskin Keratinocyte Primary Cells skin02 | 1.3535868 | 0 | 0 | 0.4408439 | 1.4460868 | 0 | 0.6178977 | 0 | 0 | 0.8710003 | 0 |
| Foreskin Keratinocyte Primary Cells skin03 | 0 | 0 | 0.6984169 | 0.7743079 | 1.4642522 | 0 | 0.5025455 | 0 | 0 | 0.4142422 | 0 |
| Foreskin Melanocyte Primary Cells skin01 | 1.3095198 | 0 | 0.4308681 | 1.0309742 | 2.3860747 | 0 | 0.6406266 | 0 | 0 | 1.2795668 | 0 |
| Foreskin Melanocyte Primary Cells skin03 | 0 | 0 | 0.625805 | 1.1434463 | 1.663265 | 0 | 0.4838215 | 0 | 0 | 0.742255 | 0 |
| Primary mononuclear cells from peripheral blood | 0 | 0 | 0.4354683 | 0.9601976 | 0.5358968 | 0 | 0.481436 | 0 | 0.6495524 | 0 | 0 |
| Adipose Nuclei | 0 | 0 | 0.5628457 | 1.0422127 | 1.0973468 | 0 | 0.5613932 | 0 | 0.6213977 | 0 | 0 |
| Aorta | 0 | 0 | 0.3393446 | 1.3464743 | 1.9935595 | 0 | 0.3874491 | 0 | 0 | 0 | 0 |
| Liver | 0 | 0 | 0.8180801 | 1.1462869 | 0.6593848 | 0 | 0.3863947 | 0 | 0.5656632 | 0 | 0 |
| Brain Angular Gyrus | 0 | 0 | 0.9770314 | 1.296418 | 1.5148948 | 0 | 0.6134802 | 0 | 0.9501227 | 0 | 0 |
| Brain Anterior Caudate | 0 | 0 | 1.0351818 | 0.7941998 | 1.6353155 | 0 | 0.8000444 | 0 | 0.6657573 | 0 | 0 |
| Brain Cingulate Gyrus | 0 | 0 | 0.9374253 | 1.5647299 | 1.2751462 | 0 | 0.6555353 | 0 | 0.9985911 | 0 | 0 |
| Brain Germi0l Matrix | 0 | 0 | 0 | 1.1375647 | 1.0609182 | 0 | 0.5382897 | 0 | 0 | 0.6682995 | 0 |
| Brain Hippocampus Middle | 0 | 0 | 1.1858397 | 1.4306537 | 1.5894873 | 0 | 0.890056 | 0 | 0 | 0 | 0 |
| Brain Inferior Temporal Lobe | 0 | 0 | 0.9005168 | 1.4334283 | 1.8470114 | 0 | 0.7409734 | 0 | 0.7206193 | 1.7177872 | 0 |
| Brain_Dorsolateral_Prefrontal_Cortex | 0 | 0 | 1.0009915 | 1.0120501 | 1.5729499 | 0 | 0.7421976 | 0 | 0.885953 | 1.5171121 | 0 |
| Brain Substantia Nigra | 0 | 0 | 0.9312268 | 0.7639404 | 1.7142927 | 0 | 0.7222543 | 0 | 0.8195423 | 0 | 0 |
| Colonic Mucosa | 0 | 0 | 1.0918816 | 1.1310967 | 1.1038663 | 0 | 0.7149517 | 0 | 0.4167803 | 0 | 0 |
| Colon Smooth Muscle | 0 | 0 | 1.0873458 | 0.7486282 | 1.7192786 | 0 | 0.7090076 | 0 | 0.6484446 | 0 | 0 |
| Duodenum Mucosa | 0 | 0 | 0 | 2.0866722 | 1.4515692 | 0 | 0.8119212 | 0 | 0.6508571 | 0 | 0 |
| Duodenum Smooth Muscle | 0 | 0 | 1.2452167 | 1.4460718 | 1.4178611 | 0 | 0.8133381 | 0 | 0 | 0 | 0 |
| Esophagus | 0 | 0 | 0.9450594 | 1.0699313 | 1.1363727 | 0 | 0.4237157 | 0 | 0 | 0.4429732 | 0 |
| Fetal Adre0l Gland | 0.9557028 | 0 | 1.175584 | 1.1908424 | 1.8243389 | 0 | 0.5907879 | 0 | 0 | 0.4452114 | 0 |
| Fetal Brain Male | 0.4731143 | 0 | 0 | 1.4039081 | 0.9701929 | 0 | 0.4777266 | 0 | 0 | 0.4784162 | 0 |
| Fetal Brain Female | 0.3289311 | 0 | 0 | 1.1238496 | 1.1635507 | 0 | 0.6025794 | 0 | 0 | 1.3830283 | 0 |
| Fetal Heart | 0.8918624 | 0 | 0 | 1.3039839 | 3.2382066 | 0 | 1.0902815 | 0 | 1.1811435 | 0.5659863 | 0 |
| Fetal Intestine Large | 0.5431858 | 0 | 0.5753109 | 1.9113063 | 1.1509153 | 0 | 0.5011342 | 0 | 0 | 0.7943386 | 0 |
| Fetal Intestine Small | 0.533483 | 0 | 0.6270243 | 2.0545021 | 1.2991288 | 0 | 0.563186 | 0 | 0 | 0.7908691 | 0 |
| Fetal Kidney | 1.0329082 | 0 | 0 | 0.5844677 | 2.5394586 | 0 | 0.4303293 | 0 | 0.3100218 | 0 | 0 |
| Pancreatic Islets | 0 | 0 | 0.4818914 | 1.3030351 | 3.0072456 | 0 | 0.4980352 | 0 | 0.396658 | 0 | 0 |
| Fetal Lung | 1.2354823 | 0 | 0 | 1.3618202 | 1.4366687 | 0 | 0.5687294 | 0 | 0.773264 | 0 | 0 |
| Fetal Muscle Trunk | 1.0086317 | 0 | 0.5983252 | 1.1933552 | 1.523237 | 0 | 0.4582807 | 0 | 0 | 0 | 0 |
| Fetal Muscle Leg | 0.7938888 | 0 | 0.3064811 | 1.1457231 | 1.2514412 | 0 | 0.5155713 | 0 | 0 | 1.3742508 | 0 |
| Placenta | 0.5583154 | 0 | 0.4658464 | 0.6876161 | 1.075919 | 0 | 0.2644821 | 0 | 0 | 0 | 0 |
| Fetal Stomach | 1.1385957 | 0 | 1.006661 | 0.8156432 | 1.4640117 | 0 | 0.655474 | 0 | 0 | 1.1212723 | 0 |
| Fetal Thymus | 0.470598 | 0 | 0.5009019 | 1.6732306 | 1.1510451 | 0 | 0.4983597 | 0 | 0 | 0.496319 | 0 |
| Gastric | 0.6447541 | 0 | 0.4754487 | 1.2674303 | 1.918896 | 0 | 0.5441504 | 0 | 0 | 0.4742556 | 0 |
| Left Ventricle | 0 | 0 | 0.7661627 | 1.0833766 | 2.3363243 | 0 | 0.5577677 | 0 | 0 | 1.1405426 | 0 |
| Lung | 0 | 0 | 1.1864602 | 1.129741 | 1.6793523 | 0 | 0.694614 | 0 | 0 | 0.5800576 | 0 |
| Ovary | 0.8774715 | 0 | 0.7349956 | 0.7665788 | 1.9443673 | 0 | 0.529846 | 0 | 0 | 1.2566061 | 0 |
| Pancreas | 1.1268368 | 0 | 0.2524497 | 0 | 1.1999815 | 0 | 0.3589158 | 0 | 0 | 1.0907082 | 0 |
| Placenta Amnion | 0 | 0 | 0.3812145 | 0.779936 | 0.7676837 | 0 | 0.4253548 | 0 | 0 | 2.5278334 | 0 |
| Psoas Muscle | 0.6581224 | 0 | 0.5251802 | 0.422913 | 1.2734053 | 0 | 0.5135807 | 0 | 0 | 0 | 0 |
| Rectal Mucosa Donor 29 | 0 | 0 | 0.9801163 | 1.1790097 | 1.2661831 | 0 | 0.7617676 | 0 | 0.8123134 | 0 | 0 |
| Rectal Mucosa Donor 31 | 0 | 0 | 0.9849839 | 2.4395973 | 1.2878928 | 0 | 0.4554675 | 0 | 0.5946458 | 0 | 0 |
| Rectal Smooth Muscle | 0 | 0 | 0.6949723 | 0.6428956 | 2.0538701 | 0 | 0.6302845 | 0 | 0.473236 | 0 | 0 |
| Right Atrium | 0 | 0 | 0.846307 | 8.8792025 | 2.9424292 | 0 | 0.6624733 | 0 | 0 | 0 | 0 |
| Right Ventricle | 0 | 0 | 0.8740936 | 0.9940739 | 2.4536911 | 0 | 0.5876215 | 0 | 0 | 0 | 0 |
| Sigmoid Colon | 0 | 0 | 1.1441948 | 1.5035034 | 0.9876084 | 0 | 0.3732995 | 0 | 0 | 0 | 0 |
| Skeletal Muscle Male | 0 | 0 | 0 | 1.6060688 | 1.4593364 | 0 | 0.604464 | 0 | 0.6152663 | 1.0870658 | 0 |
| Skeletal Muscle Female | 0 | 0 | 0.8599635 | 1.1588828 | 1.3629218 | 0 | 0.4869172 | 0 | 0.4709915 | 0 | 0 |
| Small Intestine | 0.4349024 | 0 | 1.2329074 | 0.921776 | 1.980802 | 0 | 0.2079779 | 0 | 0 | 0 | 0 |
| Stomach Mucosa | 0 | 0 | 0 | 1.6073953 | 1.4785467 | 0 | 0.1985327 | 0 | 0.6697711 | 0 | 0 |
| Stomach Smooth Muscle | 0 | 0 | 0.8050653 | 1.2007878 | 1.5701712 | 0 | 0.7706043 | 0 | 0.8825163 | 0 | 0 |
| Thymus | 0 | 0 | 0.3833766 | 1.5502791 | 0.1143654 | 0 | 0.3155152 | 0 | 0 | 0 | 0 |
| Spleen | 0 | 0 | 0.9304265 | 0.2706472 | 1.3093425 | 0 | 0.5424409 | 0 | 0 | 0.7398543 | 0 |
| A549 EtOH 0.02pct Lung Carcinoma Cell Line | 0.8502463 | 0.4249275 | 0.455503 | 1.6794824 | 1.0896374 | 0.5879881 | 0.4053137 | 0.3966028 | 0.5352355 | 0 | 1.7986316 |
| Dnd41 TCell Leukemia Cell Line | 0 | 0.0858236 | 0.264963 | 2.9952586 | 0.5163834 | 0.0680477 | 0.0773679 | 0.1100551 | 0.2445833 | 0 | 1.1585334 |
| GM12878 Lymphoblastoid Cells | 1.1013649 | 0.2149363 | 0.6921584 | 0.6973616 | 2.0115702 | 0.7712449 | 0.3748991 | 0.6453637 | 0.323806 | 1.37308 | 2.3190233 |
| HeLa-S3 Cervical Carcinoma Cell Line | 1.0896775 | 0.8081858 | 0.8362847 | 3.1431696 | 1.6569144 | 0.8635756 | 0.7288592 | 0.4494045 | 0.7638541 | 2.9135839 | 0.4620914 |
| HepG2 Hepatocellular Carcinoma Cell Line | 0.9494246 | 0.1767604 | 0.4757725 | 0.2627955 | 1.2258705 | 0.7572689 | 0.6168433 | 0.7507927 | 0.5934011 | 0 | 0.9247184 |
| HMEC Mammary Epithelial Primary Cells | 1.0149723 | 0.6375453 | 0.7497952 | 0.6222899 | 1.4830345 | 0.9573975 | 0.3134653 | 0.5105229 | 0.8398566 | 0.8076886 | 4.2547974 |
| HSMM Skeletal Muscle Myoblasts Cells | 1.1631458 | 0.2970841 | 0.5807157 | 0.923716 | 2.3803113 | 0.6927646 | 0.5944134 | 1.1205902 | 0.6879709 | 0 | 4.0931022 |
| HSMM cell derived Skeletal Muscle Myotubes Cells | 1.0340612 | 0.7720396 | 0.8453933 | 1.567985 | 2.3921212 | 0.9587712 | 0.6054499 | 0.3048328 | 0.6370279 | 0 | 2.1521531 |
| HUVEC Umbilical Vein Endothelial Primary Cells | 0.7116927 | 0.7495513 | 0.4352349 | 1.8318219 | 1.2019343 | 0.6651893 | 0.3204726 | 0.3335872 | 0.450726 | 1.320785 | 0 |
| K562 Leukemia Cells | 0.2910802 | 0.5518281 | 0.1688139 | 0.3158464 | 1.1198362 | 0.3161355 | 0.2851453 | 0.4156287 | 0.3375077 | 0 | 0 |
| Monocytes-CD14+ RO01746 Primary Cells | 1.2486535 | 0.8665824 | 1.1774607 | 1.2389622 | 1.3362494 | 0.8740248 | 0.9603401 | 0.6462416 | 0.5925125 | 0.6635129 | 1.9157129 |
| NH-A Astrocytes Primary Cells | 1.4430566 | 0.5784367 | 0.7650017 | 0.7185672 | 1.6192763 | 0.9755005 | 0.6304669 | 0.6743066 | 0.7752875 | 1.8825865 | 0.7313857 |
| NHDF-Ad Adult Dermal Fibroblast Primary Cells | 0.9810796 | 0.6918334 | 0.5049783 | 0.7484547 | 2.7392645 | 0.5307294 | 0.5486837 | 0.6200082 | 0.535994 | 0 | 2.1739756 |
| NHEK-Epidermal Keratinocyte Primary Cells | 1.2034148 | 0.4956914 | 0.9423724 | 0.7681728 | 1.7257667 | 1.1568889 | 0.4480078 | 0.7028685 | 0.8793432 | 0.6712473 | 0.6357287 |
| NHLF Lung Fibroblast Primary Cells | 0.9418067 | 0.8252589 | 0.9162817 | 0.8703109 | 2.423334 | 1.0452032 | 0.4234906 | 0.6421225 | 0.5657646 | 0 | 0 |
| Osteoblast Primary Cells | 0 | 0.5433293 | 0.8344708 | 0.4331554 | 1.7653861 | 0.6891051 | 0.5391013 | 0.5076041 | 0 | 0 | 1.2776177 |

Table notes：AD: Alzheimer's disease; ADNI: AD Neuroimaging Initiative; ROSMAP: Religious Orders Study and Rush Memory and Aging Project.
