# Supplementary material for: Genetic and pathway complexity in Alzheimer’s disease: Insights from multi-omic data about the immune response and mitochondrial function
Source: Neural Regen Res. 2025 Sep 29;21(9):4406–23. doi: 10.4103/NRR.NRR-D-25-00184 (PMC13557549; doi:10.4103/NRR.NRR-D-25-00184)
Supplement: Supplementary file 6 [file NRR-21-4406_Suppl14.pdf]

Additional Table 14 Tissue-specific annotation of important CpGs loci

ADNI					
probe	hyper	hypo	dif	min_tissue	max_tissue
cg00179070	0.441	0.092	0.349	fallopian_tube	skin
cg14005211	0.465	0.266	0.2	cartilage	adrenal_gland
cg09404376	0.452	0.318	0.134	endometrium	thyroid
cg18544564	0.477	0.387	0.091	cartilage	brain-cerebellum
cg09936561	0.524	0.157	0.367	brain-cerebellum	small_intestine
cg09211763	0.513	0.102	0.411	cartilage	small_intestine
cg05618426	0.433	0.328	0.105	testis	brain-cerebellum
cg06499030	0.21	0.237	0.027	stomach	bone_marrow
cg05262549	0.368	0.524	0.156	brain-cerebellum	cervix
cg22132982	0.308	0.346	0.038	cartilage	brain-cerebellum
cg04069951	0.259	0.164	0.096	pituitary	pancreas
cg13411522	0.241	0.488	0.247	cartilage	blood
cg21490635	0.531	0.393	0.138	cartilage	brain-cerebellum
cg00000321	0.451	0.211	0.24	cartilage	brain-cerebrum
cg27255672	0.16	0.802	0.642	brain-cerebellum	fallopian_tube
cg09473826	0.291	0.369	0.078	adrenal_gland	brain-cerebrum
cg02482042	0.297	0.277	0.02	thyroid	brain-cerebrum
cg07472953	0.406	0.436	0.03	skeletal_muscle	brain-cerebellum
cg24899315	0.259	0.097	0.162	salivary_gland	colon
cg15718287	0.327	0.535	0.208	small_intestine	brain-cerebellum
cg00499277	0.416	0.256	0.159	cartilage	brain-cerebellum
cg09559780	0.462	0.199	0.263	cartilage	bone_marrow
cg14244439	0.414	0.413	0.001	small_intestine	cartilage
cg16645423	0.257	0.404	0.147	brain-cerebellum	fallopian_tube
cg00953256	0.311	0.433	0.122	prostate	bone_marrow
cg15324756	0.925	0.026	0.899	fallopian_tube	adrenal_gland
cg12325605	0.239	0.684	0.445	brain-cerebellum	endometrium
cg21567371	0.044	0.66	0.616	skin	fallopian_tube
cg09618904	0.519	0.356	0.163	cartilage	blood
cg10434653	0.494	0.008	0.485	subcutaneous_adipose	small_intestine
cg10591607	0.64	0.251	0.389	brain-cerebellum	small_intestine
cg03176423	0.366	0.129	0.237	endometrium	blood
cg16402547	0.199	0.258	0.059	brain-cerebellum	blood
cg12623364	0.291	0.489	0.197	blood	fallopian_tube
cg09568217	0.208	0.492	0.284	small_intestine	brain-cerebellum
cg00138126	0.231	0.571	0.34	cartilage	fallopian_tube
cg07612655	0.412	0.322	0.09	spleen	brain-cerebellum
cg09699787	0.647	0.259	0.387	endometrium	brain-cerebellum
cg10324998	0.663	0.186	0.477	endometrium	brain-cerebellum
cg06545761	0.304	0.564	0.26	brain-cerebellum	liver

ROSMAP					
probe	hyper	hypo	dif	min_tissue	max_tissue
cg09595163	0.836	0.065	0.772	brain-cerebellum	liver
cg09520393	0.465	0.077	0.388	bone_marrow	small_intestine
cg01802545	0.769	0.225	0.544	blood	brain-cerebellum
cg15505615	0.235	0.413	0.178	cartilage	thyroid
cg15901783	0.549	0.333	0.216	cartilage	blood
cg08300930	0.18	0.548	0.368	subcutaneous_adipose	blood
cg09079275	0.726	0.062	0.664	brain-cerebellum	liver
cg11435826	0.36	0.559	0.199	bone_marrow	brain-cerebellum
cg04193923	0.439	0.409	0.029	skeletal_muscle	brain-cerebellum
cg10426370	0.32	0.445	0.125	skeletal_muscle	small_intestine
cg22904711	0.49	0.386	0.104	brain-cerebellum	blood
cg17693222	0.048	0.37	0.322	testis	thyroid
cg13491071	0.483	0.25	0.234	cartilage	brain-cerebrum
cg15343510	0.166	0.322	0.156	lung	fallopian_tube
cg08959039	0.349	0.397	0.048	spleen	brain-cerebellum
cg12699313	0.023	0.805	0.783	prostate	blood
cg06285439	0.297	0.56	0.263	esophagus	brain-cerebellum
cg12325605	0.239	0.684	0.445	brain-cerebellum	endometrium
cg17913386	0.491	0.08	0.411	endometrium	stomach
cg14622549	0.039	0.792	0.753	spleen	fallopian_tube
cg00094412	0.335	0.456	0.121	brain-cerebellum	adrenal_gland
cg24351901	0.352	0.161	0.191	cartilage	endometrium
cg25384595	0.26	0.565	0.305	bone_marrow	brain-cerebellum

Table notes：CpGs: Cytosine-phosphate-Guanine dinucleotides
