# Supplementary material for: Genetic and pathway complexity in Alzheimer’s disease: Insights from multi-omic data about the immune response and mitochondrial function
Source: Neural Regen Res. 2025 Sep 29;21(9):4406–23. doi: 10.4103/NRR.NRR-D-25-00184 (PMC13557549; doi:10.4103/NRR.NRR-D-25-00184)
Supplement: Supplementary file 7 [file NRR-21-4406_Suppl15.pdf]

Additional Table 15 Relationship between methylation levels of important CpGs loci and nearby gene expression

ADNI

probe	gene	region	r	p	tissue	source	gene_symbol
cg10324998	ENSG00000069702	body	0.259	3.30E-10	brain	TCGA-GBM,TCGA-LGG	TGFBR3
cg09473826	ENSG00000116147	body	0.371	5.04E-20	brain	TCGA-GBM,TCGA-LGG	TNR
cg11341981	ENSG00000163485	body	-0.48	1.69E-34	brain	TCGA-GBM,TCGA-LGG	ADORA1
cg09699787	ENSG00000130508	body	-0.16	9.52E-05	brain	TCGA-GBM,TCGA-LGG	PXDN
cg14005211	ENSG00000128683	body	-0.36	3.28E-19	brain	TCGA-GBM,TCGA-LGG	GAD1
cg18544564	ENSG00000168542	body	-0.47	3.55E-32	brain	TCGA-GBM,TCGA-LGG	COL3A1
cg09211763	ENSG00000204262	body	-0.65	2.95E-68	brain	TCGA-GBM,TCGA-LGG	COL5A2
cg02482042	ENSG00000157087	body	0.308	5.59E-14	brain	TCGA-GBM,TCGA-LGG	ATP2B2
cg22132982	ENSG00000154764	body	0.382	3.16E-21	brain	TCGA-GBM,TCGA-LGG	WNT7A
cg24899315	ENSG00000114646	body	0.149	0.0003722	brain	TCGA-GBM,TCGA-LGG	CSPG5
cg12325605	ENSG00000163947	body	-0.3	5.93E-13	brain	TCGA-GBM,TCGA-LGG	ARHGEF3
cg04376312	ENSG00000065534	body	-0.25	2.28E-09	brain	TCGA-GBM,TCGA-LGG	MYLK
cg09936561	ENSG00000169676	body	-0.2	2.13E-06	brain	TCGA-GBM,TCGA-LGG	DRD5
cg24279243	ENSG00000109787	body	-0.14	0.0011282	brain	TCGA-GBM,TCGA-LGG	KLF3
cg12623364	ENSG00000138795	body	0.19	4.12E-06	brain	TCGA-GBM,TCGA-LGG	LEF1
cg25403205	ENSG00000204472	body	-0.51	4.79E-39	brain	TCGA-GBM,TCGA-LGG	AIF1
cg06499030	ENSG00000232629	promoter	-0.11	0.0089007	brain	TCGA-GBM,TCGA-LGG	HLA-DQB2
cg10591607	ENSG00000164418	body	-0.25	8.78E-10	brain	TCGA-GBM,TCGA-LGG	GRIK2
cg02196834	ENSG00000081087	body	-0.34	3.03E-17	brain	TCGA-GBM,TCGA-LGG	OSTM1
cg14244439	ENSG00000169071	body	-0.19	9.27E-06	brain	TCGA-GBM,TCGA-LGG	ROR2
cg04069951	ENSG00000238184	body	0.614	2.36E-60	brain	TCGA-GBM,TCGA-LGG	AC129929.5
cg04069951	ENSG00000110651	body	0.149	0.00035638	brain	TCGA-GBM,TCGA-LGG	CD81
cg00953256	ENSG00000110092	body	-0.21	4.90E-07	brain	TCGA-GBM,TCGA-LGG	CCND1
cg21490635	ENSG00000134531	promoter	-0.26	6.88E-10	brain	TCGA-GBM,TCGA-LGG	EMP1
cg02962597	ENSG00000089250	body	0.351	6.49E-18	brain	TCGA-GBM,TCGA-LGG	NOS1
cg25759064	ENSG00000102606	body	-0.3	9.28E-20	brain	TCGA-GBM,TCGA-LGG	ARHGEF7
cg00499277	ENSG00000197616	promoter	0.346	1.92E-17	brain	TCGA-GBM,TCGA-LGG	MYH6
cg10434653	ENSG00000140632	promoter	-0.1	0.00137977	brain	TCGA-GBM,TCGA-LGG	GLYR1
cg16645423	ENSG00000188322	body	0.24	7.10E-09	brain	TCGA-GBM,TCGA-LGG	SBK1
cg09559780	ENSG00000167207	promoter	-0.2	3.11E-07	brain	TCGA-GBM,TCGA-LGG	NOD2
cg06545761	ENSG00000140948	body	0.355	2.34E-18	brain	TCGA-GBM,TCGA-LGG	ZCCHC14
cg09568217	ENSG00000169992	body	0.378	9.46E-21	brain	TCGA-GBM,TCGA-LGG	NLGN2
cg07472953	ENSG00000108342	promoter	-0.1	0.00018686	brain	TCGA-GBM,TCGA-LGG	CSF3
cg11321181	ENSG00000130477	body	0.341	6.08E-17	brain	TCGA-GBM,TCGA-LGG	UNC13A
cg02149446	ENSG00000197405	body	-0.2	1.37E-09	brain	TCGA-GBM,TCGA-LGG	C5AR1
cg15659599	ENSG00000239998	body	0.167	6.12E-05	brain	TCGA-GBM,TCGA-LGG	LILRA2
cg09618904	ENSG00000125965	body	-0.1	0.00240113	brain	TCGA-GBM,TCGA-LGG	GDF5
cg23480341	ENSG00000124107	promoter	-0.1	0.00245986	brain	TCGA-GBM,TCGA-LGG	SLPI
cg00138126	ENSG00000278709	body	-0.3	7.09E-13	brain	TCGA-GBM,TCGA-LGG	RP5-1059L7.1
cg13411522	ENSG00000092758	body	-0.6	6.53E-58	brain	TCGA-GBM,TCGA-LGG	COL9A3
cg16402547	ENSG00000075043	promoter	0.363	3.96E-19	brain	TCGA-GBM,TCGA-LGG	KCNQ2
cg05262549	ENSG00000157064	body	0.183	0.00538497	colon	TCGA-COAD	NMNAT2
cg11341981	ENSG00000163485	body	0.235	0.00032543	colon	TCGA-COAD	ADORA1
cg09699787	ENSG00000130508	body	0.276	2.35E-05	colon	TCGA-COAD	PXDN
cg06968859	ENSG00000066032	body	0.269	3.63E-05	colon	TCGA-COAD	CTNNA2
cg14005211	ENSG00000128683	body	0.447	1.24E-12	colon	TCGA-COAD	GAD1
cg18544564	ENSG00000168542	body	0.415	6.34E-11	colon	TCGA-COAD	COL3A1
cg02482042	ENSG00000157087	body	0.234	0.00036524	colon	TCGA-COAD	ATP2B2
cg12325605	ENSG00000163947	body	-0.19	0.00339574	colon	TCGA-COAD	ARHGEF3
cg04376312	ENSG00000065534	body	-0.24	0.00029474	colon	TCGA-COAD	MYLK
cg05618426	ENSG00000151617	promoter	-0.39	1.15E-09	colon	TCGA-COAD	EDNRA
cg25403205	ENSG00000204472	body	-0.3	3.44E-06	colon	TCGA-COAD	AIF1
cg25764570	ENSG00000204287	promoter	0.244	0.00018778	colon	TCGA-COAD	HLA-DRA
cg06204447	ENSG00000196126	body	0.208	0.00157095	colon	TCGA-COAD	HLA-DRB1
cg06499030	ENSG00000232629	promoter	0.277	2.10E-05	colon	TCGA-COAD	HLA-DQB2
cg09404376	ENSG00000118515	body	0.428	1.33E-11	colon	TCGA-COAD	SGK1
cg14244439	ENSG00000169071	body	0.459	2.44E-13	colon	TCGA-COAD	ROR2
cg00953256	ENSG00000110092	body	-0.25	0.0001209	colon	TCGA-COAD	CCND1
cg00953256	ENSG00000149716	body	-0.23	0.00036762	colon	TCGA-COAD	ORAOV1
cg27255672	ENSG00000149294	body	0.249	0.00014037	colon	TCGA-COAD	NCAM1
cg21490635	ENSG00000134531	promoter	0.225	0.00059917	colon	TCGA-COAD	EMP1
cg15718287	ENSG00000133083	body	0.61	1.01E-24	colon	TCGA-COAD	DCLK1
cg25759064	ENSG00000102606	body	-0.27	3.10E-05	colon	TCGA-COAD	ARHGEF7
cg17147440	ENSG00000140416	body	-0.33	3.10E-07	colon	TCGA-COAD	TPM1
cg16645423	ENSG00000188322	body	0.3	3.68E-06	colon	TCGA-COAD	SBK1
cg09559780	ENSG00000167207	promoter	-0.3	3.62E-06	colon	TCGA-COAD	NOD2
cg08823411	ENSG00000171246	body	0.178	0.00676569	colon	TCGA-COAD	NPTX1
cg02149446	ENSG00000197405	body	0.231	0.00041767	colon	TCGA-COAD	C5AR1
cg15659599	ENSG00000239998	body	0.438	3.69E-12	colon	TCGA-COAD	LILRA2
cg23480341	ENSG00000124107	promoter	-0.2	0.0011248	colon	TCGA-COAD	SLPI
cg13411522	ENSG00000092758	body	-0.2	9.88E-06	colon	TCGA-COAD	COL9A3
cg10324998	ENSG00000069702	body	-0.1	0.00012312	kidney	TCGA-KIRP,TCGA-KIRC,TCGA-KICH	TGFBR3
cg09473826	ENSG00000116147	body	0.169	1.54E-05	kidney	TCGA-KIRP,TCGA-KIRC,TCGA-KICH	TNR
cg05262549	ENSG00000157064	body	-0.3	3.42E-16	kidney	TCGA-KIRP,TCGA-KIRC,TCGA-KICH	NMNAT2
cg09699787	ENSG00000130508	body	-0.2	1.92E-07	kidney	TCGA-KIRP,TCGA-KIRC,TCGA-KICH	PXDN
cg14005211	ENSG00000128683	body	0.134	0.00058692	kidney	TCGA-KIRP,TCGA-KIRC,TCGA-KICH	GAD1
cg18544564	ENSG00000168542	body	-0.3	4.99E-21	kidney	TCGA-KIRP,TCGA-KIRC,TCGA-KICH	COL3A1
cg09211763	ENSG00000204262	body	-0.1	0.0003542	kidney	TCGA-KIRP,TCGA-KIRC,TCGA-KICH	COL5A2
cg02482042	ENSG00000157087	body	0.218	1.89E-08	kidney	TCGA-KIRP,TCGA-KIRC,TCGA-KICH	ATP2B2
cg22132982	ENSG00000154764	body	0.174	8.15E-06	kidney	TCGA-KIRP,TCGA-KIRC,TCGA-KICH	WNT7A
cg24899315	ENSG00000114646	body	0.219	1.77E-08	kidney	TCGA-KIRP,TCGA-KIRC,TCGA-KICH	CSPG5
cg21213853	ENSG00000164082	body	-0.3	7.90E-22	kidney	TCGA-KIRP,TCGA-KIRC,TCGA-KICH	GRM2
cg12325605	ENSG00000163947	body	-0.1	0.00242212	kidney	TCGA-KIRP,TCGA-KIRC,TCGA-KICH	ARHGEF3
cg04376312	ENSG00000065534	body	-0.3	4.57E-17	kidney	TCGA-KIRP,TCGA-KIRC,TCGA-KICH	MYLK
cg09936561	ENSG00000109667	body	0.221	1.17E-08	kidney	TCGA-KIRP,TCGA-KIRC,TCGA-KICH	SLC2A9
cg09936561	ENSG00000169676	body	0.144	0.00023146	kidney	TCGA-KIRP,TCGA-KIRC,TCGA-KICH	DRD5
cg12623364	ENSG00000138795	body	-0.2	3.06E-09	kidney	TCGA-KIRP,TCGA-KIRC,TCGA-KICH	LEF1
cg05618426	ENSG00000151617	promoter	-0.2	5.45E-11	kidney	TCGA-KIRP,TCGA-KIRC,TCGA-KICH	EDNRA
cg03176423	ENSG00000204642	body	-0.1	0.00331005	kidney	TCGA-KIRP,TCGA-KIRC,TCGA-KICH	HLA-F
cg25403205	ENSG00000204472	body	-0.5	5.35E-48	kidney	TCGA-KIRP,TCGA-KIRC,TCGA-KICH	AIF1
cg10591607	ENSG00000164418	body	0.139	0.00038525	kidney	TCGA-KIRP,TCGA-KIRC,TCGA-KICH	GRIK2
cg02196834	ENSG00000081087	body	0.126	0.00128146	kidney	TCGA-KIRP,TCGA-KIRC,TCGA-KICH	OSTM1
cg09404376	ENSG00000118515	body	-0.1	3.53E-05	kidney	TCGA-KIRP,TCGA-KIRC,TCGA-KICH	SGK1
cg00000321	ENSG00000104332	promoter	-0.1	0.00901566	kidney	TCGA-KIRP,TCGA-KIRC,TCGA-KICH	SFRP1
cg14244439	ENSG00000169071	body	-0.3	1.63E-15	kidney	TCGA-KIRP,TCGA-KIRC,TCGA-KICH	ROR2
cg04069951	ENSG00000238184	body	0.244	3.07E-10	kidney	TCGA-KIRP,TCGA-KIRC,TCGA-KICH	AC129929.5
cg23904595	ENSG00000129757	promoter	-0.2	2.71E-14	kidney	TCGA-KIRP,TCGA-KIRC,TCGA-KICH	CDKN1C
cg00953256	ENSG00000110092	body	-0.5	2.02E-60	kidney	TCGA-KIRP,TCGA-KIRC,TCGA-KICH	CCND1
cg27255672	ENSG00000149294	body	0.255	4.32E-11	kidney	TCGA-KIRP,TCGA-KIRC,TCGA-KICH	NCAM1
cg21490635	ENSG00000134531	promoter	-0.2	4.07E-07	kidney	TCGA-KIRP,TCGA-KIRC,TCGA-KICH	EMP1
cg02962597	ENSG00000089250	body	0.134	0.00059376	kidney	TCGA-KIRP,TCGA-KIRC,TCGA-KICH	NOS1
cg10434653	ENSG00000118900	body	-0.1	0.00520577	kidney	TCGA-KIRP,TCGA-KIRC,TCGA-KICH	UBN1
cg16645423	ENSG00000188322	body	0.126	0.00129945	kidney	TCGA-KIRP,TCGA-KIRC,TCGA-KICH	SBK1
cg09559780	ENSG00000167207	promoter	0.147	0.00016879	kidney	TCGA-KIRP,TCGA-KIRC,TCGA-KICH	NOD2
cg09568217	ENSG00000169992	body	0.201	2.41E-07	kidney	TCGA-KIRP,TCGA-KIRC,TCGA-KICH	NLGN2
cg07472953	ENSG00000108342	promoter	-0.1	3.09E-05	kidney	TCGA-KIRP,TCGA-KIRC,TCGA-KICH	CSF3
cg00179070	ENSG00000108821	promoter	0.129	0.00096496	kidney	TCGA-KIRP,TCGA-KIRC,TCGA-KICH	COL1A1
cg15659599	ENSG00000239998	body	0.232	2.13E-09	kidney	TCGA-KIRP,TCGA-KIRC,TCGA-KICH	LILRA2
cg09618904	ENSG00000125965	body	-0.1	0.00092865	kidney	TCGA-KIRP,TCGA-KIRC,TCGA-KICH	GDF5
cg23480341	ENSG00000124107	promoter	-0.3	5.44E-17	kidney	TCGA-KIRP,TCGA-KIRC,TCGA-KICH	SLPI
cg00138126	ENSG00000124225	promoter	-0.3	3.13E-25	kidney	TCGA-KIRP,TCGA-KIRC,TCGA-KICH	PMEPA1
cg13411522	ENSG00000092758	body	-0.1	0.00224363	kidney	TCGA-KIRP,TCGA-KIRC,TCGA-KICH	COL9A3
cg10324998	ENSG00000069702	body	-0.1	0.00045767	liver	TCGA-LIHC	TGFBR3
cg09473826	ENSG00000116147	body	0.302	8.51E-10	liver	TCGA-LIHC	TNR
cg11341981	ENSG00000163485	body	0.427	5.98E-19	liver	TCGA-LIHC	ADORA1
cg09699787	ENSG00000130508	body	0.305	6.37E-10	liver	TCGA-LIHC	PXDN
cg06968859	ENSG00000066032	body	0.143	0.00452589	liver	TCGA-LIHC	CTNNA2
cg14005211	ENSG00000128683	body	0.351	6.84E-13	liver	TCGA-LIHC	GAD1
cg18544564	ENSG00000168542	body	0.147	0.00347973	liver	TCGA-LIHC	COL3A1
cg09211763	ENSG00000204262	body	-0.2	9.36E-08	liver	TCGA-LIHC	COL5A2
cg02482042	ENSG00000157087	body	0.605	7.17E-41	liver	TCGA-LIHC	ATP2B2
cg22132982	ENSG00000154764	body	0.355	3.65E-13	liver	TCGA-LIHC	WNT7A
cg24899315	ENSG00000114646	body	0.184	0.00023736	liver	TCGA-LIHC	CSPG5
cg12325605	ENSG00000163947	body	0.416	5.69E-18	liver	TCGA-LIHC	ARHGEF3
cg24279243	ENSG00000109787	body	-0.1	0.00094749	liver	TCGA-LIHC	KLF3
cg12623364	ENSG00000138795	body	0.288	5.88E-09	liver	TCGA-LIHC	LEF1
cg05618426	ENSG00000151617	promoter	0.168	0.00080932	liver	TCGA-LIHC	EDNRA
cg25403205	ENSG00000204472	body	-0.1	0.00020484	liver	TCGA-LIHC	AIF1
cg25764570	ENSG00000204287	promoter	0.356	3.05E-13	liver	TCGA-LIHC	HLA-DRA
cg06204447	ENSG00000196126	body	0.363	9.04E-14	liver	TCGA-LIHC	HLA-DRB1
cg06499030	ENSG00000232629	promoter	0.383	2.82E-15	liver	TCGA-LIHC	HLA-DQB2
cg02196834	ENSG00000081087	body	-0.1	0.00400898	liver	TCGA-LIHC	OSTM1
cg14244439	ENSG00000169071	body	0.343	2.34E-12	liver	TCGA-LIHC	ROR2
cg04069951	ENSG00000110651	body	-0.3	8.91E-15	liver	TCGA-LIHC	CD81
cg23904595	ENSG00000129757	promoter	0.202	5.18E-05	liver	TCGA-LIHC	CDKN1C
cg27255672	ENSG00000149294	body	0.427	6.58E-19	liver	TCGA-LIHC	NCAM1
cg02962597	ENSG00000089250	body	0.169	0.00075112	liver	TCGA-LIHC	NOS1
cg15718287	ENSG00000133083	body	0.425	9.79E-19	liver	TCGA-LIHC	DCLK1
cg25759064	ENSG00000102606	body	-0.2	1.82E-08	liver	TCGA-LIHC	ARHGEF7
cg09559780	ENSG00000167207	promoter	-0.2	3.10E-07	liver	TCGA-LIHC	NOD2
cg06545761	ENSG00000140948	body	0.152	0.00253009	liver	TCGA-LIHC	ZCCHC14
cg09568217	ENSG00000169992	body	0.182	0.00028373	liver	TCGA-LIHC	NLGN2
cg00179070	ENSG00000108821	promoter	0.185	0.00021581	liver	TCGA-LIHC	COL1A1
cg08823411	ENSG00000171246	body	0.264	1.03E-07	liver	TCGA-LIHC	NPTX1
cg15659599	ENSG00000239998	body	0.564	1.32E-34	liver	TCGA-LIHC	LILRA2
cg23480341	ENSG00000124107	promoter	0.171	0.00063028	liver	TCGA-LIHC	SLPI
cg00138126	ENSG00000124225	promoter	0.614	3.13E-42	liver	TCGA-LIHC	PMEPA1
cg00138126	ENSG00000278709	body	0.456	1.21E-21	liver	TCGA-LIHC	RP5-1059L7.1
cg10324998	ENSG00000069702	body	0.256	2.69E-06	stomach	TCGA-STAD	TGFBR3
cg09473826	ENSG00000116147	body	0.146	0.00832183	stomach	TCGA-STAD	TNR
cg05262549	ENSG00000157064	body	0.173	0.00173055	stomach	TCGA-STAD	NMNAT2
cg11341981	ENSG00000163485	body	0.376	1.90E-12	stomach	TCGA-STAD	ADORA1
cg06968859	ENSG00000066032	body	0.252	3.88E-06	stomach	TCGA-STAD	CTNNA2
cg14005211	ENSG00000128683	body	0.586	1.66E-31	stomach	TCGA-STAD	GAD1
cg02482042	ENSG00000157087	body	0.152	0.00602944	stomach	TCGA-STAD	ATP2B2
cg22132982	ENSG00000154764	body	0.19	0.00054573	stomach	TCGA-STAD	WNT7A
cg24899315	ENSG00000114646	body	-0.2	0.00016877	stomach	TCGA-STAD	CSPG5
cg21567371	ENSG00000114270	body	0.179	0.00117557	stomach	TCGA-STAD	COL7A1
cg04376312	ENSG00000065534	body	-0.1	0.00125505	stomach	TCGA-STAD	MYLK
cg24279243	ENSG00000109787	body	-0.3	4.71E-09	stomach	TCGA-STAD	KLF3
cg12623364	ENSG00000138795	body	0.232	2.23E-05	stomach	TCGA-STAD	LEF1
cg05618426	ENSG00000151617	promoter	-0.4	1.02E-13	stomach	TCGA-STAD	EDNRA
cg03176423	ENSG00000214922	body	0.173	0.00165304	stomach	TCGA-STAD	XXbac-BPG170G13.33
cg25403205	ENSG00000204472	body	-0.2	9.45E-06	stomach	TCGA-STAD	AIF1
cg25764570	ENSG00000204287	promoter	0.285	1.53E-07	stomach	TCGA-STAD	HLA-DRA
cg06204447	ENSG00000196126	body	0.37	4.67E-12	stomach	TCGA-STAD	HLA-DRB1
cg06499030	ENSG00000232629	promoter	0.218	7.14E-05	stomach	TCGA-STAD	HLA-DQB2
cg09404376	ENSG00000118515	body	0.272	5.74E-07	stomach	TCGA-STAD	SGK1
cg14244439	ENSG00000169071	body	0.466	5.19E-19	stomach	TCGA-STAD	ROR2
cg00953256	ENSG00000110092	body	-0.2	8.41E-06	stomach	TCGA-STAD	CCND1
cg00953256	ENSG00000149716	body	-0.1	0.00041215	stomach	TCGA-STAD	ORAOV1
cg27255672	ENSG00000149294	body	0.467	4.33E-19	stomach	TCGA-STAD	NCAM1
cg02962597	ENSG00000089250	body	0.161	0.00349891	stomach	TCGA-STAD	NOS1
cg15718287	ENSG00000133083	body	0.499	5.34E-22	stomach	TCGA-STAD	DCLK1
cg15324756	ENSG00000134871	body	-0.1	0.00125087	stomach	TCGA-STAD	COL4A2
cg25759064	ENSG00000102606	body	-0.2	0.00013847	stomach	TCGA-STAD	ARHGEF7
cg10434653	ENSG00000140632	promoter	-0.1	0.00836826	stomach	TCGA-STAD	GLYR1
cg10434653	ENSG00000118900	body	-0.1	0.00874039	stomach	TCGA-STAD	UBN1
cg16645423	ENSG00000188322	body	0.287	1.26E-07	stomach	TCGA-STAD	SBK1
cg09568217	ENSG00000169992	body	0.143	0.00945085	stomach	TCGA-STAD	NLGN2
cg11321181	ENSG00000130477	body	0.241	1.03E-05	stomach	TCGA-STAD	UNC13A
cg02149446	ENSG00000197405	body	0.356	3.12E-11	stomach	TCGA-STAD	C5AR1
cg15659599	ENSG00000239998	body	0.494	1.58E-21	stomach	TCGA-STAD	LILRA2
cg23480341	ENSG00000124107	promoter	-0.4	3.10E-14	stomach	TCGA-STAD	SLPI
cg13411522	ENSG00000092758	body	-0.4	2.03E-20	stomach	TCGA-STAD	COL9A3
cg09473826	ENSG00000116147	body	0.385	3.93E-06	testis	TCGA-TGCT	TNR
cg05262549	ENSG00000157064	body	0.552	3.97E-12	testis	TCGA-TGCT	NMNAT2
cg11341981	ENSG00000163485	body	0.651	1.31E-17	testis	TCGA-TGCT	ADORA1
cg14005211	ENSG00000128683	body	0.553	3.60E-12	testis	TCGA-TGCT	GAD1
cg22132982	ENSG00000154764	body	0.271	0.00149285	testis	TCGA-TGCT	WNT7A
cg21567371	ENSG00000114270	body	0.587	7.41E-14	testis	TCGA-TGCT	COL7A1
cg21213853	ENSG00000164082	body	0.245	0.00422413	testis	TCGA-TGCT	GRM2
cg04376312	ENSG00000065534	body	0.398	1.78E-06	testis	TCGA-TGCT	MYLK
cg05618426	ENSG00000151617	promoter	0.433	1.55E-07	testis	TCGA-TGCT	EDNRA
cg25764570	ENSG00000204287	promoter	-0.2	0.00566348	testis	TCGA-TGCT	HLA-DRA
cg10591607	ENSG00000164418	body	0.292	0.00060075	testis	TCGA-TGCT	GRIK2
cg00000321	ENSG00000104332	promoter	0.482	3.32E-09	testis	TCGA-TGCT	SFRP1
cg14244439	ENSG00000169071	body	0.295	0.00050411	testis	TCGA-TGCT	ROR2
cg04069951	ENSG00000238184	body	0.318	0.00017029	testis	TCGA-TGCT	AC129929.5
cg23904595	ENSG00000129757	promoter	0.568	6.71E-13	testis	TCGA-TGCT	CDKN1C
cg00953256	ENSG00000110092	body	0.563	1.18E-12	testis	TCGA-TGCT	CCND1
cg00953256	ENSG00000149716	body	-0.3	3.77E-06	testis	TCGA-TGCT	ORAOV1
cg27255672	ENSG00000149294	body	0.477	4.75E-09	testis	TCGA-TGCT	NCAM1
cg21490635	ENSG00000134531	promoter	0.385	4.08E-06	testis	TCGA-TGCT	EMP1
cg02962597	ENSG00000089250	body	0.493	1.20E-09	testis	TCGA-TGCT	NOS1
cg15718287	ENSG00000133083	body	0.572	4.19E-13	testis	TCGA-TGCT	DCLK1
cg10434653	ENSG00000140632	promoter	-0.2	0.0027515	testis	TCGA-TGCT	GLYR1
cg09568217	ENSG00000169992	body	0.387	3.44E-06	testis	TCGA-TGCT	NLGN2
cg00179070	ENSG00000108821	promoter	0.319	0.00016598	testis	TCGA-TGCT	COL1A1
cg08823411	ENSG00000171246	body	0.495	1.05E-09	testis	TCGA-TGCT	NPTX1
cg02149446	ENSG00000197405	body	0.408	9.08E-07	testis	TCGA-TGCT	C5AR1
cg15659599	ENSG00000239998	body	0.316	0.00018767	testis	TCGA-TGCT	LILRA2
cg23480341	ENSG00000124107	promoter	0.394	2.29E-06	testis	TCGA-TGCT	SLPI
cg07612655	ENSG00000124212	promoter	0.551	4.37E-12	testis	TCGA-TGCT	PTGIS
cg00138126	ENSG00000124225	promoter	0.387	3.45E-06	testis	TCGA-TGCT	PMEPA1
cg00138126	ENSG00000278709	body	0.388	3.33E-06	testis	TCGA-TGCT	RP5-1059L7.1
cg16402547	ENSG00000075043	promoter	0.449	4.79E-08	testis	TCGA-TGCT	KCNQ2

ROSMAP

probe	gene	location	cor	p	tissue	source	gene_symbol
cg06285439	ENSG00000118257	body	-0.24	6.34E-09	brain	TCGA-GBM,TCGA-LGG	NRP2
cg17913386	ENSG00000114270	body	0.268	7.75E-11	brain	TCGA-GBM,TCGA-LGG	COL7A1
cg12325605	ENSG00000163947	body	-0.3	5.93E-13	brain	TCGA-GBM,TCGA-LGG	ARHGEF3
cg10426370	ENSG00000164283	body	-0.23	4.24E-08	brain	TCGA-GBM,TCGA-LGG	ESM1
cg09079275	ENSG00000135074	promoter	-0.24	6.30E-09	brain	TCGA-GBM,TCGA-LGG	ADAM19
cg00094412	ENSG00000204681	body	-0.19	5.39E-06	brain	TCGA-GBM,TCGA-LGG	GABBR1
cg15343510	ENSG00000204472	promoter	-0.43	1.42E-27	brain	TCGA-GBM,TCGA-LGG	AIF1
cg17606183	ENSG00000204287	body	-0.31	3.49E-14	brain	TCGA-GBM,TCGA-LGG	HLA-DRA
cg09310966	ENSG00000124731	promoter	-0.43	1.91E-27	brain	TCGA-GBM,TCGA-LGG	TREM1
cg17693222	ENSG00000104368	body	-0.24	8.86E-09	brain	TCGA-GBM,TCGA-LGG	PLAT
cg13491071	ENSG00000169071	body	-0.49	4.29E-35	brain	TCGA-GBM,TCGA-LGG	ROR2
cg13734061	ENSG00000066468	body	0.112	0.00763801	brain	TCGA-GBM,TCGA-LGG	FGFR2
cg15974867	ENSG00000110092	body	-0.28	5.27E-12	brain	TCGA-GBM,TCGA-LGG	CCND1
cg15418499	ENSG00000150782	body	-0.48	2.58E-34	brain	TCGA-GBM,TCGA-LGG	IL18
cg08300930	ENSG00000175899	body	-0.23	2.96E-08	brain	TCGA-GBM,TCGA-LGG	A2M
cg14622549	ENSG00000183495	body	0.131	0.00178359	brain	TCGA-GBM,TCGA-LGG	EP400
cg15901783	ENSG00000178695	promoter	-0.21	3.07E-07	brain	TCGA-GBM,TCGA-LGG	KCTD12
cg15901783	ENSG00000264908	promoter	-0.14	0.00129533	brain	TCGA-GBM,TCGA-LGG	AC000403.1
cg15901783	ENSG00000278727	promoter	-0.21	5.33E-07	brain	TCGA-GBM,TCGA-LGG	AC000403.4
cg08959059	ENSG00000134871	body	-0.51	1.25E-39	brain	TCGA-GBM,TCGA-LGG	COL4A2
cg01802545	ENSG00000100600	body	-0.16	8.80E-05	brain	TCGA-GBM,TCGA-LGG	LGMN
cg04193923	ENSG00000108342	promoter	-0.17	7.82E-05	brain	TCGA-GBM,TCGA-LGG	CSF3
cg09764761	ENSG00000186868	body	0.153	0.00025727	brain	TCGA-GBM,TCGA-LGG	MAPT
cg25384595	ENSG00000104974	body	-0.4	8.75E-23	brain	TCGA-GBM,TCGA-LGG	LILRA1
cg15505615	ENSG00000092758	promoter	-0.16	0.00014238	brain	TCGA-GBM,TCGA-LGG	COL9A3
cg01336162	ENSG00000069702	body	-0.2	0.00028717	colon	TCGA-COAD	TGFBR3
cg07781364	ENSG00000179915	body	0.312	1.41E-06	colon	TCGA-COAD	NRXN1
cg06285439	ENSG00000118257	body	0.366	1.18E-08	colon	TCGA-COAD	NRP2
cg12325605	ENSG00000163947	body	-0.1	0.00339574	colon	TCGA-COAD	ARHGEF3
cg09520393	ENSG00000109667	body	-0.1	0.00378366	colon	TCGA-COAD	SLC2A9
cg10426370	ENSG00000164283	body	-0.2	0.0023334	colon	TCGA-COAD	ESM1
cg09079275	ENSG00000135074	promoter	-0.2	4.54E-05	colon	TCGA-COAD	ADAM19
cg00094412	ENSG00000204681	body	0.5	6.70E-16	colon	TCGA-COAD	GABBR1
cg24351901	ENSG00000204642	body	0.4	6.16E-14	colon	TCGA-COAD	HLA-F
cg24351901	ENSG00000225864	body	-0.2	0.00023188	colon	TCGA-COAD	HCG4P11
cg13734061	ENSG00000066468	body	0.193	0.00334494	colon	TCGA-COAD	FGFR2
cg15974867	ENSG00000110092	body	0.181	0.00588837	colon	TCGA-COAD	CCND1
cg11435826	ENSG00000151067	body	0.397	4.66E-10	colon	TCGA-COAD	CACNA1C
cg15901783	ENSG00000178695	promoter	0.181	0.00589976	colon	TCGA-COAD	KCTD12
cg22904711	ENSG00000104783	body	-0.1	0.00597964	colon	TCGA-COAD	KCNN4
cg15505615	ENSG00000092758	promoter	-0.1	0.00654119	colon	TCGA-COAD	COL9A3
cg01336162	ENSG00000069702	body	-0.49	4.90E-40	kidney	TCGA-KIRP,TCGA-KIRC,TCGA-KICH	TGFBR3
cg06285439	ENSG00000118257	body	0.298	8.47E-15	kidney	TCGA-KIRP,TCGA-KIRC,TCGA-KICH	NRP2
cg09595163	ENSG00000114251	body	0.322	3.58E-17	kidney	TCGA-KIRP,TCGA-KIRC,TCGA-KICH	WNT5A
cg12325605	ENSG00000163947	body	-0.12	0.00242212	kidney	TCGA-KIRP,TCGA-KIRC,TCGA-KICH	ARHGEF3
cg09520393	ENSG00000109667	body	0.119	0.00239435	kidney	TCGA-KIRP,TCGA-KIRC,TCGA-KICH	SLC2A9
cg10426370	ENSG00000164283	body	-0.37	7.80E-23	kidney	TCGA-KIRP,TCGA-KIRC,TCGA-KICH	ESM1
cg00094412	ENSG00000204681	body	0.31	5.75E-16	kidney	TCGA-KIRP,TCGA-KIRC,TCGA-KICH	GABBR1
cg24351901	ENSG00000204642	body	-0.38	5.85E-24	kidney	TCGA-KIRP,TCGA-KIRC,TCGA-KICH	HLA-F
cg15343510	ENSG00000204472	promoter	-0.51	1.22E-44	kidney	TCGA-KIRP,TCGA-KIRC,TCGA-KICH	AIF1
cg17606183	ENSG00000204287	body	-0.22	1.56E-08	kidney	TCGA-KIRP,TCGA-KIRC,TCGA-KICH	HLA-DRA
cg09310966	ENSG00000124731	promoter	-0.39	5.77E-25	kidney	TCGA-KIRP,TCGA-KIRC,TCGA-KICH	TREM1
cg17693222	ENSG00000104368	body	-0.23	1.54E-09	kidney	TCGA-KIRP,TCGA-KIRC,TCGA-KICH	PLAT
cg13491071	ENSG00000169071	body	0.13	0.00089742	kidney	TCGA-KIRP,TCGA-KIRC,TCGA-KICH	ROR2
cg15974867	ENSG00000110092	body	-0.37	4.95E-22	kidney	TCGA-KIRP,TCGA-KIRC,TCGA-KICH	CCND1
cg15418499	ENSG00000150782	body	-0.33	1.47E-17	kidney	TCGA-KIRP,TCGA-KIRC,TCGA-KICH	IL18
cg08300930	ENSG00000175899	body	-0.59	2.97E-62	kidney	TCGA-KIRP,TCGA-KIRC,TCGA-KICH	A2M
cg15901783	ENSG00000178695	promoter	-0.28	1.36E-13	kidney	TCGA-KIRP,TCGA-KIRC,TCGA-KICH	KCTD12
cg15901783	ENSG00000278727	promoter	-0.13	0.00103998	kidney	TCGA-KIRP,TCGA-KIRC,TCGA-KICH	AC000403.4
cg08959059	ENSG00000134871	body	0.47	2.54E-36	kidney	TCGA-KIRP,TCGA-KIRC,TCGA-KICH	COL4A2
cg01802545	ENSG00000100600	body	0.24	3.25E-10	kidney	TCGA-KIRP,TCGA-KIRC,TCGA-KICH	LGMN
cg04193923	ENSG00000265799	body	0.11	0.00642363	kidney	TCGA-KIRP,TCGA-KIRC,TCGA-KICH	RP11-387H17.6
cg04193923	ENSG00000108342	promoter	-0.16	3.55E-05	kidney	TCGA-KIRP,TCGA-KIRC,TCGA-KICH	CSF3
cg09764761	ENSG00000186868	body	-0.32	6.02E-17	kidney	TCGA-KIRP,TCGA-KIRC,TCGA-KICH	MAPT
cg22904711	ENSG00000104783	body	0.664	7.39E-84	kidney	TCGA-KIRP,TCGA-KIRC,TCGA-KICH	KCNN4
cg25384595	ENSG00000104974	body	-0.37	4.54E-23	kidney	TCGA-KIRP,TCGA-KIRC,TCGA-KICH	LILRA1
cg01336162	ENSG00000069702	body	-0.2	3.24E-05	liver	TCGA-LIHC	TGFBR3
cg06285439	ENSG00000118257	body	0.245	8.02E-07	liver	TCGA-LIHC	NRP2
cg17913386	ENSG00000114270	body	0.169	0.00074666	liver	TCGA-LIHC	COL7A1
cg09595163	ENSG00000114251	body	0.28	1.42E-08	liver	TCGA-LIHC	WNT5A
cg12325605	ENSG00000163947	body	0.416	5.69E-18	liver	TCGA-LIHC	ARHGEF3
cg10426370	ENSG00000164283	body	0.3	9.40E-08	liver	TCGA-LIHC	ESM1
cg09079275	ENSG00000135074	promoter	-0.2	2.71E-06	liver	TCGA-LIHC	ADAM19
cg00094412	ENSG00000204681	body	0.645	9.30E-48	liver	TCGA-LIHC	GABBR1
cg24351901	ENSG00000204642	body	0.2	8.96E-09	liver	TCGA-LIHC	HLA-F
cg24351901	ENSG00000225864	promoter	-0.2	5.21E-05	liver	TCGA-LIHC	HCG4P11
cg17606183	ENSG00000204287	body	0.354	4.00E-13	liver	TCGA-LIHC	HLA-DRA
cg09310966	ENSG00000124731	promoter	0.158	0.00163277	liver	TCGA-LIHC	TREM1
cg17693222	ENSG00000104368	body	0.189	0.00015637	liver	TCGA-LIHC	PLAT
cg13491071	ENSG00000169071	body	0.187	0.00018702	liver	TCGA-LIHC	ROR2
cg13734061	ENSG00000066468	body	0.592	1.05E-38	liver	TCGA-LIHC	FGFR2
cg15974867	ENSG00000110092	body	0.3	5.07E-10	liver	TCGA-LIHC	CCND1
cg15418499	ENSG00000150782	body	0.425	8.68E-19	liver	TCGA-LIHC	IL18
cg11435826	ENSG00000151067	body	0.211	2.37E-05	liver	TCGA-LIHC	CACNA1C
cg08300930	ENSG00000175899	body	0.6	1.17E-42	liver	TCGA-LIHC	A2M
cg15901783	ENSG00000178695	promoter	0.363	9.59E-14	liver	TCGA-LIHC	KCTD12
cg15901783	ENSG00000278727	promoter	0.259	1.69E-07	liver	TCGA-LIHC	AC000403.4
cg08959059	ENSG00000134871	body	0.157	0.00180657	liver	TCGA-LIHC	COL4A2
cg22904711	ENSG00000104783	body	0.591	1.27E-38	liver	TCGA-LIHC	KCNN4
cg25384595	ENSG00000104974	body	0.284	9.59E-09	liver	TCGA-LIHC	LILRA1
cg15505615	ENSG00000092758	promoter	-0.1	0.0003804	liver	TCGA-LIHC	COL9A3
cg03008387	ENSG00000075043	body	0.138	0.00616505	liver	TCGA-LIHC	KCNQ2
cg01336162	ENSG00000069702	body	0.2	2.43E-05	stomach	TCGA-STAD	TGFBR3
cg06285439	ENSG00000118257	body	0.474	1.06E-19	stomach	TCGA-STAD	NRP2
cg09520393	ENSG00000109667	body	0.1	0.0057324	stomach	TCGA-STAD	SLC2A9
cg09079275	ENSG00000135074	promoter	0.2	1.64E-05	stomach	TCGA-STAD	ADAM19
cg00094412	ENSG00000204681	body	0.468	3.07E-19	stomach	TCGA-STAD	GABBR1
cg24351901	ENSG00000204642	body	0.3	9.58E-11	stomach	TCGA-STAD	HLA-F
cg24351901	ENSG00000225864	promoter	-0.2	7.72E-08	stomach	TCGA-STAD	HCG4P11
cg15418499	ENSG00000150782	body	0.184	0.00060684	stomach	TCGA-STAD	IL18
cg11435826	ENSG00000151067	body	0.286	1.36E-07	stomach	TCGA-STAD	CACNA1C
cg01802545	ENSG00000100600	body	0.152	0.00447752	stomach	TCGA-STAD	LGMN
cg09764761	ENSG00000186868	body	0.1	0.00474262	stomach	TCGA-STAD	MAPT
cg22904711	ENSG00000104783	body	-0.2	1.74E-06	stomach	TCGA-STAD	KCNN4
cg15505615	ENSG00000092758	promoter	-0.2	3.68E-17	stomach	TCGA-STAD	COL9A3
cg03008387	ENSG00000075043	body	0.278	3.16E-07	stomach	TCGA-STAD	KCNQ2
cg06285439	ENSG00000118257	body	0.79	5.14E-30	testis	TCGA-TGCT	NRP2
cg17913386	ENSG00000114270	body	0.239	0.00534143	testis	TCGA-TGCT	COL7A1
cg09595163	ENSG00000114251	body	0.473	6.99E-09	testis	TCGA-TGCT	WNT5A
cg09520393	ENSG00000109667	body	0.274	0.00530974	testis	TCGA-TGCT	SLC2A9
cg09520393	ENSG00000169676	promoter	0.369	1.04E-05	testis	TCGA-TGCT	DRD5
cg10426370	ENSG00000164283	body	0.396	2.01E-06	testis	TCGA-TGCT	ESM1
cg00094412	ENSG00000204681	body	0.208	0.00173908	testis	TCGA-TGCT	GABBR1
cg09310966	ENSG00000124731	promoter	0.335	7.23E-05	testis	TCGA-TGCT	TREM1
cg17693222	ENSG00000104368	body	0.764	4.46E-27	testis	TCGA-TGCT	PLAT
cg13491071	ENSG00000169071	body	-0.3	3.40E-06	testis	TCGA-TGCT	ROR2
cg15974867	ENSG00000110092	body	0.812	6.92E-33	testis	TCGA-TGCT	CCND1
cg11435826	ENSG00000151067	body	0.443	7.66E-08	testis	TCGA-TGCT	CACNA1C
cg11435826	ENSG00000246627	body	0.6	6.78E-20	testis	TCGA-TGCT	RP11-885B4.3
cg14622549	ENSG00000183495	body	-0.4	1.09E-08	testis	TCGA-TGCT	EP400
cg08959059	ENSG00000134871	body	0.459	2.09E-08	testis	TCGA-TGCT	COL4A2
cg22904711	ENSG00000104783	body	0.585	8.79E-14	testis	TCGA-TGCT	KCNN4
cg25384595	ENSG00000104974	body	-0.2	0.00060783	testis	TCGA-TGCT	LILRA1
cg03008387	ENSG00000075043	body	0.493	1.21E-09	testis	TCGA-TGCT	KCNQ2

[illegible]
