# Supplementary material for: Genetic and pathway complexity in Alzheimer’s disease: Insights from multi-omic data about the immune response and mitochondrial function
Source: Neural Regen Res. 2025 Sep 29;21(9):4406–23. doi: 10.4103/NRR.NRR-D-25-00184 (PMC13557549; doi:10.4103/NRR.NRR-D-25-00184)
Supplement: Supplementary file 8 [file NRR-21-4406_Suppl16.pdf]

Additional Table [illegible]: Enrichment results of important CpGs [illegible] for traits and biological functions

[illegible]

| trait | p | [illegible] | [illegible] | [illegible] | [illegible] | [illegible] |
| --- | --- | --- | --- | --- | --- | --- |
| primary Sjögren's Syndrome (pSS) | [illegible] | [illegible] | [illegible] | [illegible] | [illegible] | [illegible] |
| derivatives of reactive oxygen metabolites (D-ROM) in plasma | [illegible] | [illegible] | [illegible] | [illegible] | [illegible] | [illegible] |
| weight loss | [illegible] | [illegible] | [illegible] | [illegible] | [illegible] | [illegible] |
| leukoaraiosis (LA) | [illegible] | [illegible] | [illegible] | [illegible] | [illegible] | [illegible] |
| down syndrome | [illegible] | [illegible] | [illegible] | [illegible] | [illegible] | [illegible] |
| aging | [illegible] | [illegible] | [illegible] | [illegible] | [illegible] | [illegible] |
| depressive disorder | [illegible] | [illegible] | [illegible] | [illegible] | [illegible] | [illegible] |
| lifetime estrogen exposure | [illegible] | [illegible] | [illegible] | [illegible] | [illegible] | [illegible] |
| Hyperdiploid B-Acute Lymphoblastic Leukemia | [illegible] | [illegible] | [illegible] | [illegible] | [illegible] | [illegible] |
| asthma | [illegible] | [illegible] | [illegible] | [illegible] | [illegible] | [illegible] |
| short-term diesel exhaust inhalation | [illegible] | [illegible] | [illegible] | [illegible] | [illegible] | [illegible] |
| soluble tumor necrosis factor receptor 2 (sTNFR2) levels in plasma | [illegible] | [illegible] | [illegible] | [illegible] | [illegible] | [illegible] |
| smoking | [illegible] | [illegible] | [illegible] | [illegible] | [illegible] | [illegible] |
| colorectal laterally spreading tumor | [illegible] | [illegible] | [illegible] | [illegible] | [illegible] | [illegible] |
| hepatocellular carcinoma (HCC) | [illegible] | [illegible] | [illegible] | [illegible] | [illegible] | [illegible] |
| air pollution (NO2) | [illegible] | [illegible] | [illegible] | [illegible] | [illegible] | [illegible] |
| infertility | [illegible] | [illegible] | [illegible] | [illegible] | [illegible] | [illegible] |
| atherosclerosis | [illegible] | [illegible] | [illegible] | [illegible] | [illegible] | [illegible] |
| cognitive function | [illegible] | [illegible] | [illegible] | [illegible] | [illegible] | [illegible] |
| prostate cancer | [illegible] | [illegible] | [illegible] | [illegible] | [illegible] | [illegible] |

| KEGG pathway | term | [illegible] | [illegible] | p | [illegible] |
| --- | --- | --- | --- | --- | --- |
| [illegible] | Viral myocarditis | [illegible] | [illegible] | [illegible] | [illegible] |
| [illegible] | PI3K-Akt signaling pathway | [illegible] | [illegible] | [illegible] | [illegible] |
| [illegible] | Hematopoietic cell lineage | [illegible] | [illegible] | [illegible] | [illegible] |
| [illegible] | Hippo signaling pathway | [illegible] | [illegible] | [illegible] | [illegible] |
| [illegible] | Type I diabetes mellitus | [illegible] | [illegible] | [illegible] | [illegible] |
| [illegible] | Protein digestion and absorption | [illegible] | [illegible] | [illegible] | [illegible] |
| [illegible] | Staphylococcus aureus infection | [illegible] | [illegible] | [illegible] | [illegible] |
| [illegible] | Cell adhesion molecules | [illegible] | [illegible] | [illegible] | [illegible] |
| [illegible] | Neuroactive ligand-receptor interaction | [illegible] | [illegible] | [illegible] | [illegible] |
| [illegible] | Allograft rejection | [illegible] | [illegible] | [illegible] | [illegible] |
| [illegible] | Intestinal immune network for IgA production | [illegible] | [illegible] | [illegible] | [illegible] |
| [illegible] | Graft-versus-host disease | [illegible] | [illegible] | [illegible] | [illegible] |
| [illegible] | Rheumatoid arthritis | [illegible] | [illegible] | [illegible] | [illegible] |
| [illegible] | Focal adhesion | [illegible] | [illegible] | [illegible] | [illegible] |
| [illegible] | Autoimmune thyroid disease | [illegible] | [illegible] | [illegible] | [illegible] |
| [illegible] | Cytoskeleton in muscle cells | [illegible] | [illegible] | [illegible] | [illegible] |
| [illegible] | ECM-receptor interaction | [illegible] | [illegible] | [illegible] | [illegible] |
| [illegible] | Pathways in cancer | [illegible] | [illegible] | [illegible] | [illegible] |
| [illegible] | Gastric cancer | [illegible] | [illegible] | [illegible] | [illegible] |
| [illegible] | Asthma | [illegible] | [illegible] | [illegible] | [illegible] |
| [illegible] | Inflammatory bowel disease | [illegible] | [illegible] | [illegible] | [illegible] |
| [illegible] | Relaxin signaling pathway | [illegible] | [illegible] | [illegible] | [illegible] |
| [illegible] | Leishmaniasis | [illegible] | [illegible] | [illegible] | [illegible] |
| [illegible] | Wnt signaling pathway | [illegible] | [illegible] | [illegible] | [illegible] |
| [illegible] | Nicotinate and nicotinamide metabolism | [illegible] | [illegible] | [illegible] | [illegible] |
| [illegible] | Systemic lupus erythematosus | [illegible] | [illegible] | [illegible] | [illegible] |
| [illegible] | Apelin signaling pathway | [illegible] | [illegible] | [illegible] | [illegible] |
| [illegible] | AGE-RAGE signaling pathway in diabetic complications | [illegible] | [illegible] | [illegible] | [illegible] |
| [illegible] | Glutamatergic synapse | [illegible] | [illegible] | [illegible] | [illegible] |
| [illegible] | Calcium signaling pathway | [illegible] | [illegible] | [illegible] | [illegible] |
| [illegible] | GABAergic synapse | [illegible] | [illegible] | [illegible] | [illegible] |
| [illegible] | Phagosome | [illegible] | [illegible] | [illegible] | [illegible] |
| [illegible] | cAMP signaling pathway | [illegible] | [illegible] | [illegible] | [illegible] |
| [illegible] | Malaria | [illegible] | [illegible] | [illegible] | [illegible] |
| [illegible] | Hepatocellular carcinoma | [illegible] | [illegible] | [illegible] | [illegible] |
| [illegible] | cGMP-PKG signaling pathway | [illegible] | [illegible] | [illegible] | [illegible] |
| [illegible] | Human papillomavirus infection | [illegible] | [illegible] | [illegible] | [illegible] |
| [illegible] | Human T-cell leukemia virus 1 infection | [illegible] | [illegible] | [illegible] | [illegible] |
| [illegible] | Dopaminergic synapse | [illegible] | [illegible] | [illegible] | [illegible] |
| [illegible] | Amoebiasis | [illegible] | [illegible] | [illegible] | [illegible] |
| [illegible] | Arginine biosynthesis | [illegible] | [illegible] | [illegible] | [illegible] |
| [illegible] | Th17 cell differentiation | [illegible] | [illegible] | [illegible] | [illegible] |
| [illegible] | Antigen processing and presentation | [illegible] | [illegible] | [illegible] | [illegible] |
| [illegible] | Toxoplasmosis | [illegible] | [illegible] | [illegible] | [illegible] |
| [illegible] | Hypertrophic cardiomyopathy | [illegible] | [illegible] | [illegible] | [illegible] |
| [illegible] | Melanogenesis | [illegible] | [illegible] | [illegible] | [illegible] |

| GO | term | [illegible] | [illegible] | [illegible] | p | [illegible] |
| --- | --- | --- | --- | --- | --- | --- |
| [illegible] | signaling | [illegible] | [illegible] | [illegible] | [illegible] | [illegible] |
| [illegible] | cellular response to stimulus | [illegible] | [illegible] | [illegible] | [illegible] | [illegible] |
| [illegible] | cell communication | [illegible] | [illegible] | [illegible] | [illegible] | [illegible] |
| [illegible] | signal transduction | [illegible] | [illegible] | [illegible] | [illegible] | [illegible] |
| [illegible] | cell surface receptor signaling pathway | [illegible] | [illegible] | [illegible] | [illegible] | [illegible] |
| [illegible] | response to stimulus | [illegible] | [illegible] | [illegible] | [illegible] | [illegible] |
| [illegible] | response to organic substance | [illegible] | [illegible] | [illegible] | [illegible] | [illegible] |
| [illegible] | regulation of response to stimulus | [illegible] | [illegible] | [illegible] | [illegible] | [illegible] |
| [illegible] | multicellular organismal process | [illegible] | [illegible] | [illegible] | [illegible] | [illegible] |
| [illegible] | system development | [illegible] | [illegible] | [illegible] | [illegible] | [illegible] |
| [illegible] | response to chemical | [illegible] | [illegible] | [illegible] | [illegible] | [illegible] |
| [illegible] | response to endogenous stimulus | [illegible] | [illegible] | [illegible] | [illegible] | [illegible] |
| [illegible] | regulation of multicellular organismal process | [illegible] | [illegible] | [illegible] | [illegible] | [illegible] |
| [illegible] | [illegible] | [illegible] | [illegible] | [illegible] | [illegible] | [illegible] |

[illegible]

| trait | p | [illegible] | [illegible] | [illegible] | [illegible] | [illegible] |
| --- | --- | --- | --- | --- | --- | --- |
| Alzheimer's disease (AD) | [illegible] | [illegible] | [illegible] | [illegible] | [illegible] | [illegible] |
| preterm birth | [illegible] | [illegible] | [illegible] | [illegible] | [illegible] | [illegible] |
| smoking | [illegible] | [illegible] | [illegible] | [illegible] | [illegible] | [illegible] |
| psoriasis | [illegible] | [illegible] | [illegible] | [illegible] | [illegible] | [illegible] |
| depressive disorder | [illegible] | [illegible] | [illegible] | [illegible] | [illegible] | [illegible] |
| oral squamous cell carcinoma (OSCC) | [illegible] | [illegible] | [illegible] | [illegible] | [illegible] | [illegible] |
| body mass index (BMI) | [illegible] | [illegible] | [illegible] | [illegible] | [illegible] | [illegible] |
| epithelial ovarian cancer risk | [illegible] | [illegible] | [illegible] | [illegible] | [illegible] | [illegible] |
| multiple sclerosis | [illegible] | [illegible] | [illegible] | [illegible] | [illegible] | [illegible] |
| aging | [illegible] | [illegible] | [illegible] | [illegible] | [illegible] | [illegible] |
| ancestry | [illegible] | [illegible] | [illegible] | [illegible] | [illegible] | [illegible] |
| cognitive function | [illegible] | [illegible] | [illegible] | [illegible] | [illegible] | [illegible] |
| folic acid supplement during pregnancy | [illegible] | [illegible] | [illegible] | [illegible] | [illegible] | [illegible] |
| air pollution (NO2) | [illegible] | [illegible] | [illegible] | [illegible] | [illegible] | [illegible] |
| childhood stress | [illegible] | [illegible] | [illegible] | [illegible] | [illegible] | [illegible] |
| down syndrome | [illegible] | [illegible] | [illegible] | [illegible] | [illegible] | [illegible] |
| household socioeconomic status in childhood | [illegible] | [illegible] | [illegible] | [illegible] | [illegible] | [illegible] |
| Kashin-Beck disease (KBD) | [illegible] | [illegible] | [illegible] | [illegible] | [illegible] | [illegible] |
| papillary thyroid carcinoma | [illegible] | [illegible] | [illegible] | [illegible] | [illegible] | [illegible] |
| [illegible] | [illegible] | [illegible] | [illegible] | [illegible] | [illegible] | [illegible] |
| maternal hypertensive disorders in pregnancy | [illegible] | [illegible] | [illegible] | [illegible] | [illegible] | [illegible] |
| smoking cessation | [illegible] | [illegible] | [illegible] | [illegible] | [illegible] | [illegible] |
| systemic lupus erythematosus (SLE) | [illegible] | [illegible] | [illegible] | [illegible] | [illegible] | [illegible] |
| Werner syndrome | [illegible] | [illegible] | [illegible] | [illegible] | [illegible] | [illegible] |
| allergic asthma | [illegible] | [illegible] | [illegible] | [illegible] | [illegible] | [illegible] |
| alcohol consumption | [illegible] | [illegible] | [illegible] | [illegible] | [illegible] | [illegible] |

| KEGG pathway | term | [illegible] | [illegible] | p | [illegible] |
| --- | --- | --- | --- | --- | --- |
| [illegible] | Protein digestion and absorption | [illegible] | [illegible] | [illegible] | [illegible] |
| [illegible] | GnRH secretion | [illegible] | [illegible] | [illegible] | [illegible] |
| [illegible] | Antigen processing and presentation | [illegible] | [illegible] | [illegible] | [illegible] |
| [illegible] | Viral myocarditis | [illegible] | [illegible] | [illegible] | [illegible] |
| [illegible] | Human papillomavirus infection | [illegible] | [illegible] | [illegible] | [illegible] |
| [illegible] | PI3K-Akt signaling pathway | [illegible] | [illegible] | [illegible] | [illegible] |
| [illegible] | Prostate cancer | [illegible] | [illegible] | [illegible] | [illegible] |
| [illegible] | Malaria | [illegible] | [illegible] | [illegible] | [illegible] |
| [illegible] | Allograft rejection | [illegible] | [illegible] | [illegible] | [illegible] |
| [illegible] | Graft-versus-host disease | [illegible] | [illegible] | [illegible] | [illegible] |
| [illegible] | Complement and coagulation cascades | [illegible] | [illegible] | [illegible] | [illegible] |
| [illegible] | Cell adhesion molecules | [illegible] | [illegible] | [illegible] | [illegible] |
| [illegible] | Autoimmune thyroid disease | [illegible] | [illegible] | [illegible] | [illegible] |
| [illegible] | Gastric cancer | [illegible] | [illegible] | [illegible] | [illegible] |
| [illegible] | Cushing syndrome | [illegible] | [illegible] | [illegible] | [illegible] |
| [illegible] | Type I diabetes mellitus | [illegible] | [illegible] | [illegible] | [illegible] |
| [illegible] | Taste transduction | [illegible] | [illegible] | [illegible] | [illegible] |
| [illegible] | Epstein-Barr virus infection | [illegible] | [illegible] | [illegible] | [illegible] |
| [illegible] | Inflammatory bowel disease | [illegible] | [illegible] | [illegible] | [illegible] |

| GO | term | [illegible] | [illegible] | [illegible] | p | [illegible] |
| --- | --- | --- | --- | --- | --- | --- |
| [illegible] | signal transduction | [illegible] | [illegible] | [illegible] | [illegible] | [illegible] |
| [illegible] | cell communication | [illegible] | [illegible] | [illegible] | [illegible] | [illegible] |
| [illegible] | signaling | [illegible] | [illegible] | [illegible] | [illegible] | [illegible] |
| [illegible] | extracellular region | [illegible] | [illegible] | [illegible] | [illegible] | [illegible] |
| [illegible] | immune system process | [illegible] | [illegible] | [illegible] | [illegible] | [illegible] |
| [illegible] | regulation of immune system process | [illegible] | [illegible] | [illegible] | [illegible] | [illegible] |
| [illegible] | immune response | [illegible] | [illegible] | [illegible] | [illegible] | [illegible] |
| [illegible] | positive regulation of cell proliferation | [illegible] | [illegible] | [illegible] | [illegible] | [illegible] |
| [illegible] | extracellular matrix | [illegible] | [illegible] | [illegible] | [illegible] | [illegible] |
| [illegible] | positive regulation of immune system process | [illegible] | [illegible] | [illegible] | [illegible] | [illegible] |
| [illegible] | antigen binding | [illegible] | [illegible] | [illegible] | [illegible] | [illegible] |
| [illegible] | positive regulation of response to stimulus | [illegible] | [illegible] | [illegible] | [illegible] | [illegible] |
| [illegible] | cellular response to stimulus | [illegible] | [illegible] | [illegible] | [illegible] | [illegible] |
| [illegible] | regulation of response to stimulus | [illegible] | [illegible] | [illegible] | [illegible] | [illegible] |
| [illegible] | cell surface receptor signaling pathway | [illegible] | [illegible] | [illegible] | [illegible] | [illegible] |
| [illegible] | collagen-containing extracellular matrix | [illegible] | [illegible] | [illegible] | [illegible] | [illegible] |
| [illegible] | defense response | [illegible] | [illegible] | [illegible] | [illegible] | [illegible] |
| [illegible] | growth factor binding | [illegible] | [illegible] | [illegible] | [illegible] | [illegible] |
| [illegible] | regulation of immune response | [illegible] | [illegible] | [illegible] | [illegible] | [illegible] |
| [illegible] | signaling receptor activity | [illegible] | [illegible] | [illegible] | [illegible] | [illegible] |
| [illegible] | multicellular organismal process | [illegible] | [illegible] | [illegible] | [illegible] | [illegible] |
| [illegible] | positive regulation of multicellular organismal process | [illegible] | [illegible] | [illegible] | [illegible] | [illegible] |
| [illegible] | [illegible] | [illegible] | [illegible] | [illegible] | [illegible] | [illegible] |

[illegible]
