# Supplementary material for: Genetic and pathway complexity in Alzheimer’s disease: Insights from multi-omic data about the immune response and mitochondrial function
Source: Neural Regen Res. 2025 Sep 29;21(9):4406–23. doi: 10.4103/NRR.NRR-D-25-00184 (PMC13557549; doi:10.4103/NRR.NRR-D-25-00184)
Supplement: Supplementary file 9 [file NRR-21-4406_Suppl17.pdf]

Additional Table 17 Results of mediation analysis of AD pathologic features and disease gene sets

ADNI

```
Indirect Path: "Abeta" (X) ==> "C8_HAY_BONE_MARROW_STROMAL" (M) ==> "AD" (Y)
──────────────────────────────────────────────────────────
                 Effect    S.E.       z      p       [Boot 95% CI]
──────────────────────────────────────────────────────────
Indirect (ab)   0.000 (0.000)   0.003  .998      [-0.000,  0.000]
Direct (c')    -0.000 (0.000) -13.140 <.001 *** [-0.000, -0.000]
──────────────────────────────────────────────────────────
Percentile Bootstrap Confidence Interval
(SE and CI are estimated based on 1000 Bootstrap samples.)

Running 1000 simulations...
Indirect Path: "Abeta" (X) ==> "H_HALLMARK_COMPLEMENT" (M) ==> "AD" (Y)
──────────────────────────────────────────────────────────
                 Effect    S.E.       z      p       [Boot 95% CI]
──────────────────────────────────────────────────────────
Indirect (ab)  -0.000 (0.000)  -0.184  .854      [-0.000,  0.000]
Direct (c')    -0.000 (0.000) -13.130 <.001 *** [-0.000, -0.000]
──────────────────────────────────────────────────────────
Percentile Bootstrap Confidence Interval
(SE and CI are estimated based on 1000 Bootstrap samples.)

Running 1000 simulations...
Indirect Path: "Abeta" (X) ==> "C3_ZFP91_TARGET_GENES" (M) ==> "AD" (Y)
──────────────────────────────────────────────────────────
                 Effect    S.E.       z      p       [Boot 95% CI]
──────────────────────────────────────────────────────────
Indirect (ab)  -0.000 (0.000)  -1.365  .172      [-0.000,  0.000]
Direct (c')    -0.000 (0.000) -13.134 <.001 *** [-0.000, -0.000]
──────────────────────────────────────────────────────────
Percentile Bootstrap Confidence Interval
(SE and CI are estimated based on 1000 Bootstrap samples.)
```

```
Indirect Path: "tau" (X) ==> "C8_HAY_BONE_MARROW_STROMAL" (M) ==> "AD" (Y)
──────────────────────────────────────────────────────────
                 Effect    S.E.       z      p       [Boot 95% CI]
──────────────────────────────────────────────────────────
Indirect (ab)   0.000 (0.000)  2.880  .004 **  [0.000, 0.000]
Direct (c')     0.000 (0.000) 12.734 <.001 *** [0.000, 0.001]
Total (c)       0.000 (0.000) 12.379 <.001 *** [0.000, 0.001]
──────────────────────────────────────────────────────────
Percentile Bootstrap Confidence Interval
```

```
Indirect Path: "ptau" (X) ==> "C8_HAY_BONE_MARROW_STROMAL" (M) ==> "AD" (Y)
──────────────────────────────────────────────────────────
                 Effect    S.E.       z      p       [Boot 95% CI]
──────────────────────────────────────────────────────────
Indirect (ab)   0.000 (0.000)  2.847  .004 **  [0.000, 0.001]
Direct (c')     0.005 (0.000) 16.674 <.001 *** [0.004, 0.006]
──────────────────────────────────────────────────────────
Percentile Bootstrap Confidence Interval
(SE and CI are estimated based on 1000 Bootstrap samples.)

Running 1000 simulations...
Indirect Path: "ptau" (X) ==> "C2_ZHANG_ADIPOGENESIS_BY_BMP7" (M) ==> "AD" (Y)
──────────────────────────────────────────────────────────
                 Effect    S.E.       z      p       [Boot 95% CI]
──────────────────────────────────────────────────────────
Indirect (ab)   0.000 (0.000)  0.912  .362      [-0.000, 0.000]
Direct (c')     0.005 (0.000) 17.738 <.001 *** [ 0.005, 0.006]
──────────────────────────────────────────────────────────
Percentile Bootstrap Confidence Interval
(SE and CI are estimated based on 1000 Bootstrap samples.)
```

ROSMAP

```
Indirect Path: "plaq_n" (X) ==> "C8_MANNO_MIDBRAIN_NEUROTYPES_HNBML1" (M) ==> "AD" (Y
──────────────────────────────────────────────────────────
                 Effect    S.E.       z      p       [Boot 95% CI]
──────────────────────────────────────────────────────────
Indirect (ab)   0.010 (0.003)  3.267  .001 **  [0.005, 0.017]
Direct (c')     0.195 (0.017) 11.232 <.001 *** [0.163, 0.230]
──────────────────────────────────────────────────────────
Percentile Bootstrap Confidence Interval
(SE and CI are estimated based on 1000 Bootstrap samples.)

Indirect Path: "plaq_n" (X) ==> "C3_ZFP91_TARGET_GENES" (M) ==> "AD" (Y)
──────────────────────────────────────────────────────────
                 Effect    S.E.       z      p       [Boot 95% CI]
──────────────────────────────────────────────────────────
Indirect (ab)   0.013 (0.004)  3.741 <.001 *** [0.007, 0.020]
Direct (c')     0.195 (0.017) 11.238 <.001 *** [0.162, 0.230]
──────────────────────────────────────────────────────────
Percentile Bootstrap Confidence Interval
(SE and CI are estimated based on 1000 Bootstrap samples.)

Running 1000 simulations...
Indirect Path: "plaq_n" (X) ==> "C4_GCM_MAP1B" (M) ==> "AD" (Y)
──────────────────────────────────────────────────────────
                 Effect    S.E.       z      p       [Boot 95% CI]
──────────────────────────────────────────────────────────
Indirect (ab)   0.003 (0.002)  1.649  .099 .   [0.000, 0.007]
Direct (c')     0.195 (0.017) 11.194 <.001 *** [0.163, 0.231]
──────────────────────────────────────────────────────────
Percentile Bootstrap Confidence Interval
(SE and CI are estimated based on 1000 Bootstrap samples.)

Indirect Path: "plaq_n" (X) ==> "C2_PETROVA_PROX1_TARGETS_UP" (M) ==> "AD" (Y)
──────────────────────────────────────────────────────────
                 Effect    S.E.       z      p       [Boot 95% CI]
──────────────────────────────────────────────────────────
Indirect (ab)   0.002 (0.001)  1.085  .278     [-0.001, 0.005]
Direct (c')     0.195 (0.017) 11.191 <.001 *** [ 0.163, 0.231]
──────────────────────────────────────────────────────────
Percentile Bootstrap Confidence Interval
```

```
Indirect Path: "nft" (X) ==> "C8_MANNO_MIDBRAIN_NEUROTYPES_HNBML1" (M) ==> "AD" (Y)
──────────────────────────────────────────────────────────
                 Effect    S.E.       z      p       [Boot 95% CI]
──────────────────────────────────────────────────────────
Indirect (ab)   0.009 (0.003)  3.081  .002 **  [0.004, 0.015]
Direct (c')     0.202 (0.017) 12.201 <.001 *** [0.170, 0.237]
──────────────────────────────────────────────────────────
Percentile Bootstrap Confidence Interval
(SE and CI are estimated based on 1000 Bootstrap samples.)

Indirect Path: "nft" (X) ==> "C5_GOBP_ANTIGEN_PROCESSING_AND_PRESENTATION_OF_EXOGENOUS_PEPTIDE_ANTIGEN" (M) ==> "AD"
──────────────────────────────────────────────────────────
                 Effect    S.E.       z      p       [Boot 95% CI]
──────────────────────────────────────────────────────────
Indirect (ab)   0.001 (0.001)  0.447  .655     [-0.002, 0.004]
Direct (c')     0.202 (0.017) 12.201 <.001 *** [ 0.170, 0.237]
──────────────────────────────────────────────────────────
Percentile Bootstrap Confidence Interval
(SE and CI are estimated based on 1000 Bootstrap samples.)

Running 1000 simulations...
Indirect Path: "nft" (X) ==> "C5_GOMF_MHC_CLASS_II_PROTEIN_COMPLEX_BINDING" (M) ==> "AD" (Y)
──────────────────────────────────────────────────────────
                 Effect    S.E.       z      p       [Boot 95% CI]
──────────────────────────────────────────────────────────
Indirect (ab)  -0.000 (0.001) -0.272  .786     [-0.003, 0.002]
Direct (c')     0.202 (0.017) 12.201 <.001 *** [ 0.170, 0.237]
──────────────────────────────────────────────────────────
Percentile Bootstrap Confidence Interval
(SE and CI are estimated based on 1000 Bootstrap samples.)

Indirect Path: "nft" (X) ==> "C3_MIR6794_5P" (M) ==> "AD" (Y)
──────────────────────────────────────────────────────────
                 Effect    S.E.       z      p       [Boot 95% CI]
──────────────────────────────────────────────────────────
Indirect (ab)   0.002 (0.001)  1.071  .284     [-0.000, 0.005]
Direct (c')     0.202 (0.017) 12.201 <.001 *** [ 0.170, 0.237]
──────────────────────────────────────────────────────────
Percentile Bootstrap Confidence Interval
(SE and CI are estimated based on 1000 Bootstrap samples.)

Running 1000 simulations...
Indirect Path: "nft" (X) ==> "C3_OCT1_Q5_01" (M) ==> "AD" (Y)
──────────────────────────────────────────────────────────
                 Effect    S.E.       z      p       [Boot 95% CI]
──────────────────────────────────────────────────────────
Indirect (ab)   0.002 (0.002)  1.043  .297     [-0.001, 0.005]
Direct (c')     0.202 (0.017) 12.201 <.001 *** [ 0.170, 0.237]
──────────────────────────────────────────────────────────
Percentile Bootstrap Confidence Interval
(SE and CI are estimated based on 1000 Bootstrap samples.)

Running 1000 simulations...
Indirect Path: "nft" (X) ==> "C6_E2F3_UP.V1_UP" (M) ==> "AD" (Y)
──────────────────────────────────────────────────────────
                 Effect    S.E.       z      p       [Boot 95% CI]
──────────────────────────────────────────────────────────
Indirect (ab)   0.003 (0.002)  1.733  .083 .   [0.000, 0.007]
Direct (c')     0.202 (0.017) 12.201 <.001 *** [0.170, 0.237]
──────────────────────────────────────────────────────────
Percentile Bootstrap Confidence Interval
(SE and CI are estimated based on 1000 Bootstrap samples.)

Running 1000 simulations...
Indirect Path: "nft" (X) ==> "C2_BANDRES_RESPONSE_TO_CARMUSTIN_WITHOUT_MGMT_48HR_UP" (M) ==> "AD" (Y)
──────────────────────────────────────────────────────────
                 Effect    S.E.       z      p       [Boot 95% CI]
──────────────────────────────────────────────────────────
Indirect (ab)  -0.001 (0.001) -1.165  .244     [-0.004, 0.001]
Direct (c')     0.202 (0.017) 12.201 <.001 *** [ 0.170, 0.237]
──────────────────────────────────────────────────────────
Percentile Bootstrap Confidence Interval
(SE and CI are estimated based on 1000 Bootstrap samples.)

Running 1000 simulations...
Indirect Path: "nft" (X) ==> "C8_CUI_DEVELOPING_HEART_C8_MACROPHAGE" (M) ==> "AD" (Y)
──────────────────────────────────────────────────────────
                 Effect    S.E.       z      p       [Boot 95% CI]
──────────────────────────────────────────────────────────
Indirect (ab)   0.005 (0.002)  2.253  .124     [0.001, 0.010]
Direct (c')     0.202 (0.017) 12.201 <.001 *** [0.170, 0.237]
──────────────────────────────────────────────────────────
Percentile Bootstrap Confidence Interval
(SE and CI are estimated based on 1000 Bootstrap samples.)

Running 1000 simulations...
Indirect Path: "nft" (X) ==> "C7_GSE17186_MEMORY_VS_CD21HIGH_TRANSITIONAL_BCELL_DN" (M) ==> "AD" (Y)
──────────────────────────────────────────────────────────
                 Effect    S.E.       z      p       [Boot 95% CI]
──────────────────────────────────────────────────────────
Indirect (ab)   0.003 (0.002)  1.621  .105     [0.000, 0.007]
Direct (c')     0.202 (0.017) 12.201 <.001 *** [0.170, 0.237]
──────────────────────────────────────────────────────────
Percentile Bootstrap Confidence Interval
(SE and CI are estimated based on 1000 Bootstrap samples.)

Running 1000 simulations...
Indirect Path: "nft" (X) ==> "C4_GNF2_MAPT" (M) ==> "AD" (Y)
──────────────────────────────────────────────────────────
                 Effect    S.E.       z      p       [Boot 95% CI]
──────────────────────────────────────────────────────────
Indirect (ab)   0.001 (0.001)  0.788  .431     [-0.001, 0.003]
Direct (c')     0.202 (0.017) 12.201 <.001 *** [ 0.170, 0.237]
──────────────────────────────────────────────────────────
Percentile Bootstrap Confidence Interval
(SE and CI are estimated based on 1000 Bootstrap samples.)

Running 1000 simulations...
Indirect Path: "nft" (X) ==> "C7_SCHERER_PBMC_APSV_WETVAX_AGE_18_48YO_5_TO_7DY_UP" (M) ==> "AD" (Y)
──────────────────────────────────────────────────────────
                 Effect    S.E.       z      p       [Boot 95% CI]
──────────────────────────────────────────────────────────
Indirect (ab)   0.000 (0.001)  0.197  .844     [-0.001, 0.002]
Direct (c')     0.202 (0.017) 12.201 <.001 *** [ 0.170, 0.237]
──────────────────────────────────────────────────────────
Percentile Bootstrap Confidence Interval
(SE and CI are estimated based on 1000 Bootstrap samples.)

Running 1000 simulations...
Indirect Path: "nft" (X) ==> "C5_GOBP_PEPTIDYL_LYSINE_ACETYLATION" (M) ==> "AD" (Y)
──────────────────────────────────────────────────────────
                 Effect    S.E.       z      p       [Boot 95% CI]
──────────────────────────────────────────────────────────
Indirect (ab)   0.003 (0.002)  1.649  .099 .   [0.000, 0.006]
Direct (c')     0.202 (0.017) 12.201 <.001 *** [0.170, 0.237]
──────────────────────────────────────────────────────────
Percentile Bootstrap Confidence Interval
(SE and CI are estimated based on 1000 Bootstrap samples.)

Running 1000 simulations...
Indirect Path: "nft" (X) ==> "C8_DURANTE_ADULT_OLFACTORY_NEUROEPITHELIUM_IMMATURE_NEURONS" (M) ==> "AD" (Y)
──────────────────────────────────────────────────────────
                 Effect    S.E.       z      p       [Boot 95% CI]
──────────────────────────────────────────────────────────
Indirect (ab)   0.004 (0.002)  1.839  .066 .   [0.000, 0.010]
Direct (c')     0.202 (0.017) 12.201 <.001 *** [0.170, 0.237]
──────────────────────────────────────────────────────────
Percentile Bootstrap Confidence Interval
(SE and CI are estimated based on 1000 Bootstrap samples.)

Running 1000 simulations...
Indirect Path: "nft" (X) ==> "C3_MIR8078" (M) ==> "AD" (Y)
──────────────────────────────────────────────────────────
                 Effect    S.E.       z      p       [Boot 95% CI]
──────────────────────────────────────────────────────────
Indirect (ab)   0.002 (0.001)  1.286  .198     [-0.000, 0.005]
Direct (c')     0.202 (0.017) 12.201 <.001 *** [ 0.170, 0.237]
──────────────────────────────────────────────────────────
Percentile Bootstrap Confidence Interval
(SE and CI are estimated based on 1000 Bootstrap samples.)

Running 1000 simulations...
Indirect Path: "nft" (X) ==> "H_HALLMARK_IL6_JAK_STAT3_SIGNALING" (M) ==> "AD" (Y)
──────────────────────────────────────────────────────────
                 Effect    S.E.       z      p       [Boot 95% CI]
──────────────────────────────────────────────────────────
Indirect (ab)   0.001 (0.001)  0.534  .594     [-0.001, 0.003]
Direct (c')     0.202 (0.017) 12.201 <.001 *** [ 0.170, 0.237]
──────────────────────────────────────────────────────────
Percentile Bootstrap Confidence Interval
(SE and CI are estimated based on 1000 Bootstrap samples.)

Running 1000 simulations...
Indirect Path: "nft" (X) ==> "C6_CAHOY_NEURONAL" (M) ==> "AD" (Y)
──────────────────────────────────────────────────────────
                 Effect    S.E.       z      p       [Boot 95% CI]
──────────────────────────────────────────────────────────
Indirect (ab)   0.003 (0.002)  1.445  .148     [-0.001, 0.007]
Direct (c')     0.202 (0.017) 12.201 <.001 *** [ 0.170, 0.237]
──────────────────────────────────────────────────────────
Percentile Bootstrap Confidence Interval
(SE and CI are estimated based on 1000 Bootstrap samples.)
```

```
Indirect Path: "amyloid" (X) ==> "C2_TOOKER_GEMCITABINE_RESISTANCE_UP" (M) ==> "AD" (Y)
──────────────────────────────────────────────────────────
                 Effect    S.E.       z      p       [Boot 95% CI]
──────────────────────────────────────────────────────────
Indirect (ab)   0.000 (0.000) 1.302  .193      [-0.000, 0.001]
Direct (c')     0.027 (0.003) 8.875 <.001 *** [ 0.021, 0.033]
──────────────────────────────────────────────────────────
Percentile Bootstrap Confidence Interval
(SE and CI are estimated based on 1000 Bootstrap samples.)

Running 1000 simulations...
Indirect Path: "amyloid" (X) ==> "C6_CAHOY_NEURONAL" (M) ==> "AD" (Y)
──────────────────────────────────────────────────────────
                 Effect    S.E.       z      p       [Boot 95% CI]
──────────────────────────────────────────────────────────
Indirect (ab)   0.001 (0.000) 2.253  .024 *   [0.000, 0.002]
Direct (c')     0.027 (0.003) 8.882 <.001 *** [0.021, 0.033]
──────────────────────────────────────────────────────────
Percentile Bootstrap Confidence Interval
(SE and CI are estimated based on 1000 Bootstrap samples.)

Running 1000 simulations...
Indirect Path: "amyloid" (X) ==> "C2_BANDRES_RESPONSE_TO_CARMUSTIN_WITHOUT_MGMT_48HR_UP" (M) ==> "AD" (Y)
──────────────────────────────────────────────────────────
                 Effect    S.E.       z      p       [Boot 95% CI]
──────────────────────────────────────────────────────────
Indirect (ab)   0.000 (0.000) 1.039  .299      [-0.000, 0.001]
Direct (c')     0.027 (0.003) 8.866 <.001 *** [ 0.021, 0.033]
──────────────────────────────────────────────────────────
Percentile Bootstrap Confidence Interval
(SE and CI are estimated based on 1000 Bootstrap samples.)

Running 1000 simulations...
Indirect Path: "amyloid" (X) ==> "C3_MIR6794_5P" (M) ==> "AD" (Y)
──────────────────────────────────────────────────────────
                 Effect    S.E.       z      p       [Boot 95% CI]
──────────────────────────────────────────────────────────
Indirect (ab)   0.001 (0.000) 1.741  .082 .   [0.000, 0.001]
Direct (c')     0.027 (0.003) 8.881 <.001 *** [0.021, 0.033]
──────────────────────────────────────────────────────────
Percentile Bootstrap Confidence Interval
(SE and CI are estimated based on 1000 Bootstrap samples.)
```

```
Indirect Path: "ptau" (X) ==> "C3_TCTAGAG_MIR517" (M) ==> "AD" (Y)
──────────────────────────────────────────────────────────
                 Effect    S.E.       z      p       [Boot 95% CI]
──────────────────────────────────────────────────────────
Indirect (ab)  -0.000 (0.000) -0.277  .782     [-0.000, 0.000]
Direct (c')     0.026 (0.002) 14.011 <.001 *** [ 0.023, 0.030]
──────────────────────────────────────────────────────────
Percentile Bootstrap Confidence Interval
(SE and CI are estimated based on 1000 Bootstrap samples.)

Running 1000 simulations...
Indirect Path: "ptau" (X) ==> "C7_GSE17186_MEMORY_VS_CD21HIGH_TRANSITIONAL_BCELL_DN" (M) ==> "AD" (Y)
──────────────────────────────────────────────────────────
                 Effect    S.E.       z      p       [Boot 95% CI]
──────────────────────────────────────────────────────────
Indirect (ab)   0.000 (0.000)  1.778  .075 .   [0.000, 0.001]
Direct (c')     0.026 (0.002) 13.978 <.001 *** [0.022, 0.030]
──────────────────────────────────────────────────────────
Percentile Bootstrap Confidence Interval
(SE and CI are estimated based on 1000 Bootstrap samples.)

Running 1000 simulations...
Indirect Path: "ptau" (X) ==> "C6_STK33_UP" (M) ==> "AD" (Y)
──────────────────────────────────────────────────────────
                 Effect    S.E.       z      p       [Boot 95% CI]
──────────────────────────────────────────────────────────
Indirect (ab)   0.001 (0.000)  3.312 <.001 *** [0.000, 0.001]
Direct (c')     0.026 (0.002) 13.899 <.001 *** [0.022, 0.030]
──────────────────────────────────────────────────────────
Percentile Bootstrap Confidence Interval
(SE and CI are estimated based on 1000 Bootstrap samples.)
```

```
Indirect Path: "plaq_d" (X) ==> "C2_BANDRES_RESPONSE_TO_CARMUSTIN_WITHOUT_MGMT_48HR_UP" (M) ==> "AD" (Y
──────────────────────────────────────────────────────────
                 Effect    S.E.       z      p       [Boot 95% CI]
──────────────────────────────────────────────────────────
Indirect (ab)   0.002 (0.002) 1.398  .162      [-0.000, 0.006]
Direct (c')     0.099 (0.019) 5.240 <.001 *** [ 0.065, 0.139]
──────────────────────────────────────────────────────────
Percentile Bootstrap Confidence Interval
(SE and CI are estimated based on 1000 Bootstrap samples.)

Running 1000 simulations...
Indirect Path: "plaq_d" (X) ==> "C8_ZHONG_PFC_MAJOR_TYPES_ASTROCYTES" (M) ==> "AD" (Y)
──────────────────────────────────────────────────────────
                 Effect    S.E.       z      p       [Boot 95% CI]
──────────────────────────────────────────────────────────
Indirect (ab)   0.005 (0.002) 2.264  .024 *   [0.002, 0.010]
Direct (c')     0.099 (0.019) 5.238 <.001 *** [0.065, 0.139]
──────────────────────────────────────────────────────────
Percentile Bootstrap Confidence Interval
(SE and CI are estimated based on 1000 Bootstrap samples.)

Running 1000 simulations...
Indirect Path: "plaq_d" (X) ==> "C3_MIR6794_5P" (M) ==> "AD" (Y)
──────────────────────────────────────────────────────────
                 Effect    S.E.       z      p       [Boot 95% CI]
──────────────────────────────────────────────────────────
Indirect (ab)   0.003 (0.002) 1.588  .112      [0.000, 0.007]
Direct (c')     0.099 (0.019) 5.239 <.001 *** [0.065, 0.139]
──────────────────────────────────────────────────────────
Percentile Bootstrap Confidence Interval
(SE and CI are estimated based on 1000 Bootstrap samples.)

Running 1000 simulations...
Indirect Path: "plaq_d" (X) ==> "C4_GNF2_AF1Q" (M) ==> "AD" (Y)
──────────────────────────────────────────────────────────
                 Effect    S.E.       z      p       [Boot 95% CI]
──────────────────────────────────────────────────────────
Indirect (ab)  -0.004 (0.003) -1.615  .106     [-0.010, 0.000]
Direct (c')     0.099 (0.019)  5.232 <.001 *** [ 0.065, 0.139]
──────────────────────────────────────────────────────────
Percentile Bootstrap Confidence Interval
(SE and CI are estimated based on 1000 Bootstrap samples.)
```
