# Supplementary material for: Genetic and pathway complexity in Alzheimer’s disease: Insights from multi-omic data about the immune response and mitochondrial function
Source: Neural Regen Res. 2025 Sep 29;21(9):4406–23. doi: 10.4103/NRR.NRR-D-25-00184 (PMC13557549; doi:10.4103/NRR.NRR-D-25-00184)
Supplement: Supplementary file 10 [file NRR-21-4406_Suppl18.pdf]

Additional Table 18 Important regulatory relationships in four cell-specific gene regulatory networks

Microglia

TG	TF	enhancer	promoter	TFb	coef	mse	group	coef_ab
[illegible]	[illegible]	[illegible]	[illegible]	[illegible]	[illegible]	[illegible]	[illegible]	[illegible]

Additional Table 18 Important regulatory relationships in four cell-specific gene regulatory networks

Inhibitory neurons

TG	TF	enhancer	promoter	TFb	coef	mse	group	coef_ab
GPR22	TBR1	chr7:106839639-106839826	chr7:107108002-107113002	enhancer	0.6908998	0.003595	CAHOY_NEURONAL	0.6909
KCNIP4	MEF2C	chr4:21964548-21967810	chr4:20881833-20886833	promoter	-0.66772	0.009676	CAHOY_NEURONAL	0.66772
HCN1	MEF2C	chr5:44826733-44836534	chr5:45693721-45698721	both	0.3467318	0.013238	CAHOY_NEURONAL	0.346732
SLC8A1	MEF2C	chr2:40129806-40130445	chr2:40654945-40659945	enhancer	-0.287294	0.07703	CAHOY_NEURONAL	0.287294
BCL11A	MEF2C	chr2:60290867-60292500	chr2:60778134-60783134	promoter	0.2322196	0.022751	CAHOY_NEURONAL	0.23222
CALN1	MEF2C	chr7:71264927-71265833	chr7:71874861-71879861	promoter	0.2046613	0.036185	CAHOY_NEURONAL	0.204661
MYT1L	MEF2C	chr2:2564208-2566060	chr2:1844159-1849159	promoter	0.1033351	0.008487	CAHOY_NEURONAL	0.103335
PPFIA2	MEF2C	chr12:81710318-81715089	chr12:81760474-81765474	promoter	0.1024365	0.011027	CAHOY_NEURONAL	0.102436
SYT1	MEF2C	chr12:78795524-78797813	chr12:79255273-79260273	promoter	0.0942695	0.043545	CAHOY_NEURONAL	0.094269
STXBP1	MEF2C	chr9:130440091-130468087	chr9:130371986-130376986	promoter	0.0469243	0.006471	CAHOY_NEURONAL	0.046924
NRXN1	NHLH1	chr2:50949961-50951523	chr2:50198805-50203805	enhancer	-4.767708	0.043234	DURANTE_ADULT_OLFACTORY_NEUROEPITHELIUM_IMMATURE_NEURONS	4.767708
NBEA	EBF1	chr13:34617681-34619039	chr13:36043147-36048147	promoter	2.432066	0.033332	DURANTE_ADULT_OLFACTORY_NEUROEPITHELIUM_IMMATURE_NEURONS	2.432066
FSTL5	EBF1	chr4:162682260-162682660	chr4:163082687-163087687	enhancer	-1.89659	0.100251	DURANTE_ADULT_OLFACTORY_NEUROEPITHELIUM_IMMATURE_NEURONS	1.89659
SPOCK1	EBF1	chr5:136730394-136732001	chr5:136831939-136836939	promoter	-1.763779	0.071614	DURANTE_ADULT_OLFACTORY_NEUROEPITHELIUM_IMMATURE_NEURONS	1.763779
MYT1L	NHLH1	chr2:2524250-2524796	chr2:1844159-1849159	enhancer	-1.597117	0.008487	DURANTE_ADULT_OLFACTORY_NEUROEPITHELIUM_IMMATURE_NEURONS	1.597117
SYT1	EBF1	chr12:78951954-78954452	chr12:79255273-79260273	enhancer	1.4620388	0.043545	DURANTE_ADULT_OLFACTORY_NEUROEPITHELIUM_IMMATURE_NEURONS	1.462039
TUBB2A	EBF1	chr6:3208230-3209680	chr6:3153983-3158983	promoter	-1.405988	0.009697	DURANTE_ADULT_OLFACTORY_NEUROEPITHELIUM_IMMATURE_NEURONS	1.405988
SYT1	NHLH1	chr12:78800092-78801747	chr12:79255273-79260273	enhancer	-1.37375	0.043545	DURANTE_ADULT_OLFACTORY_NEUROEPITHELIUM_IMMATURE_NEURONS	1.37375
UCHL1	EBF1	chr4:41688449-41689521	chr4:41256398-41261398	enhancer	-1.248115	0.015759	DURANTE_ADULT_OLFACTORY_NEUROEPITHELIUM_IMMATURE_NEURONS	1.248115
CALM1	EBF1	chr14:91046299-91047241	chr14:90860827-90865827	enhancer	-1.195119	0.017626	DURANTE_ADULT_OLFACTORY_NEUROEPITHELIUM_IMMATURE_NEURONS	1.195119
ENOX1	TEAD3	chr13:44007911-44008481	chr13:44201114-44206114	enhancer	6.4379502	0.034886	HAY_BONE_MARROW_STROMAL	6.43795
CHSY3	TEAD2	chr5:129500546-129500933	chr5:129238023-129243023	enhancer	-5.704476	0.028501	HAY_BONE_MARROW_STROMAL	5.704476
TENM2	TCF7L1	chr5:167646208-167646727	chr5:166709343-166714343	promoter	2.8380814	0.086602	HAY_BONE_MARROW_STROMAL	2.838081
FARP1	TEAD2	chr13:98992754-98993973	chr13:98792316-98797316	enhancer	2.5839949	0.006182	HAY_BONE_MARROW_STROMAL	2.583995
CNTN1	ZIC1	chr12:41212888-41213777	chr12:41083744-41088744	promoter	-2.458273	0.011767	HAY_BONE_MARROW_STROMAL	2.458273
CHSY3	ID4	chr5:129498090-129498613	chr5:129238023-129243023	enhancer	2.2274664	0.028501	HAY_BONE_MARROW_STROMAL	2.227466
LIMCH1	TCF7L1	chr4:41688449-41689521	chr4:41360304-41365304	enhancer	1.8088241	0.031376	HAY_BONE_MARROW_STROMAL	1.808824
FRMD4A	ZIC1	chr10:14214442-14216785	chr10:14370367-14375367	promoter	-1.75577	0.04787	HAY_BONE_MARROW_STROMAL	1.75577
PPFIA2	ID4	chr12:81952234-81952560	chr12:81760474-81765474	enhancer	-1.659024	0.011027	HAY_BONE_MARROW_STROMAL	1.659024
PMEPA1	TEAD2	chr20:56490059-56491420	chr20:56263181-56268181	enhancer	-1.64818	0.005605	HAY_BONE_MARROW_STROMAL	1.64818
LRRTM4	POU3F1	chr2:76902567-76904189	chr2:77747003-77752003	enhancer	-4.91165	0.104112	MANNO_MIDBRAIN_NEUROTYPES_HNBML1	4.91165
EDIL3	ONECUT2	chr5:83179404-83179526	chr5:83678112-83683112	promoter	4.1933977	0.029733	MANNO_MIDBRAIN_NEUROTYPES_HNBML1	4.193398
ZMAT4	POU3F2	chr8:40629353-40631306	chr8:40752844-40757844	enhancer	3.4439193	0.069566	MANNO_MIDBRAIN_NEUROTYPES_HNBML1	3.443919
ZMAT4	INSM1	chr8:40629353-40631306	chr8:40752844-40757844	enhancer	-3.394087	0.069566	MANNO_MIDBRAIN_NEUROTYPES_HNBML1	3.394087
HS6ST3	EBF1	chr13:97395685-97395979	chr13:96740593-96745593	enhancer	-3.08932	0.088342	MANNO_MIDBRAIN_NEUROTYPES_HNBML1	3.08932
NKAIN2	SOX4	chr6:124794292-124794504	chr6:124122569-124127569	promoter	2.911741	0.084312	MANNO_MIDBRAIN_NEUROTYPES_HNBML1	2.911741
BCL11A	INSM1	chr2:60290867-60292500	chr2:60778134-60783134	enhancer	2.6200921	0.022751	MANNO_MIDBRAIN_NEUROTYPES_HNBML1	2.620092
PEX5L	ONECUT2	chr3:179645158-179645321	chr3:179689509-179694509	promoter	-2.285795	0.025785	MANNO_MIDBRAIN_NEUROTYPES_HNBML1	2.285795
SEZ6L	ONECUT2	chr22:26176307-26176452	chr22:26685717-26690717	promoter	2.2258301	0.030448	MANNO_MIDBRAIN_NEUROTYPES_HNBML1	2.22583
SYN3	INSM1	chr22:33469512-33470746	chr22:33400310-33405310	enhancer	-2.029881	0.026661	MANNO_MIDBRAIN_NEUROTYPES_HNBML1	2.029881
DOCK4	MAFF	chr7:111393062-111398936	chr7:111377772-111382772	enhancer	-1.83345	0.014431	STK33_UP	1.833457
SCG2	TGIF1	chr2:223815232-223816415	chr2:224464718-224469718	both	-1.77418	0.023107	STK33_UP	1.774189
DUSP8	TGIF1	chr11:1567540-1570516	chr11:1590651-1595651	promoter	1.4418043	0.001709	STK33_UP	1.441804
ANKRD12	TGIF1	chr18:9116683-9117290	chr18:9134251-9139251	promoter	-1.37207	0.014442	STK33_UP	1.372078
SETBP1	EGR3	chr18:42928728-42929541	chr18:42257638-42262638	promoter	1.3551154	0.018282	STK33_UP	1.355115
ARID5B	TFEC	chr10:64156746-64157379	chr10:63658513-63663513	enhancer	1.1873927	0.008049	STK33_UP	1.187393
SCG2	JUN	chr2:223815232-223816415	chr2:224464718-224469718	enhancer	1.1455818	0.023107	STK33_UP	1.145582
PTPRO	EGR3	chr12:15539642-15541692	chr12:15472691-15477691	promoter	1.140433	0.029513	STK33_UP	1.140433
ARID5B	TGIF1	chr10:63763236-63765122	chr10:63658513-63663513	promoter	-1.08790	0.008049	STK33_UP	1.087903
SGK1	MAFF	chr6:135050320-135051728	chr6:134493500-134498500	promoter	1.0418059	0.015444	STK33_UP	1.041806
PBX1	CREB3L1	chr1:164759601-164764635	chr1:164526097-164531097	enhancer	-2.19836	0.046502	ZFP91_TARGET_GENES	2.198367
TNRC18	GATA3	chr7:5507079-5507602	chr7:5460678-5465678	enhancer	1.9687041	0.00506	ZFP91_TARGET_GENES	1.968704
PRICKLE1	GATA3	chr12:43314751-43316410	chr12:42874917-42879917	enhancer	1.8401021	0.006286	ZFP91_TARGET_GENES	1.840102
CACNA1A	FOXO4	chr19:13604309-13604976	chr19:13365866-13370866	enhancer	-1.70126	0.016953	ZFP91_TARGET_GENES	1.701265
LINGO1	ESRRB	chr15:78162758-78164729	chr15:77922210-77927210	enhancer	-1.63399	0.023071	ZFP91_TARGET_GENES	1.633994
BICD1	GATA3	chr12:32353456-32353609	chr12:32257685-32262685	enhancer	1.6077357	0.013514	ZFP91_TARGET_GENES	1.607736
DLG2	CREB3L1	chr11:83425558-83425893	chr11:83390969-83395969	promoter	1.4743861	0.017211	ZFP91_TARGET_GENES	1.474386
CACNA1A	ESRRB	chr19:13507141-13508496	chr19:13365866-13370866	enhancer	1.4733559	0.016953	ZFP91_TARGET_GENES	1.473356
CACNA1A	GATA3	chr19:13685556-13685776	chr19:13365866-13370866	enhancer	-1.31218	0.016953	ZFP91_TARGET_GENES	1.312185
UBASH3B	CREB3L1	chr11:122614389-122616258	chr11:122523898-122528898	enhancer	-1.31154	0.009829	ZFP91_TARGET_GENES	1.31154
SEZ6L	HES5	chr22:26176307-26176452	chr22:26562940-26567940	enhancer	1.6724804	0.030448	ZHONG_PFC_MAJOR_TYPES_ASTROCYTES	1.67248
CPNE5	HES5	chr6:36819171-36821039	chr6:36804721-36809721	promoter	1.3753207	0.002009	ZHONG_PFC_MAJOR_TYPES_ASTROCYTES	1.375321
DKK3	HES5	chr11:11983773-11984367	chr11:12027656-12032656	enhancer	1.2643258	0.007855	ZHONG_PFC_MAJOR_TYPES_ASTROCYTES	1.264326
SEZ6L	SOX8	chr22:26176307-26176452	chr22:26562940-26567940	promoter	1.1180067	0.030448	ZHONG_PFC_MAJOR_TYPES_ASTROCYTES	1.118007
BBS2	ID4	chr16:56742764-56743531	chr16:56551509-56556509	enhancer	-0.794267	0.002065	ZHONG_PFC_MAJOR_TYPES_ASTROCYTES	0.794267
NTRK2	SOX8	chr9:87652806-87660256	chr9:87280966-87285966	enhancer	0.6959488	0.009068	ZHONG_PFC_MAJOR_TYPES_ASTROCYTES	0.695949
NTRK2	SOX9	chr9:87452107-87452332	chr9:87280966-87285966	enhancer	0.6453429	0.009068	ZHONG_PFC_MAJOR_TYPES_ASTROCYTES	0.645343
NTRK2	ID4	chr9:87346603-87347008	chr9:87280966-87285966	enhancer	0.6262304	0.009068	ZHONG_PFC_MAJOR_TYPES_ASTROCYTES	0.62623
DKK3	MEIS1	chr11:11983773-11984367	chr11:12027656-12032656	enhancer	0.5388713	0.007855	ZHONG_PFC_MAJOR_TYPES_ASTROCYTES	0.538871
SDC3	HES5	chr1:31295728-31299041	chr1:31349088-31354088	promoter	0.4497181	0.001823	ZHONG_PFC_MAJOR_TYPES_ASTROCYTES	0.449718

Excitatory neurons

TG	TF	enhancer	promoter	TFb	coef	mse	group	coef_ab
SYT1	MEF2C	chr12:78556343-78557019	chr12:79255273-79260273	promoter	0.5464745	0.012049	CAHOY_NEURONAL	0.546475
CAMK2B	MEF2C	chr7:44483279-44489130	chr7:44362731-44367731	enhancer	0.3924428	0.017565	CAHOY_NEURONAL	0.392443
CCK	MEF2C	chr3:42470723-42471568	chr3:42302940-42307940	promoter	0.2856921	0.086487	CAHOY_NEURONAL	0.285692
GABRG2	MEF2C	chr5:161297078-161306392	chr5:161492148-161497148	promoter	0.1736153	0.014521	CAHOY_NEURONAL	0.173615
STXBP1	MEF2C	chr9:130440091-130468087	chr9:130371986-130376986	promoter	0.1614458	0.024155	CAHOY_NEURONAL	0.161446
MYT1L	MEF2C	chr2:2829656-2846635	chr2:1844159-1849159	promoter	0.130832	0.007719	CAHOY_NEURONAL	0.130832
KCNIP4	MEF2C	chr4:21964548-21967810	chr4:20881833-20886833	promoter	-0.116704	0.133947	CAHOY_NEURONAL	0.116704
HCN1	MEF2C	chr5:45639588-45642536	chr5:45693721-45698721	both	0.1014217	0.009352	CAHOY_NEURONAL	0.101422
CPNE5	MEF2C	chr6:36819171-36821039	chr6:36722678-36727678	enhancer	0.0806314	0.006598	CAHOY_NEURONAL	0.080631
CALN1	MEF2C	chr7:71275943-71276560	chr7:71874861-71879861	promoter	-0.071935	0.019709	CAHOY_NEURONAL	0.071935
SYT1	OLIG2	chr12:78557656-78558722	chr12:79255273-79260273	enhancer	-2.251281	0.012049	DURANTE_ADULT_OLFACTORY_NEUROEPITHELIUM_IMMATURE_NEURONS	2.251281
TUBA1A	EBF1	chr12:49563759-49567651	chr12:49580608-49585608	promoter	-1.751522	0.028015	DURANTE_ADULT_OLFACTORY_NEUROEPITHELIUM_IMMATURE_NEURONS	1.751522
CADPS	EBF1	chr3:62613544-62613911	chr3:62858565-62863565	promoter	-1.613054	0.03128	DURANTE_ADULT_OLFACTORY_NEUROEPITHELIUM_IMMATURE_NEURONS	1.613054
NRXN1	NHLH1	chr2:49831989-49832789	chr2:50198805-50203805	enhancer	-1.423633	0.029484	DURANTE_ADULT_OLFACTORY_NEUROEPITHELIUM_IMMATURE_NEURONS	1.423633
NCAM1	NHLH1	chr11:112482523-112485319	chr11:112829469-112834469	enhancer	0.8799918	0.010887	DURANTE_ADULT_OLFACTORY_NEUROEPITHELIUM_IMMATURE_NEURONS	0.879992
TUBB2A	EBF1	chr6:3205814-3205986	chr6:3153983-3158983	promoter	-0.7034	0.02129	DURANTE_ADULT_OLFACTORY_NEUROEPITHELIUM_IMMATURE_NEURONS	0.7034
SSBP2	EBF1	chr5:81521178-81521525	chr5:81044573-81049573	promoter	-0.688183	0.012575	DURANTE_ADULT_OLFACTORY_NEUROEPITHELIUM_IMMATURE_NEURONS	0.688183
NRXN1	OLIG2	chr2:49831989-49832789	chr2:50198805-50203805	enhancer	-0.622673	0.029484	DURANTE_ADULT_OLFACTORY_NEUROEPITHELIUM_IMMATURE_NEURONS	0.622673
NCAM1	INSM1	chr11:113016437-113016789	chr11:112829469-112834469	enhancer	-0.560474	0.010887	DURANTE_ADULT_OLFACTORY_NEUROEPITHELIUM_IMMATURE_NEURONS	0.560474
DAAM1	NHLH1	chr14:59894478-59895221	chr14:59652881-59657881	enhancer	-0.536727	0.004337	DURANTE_ADULT_OLFACTORY_NEUROEPITHELIUM_IMMATURE_NEURONS	0.536727
SORBS2	ZIC1	chr4:186621444-186621989	chr4:186875371-186880371	promoter	2.0717034	0.033315	HAY_BONE_MARROW_STROMAL	2.071703
PDE1A	TCF7L1	chr2:183181645-183185242	chr2:183289276-183294276	promoter	1.9986846	0.053797	HAY_BONE_MARROW_STROMAL	1.998685
ENOX1	ZIC1	chr13:43730720-43734013	chr13:44201114-44206114	enhancer	1.6292347	0.030866	HAY_BONE_MARROW_STROMAL	1.629235
NRXN3	ID4	chr14:78839205-78839791	chr14:78634216-78639216	enhancer	-1.534429	0.057347	HAY_BONE_MARROW_STROMAL	1.534429
LRRC4C	PPARG	chr11:39433443-39433584	chr11:40312181-40317181	promoter	-1.4518	0.126582	HAY_BONE_MARROW_STROMAL	1.4518
SLIT3	TEAD2	chr5:168414838-168415280	chr5:168725634-168730634	enhancer	1.3563205	0.017022	HAY_BONE_MARROW_STROMAL	1.356321
NRP1	ZIC1	chr10:34190002-34190294	chr10:33621269-33626269	promoter	1.3392318	0.006408	HAY_BONE_MARROW_STROMAL	1.339232
PDZRN4	TCF7L1	chr12:42299250-42301688	chr12:41579750-41584750	enhancer	-1.253058	0.059688	HAY_BONE_MARROW_STROMAL	1.253058
SLIT3	ID4	chr5:168414838-168415280	chr5:168725634-168730634	enhancer	-1.224054	0.017022	HAY_BONE_MARROW_STROMAL	1.224054
NFIB	GLIS2	chr9:13568855-13571665	chr9:14178298-14183298	enhancer	-1.18578	0.031476	HAY_BONE_MARROW_STROMAL	1.18578
PDE1A	ONECUT1	chr2:183181645-183185242	chr2:183384614-183389614	enhancer	4.1601342	0.053797	MANNO_MIDBRAIN_NEUROTYPES_HNBML1	4.160134
HS6ST3	ONECUT1	chr13:97409116-97409286	chr13:96740593-96745593	promoter	3.3068317	0.132259	MANNO_MIDBRAIN_NEUROTYPES_HNBML1	3.306832
SYN3	SOX4	chr22:33467065-33468926	chr22:33400310-33405310	enhancer	-2.797876	0.056538	MANNO_MIDBRAIN_NEUROTYPES_HNBML1	2.797876
DOCK4	ONECUT1	chr7:111393062-111398936	chr7:111843963-111848963	promoter	-2.58424	0.024435	MANNO_MIDBRAIN_NEUROTYPES_HNBML1	2.58424
UCHL1	NKX6-2	chr4:40911029-40911252	chr4:41256398-41261398	enhancer	-2.401128	0.062898	MANNO_MIDBRAIN_NEUROTYPES_HNBML1	2.401128
CTNNA2	SOX4	chr2:80086787-80087625	chr2:79409857-79414857	promoter	-2.192363	0.072713	MANNO_MIDBRAIN_NEUROTYPES_HNBML1	2.192363
SYN3	ONECUT1	chr22:33382248-33383161	chr22:33400310-33405310	enhancer	2.1204392	0.056538	MANNO_MIDBRAIN_NEUROTYPES_HNBML1	2.120439
STMN1	ONECUT1	chr1:26284624-26285331	chr1:26216599-26221599	promoter	-2.115601	0.047597	MANNO_MIDBRAIN_NEUROTYPES_HNBML1	2.115601
CTNNA2	ONECUT1	chr2:80086787-80087625	chr2:79737560-79742560	promoter	1.9432977	0.072713	MANNO_MIDBRAIN_NEUROTYPES_HNBML1	1.943298
DOCK4	EBF1	chr7:111393062-111398936	chr7:111422036-111427036	promoter	-1.895579	0.024435	MANNO_MIDBRAIN_NEUROTYPES_HNBML1	1.895579
ZEB2	MAFF	chr2:145154680-145171131	chr2:145275459-145280459	both	2.20988	0.017115	STK33_UP	2.209886
SETBP1	MAFF	chr18:42949747-42951103	chr18:42257638-42262638	promoter	1.4558275	0.016097	STK33_UP	1.455827
ATXN1	MAFF	chr6:16964807-16965568	chr6:16759222-16764222	promoter	-1.30531	0.010562	STK33_UP	1.305316
ZBTB18	TGIF1	chr1:244086259-244088998	chr1:244213061-244217061	promoter	-1.05400	0.011038	STK33_UP	1.054001
DOCK4	TGIF1	chr7:111393062-111398936	chr7:111377772-111382772	promoter	-0.90165	0.024435	STK33_UP	0.901651
JUN	MAFF	chr1:60172617-60173194	chr1:59247286-59252286	promoter	0.8688205	0.003721	STK33_UP	0.868821
NRP1	MAFF	chr10:34190002-34190294	chr10:33621269-33626269	promoter	0.6848205	0.006408	STK33_UP	0.68482
ATXN1	EGR2	chr6:16374962-16378454	chr6:16759222-16764222	enhancer	0.615223	0.010562	STK33_UP	0.615223
EGR3	MAFF	chr8:22860782-22865831	chr8:22547403-22552403	enhancer	-0.43197	0.002061	STK33_UP	0.431971
ZBTB18	EGR1	chr1:244086259-244088998	chr1:244209741-244214741	promoter	0.4315865	0.011038	STK33_UP	0.431586
NRXN3	GATA3	chr14:78839205-78839791	chr14:78634216-78639216	enhancer	-3.55520	0.057347	ZFP91_TARGET_GENES	3.555209
RGS12	TAL1	chr4:3418846-3420620	chr4:3292255-3297255	enhancer	-3.45041	0.01835	ZFP91_TARGET_GENES	3.450412
NFIB	PRDM1	chr9:13568855-13571665	chr9:14178298-14183298	promoter	-3.00733	0.031476	ZFP91_TARGET_GENES	3.007333
NRXN3	PRDM1	chr14:78839205-78839791	chr14:78634216-78639216	promoter	1.8059939	0.057347	ZFP91_TARGET_GENES	1.805994
KLHL5	CREB3L1	chr4:38940551-38940819	chr4:39061352-39066352	promoter	1.765327	0.008233	ZFP91_TARGET_GENES	1.765327
NFIB	RARA	chr9:13568855-13571665	chr9:14178298-14183298	both	-1.53695	0.031476	ZFP91_TARGET_GENES	1.536957
PBX1	PRDM1	chr1:164759601-164764635	chr1:164526097-164531097	both	-1.31212	0.017777	ZFP91_TARGET_GENES	1.312126
LCORL	CREB3L1	chr4:18580051-18580882	chr4:18020984-18025984	promoter	-1.16552	0.008887	ZFP91_TARGET_GENES	1.165526
CALN1	PRDM1	chr7:71275943-71276560	chr7:71799709-71804709	promoter	-1.05729	0.019709	ZFP91_TARGET_GENES	1.05729
NRXN3	ESRRB	chr14:78839205-78839791	chr14:79743182-79748182	promoter	-1.05603	0.057347	ZFP91_TARGET_GENES	1.056032
LRRTM3	SOX8	chr10:69280640-69280795	chr10:68683292-68688292	promoter	-0.959601	0.030631	ZHONG_PFC_MAJOR_TYPES_ASTROCYTES	0.959601
SEZ6L	ID4	chr22:26176307-26176452	chr22:26562940-26567940	enhancer	-0.936539	0.023941	ZHONG_PFC_MAJOR_TYPES_ASTROCYTES	0.936539
DKK3	SOX8	chr11:11985033-11986153	chr11:12027656-12032656	enhancer	-0.823472	0.027592	ZHONG_PFC_MAJOR_TYPES_ASTROCYTES	0.823472
HOPX	RFX4	chr4:57656546-57656753	chr4:57545373-57550373	promoter	-0.6697389	0.019385	ZHONG_PFC_MAJOR_TYPES_ASTROCYTES	0.669739
LRRN3	ID4	chr7:110417151-110417600	chr7:110728562-110733562	enhancer	0.5277392	0.007606	ZHONG_PFC_MAJOR_TYPES_ASTROCYTES	0.527739
NTRK2	SOX8	chr9:87652806-87660256	chr9:87280966-87285966	enhancer	-0.522958	0.008079	ZHONG_PFC_MAJOR_TYPES_ASTROCYTES	0.522958
SNX3	SOX8	chr6:108678227-108678553	chr6:108579965-108584965	promoter	-0.50838	0.00866	ZHONG_PFC_MAJOR_TYPES_ASTROCYTES	0.50838
DKK3	HES5	chr11:11983773-11984367	chr11:12027656-12032656	enhancer	-0.482115	0.027592	ZHONG_PFC_MAJOR_TYPES_ASTROCYTES	0.482115
HMGCS1	HES5	chr5:43202992-43203301	chr5:43311115-43316115	promoter	0.4691854	0.00464	ZHONG_PFC_MAJOR_TYPES_ASTROCYTES	0.469185
DKK3	MEIS1	chr11:11983773-11984367	chr11:12027656-12032656	enhancer	0.432166	0.027592	ZHONG_PFC_MAJOR_TYPES_ASTROCYTES	0.432166

Oligodendrocytes

TG	TF	enhancer	promoter	TFb	coef	mse	group	coef_ab
CNTNAP4	MEF2C	chr16:76520620-76521426	chr16:76308676-76313676	promoter	0.2339734	0.016318	CAHOY_NEURONAL	0.233973
SATB2	MEF2C	chr2:200468959-200469032	chr2:200318320-200323320	promoter	0.0645902	0.000423	CAHOY_NEURONAL	0.06459
SLC17A6	MEF2C	chr11:22799189-22801495	chr11:22357167-22362167	enhancer	-0.00238	2.06E-05	CAHOY_NEURONAL	0.002388
PPA1	NHLH1	chr10:72123225-72124752	chr10:71990691-71995691	enhancer	1.4620705	0.006769	DURANTE_ADULT_OLFACTORY_NEUROEPITHELIUM_IMMATURE_NEURONS	1.46207
PPA1	EBF1	chr10:72123225-72124752	chr10:71990691-71995691	enhancer	1.278958	0.006769	DURANTE_ADULT_OLFACTORY_NEUROEPITHELIUM_IMMATURE_NEURONS	1.278958
PPA1	INSM1	chr10:72123225-72124752	chr10:71990691-71995691	enhancer	1.080685	0.006769	DURANTE_ADULT_OLFACTORY_NEUROEPITHELIUM_IMMATURE_NEURONS	1.080685
RAB6A	NHLH1	chr11:73574270-73574625	chr11:73468639-73473639	enhancer	0.5711888	0.002123	DURANTE_ADULT_OLFACTORY_NEUROEPITHELIUM_IMMATURE_NEURONS	0.571188
PCDH9	EBF1	chr13:67441213-67441842	chr13:67475238-67480238	enhancer	0.375909	0.00581	DURANTE_ADULT_OLFACTORY_NEUROEPITHELIUM_IMMATURE_NEURONS	0.375909
DPYSL2	EBF1	chr8:26164242-26164674	chr8:26369209-26374209	enhancer	0.267971	0.009735	DURANTE_ADULT_OLFACTORY_NEUROEPITHELIUM_IMMATURE_NEURONS	0.267971
APBB1	INSM1	chr11:6402467-6402640	chr11:6415305-6420305	enhancer	0.185925	0.002546	DURANTE_ADULT_OLFACTORY_NEUROEPITHELIUM_IMMATURE_NEURONS	0.185925
APBB1	EBF1	chr11:6400700-6401670	chr11:6438145-6443145	promoter	-0.11443	0.002546	DURANTE_ADULT_OLFACTORY_NEUROEPITHELIUM_IMMATURE_NEURONS	0.11443
CBX4	EMX2	chr17:77951218-77952275	chr17:77810714-77815714	enhancer	0.0600504	0.000307	DURANTE_ADULT_OLFACTORY_NEUROEPITHELIUM_IMMATURE_NEURONS	0.06005
NCAM1	INSM1	chr11:112656120-112656976	chr11:112829469-112834469	enhancer	0.0599506	0.023626	DURANTE_ADULT_OLFACTORY_NEUROEPITHELIUM_IMMATURE_NEURONS	0.059951
CBX4	LHX2	chr17:77951218-77952275	chr17:77810714-77815714	enhancer	0.0448198	0.000307	DURANTE_ADULT_OLFACTORY_NEUROEPITHELIUM_IMMATURE_NEURONS	0.04482
CDKN2D	INSM1	chr19:10754321-10755934	chr19:10677132-10682132	enhancer	-0.034457	0.000211	DURANTE_ADULT_OLFACTORY_NEUROEPITHELIUM_IMMATURE_NEURONS	0.034457
EDIL3	EGR1	chr5:83578465-83591197	chr5:83678112-83683112	promoter	-2.692589	0.01501	HAY_BONE_MARROW_STROMAL	2.692589
CLDN11	FOSB	chr3:170529094-170529839	chr3:170134153-170139153	enhancer	-2.455504	0.011939	HAY_BONE_MARROW_STROMAL	2.455504
GPRC5B	TBX15	chr16:19782429-19783659	chr16:19881669-19886669	enhancer	2.0054451	0.007632	HAY_BONE_MARROW_STROMAL	2.005445
CLDN11	EGR1	chr3:170408614-170408879	chr3:170134153-170139153	promoter	-1.590521	0.011939	HAY_BONE_MARROW_STROMAL	1.590521
PDE1C	EGR1	chr7:32080647-32080814	chr7:32107967-32112967	promoter	-1.545235	0.009021	HAY_BONE_MARROW_STROMAL	1.545235
UNC5C	GLIS2	chr4:96347192-96350372	chr4:96467862-96472862	both	-1.508869	0.026884	HAY_BONE_MARROW_STROMAL	1.508869
LIMCH1	TCF7L1	chr4:41624280-41626362	chr4:41360304-41365304	enhancer	-1.42147	0.017504	HAY_BONE_MARROW_STROMAL	1.42147
TF	EGR1	chr3:133434574-133437541	chr3:133416711-133421711	enhancer	1.292988	0.014774	HAY_BONE_MARROW_STROMAL	1.292988
DLC1	PPARG	chr8:13102372-13103676	chr8:12988310-12993310	promoter	1.2594843	0.012321	HAY_BONE_MARROW_STROMAL	1.259484
FBXL7	TCF7L1	chr5:15807237-15809424	chr5:15497805-15502805	enhancer	1.256378	0.006499	HAY_BONE_MARROW_STROMAL	1.256378
DLC1	EGR1	chr8:13102372-13103676	chr8:12971254-12976254	promoter	1.231449	0.012321	HAY_BONE_MARROW_STROMAL	1.231449
EDIL3	PPARG	chr5:83578465-83591197	chr5:83678112-83683112	both	1.226915	0.01501	HAY_BONE_MARROW_STROMAL	1.226915
PEX5L	ONECUT2	chr3:179692342-179703709	chr3:179689509-179694509	promoter	2.1972236	0.030833	MANNO_MIDBRAIN_NEUROTYPES_HNBML1	2.197224
ANK3	EBF1	chr10:62054037-62054245	chr10:62147135-62152135	promoter	2.018422	0.025861	MANNO_MIDBRAIN_NEUROTYPES_HNBML1	2.018422
ANK3	ONECUT1	chr10:62054037-62054245	chr10:61839996-61844996	promoter	1.758693	0.025861	MANNO_MIDBRAIN_NEUROTYPES_HNBML1	1.758693
EDIL3	SOX4	chr5:83578465-83591197	chr5:83678112-83683112	enhancer	1.6023302	0.01501	MANNO_MIDBRAIN_NEUROTYPES_HNBML1	1.60233
RASGEF1B	ONECUT1	chr4:82645456-82646840	chr4:82390562-82395562	enhancer	1.339128	0.030263	MANNO_MIDBRAIN_NEUROTYPES_HNBML1	1.339128
EDIL3	EBF1	chr5:83591547-83593877	chr5:83678112-83683112	enhancer	1.221062	0.01501	MANNO_MIDBRAIN_NEUROTYPES_HNBML1	1.221062
ANK3	ONECUT2	chr10:62054037-62054245	chr10:61839996-61844996	promoter	0.9961302	0.025861	MANNO_MIDBRAIN_NEUROTYPES_HNBML1	0.99613
ROBO1	SOX4	chr3:78868801-78870168	chr3:78735423-78740423	enhancer	0.919926	0.00824	MANNO_MIDBRAIN_NEUROTYPES_HNBML1	0.919926
PHYHIPL	ONECUT1	chr10:60978707-60979007	chr10:60933848-60938848	enhancer	0.9129646	0.005906	MANNO_MIDBRAIN_NEUROTYPES_HNBML1	0.912965
EDIL3	INSM1	chr5:83591547-83593877	chr5:83678112-83683112	enhancer	0.911167	0.01501	MANNO_MIDBRAIN_NEUROTYPES_HNBML1	0.911168
ARFGEF3	ONECUT1	chr6:138525959-138528399	chr6:138480553-138485553	enhancer	0.8934628	0.006401	MANNO_MIDBRAIN_NEUROTYPES_HNBML1	0.893463
PHYHIPL	SOX4	chr10:60978707-60979007	chr10:60933848-60938848	promoter	0.8134561	0.005906	MANNO_MIDBRAIN_NEUROTYPES_HNBML1	0.813456
TTLL7	EGR1	chr1:84387913-84388075	chr1:84462334-84467334	promoter	4.007813	0.019861	STK33_UP	4.007813
TTLL7	EGR3	chr1:84387913-84388075	chr1:84462334-84467334	promoter	-2.54584	0.019861	STK33_UP	2.545849
TTLL7	MAFF	chr1:84387913-84388075	chr1:84415571-84420571	promoter	1.99818	0.019861	STK33_UP	1.998187
SGK1	EGR1	chr6:134627533-134634986	chr6:134493500-134498500	promoter	1.46375	0.013979	STK33_UP	1.463756
ZEB2	EGR1	chr2:145177408-145243530	chr2:145275459-145280459	promoter	1.44362	0.031242	STK33_UP	1.443628
DOCK4	EGR1	chr7:111757077-111759781	chr7:111377772-111382772	enhancer	1.43976	0.020131	STK33_UP	1.439766
CREB5	EGR3	chr7:28761559-28767898	chr7:28336440-28341440	enhancer	-1.33801	0.008621	STK33_UP	1.33801
ATXN1	EGR1	chr6:17031344-17034207	chr6:16759222-16764222	enhancer	1.28093	0.017546	STK33_UP	1.280933
ZEB2	MAFF	chr2:145177408-145243530	chr2:145275459-145280459	promoter	1.24988	0.031242	STK33_UP	1.249881
ATXN1	TGIF1	chr6:17031344-17034207	chr6:16759222-16764222	both	1.1898277	0.017546	STK33_UP	1.189828
SGK1	EGR3	chr6:134627533-134634986	chr6:134493500-134498500	promoter	1.17594	0.013979	STK33_UP	1.175948
ZEB2	EGR3	chr2:145177408-145243530	chr2:145272823-145277823	promoter	1.15926	0.031242	STK33_UP	1.159265
RERE	CREB3L1	chr1:8574883-8579625	chr1:8583345-8588345	promoter	3.6792627	0.028498	ZFP91_TARGET_GENES	3.679263
PTMA	E2F3	chr2:232526079-232540332	chr2:232571991-232576991	promoter	3.21242	0.041299	ZFP91_TARGET_GENES	3.212421
TSC22D4	RFX5	chr7:100066143-100067604	chr7:100074403-100079403	enhancer	-2.65522	0.015064	ZFP91_TARGET_GENES	2.655227
DLG2	CREB3L1	chr11:83966525-83981519	chr11:83390969-83395969	promoter	2.4966951	0.005683	ZFP91_TARGET_GENES	2.496695
PTMA	RARA	chr2:232526079-232540332	chr2:232570735-232575735	both	2.4463794	0.041299	ZFP91_TARGET_GENES	2.446379
ANLN	RFX5	chr7:36407450-36412603	chr7:36426932-36431932	enhancer	2.3459398	0.008945	ZFP91_TARGET_GENES	2.34594
PTMA	PRDM1	chr2:232526079-232540332	chr2:232570735-232575735	promoter	2.06932	0.041299	ZFP91_TARGET_GENES	2.069328
KCTD8	E2F3	chr4:44257979-44260355	chr4:44448325-44453325	promoter	1.578375	0.00704	ZFP91_TARGET_GENES	1.578375
PTMA	SRF	chr2:232526079-232540332	chr2:232570735-232575735	both	1.4184994	0.041299	ZFP91_TARGET_GENES	1.418499
AGPAT4	PRDM1	chr6:161577180-161577357	chr6:161692608-161697608	promoter	1.380751	0.007285	ZFP91_TARGET_GENES	1.380751
CECR2	CREB3L1	chr22:17849168-17851509	chr22:17838339-17843339	promoter	1.3605606	0.002586	ZFP91_TARGET_GENES	1.360561
CDK18	PRDM1	chr1:205476537-205495445	chr1:205489057-205494057	promoter	1.3162425	0.014349	ZFP91_TARGET_GENES	1.316242
TSC22D4	JUN	chr7:100066143-100067604	chr7:100074403-100079403	enhancer	2.2962238	0.015064	ZHONG_PFC_MAJOR_TYPES_ASTROCYTES	2.296224
MT3	JUN	chr16:56546426-56549897	chr16:56620767-56625767	enhancer	0.8261388	0.005636	ZHONG_PFC_MAJOR_TYPES_ASTROCYTES	0.826139
GPRC5B	RFX4	chr16:19580443-19581135	chr16:19881669-19886669	enhancer	0.6978776	0.007632	ZHONG_PFC_MAJOR_TYPES_ASTROCYTES	0.697878
PLEKHB1	JUN	chr11:73124846-73127886	chr11:73354723-73359723	promoter	0.6823807	0.005153	ZHONG_PFC_MAJOR_TYPES_ASTROCYTES	0.682381
APBB2	HEY1	chr4:40845962-40846806	chr4:40856673-40861673	enhancer	-0.66449	0.006717	ZHONG_PFC_MAJOR_TYPES_ASTROCYTES	0.664449
CBR1	RFX4	chr21:37456832-37462504	chr21:37439785-37444785	enhancer	0.6503126	0.009847	ZHONG_PFC_MAJOR_TYPES_ASTROCYTES	0.650313
LRP4	RFX4	chr11:47182450-47183172	chr11:46937674-46942674	promoter	0.57050	0.006999	ZHONG_PFC_MAJOR_TYPES_ASTROCYTES	0.570501
QKI	JUN	chr6:163557115-163559311	chr6:163833175-163838175	enhancer	0.568315	0.009535	ZHONG_PFC_MAJOR_TYPES_ASTROCYTES	0.568315
GLUL	SOX8	chr1:182111883-182114942	chr1:182352218-182357218	enhancer	0.4847866	0.00834	ZHONG_PFC_MAJOR_TYPES_ASTROCYTES	0.484787
GLUL	RFX4	chr1:182082639-182084885	chr1:182352218-182357218	enhancer	0.46022	0.00834	ZHONG_PFC_MAJOR_TYPES_ASTROCYTES	0.460222
LRP4	SOX8	chr11:47182450-47183172	chr11:46881985-46886985	promoter	0.435396	0.006999	ZHONG_PFC_MAJOR_TYPES_ASTROCYTES	0.43535
PLPP3	RFX4	chr1:57023103-57024866	chr1:57042758-57047758	enhancer	0.404417	0.008411	ZHONG_PFC_MAJOR_TYPES_ASTROCYTES	0.404417
