# Supplementary material for: Genetic and pathway complexity in Alzheimer’s disease: Insights from multi-omic data about the immune response and mitochondrial function
Source: Neural Regen Res. 2025 Sep 29;21(9):4406–23. doi: 10.4103/NRR.NRR-D-25-00184 (PMC13557549; doi:10.4103/NRR.NRR-D-25-00184)
Supplement: Supplementary file 12 [file NRR-21-4406_Suppl2.pdf]

Additional Table 2 Model performance of ten machine learning models for predicting AD risk.

ADNI

	metric	file_name	rand_forest	bag_tree	discrim_flexible	boost_tree	decision_tree	svm_rbf	nearest_neighbor	pls	mlp	logistic_reg
1	auc	ADNI-C2	0.693	0.69	0.685	0.652	0.631	0.628	0.62	0.609	0.606	0.591
2	acc	ADNI-C2	0.71	0.717	0.718	0.69	0.656	0.685	0.662	0.573	0.685	0.694
3	auc	ADNI-C3	0.641	0.644	0.639	0.623	0.643	0.646	0.616	0.639	0.639	0.646
4	acc	ADNI-C3	0.708	0.737	0.727	0.685	0.691	0.685	0.678	0.589	0.694	0.682
5	auc	ADNI-C4	0.616	0.604	0.537	0.624	0.557	0.61	0.601	0.615	0.614	0.603
6	acc	ADNI-C4	0.68	0.674	0.662	0.685	0.62	0.685	0.627	0.582	0.685	0.676
7	auc	ADNI-C5	0.734	0.706	0.696	0.698	0.646	0.642	0.694	0.651	0.658	0.66
8	acc	ADNI-C5	0.723	0.714	0.699	0.702	0.656	0.685	0.667	0.617	0.661	0.678
9	auc	ADNI-C6	0.63	0.604	0.588	0.556	0.564	0.618	0.592	0.603	0.589	0.598
10	acc	ADNI-C6	0.703	0.677	0.675	0.672	0.678	0.685	0.664	0.597	0.686	0.698
11	auc	ADNI-C7	0.621	0.599	0.548	0.618	0.528	0.601	0.625	0.601	0.556	0.601
12	acc	ADNI-C7	0.68	0.663	0.665	0.648	0.589	0.685	0.686	0.569	0.635	0.69
13	auc	ADNI-C8	0.608	0.595	0.551	0.577	0.574	0.594	0.568	0.59	0.582	0.587
14	acc	ADNI-C8	0.656	0.662	0.677	0.67	0.651	0.685	0.681	0.564	0.692	0.692
15	auc	ADNI-D	0.604	0.596	0.535	0.599	0.592	0.586	0.609	0.606	0.61	0.589
16	acc	ADNI-D	0.673	0.661	0.668	0.685	0.648	0.685	0.667	0.574	0.685	0.675
17	auc	ADNI-H	0.562	0.556	0.544	0.564	0.544	0.601	0.543	0.613	0.611	0.611
18	acc	ADNI-H	0.654	0.647	0.656	0.683	0.602	0.685	0.62	0.588	0.664	0.658

MSBB

	metric	file_name	rand_forest	discrim_flexible	svm_rbf	nearest_neighbor	boost_tree	mlp	bag_tree	logistic_reg	pls	decision_tree
1	auc	MSBB-C2	0.78	0.78	0.766	0.765	0.763	0.754	0.751	0.739	0.736	0.689
2	acc	MSBB-C2	0.759	0.758	0.766	0.754	0.758	0.753	0.74	0.737	0.666	0.687
3	auc	MSBB-C3	0.827	0.824	0.846	0.811	0.82	0.852	0.808	0.816	0.806	0.738
4	acc	MSBB-C3	0.78	0.784	0.791	0.772	0.77	0.79	0.767	0.765	0.709	0.72
5	auc	MSBB-C4	0.737	0.725	0.741	0.72	0.733	0.747	0.728	0.735	0.733	0.693
6	acc	MSBB-C4	0.717	0.716	0.683	0.709	0.722	0.723	0.712	0.715	0.677	0.69
7	auc	MSBB-C5	0.795	0.786	0.786	0.765	0.786	0.801	0.775	0.773	0.767	0.739
8	acc	MSBB-C5	0.756	0.743	0.683	0.745	0.757	0.761	0.744	0.73	0.702	0.703
9	auc	MSBB-C6	0.65	0.67	0.683	0.639	0.682	0.707	0.651	0.708	0.706	0.61
10	acc	MSBB-C6	0.683	0.701	0.683	0.681	0.687	0.712	0.686	0.716	0.636	0.686
11	auc	MSBB-C7	0.694	0.722	0.724	0.673	0.723	0.728	0.711	0.726	0.727	0.673
12	acc	MSBB-C7	0.719	0.724	0.683	0.697	0.723	0.726	0.725	0.727	0.668	0.71
13	auc	MSBB-C8	0.699	0.722	0.725	0.682	0.714	0.716	0.709	0.726	0.727	0.692
14	acc	MSBB-C8	0.723	0.724	0.683	0.711	0.724	0.722	0.718	0.727	0.668	0.714
15	auc	MSBB-D	0.733	0.709	0.716	0.714	0.735	0.713	0.724	0.712	0.713	0.67
16	acc	MSBB-D	0.717	0.709	0.683	0.707	0.712	0.712	0.72	0.713	0.673	0.68
17	auc	MSBB-H	0.725	0.715	0.736	0.733	0.729	0.747	0.72	0.717	0.717	0.67
18	acc	MSBB-H	0.722	0.727	0.737	0.723	0.727	0.732	0.722	0.732	0.663	0.69

Table notes：AD: Alzheimer's disease; ADNI: AD Neuroimaging Initiative; Mayo: Mayo Clinic AD Genetics Studies; MSBB: Mount Sinai Brain Bank; ROSMAP: Religious Orders Study and Rush Memory and Aging Project. The models are Random Forest (rand_forest), Flexible Discriminant Analysis (discrim_flexible), Support Vector Machine with Radial Basis Function kernel (svm_rbf), k-Nearest Neighbors (nearest_neighbor), Boosted Tree/Gradient Boosting Decision Tree (boost_tree), Multi-Layer Perceptron (mlp), Bagged Decision Tree (bag_tree), Logistic Regression (logistic_reg), Partial Least Squares regression (pls), and Decision Tree (decision_tree).

ROSMAP

	metric	file_name	rand_forest	nearest_neighbor	boost_tree	bag_tree	svm_rbf	pls	decision_tree	discrim_flexible	logistic_reg	mlp
1	auc	ROSMAP-C2	0.794	0.778	0.776	0.775	0.748	0.745	0.733	0.728	0.724	0.721
2	acc	ROSMAP-C2	0.713	0.706	0.693	0.69	0.638	0.646	0.673	0.688	0.694	0.718
3	auc	ROSMAP-C3	0.749	0.719	0.772	0.76	0.738	0.732	0.698	0.77	0.728	0.743
4	acc	ROSMAP-C3	0.707	0.72	0.719	0.712	0.638	0.644	0.702	0.731	0.728	0.72
5	auc	ROSMAP-C4	0.691	0.644	0.698	0.684	0.726	0.718	0.636	0.689	0.721	0.715
6	acc	ROSMAP-C4	0.722	0.693	0.668	0.693	0.638	0.655	0.628	0.681	0.708	0.695
7	auc	ROSMAP-C5	0.792	0.771	0.787	0.775	0.748	0.745	0.733	0.728	0.724	0.724
8	acc	ROSMAP-C5	0.699	0.7	0.699	0.697	0.638	0.646	0.673	0.688	0.701	0.688
9	auc	ROSMAP-C6	0.72	0.694	0.694	0.707	0.718	0.671	0.661	0.731	0.67	0.707
10	acc	ROSMAP-C6	0.68	0.667	0.65	0.657	0.661	0.616	0.634	0.682	0.663	0.662
11	auc	ROSMAP-C7	0.755	0.734	0.769	0.751	0.739	0.742	0.733	0.73	0.742	0.744
12	acc	ROSMAP-C7	0.732	0.702	0.734	0.718	0.638	0.67	0.708	0.711	0.692	0.7
13	auc	ROSMAP-C8	0.758	0.761	0.784	0.757	0.719	0.713	0.752	0.719	0.712	0.754
14	acc	ROSMAP-C8	0.734	0.739	0.753	0.716	0.638	0.646	0.744	0.707	0.702	0.727
15	auc	ROSMAP-D	0.682	0.65	0.693	0.679	0.716	0.713	0.653	0.662	0.701	0.709
16	acc	ROSMAP-D	0.663	0.67	0.707	0.666	0.638	0.612	0.721	0.654	0.716	0.722
17	auc	ROSMAP-H	0.673	0.684	0.686	0.667	0.683	0.683	0.608	0.579	0.658	0.67
18	acc	ROSMAP-H	0.676	0.697	0.678	0.643	0.638	0.639	0.631	0.598	0.63	0.638

Mayo

	metric	file_name	rand_forest	svm_rbf	logistic_reg	mlp	bag_tree	discrim_flexible	pls	nearest_neighbor	boost_tree	decision_tree
1	auc	MAYO-C2	0.846	0.844	0.839	0.838	0.837	0.832	0.83	0.827	0.811	0.784
2	acc	MAYO-C2	0.761	0.748	0.734	0.737	0.748	0.75	0.746	0.751	0.743	0.703
3	auc	MAYO-C3	0.805	0.794	0.764	0.797	0.8	0.792	0.762	0.796	0.786	0.775
4	acc	MAYO-C3	0.734	0.708	0.679	0.73	0.724	0.734	0.679	0.707	0.722	0.709
5	auc	MAYO-C4	0.737	0.751	0.677	0.715	0.739	0.73	0.673	0.731	0.595	0.733
6	acc	MAYO-C4	0.695	0.69	0.625	0.66	0.692	0.693	0.62	0.7	0.625	0.7
7	auc	MAYO-C5	0.832	0.81	0.798	0.821	0.829	0.825	0.797	0.82	0.812	0.809
8	acc	MAYO-C5	0.759	0.739	0.711	0.73	0.747	0.745	0.702	0.724	0.753	0.733
9	auc	MAYO-C6	0.65	0.683	0.708	0.707	0.651	0.67	0.706	0.639	0.682	0.61
10	acc	MAYO-C6	0.683	0.683	0.716	0.712	0.686	0.701	0.636	0.681	0.687	0.686
11	auc	MAYO-C7	0.767	0.781	0.771	0.768	0.769	0.774	0.771	0.774	0.759	0.751
12	acc	MAYO-C7	0.717	0.696	0.7	0.687	0.686	0.717	0.699	0.698	0.667	0.687
13	auc	MAYO-C8	0.721	0.676	0.673	0.726	0.725	0.741	0.669	0.714	0.704	0.693
14	acc	MAYO-C8	0.66	0.526	0.633	0.647	0.659	0.67	0.618	0.66	0.678	0.627
15	auc	MAYO-D	0.73	0.605	0.626	0.776	0.725	0.749	0.626	0.752	0.648	0.713
16	acc	MAYO-D	0.679	0.526	0.63	0.7	0.665	0.707	0.608	0.714	0.583	0.689
17	auc	MAYO-H	0.762	0.764	0.763	0.775	0.771	0.768	0.761	0.764	0.758	0.736
18	acc	MAYO-H	0.68	0.526	0.684	0.701	0.687	0.674	0.678	0.709	0.689	0.656
