# Supplementary material for: Genetic and pathway complexity in Alzheimer’s disease: Insights from multi-omic data about the immune response and mitochondrial function
Source: Neural Regen Res. 2025 Sep 29;21(9):4406–23. doi: 10.4103/NRR.NRR-D-25-00184 (PMC13557549; doi:10.4103/NRR.NRR-D-25-00184)
Supplement: Supplementary file 13 [file NRR-21-4406_Suppl20.pdf]

[illegible] ID	Description	Gene	R[illegible]	pvalue	padjust	qvalue	geneID	C[illegible]
[illegible]	fatty liver disease	[illegible]	[illegible]	[illegible]	[illegible]	[illegible]	[illegible]	[illegible]
[illegible]	connective tissue cancer	[illegible]	[illegible]	[illegible]	[illegible]	[illegible]	[illegible]	[illegible]
[illegible]	[illegible]	[illegible]	[illegible]	[illegible]	[illegible]	[illegible]	[illegible]	[illegible]
[illegible]	lung adenocarcinoma	[illegible]	[illegible]	[illegible]	[illegible]	[illegible]	[illegible]	[illegible]
[illegible]	musculoskeletal system cancer	[illegible]	[illegible]	[illegible]	[illegible]	[illegible]	[illegible]	[illegible]
[illegible]	lipid storage disease	[illegible]	[illegible]	[illegible]	[illegible]	[illegible]	[illegible]	[illegible]
[illegible]	lymphoma	[illegible]	[illegible]	[illegible]	[illegible]	[illegible]	[illegible]	[illegible]
[illegible]	atherosclerosis	[illegible]	[illegible]	[illegible]	[illegible]	[illegible]	[illegible]	[illegible]
[illegible]	prostate carcinoma	[illegible]	[illegible]	[illegible]	[illegible]	[illegible]	[illegible]	[illegible]
[illegible]	pancreatitis	[illegible]	[illegible]	[illegible]	[illegible]	[illegible]	[illegible]	[illegible]
[illegible]	lysosomal storage disease	[illegible]	[illegible]	[illegible]	[illegible]	[illegible]	[illegible]	[illegible]
[illegible]	pancreas disease	[illegible]	[illegible]	[illegible]	[illegible]	[illegible]	[illegible]	[illegible]
[illegible]	[illegible]	[illegible]	[illegible]	[illegible]	[illegible]	[illegible]	[illegible]	[illegible]
[illegible]	myeloproliferative neoplasm	[illegible]	[illegible]	[illegible]	[illegible]	[illegible]	[illegible]	[illegible]
[illegible]	myeloid leukemia	[illegible]	[illegible]	[illegible]	[illegible]	[illegible]	[illegible]	[illegible]
[illegible]	bone cancer	[illegible]	[illegible]	[illegible]	[illegible]	[illegible]	[illegible]	[illegible]
[illegible]	cell type benign neoplasm	[illegible]	[illegible]	[illegible]	[illegible]	[illegible]	[illegible]	[illegible]
[illegible]	acute myeloid leukemia	[illegible]	[illegible]	[illegible]	[illegible]	[illegible]	[illegible]	[illegible]
[illegible]	esophageal carcinoma	[illegible]	[illegible]	[illegible]	[illegible]	[illegible]	[illegible]	[illegible]
[illegible]	[illegible]	[illegible]	[illegible]	[illegible]	[illegible]	[illegible]	[illegible]	[illegible]
