# Supplementary material for: Genetic and pathway complexity in Alzheimer’s disease: Insights from multi-omic data about the immune response and mitochondrial function
Source: Neural Regen Res. 2025 Sep 29;21(9):4406–23. doi: 10.4103/NRR.NRR-D-25-00184 (PMC13557549; doi:10.4103/NRR.NRR-D-25-00184)
Supplement: Supplementary file 14 [file NRR-21-4406_Suppl3.pdf]

Additional Table 1 Modeled importance of each class of important gene collections in the MsigDB and Disease database

| ID | Variable | [illegible] | Gene |
|---|---|---|---|
| REACTOME_KERATAN_SULFATE_DEGRADATION | REACTOME_KERATAN_SULFATE_DEGRADATION | [illegible] | [illegible] |
| ERKERA_ANEUPLOIDY_DN | ERKERA_ANEUPLOIDY_DN | [illegible] | [illegible] |
| ZHANG_ADIPOGENESIS_BY_BMP7 | ZHANG_ADIPOGENESIS_BY_BMP7 | [illegible] | [illegible] |
| BANDRES_RESPONSE_TO_CARMUSTIN_WITHOUT_MGMT_48HR_UP | BANDRES_RESPONSE_TO_CARMUSTIN_WITHOUT_MGMT_48HR_UP | [illegible] | [illegible] |
| WP_UREA_CYCLE_AND_METABOLISM_OF_AMINO_GROUPS | WP_UREA_CYCLE_AND_METABOLISM_OF_AMINO_GROUPS | [illegible] | [illegible] |
| REACTOME_MEIOSIS | REACTOME_MEIOSIS | [illegible] | [illegible] |
| CERVERA_SDHB_TARGETS_1_UP | CERVERA_SDHB_TARGETS_1_UP | [illegible] | [illegible] |
| REACTOME_RHO_GTPASES_ACTIVATE_KTN1 | REACTOME_RHO_GTPASES_ACTIVATE_KTN1 | [illegible] | [illegible] |
| SA_REG_CASCADE_OF_CYCLIN_EXPR | SA_REG_CASCADE_OF_CYCLIN_EXPR | [illegible] | [illegible] |
| TOOKER_GEMCITABINE_RESISTANCE_UP | TOOKER_GEMCITABINE_RESISTANCE_UP | [illegible] | [illegible] |
| PETROVA_PROX1_TARGETS_UP | PETROVA_PROX1_TARGETS_UP | [illegible] | [illegible] |
| REACTOME_ASSEMBLY_OF_COLLAGEN_FIBRILS_AND_OTHER_MULTIMERIC_STRUCTURES | REACTOME_ASSEMBLY_OF_COLLAGEN_FIBRILS_AND_OTHER_MULTIMERIC_STRUCTURES | [illegible] | [illegible] |
| GILMORE_CORE_NFKB_PATHWAY | GILMORE_CORE_NFKB_PATHWAY | [illegible] | [illegible] |
| BIOCARTA_ARAP_PATHWAY | BIOCARTA_ARAP_PATHWAY | [illegible] | [illegible] |
| KEGG_TYPE_I_DIABETES_MELLITUS | KEGG_TYPE_I_DIABETES_MELLITUS | [illegible] | [illegible] |
| SASSON_RESPONSE_TO_GONADOTROPHINS_DN | SASSON_RESPONSE_TO_GONADOTROPHINS_DN | [illegible] | [illegible] |
| REACTOME_G_PROTEIN_MEDIATED_EVENTS | REACTOME_G_PROTEIN_MEDIATED_EVENTS | [illegible] | [illegible] |
| REACTOME_GABA_SYNTHESIS_RELEASE_REUPTAKE_AND_DEGRADATION | REACTOME_GABA_SYNTHESIS_RELEASE_REUPTAKE_AND_DEGRADATION | [illegible] | [illegible] |
| BIOCARTA_AKT_PATHWAY | BIOCARTA_AKT_PATHWAY | [illegible] | [illegible] |
| BIOCARTA_BCELLSURVIVAL_PATHWAY | BIOCARTA_BCELLSURVIVAL_PATHWAY | [illegible] | [illegible] |
| BIOCARTA_DEATH_PATHWAY | BIOCARTA_DEATH_PATHWAY | [illegible] | [illegible] |
| BIOCARTA_HSP27_PATHWAY | BIOCARTA_HSP27_PATHWAY | [illegible] | [illegible] |
| BIOCARTA_INSULIN_PATHWAY | BIOCARTA_INSULIN_PATHWAY | [illegible] | [illegible] |
| BIOCARTA_MTA3_PATHWAY | BIOCARTA_MTA3_PATHWAY | [illegible] | [illegible] |
| BREUHAHN_GROWTH_FACTOR_SIGNALING_IN_LIVER_CANCER | BREUHAHN_GROWTH_FACTOR_SIGNALING_IN_LIVER_CANCER | [illegible] | [illegible] |
| CHANG_POU5F1_TARGETS_UP | CHANG_POU5F1_TARGETS_UP | [illegible] | [illegible] |
| CHEN_LVAD_SUPPORT_OF_FAILING_HEART_UP | CHEN_LVAD_SUPPORT_OF_FAILING_HEART_UP | [illegible] | [illegible] |
| KYNG_ENVIRONMENTAL_STRESS_RESPONSE_UP | KYNG_ENVIRONMENTAL_STRESS_RESPONSE_UP | [illegible] | [illegible] |
| LE_SKI_TARGETS_UP | LE_SKI_TARGETS_UP | [illegible] | [illegible] |
| MURATA_VIRULENCE_OF_H_PILORI | MURATA_VIRULENCE_OF_H_PILORI | [illegible] | [illegible] |
| OLSSON_E2F3_TARGETS_DN | OLSSON_E2F3_TARGETS_DN | [illegible] | [illegible] |
| REACTOME_SENESCENCE_ASSOCIATED_SECRETORY_PHENOTYPE_SASP | REACTOME_SENESCENCE_ASSOCIATED_SECRETORY_PHENOTYPE_SASP | [illegible] | [illegible] |
| SIMBA_TRIT_TARGETS_UP | SIMBA_TRIT_TARGETS_UP | [illegible] | [illegible] |
| WP_ALANINE_AND_ASPARTATE_METABOLISM | WP_ALANINE_AND_ASPARTATE_METABOLISM | [illegible] | [illegible] |
| WP_DIFFERENTIATION_OF_WHITE_AND_BROWN_ADIPOCYTE | WP_DIFFERENTIATION_OF_WHITE_AND_BROWN_ADIPOCYTE | [illegible] | [illegible] |
| [illegible] | [illegible] | [illegible] | [illegible] |
