# Supplementary material for: Genetic and pathway complexity in Alzheimer’s disease: Insights from multi-omic data about the immune response and mitochondrial function
Source: Neural Regen Res. 2025 Sep 29;21(9):4406–23. doi: 10.4103/NRR.NRR-D-25-00184 (PMC13557549; doi:10.4103/NRR.NRR-D-25-00184)
Supplement: Supplementary file 16 [file NRR-21-4406_Suppl5.pdf]

Additional Table 9 Pathway/enrichment results for differential genes and important characterization genes based on Reactome

ADNI.all	comparison	direction	pathwayId	pathwayName	pValue	pValueAdjusted	genes
1	AD vs CT	Up	INFLAMMATORY RESPONSE	INFLAMMATORY RESPONSE	1.39E-06	7.94E-05	[illegible]
2	AD vs CT	Up	P53 PATHWAY	P53 PATHWAY	0.00035086	0.009776858	[illegible]
3	AD vs CT	Up	COMPLEMENT	COMPLEMENT	0.00058661	0.009776858	[illegible]
4	AD vs CT	Up	TNFA SIGNALING VIA NFKB	TNFA SIGNALING VIA NFKB	0.00107094	0.013386733	[illegible]
5	AD vs CT	Up	PROTEIN SECRETION	PROTEIN SECRETION	0.00223378	0.020599803	[illegible]
6	AD vs CT	Up	IL6 JAK STAT3 SIGNALING	IL6 JAK STAT3 SIGNALING	0.00247181	0.020599803	[illegible]
7	AD vs CT	Up	EPITHELIAL MESENCHYMAL TRANSITION	EPITHELIAL MESENCHYMAL TRANSITION	0.00436743	0.031193949	[illegible]
8	AD vs CT	Down	HEME METABOLISM	HEME METABOLISM	1.34E-14	6.71E-13	[illegible]
9	AD vs CT	Down	E2F TARGETS	E2F TARGETS	3.34E-13	8.35E-12	[illegible]
10	AD vs CT	Down	MYC TARGETS V1	MYC TARGETS V1	5.96E-12	9.93E-11	[illegible]
11	AD vs CT	Down	G2M CHECKPOINT	G2M CHECKPOINT	3.10E-10	3.88E-09	[illegible]
12	AD vs CT	Down	MYC TARGETS V2	MYC TARGETS V2	2.78E-09	2.74E-08	[illegible]
13	AD vs CT	Down	UNFOLDED PROTEIN RESPONSE	UNFOLDED PROTEIN RESPONSE	2.96E-08	2.13E-07	[illegible]
14	AD vs CT	Down	OXIDATIVE PHOSPHORYLATION	OXIDATIVE PHOSPHORYLATION	8.05E-08	5.73E-07	[illegible]
15	AD vs CT	Down	MTORC1 SIGNALING	MTORC1 SIGNALING	0.00203106	0.012694147	[illegible]
