# Supplementary material for: Genetic and pathway complexity in Alzheimer’s disease: Insights from multi-omic data about the immune response and mitochondrial function
Source: Neural Regen Res. 2025 Sep 29;21(9):4406–23. doi: 10.4103/NRR.NRR-D-25-00184 (PMC13557549; doi:10.4103/NRR.NRR-D-25-00184)
Supplement: Supplementary file 17 [file NRR-21-4406_Suppl6.pdf]

AdditionalTable 6 Comparison of biological functions between each category

GO	Cluster	group	ID	Description	GeneRatio	BgRatio	pvalue	p.adjust	qvalue	geneID
1	C2	C2	GO:0005201	extracellular matrix structural constituent	3月10日	165/18369	8.45E-05	0.00126102	0.00085116	1277/3918/5549
2	C2	C2	GO:0004693	cyclin-dependent protein serine/threonine kinase activity	2月10日	30/18369	0.00011309	0.00126102	0.00085116	1017/1019
3	C2	C2	GO:0097472	cyclin-dependent protein kinase activity	2月10日	30/18369	0.00011309	0.00126102	0.00085116	1017/1019
4	C2	C2	GO:0030332	cyclin binding	2月10日	34/18369	0.00014826	0.00126102	0.00085116	1017/1019
5	C2	C2	GO:0016538	cyclin-dependent protein serine/threonine kinase regulator activity	2月10日	50/18369	0.00032224	0.00219122	0.00064056	9134/1019
6	C2	C2	GO:0008201	heparin binding	2月10日	168/18369	0.00356371	0.02020371	0.00563008	3918/5549
7	C2	C2	GO:0015643	toxic substance binding	1月10日	10/18369	0.00543196	0.02520902	0.00702419	445
8	C2	C2	GO:0048407	platelet-derived growth factor binding	1月10日	11/18369	0.00597037	0.02520902	0.00702419	1277
9	C2	C2	GO:0005539	glycosaminoglycan binding	2月10日	232/18369	0.00668563	0.02520902	0.00702419	3918/5549
10	C2	C2	GO:0070492	oligosaccharide binding	1月10日	15/18369	0.00813798	0.02520902	0.00702419	4017
11	C2	C2	GO:0016641	oxidoreductase activity, acting on the CH-NH2 group of donors, oxygen as acceptor	1月10日	16/18369	0.00867838	0.02520902	0.00702419	4017
12	C2	C2	GO:1901681	sulfur compound binding	2月10日	260/18369	0.00889073	0.02520902	0.00702419	3918/5549
13	C2	C2	GO:0016638	oxidoreductase activity, acting on the CH-NH2 group of donors	1月10日	20/18369	0.01083736	0.02700792	0.00752345	4017
14	C2	C2	GO:0035173	histone kinase activity	1月10日	21/18369	0.01137644	0.02700792	0.00752345	1017
15	C2	C2	GO:0030021	extracellular matrix structural constituent conferring compression resistance	1月10日	22/18369	0.01191326	0.02700792	0.00752345	5549
16	C2	C2	GO:0106310	protein serine kinase activity	2月10日	368/18369	0.01378057	0.03103328	0.00884364	1017/1019
17	C2	C2	GO:0016831	carboxy-lyase activity	1月10日	34/18369	0.01836049	0.03672099	0.01023185	4953
18	C2	C2	GO:0004674	protein serine/threonine kinase activity	2月10日	421/18369	0.02087993	0.03963798	0.01098892	1017/1019
19	C2	C2	GO:0030020	extracellular matrix structural constituent conferring tensile strength	1月10日	41/18369	0.02210272	0.03955223	0.01102075	1277
20	C2	C2	GO:0005044	scavenger receptor activity	1月10日	48/18369	0.02583209	0.04182339	0.01165338	4017
21	C2	C2	GO:0016879	ligase activity, forming carbon-nitrogen bonds	1月10日	48/18369	0.02583209	0.04182339	0.01165338	445
22	C2	C2	GO:0016830	carbon-carbon lyase activity	1月10日	51/18369	0.02742648	0.04238637	0.01181048	4953
23	C2	C2	GO:0016597	amino acid binding	1月10日	55/18369	0.02954867	0.04368064	0.01217108	445
24	C2	C2	GO:0005507	copper ion binding	1月10日	62/18369	0.03325247	0.04710767	0.01312398	4017
