# Supplementary material for: Genetic and pathway complexity in Alzheimer’s disease: Insights from multi-omic data about the immune response and mitochondrial function
Source: Neural Regen Res. 2025 Sep 29;21(9):4406–23. doi: 10.4103/NRR.NRR-D-25-00184 (PMC13557549; doi:10.4103/NRR.NRR-D-25-00184)
Supplement: Supplementary file 18 [file NRR-21-4406_Suppl7.pdf]

Additional Table 7 Summary of genes in the set of significant features associated with AD pathology indicators

| ADNI | Gene | Coun | Name_Combined | Total_imp |
|---|---|---|---|---|
| 1 | COL1A1 | 4 | C2_CHEVSKA_SIMR_TARGETS_1_UP/C3_ZFP91_TARGET_GENES/C4_GNF2_CDH11/C8_HAY_BONE_MARROW_STROMAL | [illegible] |
| [illegible] | [illegible] | [illegible] | [illegible] | [illegible] |
