# Supplementary material for: Genetic and pathway complexity in Alzheimer’s disease: Insights from multi-omic data about the immune response and mitochondrial function
Source: Neural Regen Res. 2025 Sep 29;21(9):4406–23. doi: 10.4103/NRR.NRR-D-25-00184 (PMC13557549; doi:10.4103/NRR.NRR-D-25-00184)
Supplement: Supplementary file 19 [file NRR-21-4406_Suppl8.pdf]

Additional Table 8 Summary of a collection of disease genes significantly associated with AD pathology

Item	Occurences	Present In
Age.related.macular.degeneration	7	ADNI_tau, ADNI_ptau, ADNI_abeta, ROSMAP_ptau, ROSMAP_np, ROSMAP_abeta, ROSMAP_nft
Nephronophthisis	6	ADNI_tau, ADNI_ptau, ADNI_abeta, ROSMAP_ptau, ROSMAP_np, ROSMAP_abeta
Arrhythmogenic.right.ventricular.cardiomyopathy	5	ADNI_tau, ADNI_ptau, ROSMAP_ptau, ROSMAP_abeta, ROSMAP_nft
Charcot.Marie.Tooth.disease	5	ADNI_tau, ROSMAP_ptau, ROSMAP_np, ROSMAP_nft, ROSMAP_dp
Joubert.syndrome	5	ADNI_tau, ADNI_ptau, ROSMAP_ptau, ROSMAP_np, ROSMAP_nft
Retinitis.pigmentosa	5	ADNI_tau, ADNI_abeta, ROSMAP_ptau, ROSMAP_abeta, ROSMAP_dp
maturity.onset.diabetes.of.the.young	5	ADNI_tau, ADNI_ptau, ROSMAP_ptau, ROSMAP_np, ROSMAP_nft
Amyotrophic.lateral.sclerosis	4	ADNI_ptau, ADNI_abeta, ROSMAP_np, ROSMAP_dp
Cholangiocarcinoma	4	ADNI_ptau, ROSMAP_np, ROSMAP_abeta, ROSMAP_dp
Leigh.disease	4	ADNI_ptau, ROSMAP_abeta, ROSMAP_nft, ROSMAP_dp
Noo n.syndrome	4	ADNI_tau, ADNI_ptau, ROSMAP_ptau, ROSMAP_nft
Alopecia.areata	3	ADNI_abeta, ROSMAP_np, ROSMAP_dp
Breast.cancer	3	ADNI_ptau, ROSMAP_abeta, ROSMAP_dp
Common.variable.immunodeficiency	3	ROSMAP_np, ROSMAP_abeta, ROSMAP_dp
Ehlers.Danlos.syndrome	3	ADNI_tau, ADNI_ptau, ROSMAP_ptau
Mitochondrial.complex.I.deficiency	3	ROSMAP_np, ROSMAP_nft, ROSMAP_dp
Ovarian.cancer	3	ADNI_ptau, ADNI_abeta, ROSMAP_nft
Autism.spectrum.disorder	2	ROSMAP_nft, ROSMAP_dp
Bardet.Biedl.syndrome	2	ADNI_ptau, ROSMAP_abeta
Rheumatoid.arthritis	2	ROSMAP_nft, ROSMAP_dp
Right.atrial.isomerism	2	ADNI_tau, ROSMAP_ptau

Table notes： AD: Alzheimer’s disease; ADNI: AD Neuroimaging Initiative; Mayo: Mayo Clinic AD Genetics Studies; MSBB: Mount Sinai Brain Bank; ROSMAP: Religious Orders Study and Rush Memory and Aging Project.
